# Synthesis and cytotoxicity evaluation of novel 1,8-acridinedione derivatives bearing phthalimide moiety as potential antitumor agents

**DOI:** 10.1038/s41598-023-41970-0

**Published:** 2023-09-12

**Authors:** Hassan A. Khatab, Sherif F. Hammad, Esmail M. El‐Fakharany, Ahmed I. Hashem, Eman A. E. El-Helw

**Affiliations:** 1 Egypt-Japan University of Science and Technology (E-JUST), Alexandria, Egypt; 2 Chemistry Department, Faculty of Science, Ain Shams University, Cairo, 11566 Egypt; 3 Department of Pharmaceutical Chemistry, Faculty of Pharmacy, Helwan University, Cairo, Egypt; 4 Protein Research Department, Genetic Engineering and Biotechnology Research Institute GEBRI, City of Scientific Research and Technological Applications, New Borg El Arab, 21934 Alexandria Egypt

**Keywords:** Chemical biology, Chemistry

## Abstract

In this study, we aimed to develop hybrid antitumor compounds by synthesizing and characterizing novel *N*-substituted acrididine-1,8-dione derivatives, designed as hybrids of phthalimide and acridine-1,8-diones. We employed a three-step synthetic strategy and characterized all compounds using IR, ^1^H NMR, ^13^C NMR, and LC–MS. The cytotoxicity and antitumor activity of five compounds (**8c**, **8f**, **8h**, **8i**, and **8L**) against four cancer cell lines (H460, A431, A549, and MDA-MB-231) compared to human skin fibroblast cells were evaluated. Among the synthesized compounds, compound **8f** showed promising activity against skin and lung cancers, with favorable IC_50_ values and selectivity index. The relative changes in mRNA expression levels of four key genes (p53, TOP2B, p38, and EGFR) in A431 cells treated with the five synthesized compounds (**8c**, **8f**, **8h**, **8i**, and **8L**) were also investigated. Additionally, molecular docking studies revealed that compound **8f** exhibited high binding affinity with TOP2B, p38, p53, and EGFR, suggesting its potential as a targeted anticancer therapy. The results obtained indicate that *N*-substituted acrididine-1,8-dione derivatives have the potential to be developed as novel antitumor agents with a dual mechanism of action, and compound **8f** is a promising candidate for further investigation.

## Introduction

Cancer is a deadly and complex disease that affects millions of people worldwide. Despite substantial advancements in its diagnosis, treatment, and research, cancer remains a major health concern and the world's leading cause of death^1,2^. Any organ of the body can develop cancer, which has several potential causes, including genetics, lifestyle habits, and environmental factors^3^. Understanding the nature of cancer, its origins, and its effects on both people and society as a whole are crucial in this situation^4^. The 1,8-dioxodecahydroacridines, also known as acridine-1,8-diones, represent a significant class of nitrogen heterocyclic compounds that possess a 1,4-dihydropyridine parent nucleus^5^. Many 1,4-dihydropyridine class (1,4-DHP) drugs kill cells by blocking the P-glycoprotein pump and reversing multidrug resistance^6,7^. Furthermore, various DHP-derived compounds have been studied as multidrug resistant (MDR) modular and antitumor agents^8,9^. 1,4-DHPs are often more cytotoxic to cancer cells than to non-cancer cells^10,11^. In the domain of synthetic and medicinal chemistry, DHP-derived compounds exhibit dual attributes as potent pharmaceutically active agents and multifaceted active intermediates, showcasing their significant role in drug development and molecular synthesis^12^. They are used as blockers of calcium channels and have a broad range of biological activities, including antimicrobial^13,14^, antimalarial^15,16^, antitumor^17^, antibacterial^18,19^, antifungals^20^, and DNA binding abilities. Their compounds have been utilized in therapy to treat cardiovascular disorders^21^ and cancer^17^. Acridine-1,8-diones are also useful as promoters^22,23^, photo sensitizers, and laser dyes^22,24,25^. Due to the fact that even small modifications to the 1,4-dihydropyridine (DHP) moiety can have significant effects on pharmacology^26,27^, the synthetic community has shown renewed interest in this core structure^28^.

**Figure 1:**
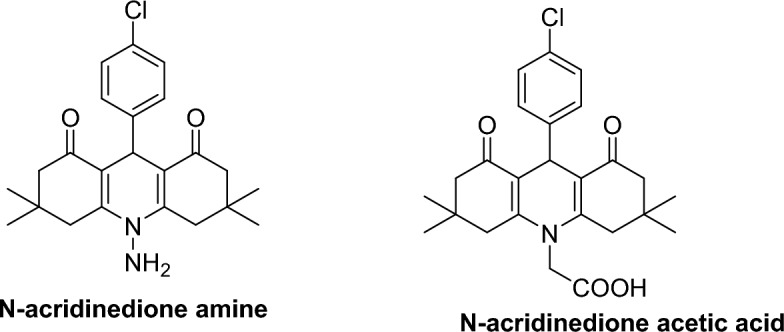
Clinically significant compounds of 1,8-acridinedione structure.

The broad spectrum of biological activity suggests that 1,8-acridinediones may hold potential as a therapeutic agent for various pathological conditions. Thus, acridinedione N-acetic acid (Fig. 1) is efficient against cancer proliferation through DNA binding^29^. Also, N-aminoacridinedione (Fig. 1) acts as an anticancer agent.

**Figure 2:**
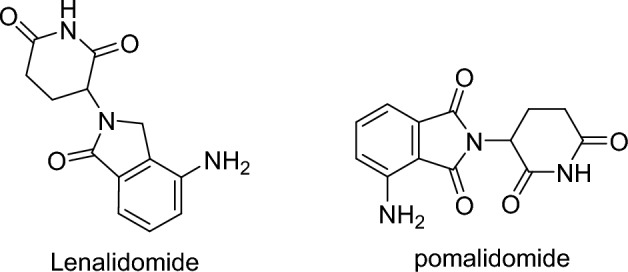
Phthalimide derivatives as significant drugs.

Phthalimide is a crucial starting material for a variety of biologically active compounds^30^. Numerous compounds having a phthalimide moiety with fascinating biological properties have been created. Among them, a significant biological property is the suppression of tumor necrosis factor-α (TNF-α) production^31,32^. Noteworthy, lenalidomide and pomalidomide, which are phthalimide derivatives, are used to treat multiple myeloma, a few other cancers, as well as leprosy and a few autoimmune illnesses (Fig. 2)^33^.

The reported diverse biological activities of acridinediones and phthalimide derivatives, coupled with our scope in the synthesis of biologically active heterocycles, initiated our interest to synthesize some acridine1,8-dione derivatives bearing a phthalimide moiety (**8a–L**). The activity of these derivatives as anticancer agents was also studied.

### Rationale and design

In our pursuit to develop potential antitumor compounds, inspiration was drawn from mitoxantrone's pharmacophoric features, which have demonstrated efficacy in treating advanced prostate cancer, acute nonlymphocytic leukemia, and certain forms of multiple sclerosis. To create a new series of novel *N*-substituted acrididine-1,8-dione derivatives, the fundamental pharmacophoric features of mitoxantrone were retained and integrated a phthalimide moieties, which are as follows:

- A planar polyaromatic moiety (chromophore) was maintained in the design, as it has been associated with antitumor activity.
- In order to form more hydrogen bonds during interaction with tumor proteins, the linker was modified by incorporating heteroatoms like oxygen (O) and nitrogen (N). Additionally, ester groups were introduced into the linker to further enhance hydrogen bonding capabilities.
- To boost the antitumor activity of the compounds, a phthalimide moiety was integrated into the design, which has been shown to have promising effects in this regard.
- We opted for aldehyde derivatives with various substituents to promote enhanced interactions with the target proteins.

**Figure 3:**
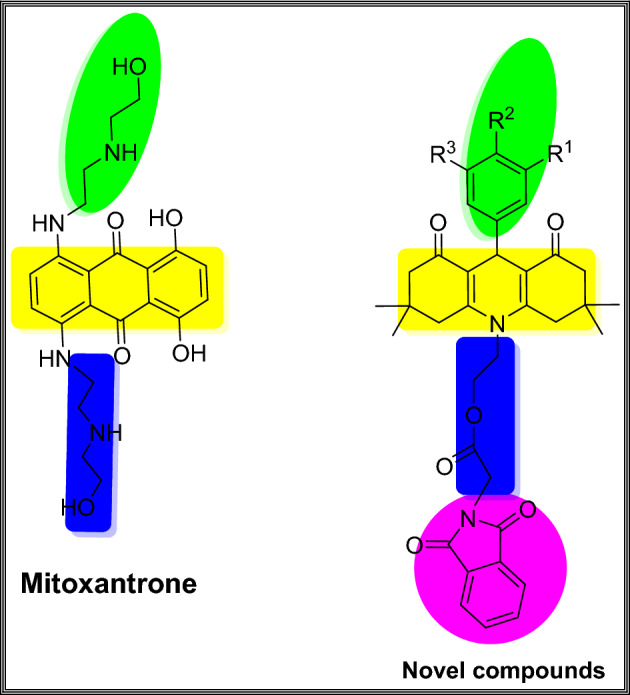
Chemical structures of Mitoxantrone and the target compounds.

By combining these features, we aim to create novel *N*-substituted acrididine-1,8-dione derivatives with improved antitumor properties. This rationale-based design holds the potential for the development of promising antitumor compounds with the desired therapeutic effects (Fig. 3).

## Results and discussion

### Chemistry

**Scheme 1:**
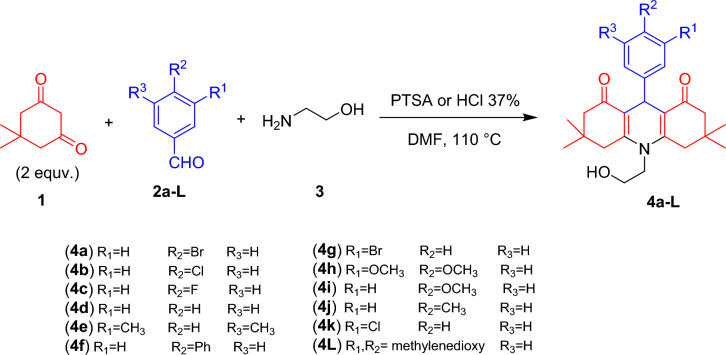
Synthesis of some acridine-1,8-diones **4a–L**.

Continuous research is required to create new chemicals in order to treat people with cancer effectively because of the urgent need for highly selective innovative anticancer medications^34^. Molecular hybridization, which involves joining the biologically active moieties of several molecules, is a potential technique for creating novel structures with more biological activity than their precursors^35^. Thus, in continuation to our research for new heterocycles of anticancer activity^36–45^, this work disclosed the synthesis of acrididine-1,8-diones **4a–L** using Hantzsch method by reacting dimedone **1**, various aromatic aldehydes **2a–L** and 2-aminoethanol **3** in dimethylformamide including PTSA (*p*-toluenesulfonic acid) or HCl (37%) (Scheme 1). This process was easy to carry out, and the yields of the desired products were high. The chemical structures of **4a–L** were supported by their analytical and spectral data. For example, the structure of the 1,8-acridinedione derivative **4a** was substantiated from its analytical and spectral data. Thus, the infrared spectrum showed absorption broad band at 3300–3600cm^−1^ characteristic of the O–H stretching vibrations and strong band at 1629 cm^**−**1^ due to the conjugated C=O groups. The ^1^H NMR spectrum of compound **4a** in DMSO-*d*_6_ exhibited characteristic peaks at δ = 0.87 and 1.01 ppm, assigned to the protons of the four methyl groups of the dimedone moieties. Four doublet peaks at δ = 2.02, 2.14, 2.44, and 2.72 ppm were assigned to the protons of the four methylene groups (CH_2_) of acridinedione. A sharp singlet peak at δ = 4.94 ppm was assigned to the methine (CH) acridinedione-H9. Additionally, a sharp singlet peak at δ = 5.04 ppm, corresponding to the D_2_O-exchangeable O–H proton, was also observed. The four aromatic protons appeared as two doublets at δ = 7.16 and 7.30 ppm. The ^13^C NMR spectrum of compound **4a** in DMSO-*d*_6_ was analyzed using Distortion-less Enhancement by Polarization Transfer (DEPT-135) to confirm its structure. The spectrum displayed fifteen peaks, representing all types of carbon atoms present in the compound. The DEPT-135 overlay indicated the absence of C=O carbon atoms and quaternary carbon atoms, which were observed in the ^13^C NMR spectrum at δ = 195.63, 152.40, 113.60, 32.48, and 146.16 & 118.95 ppm, respectively. The methylene carbon atoms were observed with negative signals in the DEPT-135 spectrum, which were observed in the ^13^C NMR spectrum at δ = 61.30, 49.93, 46.61, and 39.68 ppm. The DEPT-135 overlay also showed the presence of methine and methyl carbon atoms as positive signals. Specifically, methine carbon of the acridinedione ring appeared at δ = 31.75 ppm, while the carbon atoms of the four CH_3_ groups resonated at δ = 29.29 and 27.36 ppm. More, its LCMS analysis revealed the presence of one major compound with retention times of 0.67 min, corresponding to *m/z* values of 474 and expected MS (ESI +) *m/z* for C_25_H_30_BrNO_3_ [M + H]^+^: 474.14.

**Scheme 2:**
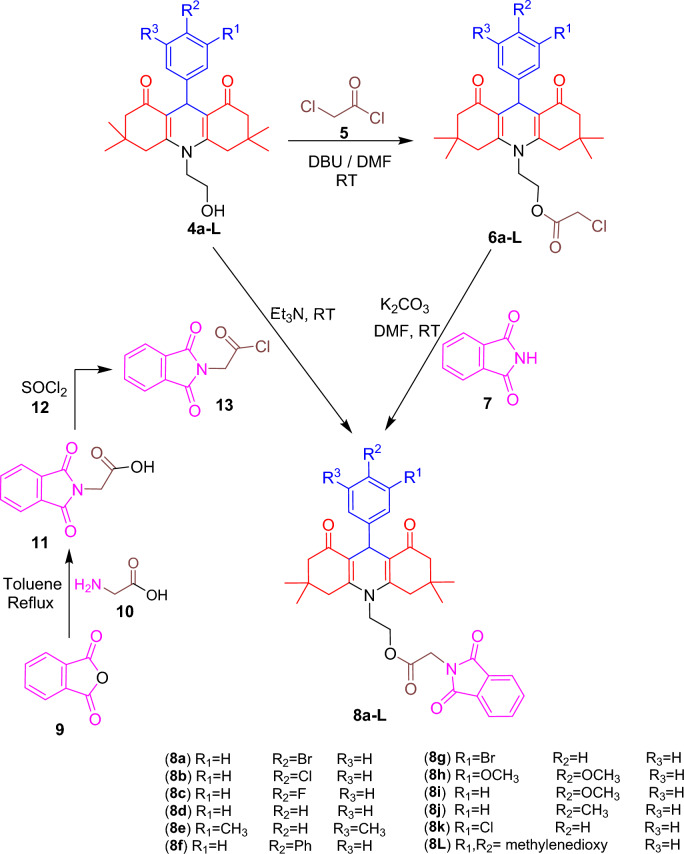
Synthesis of some acridinedione phthalimide derivatives **8a–L**.

The insertion of phthalimide moiety to acridinediones **4a–L** backbone was performed through two steps (cf. Scheme 2). Two different routes of synthesis were used to produce the compounds **8a–L**. In the first route, method A, compounds **4a–L** were treated with chloroacetyl chloride **5** in the presence of catalytic amount of DBU and then reacted with phthalimide** 7** in the presence of catalyst K_2_CO_3_ to yield the final product**s 8a–L**. In the second route, method B, phthalic anhydride **9** was treated with glycine **10** to give N-carboxymethyl phthalimide **11**. The latter was treated with thionyl chloride **12** to give the corresponding acid **13** which was coupled with **4a–L** to afford the desired products (**8a–L**) in the presence of catalyst triethyl amine.

Compound **6a** was subjected to various spectroscopic techniques to confirm its structural identity. The IR spectrum of compound **6a** showed the appearance of carbonyl stretching bands at 1754 cm^−1^ and 1626 cm^**−**1^, which correspond to the ester and conjugated ketone (acridinedione) carbonyls, respectively. The ^1^H-NMR spectrum showed the disappearance of the hydroxyl proton and the appearance of a new singlet peak at 4.18 ppm, which was assigned to the methylene protons of the acetate group (CH_2_–Cl). The ^13^C NMR spectrum displayed the appearance of a new peak at 167.53 ppm, assigned to the carbonyl carbon of the chloroacetate group. Additionally, the LCMS analysis revealed the presence of one major compound with a retention time of 0.68 min and m/z values of 550 (ESI+) corresponding to the expected molecular formula of C_27_H_31_BrClNO_4_ [M + H]^+^ at 550.11. These results confirm the successful formation of the acridinedione 2-chloroacetate derivative, compound **6a**.

The structure of compound **8e** was elucidated from its spectral data. The IR spectrum showed the presence of carbonyl groups at 1760 cm^**−**1^ (C=O, ester), 1726 cm^−1^ (C=O, phthalimide), and 1631 cm^**−**1^ (C=O, conjugated ketone, acridinedione), while the ^1^H NMR spectrum showed multiplet peaks related to the four protons of the aromatic ring of phthalimide at 7.90–7.98 ppm. The ^13^C NMR spectrum showed the appearance of peaks related to two carbonyl groups of acridinedione at 195.81 ppm, two carbonyl groups of phthalimide at 168.13 and 169.73 ppm, and one carbonyl group of ester at 167.51 ppm. The LCMS analysis confirmed the molecular weight of the compound as C_37_H_40_N_2_O_6_ with an expected [M + H]^+^ of 609.29 and a retention time of 0.68 min and m/z values of 609. These results suggest that compound **8e** contains phthalimide, acridinedione, and ester functional groups.

### Antitumor activity

**Figure 4:**
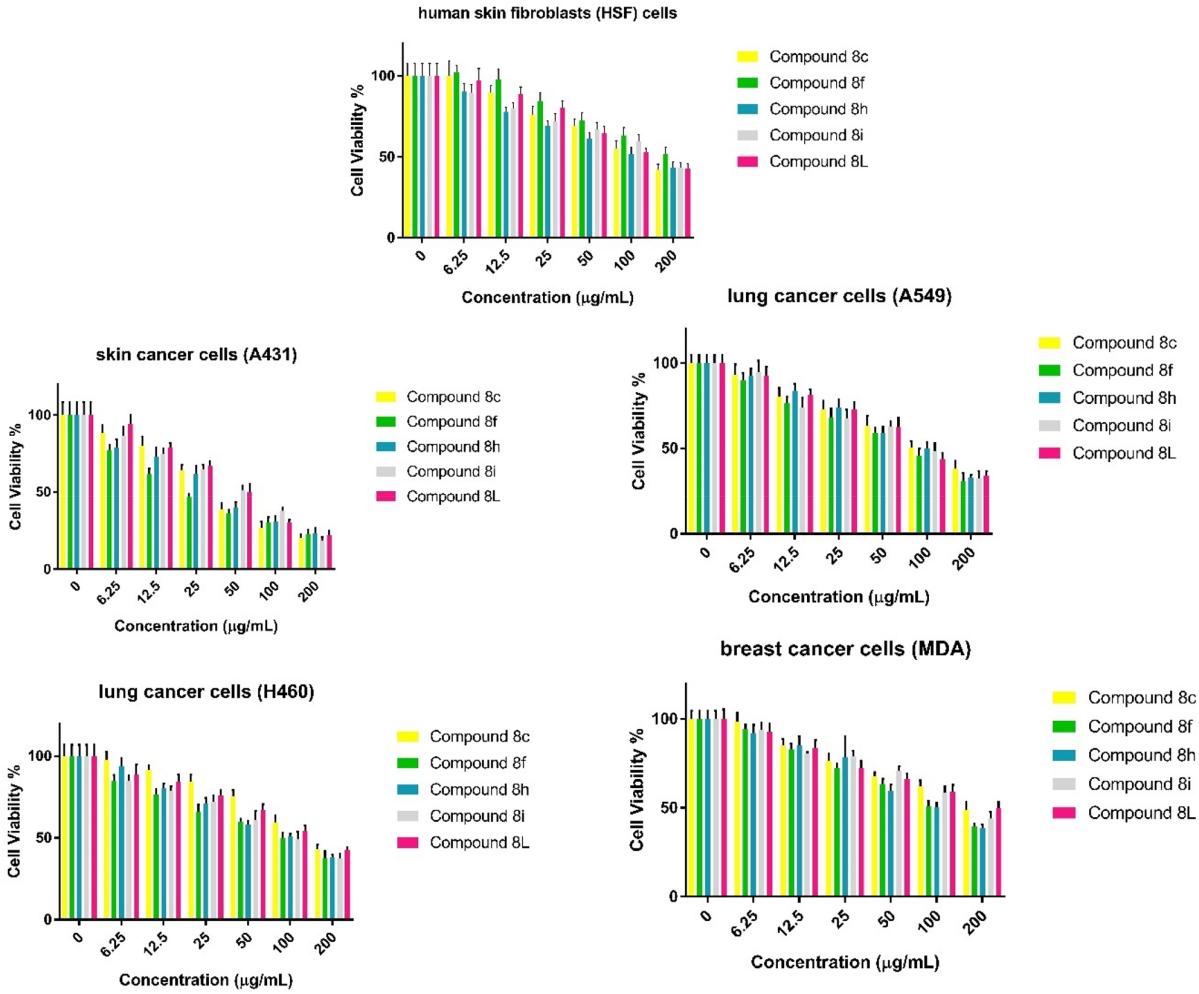
Effect of the prepared derivatives **8c, 8f, 8h, 8i,** and** 8L** on the cell viability of human skin fibroblasts (HSF) cells, lung cancer cells (H460), skin cancer cells (A431), lung cancer cells (A549), and breast cancer cells (MDA-MB-231) using different concentrations (0 to 200 µg/ml) for each compound after treatment for 48 h compared to untreated cells (expressed as triplicate values mean ± SEM).

The effect of compounds **8c, 8f, 8h, 8i,** and** 8L** on cell viability were evaluated using human skin fibroblasts (HSF) cells, lung cancer cells (H460), skin cancer cells (A431), lung cancer cells (A549), and breast cancer cells (MDA-MB-231) using different concentrations (0 to 200 µg/ml) for each compound. The results of our study show that the five compounds tested had varying effects on the viability of the normal cell line and the four cancer cell lines. Compound **8f** was the most effective against lung cancer cells (H460) and skin cancer cells(A431) (Fig. 4).

Compound **8c** was chosen as a representative of halogen electron-withdrawing group compounds, like compounds **8a**, **8b**, **8g**, and **8k**. Specifically, compound **8c** contains fluorine (F), similar to several fluorine-containing antitumor drugs like 5-fluorouracil (5-FU), which have shown successful applications in cancer therapy. Compound **8f** was selected as a representation of increased steric effect resulting from the presence of a biphenyl group. This addition can enhance lipophilicity, potentially leading to improved binding affinity of the drug to hydrophobic regions of target proteins compared to compound **8d**, which contains only one phenyl group. Compounds **8i** and **8h**, both incorporating a methoxy group, were chosen as representatives of electron-donating group compounds, in contrast to compounds **8e** and **8j**, which contain methyl groups. The inclusion of methoxy groups can promote favorable interactions, such as hydrogen bond formation through oxygen, with specific receptors or target proteins. This interaction has the potential to enhance drug potency. Moreover, compound **8h**, with its two methoxy groups, can further increase the possibility of forming interactions, adding to its potential benefits. Lastly, Compound **8L**, chosen for its combined steric effect and oxygen electron donation, is under investigation for its potential impact on cancer cells. Additionally, the piperonal moiety present in anticancer drugs such as Piperonal ciprofloxacin hydrazone has demonstrated the ability to induce growth arrest and apoptosis in human hepatocarcinoma cells. These carefully chosen compounds will help us gain insights into how different substituents impact the drugs' interactions with tumor cells and target proteins in our antitumor drug research.

**Figure 5:**
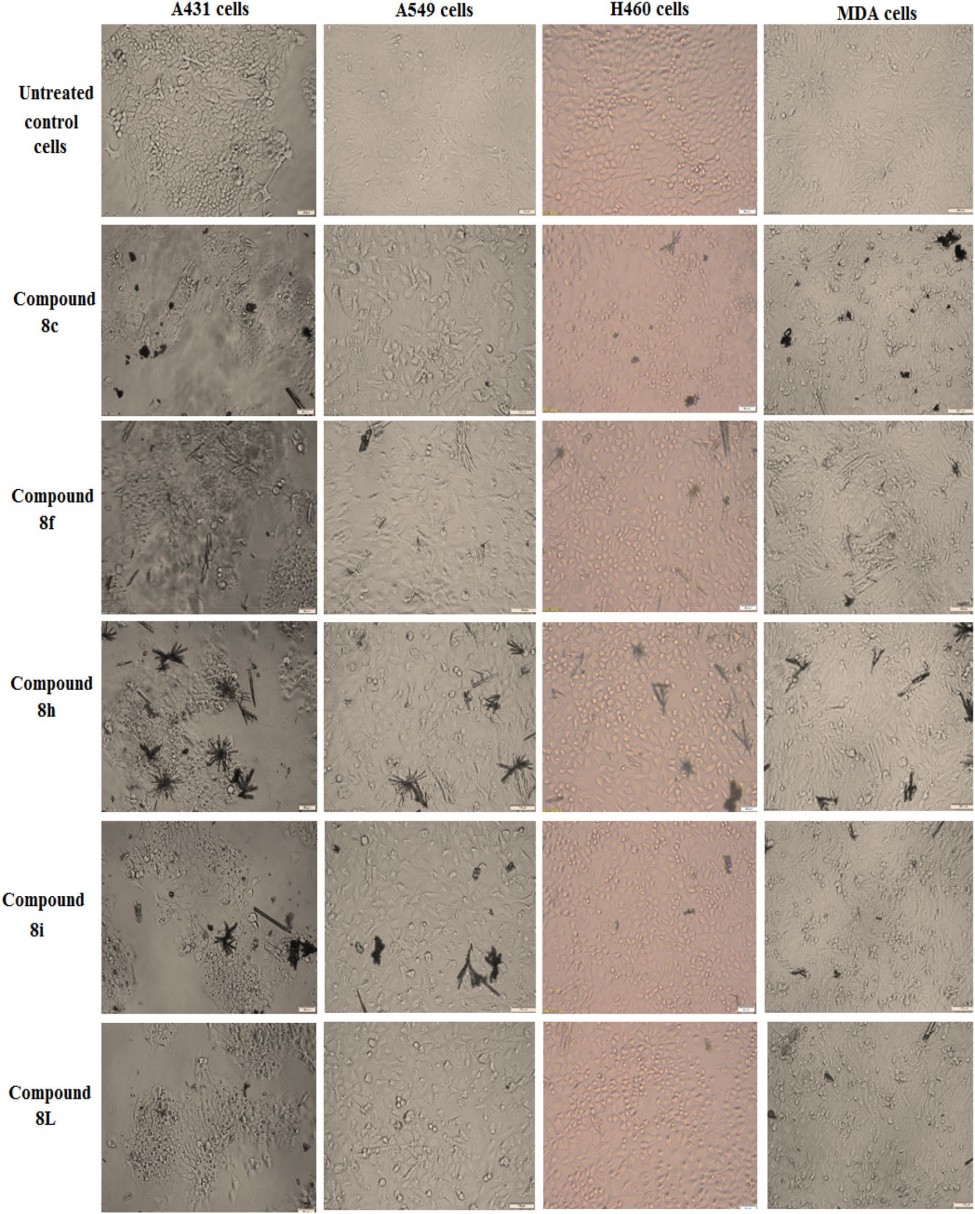
In vitro effect of the synthetized compounds on the morphological modifications of A431 cells, A549 cells, H460 cells and MDA-MB-231 cells as observed under a phase-contrast microscope.

The present study aimed to investigate the in vitro effects of five synthetic compounds (**8c**, **8f**, **8h**, **8i**, and **8L**) on the morphological modifications of four different cancer cell lines (A431, A549, H460, and MDA-MB-231) compared with untreated control cells (Fig. 5). The cells were treated with the compounds at IC_50_ doses for 48 h, and the morphological changes were observed under a phase-contrast microscope. Our results demonstrated that compounds **8f** and **8i** induced significant cell damage and alteration in the morphology of A431 cells. Similarly, compounds **8c** and **8f** also caused noticeable cell damage and alterations in the morphology of H460 cells. In the case of MDA-MB-231 cells, compounds **8f** and **8L** induced significant cell damage and alterations in cell morphology. Moreover, compounds **8f** and **8c** also caused significant cell damage and alterations in the morphology of A549 cells. These observations suggest that compounds **8f** and **8i** may have potential therapeutic applications for the treatment of cancer, as they demonstrated a significant ability to induce cell damage and alter cell morphology in multiple cancer cell lines.

All cells were treated with compounds (**8c**, **8f**, **8h**, **8i** and **8L**) at IC_50_ doses for 48 h as compared to untreated control cells.

**Figure 6:**
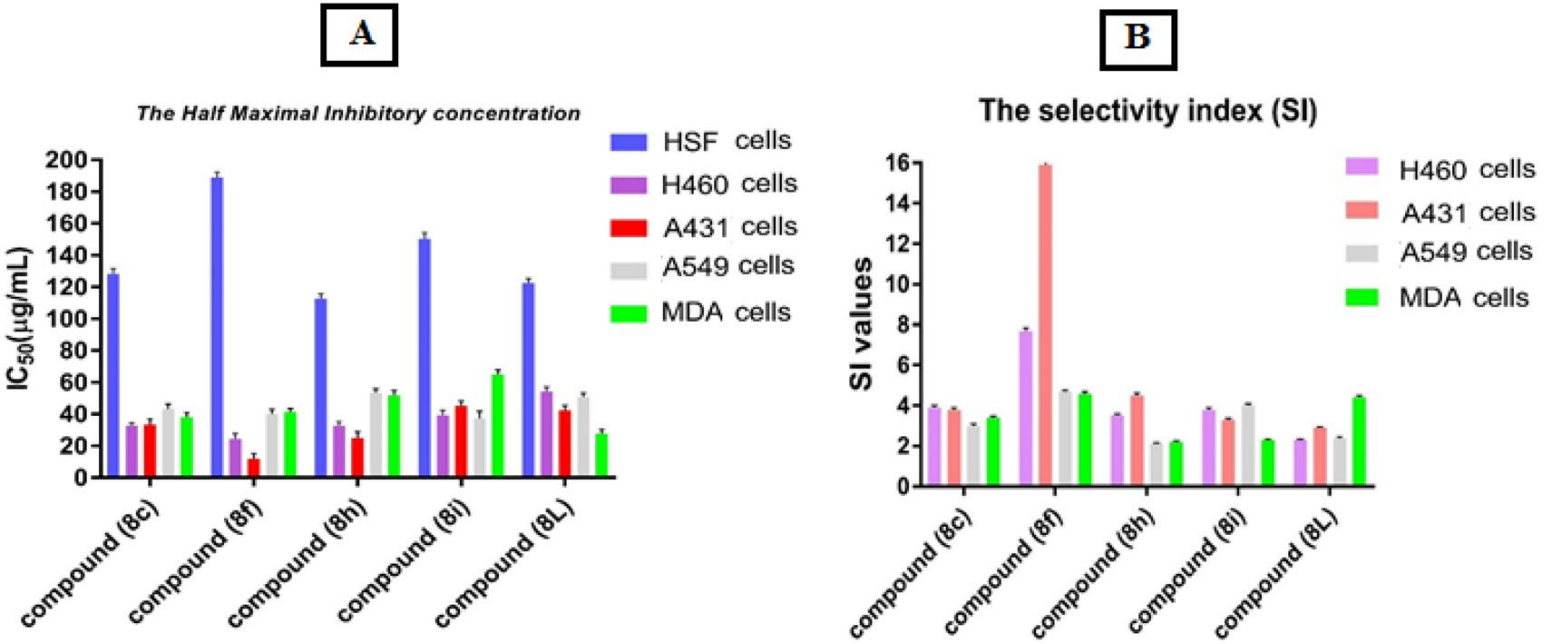
The antitumor activity of the prepared derivatives **8c, 8f, 8h, 8i**, and **8L** against lung cancer cells (H460), skin cancer cells (A431), lung cancer cells (A549), and breast cancer cells (MDA-MB-231) compared with human skin fibroblasts (HSF) cells. **(A)** The half maximal inhibitory concentration (expressed in IC_50_ (μg/mL) values as mean ± SEM). **(B)** The selectivity index (expressed as SI values mean ± SEM).

The data provided in (Fig. 6A) shows the IC_50_ values of five different compounds (**8c, 8f, 8h, 8i,** and** 8L**) on five different types of cells (human skin fibroblasts (HSF) cells, lung cancer cells (H460), skin cancer cells (A431), lung cancer cells (A549), and breast cancer cells (MDA-MB-231). The IC_50_ value represents the concentration of a compound required to inhibit 50% of cell growth in vitro. From the results, it can be seen that the IC_50_ values vary for each compound and cell type. Among the tested compounds, compound **8f** has the lowest IC_50_ value in lung cancer cells (H460) (24.58 ± 3.4) and skin cancer cells (A431) (11.9 ± 3.3), indicating that it has a higher potency in inhibiting the growth of these cell types. Besides, compound **8i** has the lowest IC_50_ value in lung cancer cells (A549) (37.44 ± 4.5) and compound **8L** has low IC_50_ value in breast cancer cells (MDA-MB-231) (27.67 ± 2.7), indicating that these compounds have higher potency in inhibiting the growth of these cell types.

The selectivity index (SI) values indicate the ability of a compound to selectively inhibit cancer cells while sparing normal cells. A higher SI value suggests greater selectivity of the compound towards cancer cells. Based on the SI values presented in (Fig. 6B), compound **8f** shows the highest selectivity towards lung cancer cells (H460) and skin cancer cells (A431), with SI values of 7.7 and 15.9, respectively. Compound **8h** shows moderate selectivity towards lung cancer cells (H460) and skin cancer cells (A431) with SI values of 3.5 and 4.5, respectively. Compound **8i** exhibits moderate selectivity towards lung cancer cells (A549) and compound **8L** towards breast cancer cells (MDA-MB-231) with SI values of 4.0 and 4.4, respectively.

**Figure 7:**
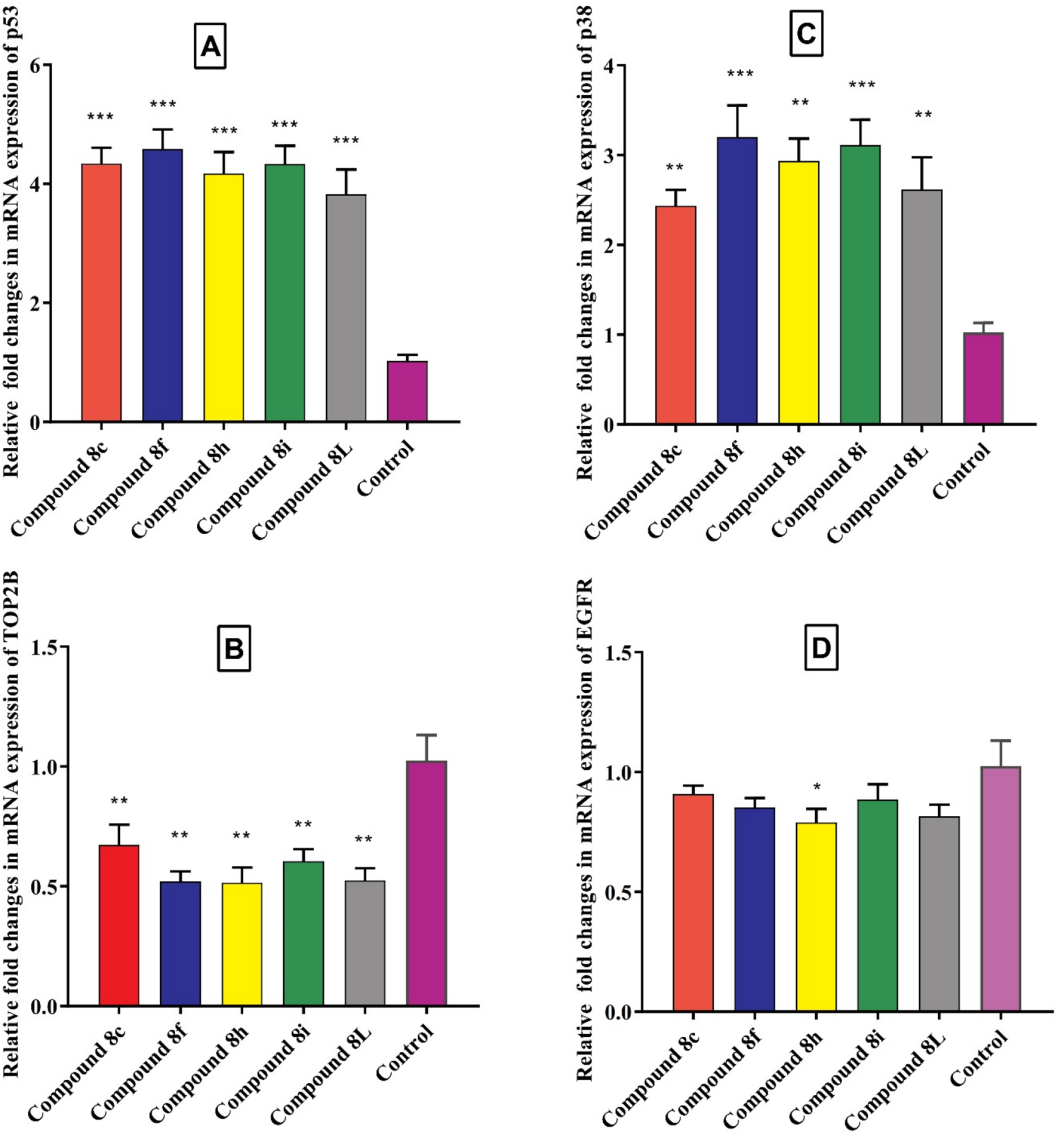
Evaluating the relative fold changes in mRNA expression levels of four genes (p53(A), TOP2B (B), p38 (C), and EGFR(D)) in A431 cells treated with the five selected synthesized compounds compared to control. Each bar represents the mean ± SEM (n = 3) and *p < 0.05, **p < 0.01, ***p < 0.001 and ****p < 0.0001 versus untreated control.

Evaluation of the relative changes in mRNA expression levels of four key genes (p53, TOP2B, p38, and EGFR) in A431 cells treated with the five synthesized compounds **8c**, **8f**, **8h**, **8i**, and **8L** revealed interesting findings (Fig. 7). TOP2B is responsible for DNA replication and transcription, while EGFR plays a crucial role in cell division and survival. The reduced expression of TOP2B may impair the cancer cells' ability to replicate DNA and transcribe genes, and the reduced expression of EGFR may limit their capacity to divide and survive. The relative changes in mRNA expression levels of TOP2B and EGFR were less than one, indicating a decrease in their expression levels in response to the compounds. Compounds **8f** and **8h** showed the lowest relative change in mRNA expression levels of TOP2B and EGFR among the tested compounds. On the other hand, the relative changes in mRNA expression levels of p53 and p38 were more than one, upregulation of p53 and p38 expression in cancer cells may reflect an attempt by the cells to activate their tumor suppressor pathways and prevent uncontrolled cell growth and tumor formation.

The results suggest that the synthesized compounds had varying effects on the mRNA expression levels of the tested genes in A431 cells. The decrease in mRNA expression levels of TOP2B and EGFR suggests a potential role of the compounds in inhibiting the survival and growth of cancer cells, as these genes are known to promote cell survival and proliferation. On the other hand, the increase in mRNA expression levels of p53 and p38 suggests a potential role of the compounds in inducing cell death, as these genes are known to play a crucial role in the regulation of the cell cycle and apoptosis. Compound **8f** showed the lowest relative change in mRNA expression levels of TOP2B and EGFR, and the highest relative change in mRNA expression levels of p53 and p38, suggesting its potential as a strong candidate for further investigation as an anticancer agent.

### Molecular docking

Molecular docking was performed for five compounds (**8c, 8f, 8h, 8i,** and **8L**) against four target proteins Topoisomerase II beta (TOP2B), p38 mitogen-activated protein kinase (MAPK), tumor protein interacted with the mouse double minute 2 homolog (p53), and Epidermal growth factor receptor (EGFR) using Molecular Operating Environment (MOE 2019) software. The binding affinities of each compound to each target protein were calculated and compared with a reference ligand known to bind to the protein with high affinity. The predicted binding poses of each compound were analyzed using the root mean square deviation (RMSD) metric to determine their similarity to the known crystal structures of the protein–ligand complexes (Table 1).

**Table 1:** Binding Energies (BE) (kcal/mol) and RMSD Values of Compounds Targeting TOP2B, p38, p53, and EGFR Compared to Reference Ligands Etoposide, Sorafenib, Chromeno triazolopyrimidine, and Erlotinib, respectively.

Compound	TOP2B		p38		p53		EGFR	
	BE	RMSD (Å)	BE	RMSD (Å)	BE	RMSD (Å)	BE	RMSD (Å)
**8c**	−7.72	1.56	−9.61	1.68	−7.36	1.43	−7.66	1.63
**8f**	−8.53	1.62	−9.90	2.42	−8.28	1.81	−8.00	1.72
**8h**	−8.57	1.73	−10.33	2.11	−7.52	1.45	−8.24	1.71
**8i**	−7.78	1.85	−9.25	1.64	−7.52	2.04	−7.63	1.37
**8L**	−7.69	2.12	−9.88	1.53	−7.43	1.53	−6.92	1.43
Reference ligand	−8.30	1.39	−10.18	1.05	−7.58	0.97	−8.93	0.84

Based on the data (Table 1), The binding energy for most compounds was very close to that of the reference drug. Our findings demonstrate that compound **8f** exhibited high binding affinity with TOP2B, p38, p53, and EGFR, with binding energies of −8.53, −9.90, −8.28, and −8.00 kcal/mol, respectively. The tabular data (Table 2), provides information on the binding amino acids involved in the interaction between each compound and its respective target protein. For each compound, the table lists the binding amino acids for each protein target, along with the type of bond involved (e.g., H-acceptor, pi-cation, etc.).

**Table 2:** Binding amino acids of five compounds against TOP2B, p38, p53, and EGFR proteins.

Compound	TOP2B	p38	p53	EGFR
	Binding amino acids (*Bond type*)	Binding amino Acids (*Bond type*)	Binding amino acids (*Bond type*)	Binding amino acids (*Bond type*)
**8c**	LYS-739 (*H-acceptor*), HIS-775 (*H-acceptor*), GLY-776 (*pi-H*)	GLU-71 (*H-donor*), HIS-148 (*H-acceptor*), ASP-168 (*H-acceptor*), LYS-53 (*pi-cation*)	TYR-100 (*H-acceptor*)	CYS-773 (*H-donor*)
**8f.**	LYS-739 (H-acceptor), HIS-775 (*pi-H*), GLY-776 (*pi-H*), ASN-786 (*pi-H*)	GLU-71 (*H-donor*), HIS-148 (*H-acceptor*), ASP-168 (*H-acceptor*), LYS-53 (*pi-cation*)	TYR-100 (*H-acceptor*), ACE-17 (*pi-H*)	ASP-831 (*H-donor*), CYS-773 (*H-donor*), LYS-721 (*H-acceptor*), LYS-692 (*pi-cation*)
**8h**	LYS-739 (*H-acceptor*), 3.15 LYS-814 (*pi-H*)	GLU-71 (*H-donor*), HIS-148 (*H-acceptor*), ASP-168 (*H-acceptor*), LYS-53 (*pi-cation*)	HIS-96 (*pi-pi*)	LYS-692 (*pi-cation*)
**8i**	LYS-505 (*H-acceptor*), 3.21 ARG-503 (*pi-cation*)	HIS-148 (*H-acceptor*), LYS-53 (*H-acceptor*), LYS-53 (*pi-cation*)	HIS-96 (*H-pi*), GLY-58 (*pi-H*)	CYS-773 (*H-donor*), LYS-692 (*H-acceptor*)
**8L**	LYS-814 (*H-acceptor*), LYS-814 (*pi-H*)	GLU-71 (*H-donor*), HIS-148 (*H-acceptor*), LYS-53 (*pi-cation*)	TYR-67 (*H-pi*)	CYS-773 (*H-donor*)

**Figure 8:**
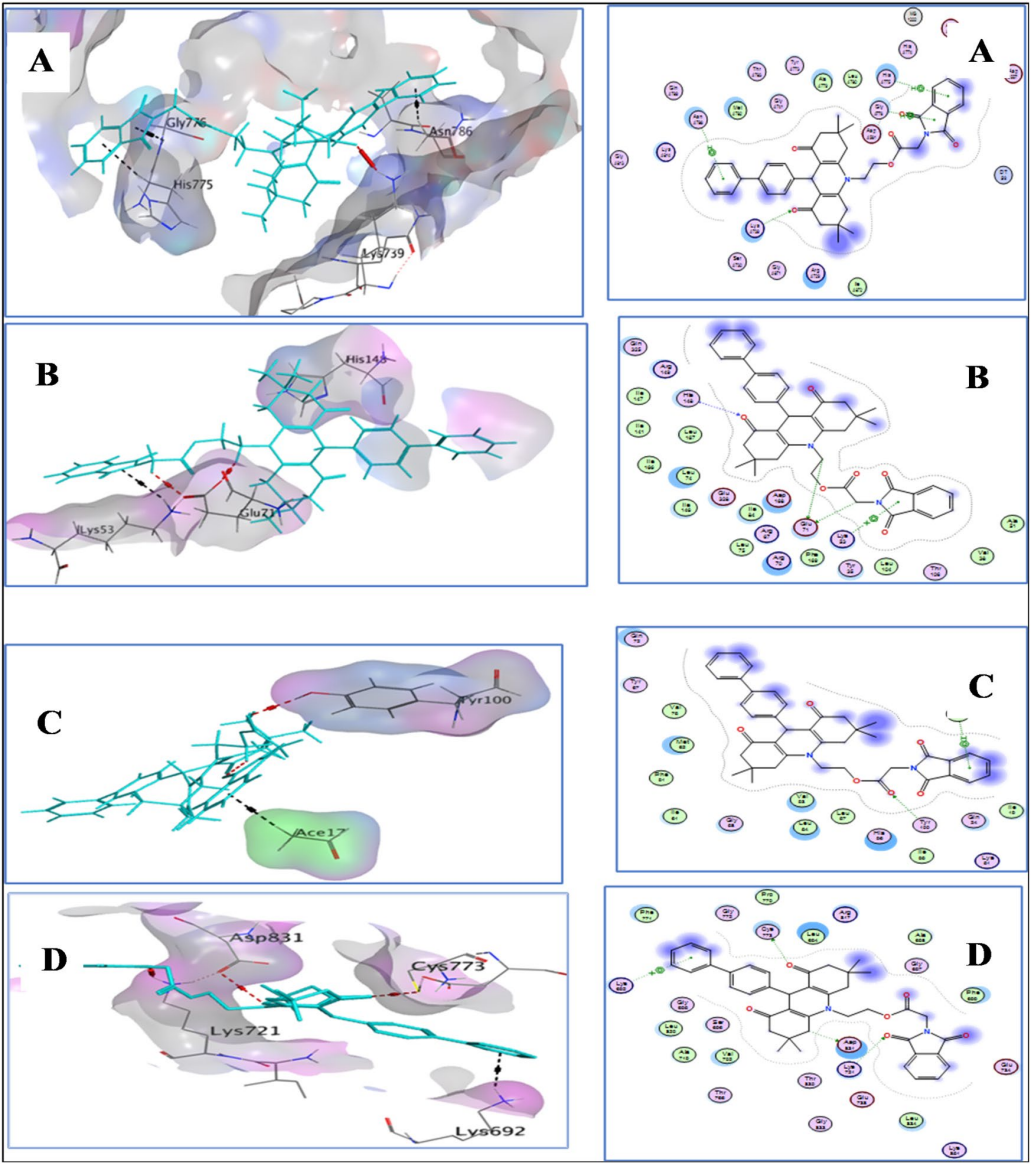
Docking results of compound **8f** with four proteins, namely TOP2B (**A**), p38 (**B**), p53 (**C**), and EGFR (**D**). The left panels of the figure depict the 3D representation of the binding interaction between the compound **8f** (shown in cyan color) and the proteins (highlighted in red color for H-bonding and black color for pi-H bonding). Meanwhile, the right panels provide a 2D representation of the binding interaction for each of the four proteins with compound **8f**.

We further investigated the interaction of compound **8f** with amino acids of the four proteins, namely TOP2B, p38, p53, and EGFR, using molecular docking simulations. Our results showed that compound **8f** formed stable hydrogen bonds, pi-cation, and pi-H bond with several key amino acids of each protein (Fig. 8).

**Figure 9:**
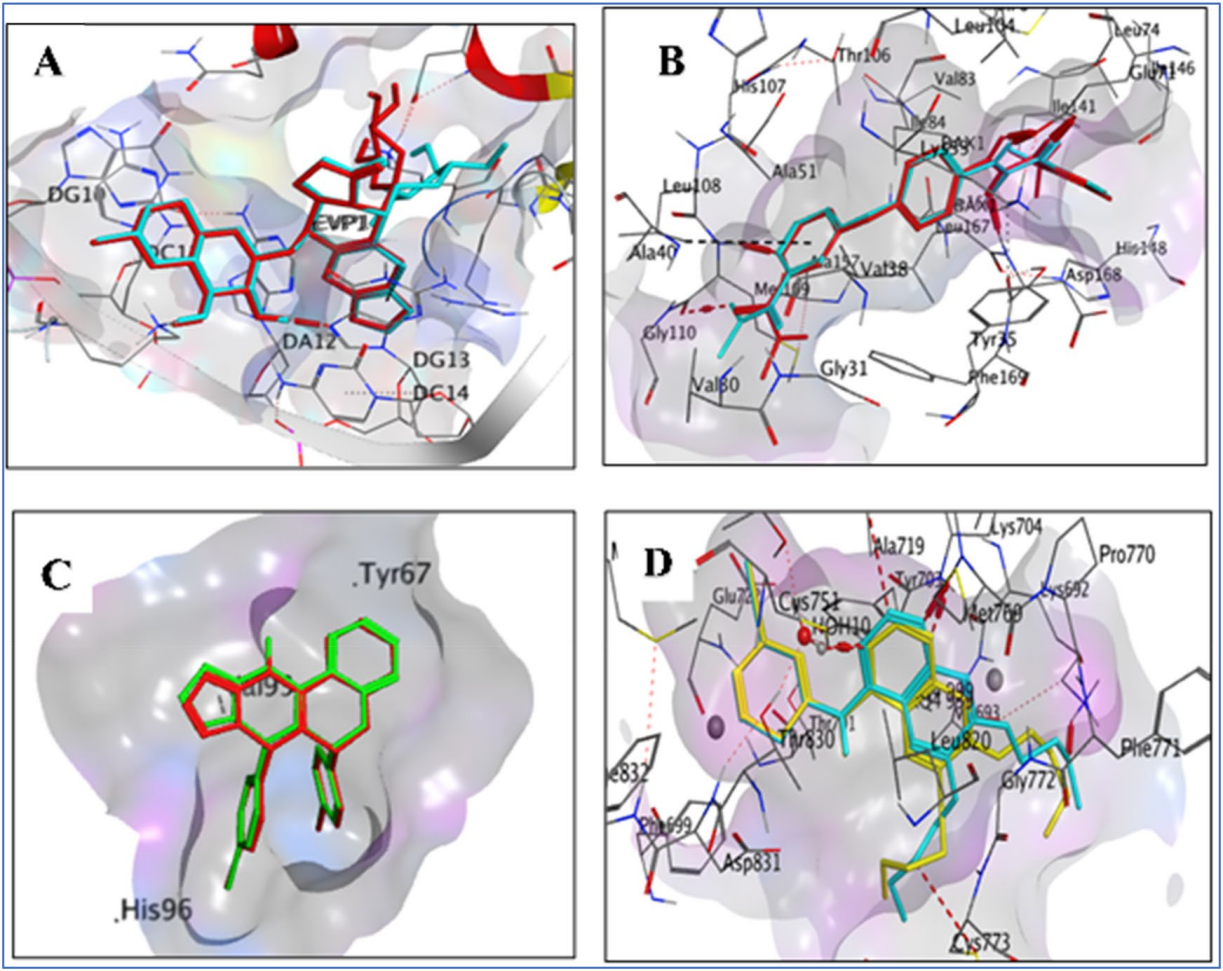
(**A**) This 3D diagram shows the superimposition of the native co-crystallized ligand Etoposide (cyan) and the redocked co-crystallized Etoposide (red) structure at the TOP2B protein target. The RMSD value, which indicates the difference between the two structures, was calculated to be 1.39 Å. (**B**) This 3D diagram displays the superimposition of the native co-crystallized ligand Sorafenib (cyan) and the redocked co-crystallized Sorafenib (red) structure at the p38 protein target. The RMSD value was calculated to be 1.05 Å. (**C**) This 3D diagram depicts the superimposition of the native co-crystallized ligand chromeno triazolopyrimidine (green) and the redocked co-crystallized chromeno triazolopyrimidine (red) structure at the p53 protein target. The RMSD value was calculated to be 0.97 Å. (**D**) This 3D diagram shows the superimposition of the native co-crystallized ligand erlotinib (cyan) and the redocked co-crystallized erlotinib (yellow) structure at the EGFR protein target. The RMSD value was calculated to be 0.84 Å.

The validation of the MOE program was confirmed using co-crystallized ligands with their respective protein targets, wherein the superimposition of the native co-crystallized ligand and the redocked co-crystallized ligand was visualized through 3D diagrams. The RMSD values for the superimpositions were calculated, and the results were presented in the form of graphs (Fig. 9).

### Structure–activity relationship (SAR)

Regarding the IC_50_ values observed on various cancer cell lines and the docking scores from the molecular modeling study, it was evident that compound **8f**, which features a biphenyl substituent, exhibited the most potent activity against skin cancer cell lines (A431) with an IC_50_ of 11.9 ± 3.3 μg/mL. Additionally, the molecular modeling study indicated that compound **8f** showed favorable binding scores on four key genes (p53, TOP2B, p38, and EGFR).

The incorporation of the phenyl group in the compound introduced distinctive characteristics that influenced its lipophilicity, receptor binding affinity, and overall biological activity. These unique properties likely contributed to its enhanced potency against skin cancer cells and its ability to interact effectively with the mentioned target genes. The SAR analysis of phthalimide acridinedione derivatives emphasized the crucial role of specific substituents in regulating the compounds' biological activity.

Notably, the presence of the biphenyl group exerted a profound influence, significantly affecting the compounds' characteristics and overall effectiveness. Compounds featuring electron-withdrawing groups, such as fluoro atom, exhibited moderate biological activity, while those with electron-donating groups, such as methoxy group, demonstrated the lowest biological activity. These findings offer valuable guidance for future compound optimization and drug design, as they pave the way for the discovery of novel and potent therapeutic agents.

## Conclusion

In this study, a series of *N*-substituted acrididine-1,8-dione derivatives were synthesized and characterized. These compounds were designed as hybrids of phthalimide and acridine-1,8-diones, with the aim of developing a dual antitumor profile. Cytotoxicity and tumor selectivity of the synthesized compounds were evaluated against four cancer cell lines and human skin fibroblast cells. Compound **8f** showed promising activity against skin and lung cancers, with favorable IC_50_ values and selectivity index. Additionally, compound **8f** showed the lowest relative change in mRNA expression levels of TOP2B and EGFR, and the highest relative change in mRNA expression levels of p53 and p38, suggesting its potential as a strong candidate for further investigation as an anticancer agent. The results also indicate that compounds **8f** and **8i** may have potential therapeutic applications for the treatment of cancer, as they showed a significant ability to induce cell damage and alter cell morphology in multiple cancer cell lines. Furthermore, molecular docking studies revealed that compound **8f** exhibited high binding affinity with TOP2B, p38, p53, and EGFR, suggesting its potential as a targeted anticancer therapy. Therefore, the *N*-substituted acrididine-1,8-dione derivatives synthesized in this study have the potential to be developed as novel antitumor agents with a dual mechanism of action, and compound **8f** is a promising candidate for further investigation.

## Materials and methods

### Chemistry

Melting points were measured on The Stuart® SMP50 Automatic Digital Melting Point Apparatus. The infrared (IR) spectra were recorded using potassium bromide (KBr) disks on Fourier transform infrared Thermo Electron Nicolet 7600 spectrometer (Thermo Fisher Scientific Inc., Waltham, MA, USA) at the Central Laboratory of Faculty of Science, Ain Shams University. The ^13^C NMR and ^1^H NMR spectra were run at Bruker 400 MHz Avance III Nuclear Magnetic Resonance (NMR) spectrometer using tetramethyl silane (TMS) as internal standard in deuterated dimethyl sulfoxide (DMSO-d_6_) at Faculty of Pharmacy, Mansoura University. The mass spectra were recorded on a Shimadzu Liquid Chromatograph Mass Spectrometer LCMS-2020 at Egypt-Japan University for Science and Technology. The reactions were monitored by the Thin Layer Chromatography (TLC) using Merck Kiesel Gel 60F254 analytical sheets obtained from Fluka. The Antitumor Activity was performed at Protein Research Dep., GEBRI, City of Scientific Research and Technological Applications (SRTA-City), New Borg Al-Arab City 21934, Alexandria, Egypt.

#### Synthesis of 9-aryl-10-(2^′^-hydroxyethyl)-3,3,6,6-tetramethyl-3,4,6,7,9,10 hexahydroacridine-1,8(2H,5H)-diones (4a-L)

In a 50 mL round flask, a mixture of dimedone **1** (2.8 g, 20 mmol), different aromatic aldehydes **2a–L** (10 mmol), 2-aminoethanol **3** (0.72 mL, 12 mmol) was dissolved in *N*,*N*-dimethyl formamide (9 mL) then *p*-toluenesulfonic acid (PTSA) (1.5 gm) or hydrochloric acid (HCl) 37% (1 mL) was added as a catalyst. The mixture was stirred at 110 °C under reflux for 24 h. The progression of the reaction was monitored by TLC. After completion, the reaction mixture was cooled to room temperature then dropped into water (120 mL) while stirring until complete precipitation. The product was filtered off, washed with water, and dried at room temperature. The crude products were recrystallized from toluene to get pure compounds **4a–L**.

##### 9-(4-Bromophenyl)-10-(2^′^-hydroxyethyl)-3,3,6,6-tetramethyl-3,4,6,7,9,10-hexahydro- acridine-1,8(2H,5H)-dione (4a)

Compound **(4a)** was obtained as pale-yellow powder, (3.3 g, 70% yield); **mp.** 229–231 °C. **IR (KBr) *ν***_**max**_** (cm**^**−1**^**)**: 3488 (O–H); 3034 (=C–H, *sp*^2^); 2955, 2869 (C–H, *sp*^3^); 1629 (C=O, conjugated ketone); 1567, 1484 (C=C); 1278 (C–N stretching); 1078 (C–O stretching). ^1^H NMR (400 MHz, DMSO-*d*_6_) *δ* (ppm): 0.87 (s, 6 H, 2 CH_3_), 1.01 (s, 6 H, 2 CH_3_), 2.02 (d, 2 H, CH_2_, *J* = 6.5 Hz), 2.14 (d, 2 H, CH_2_, *J* = 6.4 Hz), 2.44 (d, 2 H, CH_2_, *J* = 6.8 Hz), 2.72 ( d, 2 H, CH_2_, *J* = 6.7 Hz), 3.56 (t, 2 H, CH_2_–N, *J* = 7.1 Hz), 3.85 (t, 2 H, CH_2_–O, *J* = 7.0 Hz), 4.94 (s, 1 H, acridinedione-H_9_), 5.04 (br.s, 1 H, O–H, exchangeable), 7.16 (d, 2 H, Ar–H, *J* = 8.2 Hz), 7.30 (d, 2 H, Ar–H, *J* = 8.1 Hz). ^13^C NMR (100 MHz, DMSO-*d*_6_) *δ* (ppm): 195.63 (2 C=O), 152.40, 146.16, 130.93, 130.36, 118.95 (Ar–C), 113.60, 61.30, 49.93, 46.61, 39.68 (under DMSO-*d*_6_), 32.48, 31.75, 29.29 (2 CH_3_), 27.38 (2 CH_3_). MS (ESI +) *m/z*: Expected for C_25_H_30_BrNO_3_ [M + H]^+^: 474.14, found 474.

##### 9-(4-chlorophenyl)-10-(2-hydroxyethyl)-3,3,6,6-tetramethyl-3,4,6,7,9,10-hexahydroacridine-1,8(2H,5H)-dione (4b)

Compound **(4b)** was obtained as pale-yellow powder, (3.5 g, 81.8% yield); **mp.** 173–175 °C. IR (KBr) *ν*_max_ (cm^−1^): 3335 (br.O–H); 3034 (=C–H, *sp*^2^); 2959, 2886 (C–H, *sp*^3^); 1619 (C=O, conjugated ketone); 1563, 1487 (C=C); 1341 (C–N stretching);1089 (C–O stretching). ^1^H NMR (400 MHz, DMSO-*d*_6_) *δ* (ppm): 0.86 (s, 6 H, 2 CH_3_), 1.02 (s, 6 H, 2 CH_3_), 2.02 (d, 2 H, CH_2_, *J* = 6.5 Hz), 2.15 (d, 2 H, CH_2_, *J* = 6.4 Hz), 2.45 (d, 2 H, CH_2_, *J* = 6.8 Hz), 2.73 (d, 2 H, CH_2_, *J* = 6.9 Hz), 3.56 (t, 2 H, CH_2_–N, *J* = 7.2 Hz), 3.85 (t, 2 H, CH_2_–O, *J* = 7.2 Hz), 4.95 (s, 1 H, acridinedione-H_9_), 5.12 (br.s, 1 H, O–H, exchangeable), 7.18 (d, 2 H, Ar–H, *J* = 8.3 Hz), 7.23 (d, 2 H, Ar–H, *J* = 8.2 Hz). ^13^C NMR (100 MHz, DMSO-*d*_6_) *δ* (ppm): 195.69 (2 C=O), 152.40, 145.76, 130.46, 129.95, 128.06 (Ar–C), 113.56, 61.29, 49.84, 46.54, 39.68 (under DMSO-*d*_6_), 32.52, 31.66, 29.34 (2 CH_3_), 27.33 (2 CH_3_). MS (ESI+) *m/z*: Expected for C_25_H_30_ClNO_3_ [M + H]^+^: 428.19, found 428.

##### 9-(4-fluorophenyl)-10-(2-hydroxyethyl)-3,3,6,6-tetramethyl-3,4,6,7,9,10-hexahydroacridine-1,8(2H,5H)-dione (4c)

Compound **(4c)** was obtained as pale-yellow powder, (3.7 g, 90% yield); **mp.** 215–217 °C. IR (KBr) *ν*_max_ (cm^−1^): 3357 (O–H stretching); 3055, 3034 (=C–H, *sp*^2^); 2963, 2930, 2817 (C–H, *sp*^3^); 1610, 1628 (C=O, conjugated ketone); 1561, 1505, 1485 (C=C); 1334 (C–N stretching); 1087 (C–O stretching). ^1^H NMR (400 MHz, DMSO-*d*_6_) *δ* (ppm): 0.87 (s, 6 H, 2 CH_3_), 1.02 (s, 6 H, 2 CH_3_), 2.02 (d, 2 H, CH_2_, *J* = 6.9 Hz), 2.15 (d, 2 H, CH_2_, *J* = 6.8 Hz), 2.45 (d, 2 H, CH_2_, *J* = 7.3 Hz), 2.76 (d, 2 H, CH_2_, *J* = 7.4 Hz), 3.56 (t, 2 H, CH_2_–N, *J* = 7.5 Hz), 3.85 (t, 2 H, CH_2_–O, *J* = 7.5 Hz), 4.95 (s, 1 H, acridinedione-H_9_), 5.12 (br. s, 1 H, O–H), 6.93 (d, 2 H, Ar–H, *J* = 8.1 Hz), 7.22 (d, 2 H, Ar–H, *J* = 8.2 *Hz*). MS (ESI +) *m/z*: Expected for C_25_H_30_FNO_3_ [M + H]^+^: 412.22, found 412.

##### 10-(2-hydroxyethyl)-3,3,6,6-tetramethyl-9-phenyl-3,4,6,7,9,10-hexahydroacridine-1,8(2H,5H)-dione (4d)

Compound **(4d)** was obtained as pale-yellow powder, (3.1 g, 78.8% yield); **mp.** 246–249 °C. IR (KBr) *ν*_max_ (cm^−1^): 3358 (O–H stretching); 3079, 3047, 3028 (=C–H, *sp*^2^); 2956, 2932, 2869 (C–H, *sp*^3^); 1629, 1612 (C=O, conjugated ketone); 1562, 1491, 1447 (C=C); 1333 (C–N stretching); 1087 (C–O stretching). ^1^H NMR (400 MHz, DMSO-*d*_6_) *δ* (ppm): 0.87 (s, 6 H, 2 CH_3_), 1.02 (s, 6 H, 2 CH_3_), 2.03 (d, 2 H, CH_2_, *J* = 6.2 Hz), 2.15 (d, 2 H, CH_2_, *J* = 6.3 Hz), 2.46 (d, 2 H, CH_2_, *J* = 6.7 Hz), 2.74 (d, 2 H, CH_2_, *J* = 6.6 Hz), 3.58 (t, 2 H, CH_2_–N, *J* = 7.4 Hz), 3.85 (t, 2 H, CH_2_–O, *J* = 7.5 Hz), 4.98 (s, 1 H, acridinedione-H_9_), 5.08 (br. s, 1 H, O–H), 7.01–7.22 (m, 5 H, Ar–H). MS (ESI +) *m/z*: Expected for C_25_H_31_NO_3_ [M + H]^+^: 394.23, found 394.

##### 9-(3,5-dimethylphenyl)-10-(2-hydroxyethyl)-3,3,6,6-tetramethyl-3,4,6,7,9,10-hexahydroacridine-1,8(2H,5H)-dione (4e)

Compound **(4e)** was obtained as pale-yellow powder, (3.5 g, 83% yield); **mp.** 248–250 °C. IR (KBr) *ν*_max_ (cm^−1^): 3373 (O–H stretching); 3036, 2996 (=C–H, *sp*^2^); 2960, 2931, 2865 (C–H, *sp*^3^); 1643, 1620 (C=O, conjugated ketone); 1561, 1500, 1462 (C=C); 1333 (C–N stretching); 1081 (C–O stretching). ^1^H NMR (400 MHz, DMSO-*d*_6_) *δ* (ppm): 0.85 (s, 6 H, 2 CH_3_), 1.02 (s, 6 H, 2 CH_3_), 1.98 (d, 2 H, CH_2_, *J* = 6.4 Hz), 2.10 (s, 3 H, 2 Ar–CH_3_), 2.16 (d, 2 H, CH_2_, *J* = 6.5 Hz), 2.67 (s, 3 H, Ar–CH_3_), 2.47 (d, 2 H, CH_2_, *J* = 6.2 Hz), 2.74 (d, 2 H, CH_2_, *J* = 6.1 Hz), 3.70 (t, 2 H, CH_2_–N, *J* = 6.9 Hz), 3.90 (t, 2 H, CH_2_–O, *J* = 6.8 Hz), 4.93 (s, 1 H, acridinedione-H_9_), 5.08 (br.s, 1 H, O–H), 6.69–6.78 (m, 2 H, Ar–H), 7.00 (s, 1 H, Ar–H). ^13^C NMR (100 MHz, DMSO-*d*_6_) *δ* (ppm): 195.70 (2 C=O), 151.53, 146.62, 134.84, 132.61, 129.46, 129.34, 126.44 (Ar–C), 115.90, 61.20, 50.00, 46.68, 39.68 (under DMSO-*d*_6_), 32.51, 29.46, 28.67, 27.12, 20.99, 19.75. MS (ESI +) *m/z*: Expected for C_27_H_35_NO_3_ [M + H]^+^: 422.26, found 422.

##### 9-([1,1'-biphenyl]-4-yl)-10-(2-hydroxyethyl)-3,3,6,6-tetramethyl-3,4,6,7,9,10-hexahydroacridine-1,8(2H,5H)-dione (4f)

Compound **(4f)** was obtained as pale-yellow powder, (3.85 g, 82% yield); **mp.** 233–235 °C. IR (KBr) *ν*_max_ (cm^−1^): 3427 (O–H stretching); 3051, 3030 (=C–H, *sp*^2^); 2956, 2870 (C–H, *sp*^3^); 1630, 1619 (C=O, conjugated ketone); 1566, 1485, 1466 (C=C); 1331 (C–N stretching); 1076 (C–O stretching). ^1^H NMR (400 MHz, DMSO-*d*_6_) *δ* (ppm): 0.91 (s, 6 H, 2 CH_3_), 1.03 (s, 6 H, 2 CH_3_), 2.06 (d, 2 H, CH_2_, *J* = 6.4 Hz), 2.17 (d, 2 H, CH_2_, *J* = 6.3 Hz), 2.48 (d, 2 H, CH_2_, *J* = 6.9 Hz), 2.77 (d, 2 H, CH_2_, *J* = 6.9 Hz), 3.60 (t, 2 H, CH_2_–N, *J* = 7.4 Hz), 3.87 (t, 2 H, CH_2_–O, *J* = 7.5 Hz), 5.03 (s, 1 H, acridinedione-H_9_), 5.19 (br.s, 1 H, O–H), 7.31–7.33 (m, 3 H, Ar–H, phenyl-H), 7.40–7.47 (m, 4H, Ar–H, phenylene-H), 7.60 (d, 2H, phenyl-H, *J* = 8.2 Hz). ^13^C NMR (100 MHz, DMSO-*d*_6_) *δ* (ppm): 195.70 (2 C=O), 152.24, 146.32, 140.75, 137.88, 129.44, 128.39, 127.62, 126.99, 126.57 (Ar–C), 113.99, 61.45, 50.10, 46.79, 39.68 (under DMSO-d_6_), 32.71, 31.71, 29.45, 27.68. MS (ESI +) *m/z*: Expected for C_31_H_35_NO_3_ [M + H]^+^: 470.26, found 470.

##### 9-(3-bromophenyl)-10-(2-hydroxyethyl)-3,3,6,6-tetramethyl-3,4,6,7,9,10-hexahydroacridine-1,8(2H,5H)-dione (4g)

Compound **(4g)** was obtained as pale-yellow powder, (4.1 g, 86.8% yield); **mp.** 207–209 °C. IR (KBr) *ν*_max_ (cm^−1^): 3363 (O–H stretching); 3073, 3015 (=C–H, *sp*^2^); 2957, 2831, 2869 (C–H, *sp*^3^); 1630, 1613 (C=O, conjugated ketone); 1561, 1494, 1468 (C=C); 1331 (C–N stretching); 1069 (C–O stretching). ^1^H NMR (400 MHz, DMSO-*d*_6_) *δ* (ppm): 0.89 (s, 6 H, 2 CH_3_), 1.03 (s, 6 H, 2 CH_3_), 2.05 (d, 2 H, CH_2_, *J* = 6.7 Hz), 2.2 (d, 2 H, CH_2_, *J* = 6.6 Hz), 2.46 (d, 2 H, CH_2_, *J* = 6.2 Hz), 2.76 (d, 2 H, CH_2_, *J* = 6.3 Hz), 3.57 (t, 2 H, CH_2_–N, *J* = 7.2 Hz), 3.86 (t, 2 H, CH_2_–O, *J* = 7.1 Hz), 4.95 (s, 1 H, acridinedione-H_9_), 5.19 (br. s, 1 H, O–H), 7.10–7.14 (m, 1 H, Ar–H), 7.21–7.25 (m, 2 H, Ar–H), 7.38 (s, 1 H, Ar–H). ^13^C NMR (100 MHz, DMSO-*d*_6_) *δ* (ppm): 195.65 (2 C=O), 152.53, 149.49, 131.18, 130.65, 128.97, 127.08, 121.52 (Ar–C), 113.50, 61.34, 49.96, 46.77, 39.68 (under DMSO-d6), 32.67, 32.28, 29.44, 27.48. MS (ESI +) *m/z*: Expected for C_25_H_30_BrNO_3_ [M + H]^+^: 472.14, found 472.

##### 9-(3,4-dimethoxyphenyl)-10-(2-hydroxyethyl)-3,3,6,6-tetramethyl-3,4,6,7,9,10-hexahydroacridine-1,8(2H,5H)-dione (4h)

Compound **(4h)** was obtained as pale-yellow powder, (3.9 g, 86% yield); **mp.** 206–208 °C. IR (KBr) *ν*_max_ (cm^−1^): 3311 (O–H); 3075 (=C–H, sp2); 2959, 2867 (C–H, sp3); 1644, 1612 (C=O, conjugated ketone); 1563, 1513, 1460 (C=C); 1341 (C–N stretching); 1079 (C–O stretching)_._^1^H NMR (400 MHz, DMSO-*d*_6_) *δ* (ppm): 0.86 (s, 6 H, 2 CH_3_), 1.01 (s, 6 H, 2 CH_3_), 2.01 (d, 2 H, CH_2_, *J* = 6.4 Hz), 2.15 (d, 2 H, CH_2_, *J* = 6.3 Hz), 2.44 (d, 2 H, CH_2_, *J* = 6.9 Hz), 2.71 (d, 2 H, CH_2_, *J* = 6.8 Hz), 3.55 (t, 2 H, CH_2_–N, *J* = 7.5 Hz), 3.63 (s, 3 H, OCH_3_), 3.64 (s, 3 H, OCH_3_), 3.88 (t, 2 H, CH_2_–O, *J* = 7.5 Hz), 4.90 (s, 1 H, acridinedione-H_9_), 5.16 (br.s, 1 H, O–H), 6.70–6.75 (m, 3H, Ar–H). ^13^C NMR (100 MHz, DMSO-*d*_6_) *δ* (ppm): 196.25 (2 C=O), 152.12, 148.45 (C–OCH_3_), 147.03 (C-OCH_3_), 139.43, 120.20, 111.50, 111.34 (Ar–C), 113.83, 61.36, 55.72, 55.53, 49.84, 46.77, 39.68 (under DMSO-d_6_), 32.39, 31.26, 29.61, 26.94. MS (ESI +) *m/z*: Expected for C_27_H_35_NO_5_ [M + H]^+^: 454.25, found 454.

##### 10-(2-hydroxyethyl)-9-(4-methoxyphenyl)-3,3,6,6-tetramethyl-3,4,6,7,9,10-hexahydroacridine-1,8(2H,5H)-dione (4i)

Compound **(4i)** was obtained as pale-yellow powder, (3.7 g, 87.4% yield); **mp.** 198–200 °C. IR (KBr) *ν*_max_ (cm^−1^): 3472 (O–H); 3070, 3011 (=C–H, sp2); 2957, 2869, 2843 (C–H, sp3); 1615 (C=O, conjugated ketone); 1561, 1510, 1465 (C=C); 1331 (C–N stretching); 1070 (C–O stretching). ^1^H NMR (400 MHz, DMSO-*d*_6_) *δ* (ppm): 0.87 (s, 6 H, 2 CH_3_), 1.02 (s, 6 H, 2 CH_3_), 2.01 (d, 2 H, CH_2_, *J* = 6.2 Hz), 2.14 (d, 2 H, CH_2_, *J* = 6.1 Hz), 2.44 (d, 2 H, CH_2_, *J* = 6.7 Hz), 2.71 (d, 2 H, CH_2_, *J* = 6.7 Hz), 3.55 (t, 2 H, CH_2_–N, *J* = 7.6 Hz), 3.66 (s, 3 H, OCH_3_), 3.83 (t, 2 H, CH_2_–O, *J* = 7.5 Hz), 4.90 (s, 1 H, acridinedione-H_9_), 5.08 (br. s, 1 H, O–H), 6.69 (d, 2H, Ar–H, *J* = 8.4 Hz), 7.12 (d, 2H, Ar–H, *J* = 8.5 Hz). ^13^C NMR (100 MHz, DMSO-*d*_6_) *δ* (ppm): 195.69 (2 C=O), 157.53 (C-OCH_3_), 151.82, 139.20, 129.04, 114.19 (Ar–C), 113.40, 61.30, 55.25, 49.94, 46.54, 39.68 (under DMSO-*d*_6_), 32.50, 30.98, 29.43 (2 CH_3_), 27.34 (2 CH_3_). MS (ESI +) *m/z*: Expected for C_26_H_33_NO_4_ [M + H]^+^: 424.24, found 424.

##### 10-(2-hydroxyethyl)-3,3,6,6-tetramethyl-9-(p-tolyl)-3,4,6,7,9,10-hexahydroacridine-1,8(2H,5H)-dione (4j)

Compound **(4j)** was obtained as pale-yellow powder, (3.2 g, 78.6% yield); **mp.** 218–220  °C. IR (KBr) *ν*_max_ (cm^−1^): 3380 (O–H); 3020 (=C–H, *sp*^2^); 2957, 2875 (C–H, *sp*^3^); 1623 (C=O, conjugated. ketone); 1564, 1511, 1465 (C=C); 1334 (C–N stretching);1082 (C–O stretching).^1^H NMR (400 MHz, DMSO-*d*_6_) *δ* (ppm): 0.87 (s, 6 H, 2 CH_3_), 1.02 (s, 6 H, 2 CH_3_), 2.01 (d, 2 H, CH_2_, *J* = 6.1 Hz), 2.14 (d, 2 H, CH_2_, *J* = 6.1 Hz), 2.18 (s, 3H, Ar–CH_3_), 2.44 (d, 2 H, CH_2_, *J* = 6.6 Hz), 2.71 (d, 2 H, CH_2_, *J* = 6.5 Hz), 3.57 (t, 2 H, CH_2_–N, *J* = 7.2 Hz), 3.83 (t, 2 H, CH_2_–O, *J* = 7.2 Hz), 4.92 (s, 1 H, acridinedione-H_9_), 5.07 (br.s, 1 H, O–H), 6.93 (d, 2 H, Ar–H, *J* = 8.0 Hz), 7.08 (d, 2 H, Ar–H, *J* = 8.0 Hz). ^13^C NMR (100 MHz, DMSO-*d*_6_) *δ* (ppm): 195.68 (2 C=O), 151.93, 143.92, 134.74, 128.70, 127.97 (Ar–C), 114.03, 61.27, 49.92, 46.55, 39.68 (under DMSO-d6), 32.49, 31.41, 29.43 (2 CH_3_), 27.29 (2 CH_3_), 21.03 (CH_3_). MS (ESI+) *m/z*: Expected for C_26_H_33_NO_3_ [M + H]^+^: 408.25, found 408.

##### 9-(3-chlorophenyl)-10-(2-hydroxyethyl)-3,3,6,6-tetramethyl-3,4,6,7,9,10-hexahydroacridine-1,8(2H,5H)-dione (4k)

Compound **(4k)** was obtained as pale-yellow powder, (3.8 g, 88.9% yield); **mp.** 210–212  °C. IR (KBr) *ν*_max_ (cm^−1^): 3361 (O–H stretching); 3077, 3015 (=C–H, *sp*^2^); 2958, 2931, 2887 (C–H, *sp*^3^); 1629 (C=O, conjugated ketone); 1561, 1494, 1447 (C=C); 1371 (C–N stretching); 1087 (C–O stretching). ^1^H NMR (400 MHz, DMSO-*d*_6_) *δ* (ppm): 0.89 (s, 6H, 2 CH_3_), 1.03 (s, 6H, 2 CH_3_), 2.06 (d, 2H, CH_2_, *J* = 6.4 Hz), 2.16 (d, 2H, CH_2_, *J* = 6.4 Hz), 2.47 (d, 2H, CH_2_, *J* = 6.9 Hz), 2.76 (d, 2H, CH_2_, *J* = 6.8 Hz), 3.57 (t, 2H, CH_2_–N, *J* = 7.2 Hz), 3.86 (t, 2H, CH_2_–O, *J* = 7.1 Hz), 4.97 (s, 1H, acridinedione-H_9_), 5.19 (br. s, 1H, O–H), 7.09–7.12 (m, 1H, Ar–H), 7.18 (d, 2H, Ar–H, *J* = 8.2 Hz), 7.24 (s, 1H, Ar–H). MS (ESI +) *m/z*: Expected for C_25_H_30_ClNO_3_ [M + H]^+^: 428.19, found 428.

##### 9-(benzo[d][1,3] dioxol-5-yl)-10-(2-hydroxyethyl)-3,3,6,6-tetramethyl-3,4,6,7,9,10-hexahydroacridine-1,8(2H,5H)-dione (4L)

Compound **(4L)** was obtained as pale-yellow powder, (4 g, 91.5% yield); mp. 201–203  °C. IR (KBr) *ν*_max_ (cm^−1^): 3364 (O–H stretching); 3015 (=C–H, *sp*^2^); 2949, 2932, 2869 (C–H, *sp*^3^); 1618 (C=O, conjugated ketone); 1561, 1489, 1441 (C=C); 1335 (C–N stretching); 1085 (C–O stretching). ^1^H NMR (400 MHz, DMSO-*d*_6_) *δ* (ppm): 0.90 (s, 6H, 2 CH_3_), 1.02 (s, 6H, 2 CH_3_), 2.04 (d, 2H, CH_2_, *J* = 6.5 Hz), 2.14 (d, 2H, CH_2_, *J* = 6.4 Hz), 2.44 (d, 2H, CH_2_, *J* = 6.8 Hz), 2.74 (d, 2H, CH_2_, *J* = 6.8 Hz), 3.56 (t, 2H, CH_2_–N, *J* = 7.3 Hz), 3.83 (t, 2H, CH_2_–O, *J* = 7.2 Hz), 4.90 (s, 1H, acridinedione-H_9_), 5.06 (br. s, 1H, O–H), 5.89 (s, 2H, CH_2_, dioxole-H), 6.65–6.74 (m, 3 H, Ar–H). ^13^C NMR (100 MHz, DMSO-*d*_6_) *δ* (ppm): 195.70 (2 C=O), 151.99, 147.36, 145.47, 141.22, 121.14, 108.83, 108.03 (Ar–C), 114.14, 100.96, 61.29, 50.09, 46.77, 39.68 (under DMSO-d6), 32.67, 31.74, 29.21 (2 CH_3_), 27.16 (2 CH_3_). MS (ESI +) *m/z*: Expected for C_26_H_31_NO_5_ [M + H]^+^: 438.22, found 438.

#### Synthesis of 2-(3,3,6,6-tetramethyl-1,8-dioxo-9-(aryl)-2,3,4,5,6,7,8,9-octahydroacridin-10(1H)-yl) ethyl 2-chloroacetate (6a-L)

In a 20 mL round bottomed flask, compounds** (4a–L**) (5.0 mmol) were dissolved in methylene dichloride (MDC) (5 mL) in the presence of 1,8-diazabicyclo [5.4.0]undec-7-ene (DBU) (1.05 mL, 7.0 mmol) as a catalyst, then chloroacetyl chloride **5** (0.55 mL, 7.0 mmol) was added dropwise. The reaction mixture was stirred at room temperature for 2 h. The reaction was monitored by TLC. After completion, methylene dichloride was distilled out at under reduced pressure and DMF (3 mL) was added to the residue then the mixture was dropped into water (100 mL) with stirring untill complete precipitation. The product was filtered off, washed with water, and dried. The products were recrystallization from methanol to get pure compounds **6a–L**.

##### 2-(9-(4-bromophenyl)-3,3,6,6-tetramethyl-1,8-dioxo-2,3,4,5,6,7,8,9-octahydroacridin-10(1H)-yl) ethyl 2-chloroacetate (6a)

Compound **(6a)** was obtained as white powder, (2.3 g, 83.8% yield); **mp.** 199–201  °C. IR (KBr) *ν*_max_ (cm^−1^): 3015 (=C–H, *sp*^2^); 2955, 2870 (C–H, *sp*^3^); 1767 (C=O, ester); 1626 (C=O, conjugated ketone, acridinedione); 1567, 1484 (C=C); 1335 (C–N stretching); 1091 (C–O stretching). ^1^H NMR (400 MHz, DMSO-*d*_6_) *δ* (ppm): 0.88 (s, 6 H, 2 CH_3_), 1.03 (s, 6 H, 2 CH_3_), 2.11–2.21 (m, 4 H, 2 CH_2_), 2.47 (d, 2 H, CH_2_, *J* = 6.7 Hz), 2.75 (d, 2 H, CH_2_, *J* = 6.8 Hz), 4.13–4.17 (t, 2 H, CH_2_-N, *J* = 7.1 Hz), 4..22 (s, 2 H, CH_2_-Cl), 4.25 (t, 2 H, CH_2_-O, *J* = 7.2 Hz), 5.00 (s, 1 H, acridinedione-H_9_), 7.04 (d, 2 H, Ar–H, *J* = 8.3 Hz), 7.33 (d, 2 H, Ar–H, *J* = 8.3 Hz). ^13^C NMR (100 MHz, DMSO-*d*_6_) *δ* (ppm): 195.70 (2 C=O, acridinedione), 167.59 (C=O, ester), 152.14, 145.69, 131.08, 129.78, 119.16 (Ar–C), 113.85, 65.92, 49.93, 42.84, 41.39, 39.68 (under DMSO-*d*_6_), 32.57, 31.19, 29.02, 27.77. MS (ESI +) *m/z*: Expected for C_27_H_31_BrClNO_4_ [M + H]^+^: 550.11, found 550.

##### 2-(9-(4-chlorophenyl)-3,3,6,6-tetramethyl-1,8-dioxo-2,3,4,5,6,7,8,9-octahydroacridin-10(1H)-yl) ethyl 2-chloroacetate (6b)

Compound **(6b)** was obtained as white powder, (2.1 g, 83.3% yield); **mp**. 215–217 °C. IR (KBr) *ν*_max_ (cm^−1^): 3015(=C–H, *sp*^2^); 2956, 2900, 2870 (C–H, *sp*^3^); 1766 (C=O, ester); 1628 (C=O, conjugated ketone, acridinedione); 1566, 1486, 1450 (C=C); 1336 (C–N stretching); 1091 (C–O stretching). ^1^H NMR (400 MHz, DMSO-*d*_6_) *δ* (ppm): 0.92 (s, 6 H, 2 CH_3_), 1.03 (s, 6 H, 2 CH_3_), 2.11 (d, 2 H, CH_2_, *J* = 6.3 Hz), 2.21 (d, 2 H, CH_2_, *J* = 6.2 Hz), 2.47 (d, 2 H, CH_2_, *J* = 6.8 Hz), 2.75 (d, 2 H, CH_2_, *J* = 6.8 Hz), 4.13 (t, 2 H, CH_2_–N, *J* = 7.2 Hz), 4.22 (s, 2 H, CH_2_–Cl), 4.25 (t, 2 H, CH_2_–O, *J* = 7.2 Hz), 5.02 (s, 1 H, acridinedione-H_9_), 7.09 (d, 2 H, Ar–H, *J* = 8.5 Hz), 7.19 (d, 2 H, Ar–H, *J* = 8.5 Hz). MS (ESI +) *m/z*: Expected for C_27_H_31_Cl_2_NO_4_ [M + H]^+^: 504.16, found 504.

##### 2-(9-(4-fluorophenyl)-3,3,6,6-tetramethyl-1,8-dioxo-2,3,4,5,6,7,8,9-octahydroacridin-10(1H)-yl) ethyl 2-chloroacetate (6c)

Compound **(6c)** was obtained as white powder, (2.2 g, 90.1% yield); **mp.** 205–207 °C. IR (KBr) *ν*_max_ (cm^−1^): 3066, 3045 (=C–H, *sp*^2^); 2956, 2872 (C–H, *sp*^3^); 1758 (C=O, ester); 1635 (C=O, conjugated ketone); 1574, 1535, 1504, 1467 (C=C); 1335 (C–N stretching); 1092 (C–O stretching). ^1^H NMR (400 MHz, DMSO-*d*_6_) *δ* (ppm): 0.93 (s, 6 H, 2 CH_3_), 1.03 (s, 6 H, 2 CH_3_), 2.11 (d, 2 H, CH_2_, *J* = 6.1 Hz), 2.16 (d, 2 H, CH_2_, *J* = 6.2 Hz), 2.52 (d, 2 H, CH_2_, *J* = 6.5 Hz), 2.76 (d, 2 H, CH_2_, *J* = 6.5 Hz), 3.36 (t, 2 H, CH_2_–N, *J* = 7.3 Hz), 4.13 (s, 2 H, CH_2_-Cl), 4.21 (t, 2 H, CH_2_–O, *J* = 7.2 Hz), 5.03 (s, 1 H, acridinedione-H_9_), 6.95 (d, 2 H, Ar–H, *J* = 8.2 Hz), 7.10 (d, 2 H, Ar–H, *J* = 8.2 Hz). MS (ESI +) *m/z*: Expected for C_27_H_31_ClFNO_4_ [M + H]^+^: 488.19: 488.19, found 488.

##### 2-(3,3,6,6-tetramethyl-1,8-dioxo-9-phenyl-2,3,4,5,6,7,8,9-octahydroacridin-10(1H)-yl) ethyl 2-chloroacetate (6d)

Compound **(6d)** was obtained as white powder, (2 g, 85.1% yield); **mp.** 184–186 °C. IR (KBr) *ν*_max_ (cm^−1^): 3056, 3026 (=C–H, *sp*^2^); 2958, 2870 (C–H, *sp*^3^); 1756 (C=O, ester); 1631 (C=O, conjugated ketone, acridinedione); 1571, 1488, 1466 (C=C); 1337 (C–N stretching); 1090 (C–O stretching). ^1^H NMR (400 MHz, DMSO-*d*_6_) *δ* (ppm): 0.93 (s, 6 H, 2 CH_3_), 1.03 (s, 6 H, 2 CH_3_), 2.11 (d, 2 H, CH_2_, *J* = 6.2 Hz), 2.15 (d, 2 H, CH_2_, *J* = 6.3 Hz), 2.51 (d, 2 H, CH_2_, *J* = 6.9 Hz), 2.76 (d, 2 H, CH_2_, *J* = 6.9 Hz), 4.11 (t, 2 H, CH_2_–N, *J* = 7.3 Hz), 4.13 (s, 2 H, CH_2_–Cl), 4.21 (t, 2 H, CH_2_–O, *J* = 7.2 Hz), 5.03 (s, 1 H, acridinedione-H_9_), 7.03–7.15 (m, 5 H, Ar–H). MS (ESI +) *m/z*: Expected for C_27_H_32_ClNO_4_ [M + H]^+^: 470.20, found 470.

##### 2-(9-(3,5-dimethylphenyl)-3,3,6,6-tetramethyl-1,8-dioxo-2,3,4,5,6,7,8,9-octahydroacridin-10(1H)-yl) ethyl 2-chloroacetate (6e)

Compound **(6e)** was obtained as white powder, (2.3 g, 92.3% yield); **mp.** 158–160 °C.IR (KBr) *ν*_max_ (cm^−1^): 3044, 2993 (=C–H, *sp*^2^); 2958, 2872 (C–H, *sp*^3^); 1748 (C=O, ester); 1629 (C=O, conjugated ketone); 1569, 1499, 1470 (C=C); 1338 (C–N stretching); 1095 (C–O stretching). ^1^H NMR (400 MHz, DMSO-*d*_6_) *δ* (ppm): 0.87 (s, 6 H, 2 CH_3_), 1.03 (s, 6 H, 2 CH_3_), 2.00 (d, 2 H, CH_2_, *J* = 6.0 Hz), 2.04 (s, 3 H, CH_3_, Ar–CH_3_), 2.12 (d, 2 H, CH_2_, *J* = 6.0 Hz), 2.51 (d, 2 H, CH_2_, *J* = 6.7 Hz), 2.67 (s, 3 H, CH_3_, Ar–CH_3_), 2.72 (d, 2 H, CH_2_, *J* = 6.6 Hz), 4.16 (t, 2 H, CH_2_-N, *J* = 7.0 Hz), 4.38 (t, 2 H, CH_2_–O, *J* = 7.1 Hz), 4.41 (s, 2 H, CH_2_-Cl), 4.93 (s, 1 H, acridinedione-H_9_), 6.70–6.81 (m, 3 H, Ar–H). MS (ESI +) *m/z*: Expected for C_29_H_36_ClNO_4_ [M + H]^+^: 498.23, found 498.

##### 2-(9-([1,1'-biphenyl]-4-yl)-3,3,6,6-tetramethyl-1,8-dioxo-2,3,4,5,6,7,8,9-octahydroacridin-10(1H)-yl) ethyl 2-chloroacetate (6f)

Compound **(6f)** was obtained as white powder, (2.4 g, 87.9% yield); **mp.** 206–208  °C. IR (KBr) *ν*_max_ (cm^−1^): 3027 (=C–H, *sp*^2^); 2957, 2871 (C–H, *sp*^3^); 1764 (C=O, ester); 1636 (C=O, conjugated ketone, acridinedione); 1573, 1517, 1486 (C=C); 1332 (C–N stretching); 1090 (C–O stretching). ^1^H NMR (400 MHz, DMSO-*d*_6_) *δ* (ppm): 0.96 (s, 6 H, 2 CH_3_), 1.04 (s, 6 H, 2 CH_3_), 2.13 (d, 2 H, CH_2_, *J* = 6.0 Hz), 2.18 (d, 2 H, CH_2_, *J* = 6.0 Hz), 2.51 (d, 2 H, CH_2_, *J* = 6.8 Hz), 2.78 (d, 2 H, CH_2_, *J* = 6.9 Hz), 4.15 (t, 2 H, CH_2_-N, *J* = 7.4 Hz), 4.18 (s, 2 H, CH_2_-Cl), 4.25 (t, 2 H, CH_2_-O, *J* = 7.4 Hz), 5.08 (s, 1 H, acridinedione-H_9_), 7.17 (d, 2 H, Ar–H, *J* = 8.1 Hz), 7.31 (t, 1 H, Ar–H, *J* = 8.4 Hz), 7.42 (t, 4 H, Ar–H, *J* = 8.4 Hz), 7.58 (d, 2 H, Ar–H, *J* = 8.1 Hz). MS (ESI +) *m/z*: Expected for C_33_H_36_ClNO_4_ [M + H]^+^: 546.23, found 546.

##### 2-(9-(3-bromophenyl)-3,3,6,6-tetramethyl-1,8-dioxo-2,3,4,5,6,7,8,9-octahydroacridin-10(1H)-yl) ethyl 2-chloroacetate (6g)

Compound **(6g)** was obtained as white powder, (2.1 g, 76.5% yield); **mp.** 164–166  °C. IR (KBr) *ν*_max_ (cm^−1^):3030 (=C–H, *sp*^2^); 2957, 2872 (C–H, *sp*^3^); 1765 (C=O, ester); 1628 (C=O, conjugated ketone, acridinedione); 1571, 1470, 1454 (C=C); 1337 (C–N stretching); 1083 (C–O stretching). ^1^H NMR (400 MHz, DMSO-*d*_6_) *δ* (ppm): 0.93 (s, 6 H, 2 CH_3_), 1.03 (s, 6 H, 2 CH_3_), 2.11 (d, 2 H, CH_2_, *J* = 6.2 Hz), 2.18 (d, 2 H, CH_2_, *J* = 6.2 Hz), 2.47 (d, 2 H, CH_2_, *J* = 6.5 Hz), 2.77 (d, 2 H, CH_2_, *J* = 6.4 Hz), 4.14 (t, 2 H, CH_2_–N, *J* = 7.0 Hz), 4.21 (s, 2 H, CH_2_-Cl), 4.22 (t, 2 H, CH_2_–O, *J* = 7.0 Hz), 5.00 (s, 1 H, acridinedione-H_9_), 7.04–7.13 (m, 2 H, Ar–H), 7.26 (d, 1 H, Ar–H, *J* = 8.2 Hz), 7.28 (s, 1 H, Ar–H). MS (ESI+) *m/z*: Expected for C_27_H_31_BrClNO_4_ [M + H]^+^: 550.11, found 550.

##### 2-(9-(3,4-dimethoxyphenyl)-3,3,6,6-tetramethyl-1,8-dioxo-2,3,4,5,6,7,8,9-octahydroacridin-10(1H)-yl) ethyl 2-chloroacetate (6h)

Compound **(6h)** was obtained as white powder, (2.4 g, 90.5% yield); **mp.** 211–213  °C. IR (KBr) *ν*_max_ (cm^−1^): 3001 (=C–H, *sp*^2^); 2957, 2870 (C–H, *sp*^3^); 1759 (C=O, ester); 1628 (C=O, conjugated ketone, acridinedione); 1570, 1511, 1462 (C=C); 1332 (C–N stretching); 1091 (C–O stretching). ^1^H NMR (400 MHz, DMSO-*d*_6_) *δ* (ppm): 0.95 (s, 6 H, 2 CH_3_), 1.03 (s, 6 H, 2 CH_3_), 2.11 (d, 2 H, CH_2_, *J* = 6.5 Hz), 2.18 (d, 2 H, CH_2_, *J* = 6.4 Hz), 2.46 (d, 2 H, CH_2_, *J* = 6.0 Hz), 2.75 ( d, 2 H, CH_2_, *J* = 6.0 Hz), 3.64 (s, 3 H, OCH_3_), 3.66 (s, 3 H, OCH_3_), 4.11 (t, 2 H, CH_2_–N, *J* = 7.0 Hz), 4.13 (s, 2 H, CH_2_–Cl), 4.20 (t, 2 H, CH_2_–O, *J* = 7.0 Hz), 4.98 (s, 1 H, acridinedione-H_9_), 6.51 (d, 1 H, Ar–H, *J* = 8.2 Hz), 6.67 (d, 1 H, Ar–H, *J* = 8.2 Hz), 6.73 (s, 1 H, Ar–H). ^13^C NMR (100 MHz, DMSO-*d*_6_) *δ* (ppm): 195.84 (2 C=O, acridinedione), 167.53 (C=O, ester), 151.72, 148.49 (C-OCH_3_), 147.33 (C–OCH_3_), 139.00, 118.92, 112.09, 111.50 (Ar–C), 114.33, 65.76, 55.84, 55.75, 49.96, 42.64, 41.32, 39.64 (under DMSO-*d*_6_), 32.51, 30.42, 29.29 (2 CH_3_), 27.53 (2 CH_3_). MS (ESI +) *m/z*: Expected for C_29_H_36_ClNO_6_ [M + H]^+^: 530.22, found 530.

##### 2-(9-(4-methoxyphenyl)-3,3,6,6-tetramethyl-1,8-dioxo-2,3,4,5,6,7,8,9-octahydroacridin-10(1H)-yl) ethyl 2-chloroacetate (6i)

Compound **(6i)** was obtained as white powder, (2.2 g, 88% yield); **mp.** 185–187 °C. IR (KBr) *ν*_max_ (cm^−1^): 3037 (=C–H, *sp*^2^); 2959, 2870 (C–H, *sp*^3^); 1765 (C=O, ester); 1627 (C=O, conjugated ketone, acridinedione); 1566, 1508, 1467 (C=C); 1326 (C–N stretching); 1095 (C–O stretching). ^1^H NMR (400 MHz, DMSO-*d*_6_) *δ* (ppm): 0.94 (s, 6 H, 2 CH_3_), 1.03 (s, 6 H, 2 CH_3_), 2.10 (d, 2 H, CH_2_, *J* = 6.1 Hz), 2.17 (d, 2 H, CH_2_, *J* = 6.2 Hz), 2.45 (d, 2 H, CH_2_, *J* = 6.9 Hz), 2.75 (d, 2 H, CH_2_, *J* = 6.9 Hz), 3.66 (s, 3 H, OCH_3_), 4.12 (t, 2 H, CH_2_-N, *J* = 7.3 Hz), 4.15 (s, 2 H, CH_2_–Cl), 4.22 (t, 2 H, CH_2_–O, *J* = 7.2 Hz), 4.97 (s, 1 H, acridinedione-H_9_), 6.68 (d, 2 H, Ar–H, *J* = 8.2 Hz), 6.99 (d, 2 H, Ar–H, *J* = 8.2 Hz). ^13^C NMR (100 MHz, DMSO-*d*_6_) *δ* (ppm): 195.74 (2 C=O, acridinedione), 167.55 (C=O, ester), 157.64 (C–OCH_3_), 151.65, 138.41, 128.41, 113.46 (Ar–C), 114.56, 65.95, 55.26 (OCH_3_), 49.96, 42.66, 41.37, 39.67 (under DMSO-*d*_6_), 32.55, 30.32, 29.15 (2 CH_3_), 27.73 (2 CH_3_). MS (ESI +) *m/z*: Expected for C_28_H_34_ClNO_5_ [M + H]^+^: 500.21, found 500.

##### 2-(3,3,6,6-tetramethyl-1,8-dioxo-9-(p-tolyl)-2,3,4,5,6,7,8,9-octahydroacridin-10(1H)-yl) ethyl 2-chloroacetate (6j)

Compound **(6j)** was obtained as white powder, (2 g, 82.6% yield); **mp.** 198–200 °C. IR (KBr) *ν*_max_ (cm^−1^): 3032 (=C–H, *sp*^2^); 2953, 2868 (C–H, *sp*^3^); 1764 (C=O, ester); 1631 (C=O, conjugated ketone, acridinedione); 1571, 1506, 1462 (C=C); 1334 (C–N stretching); 1092 (C–O stretching). ^1^H NMR (400 MHz, DMSO-*d*_6_) *δ* (ppm): 0.93 (s, 6 H, 2 CH_3_), 1.03 (s, 6 H, 2 CH_3_), 2.10 (d, 2 H, CH_2_, *J* = 6.4 Hz), 2.17 (d, 2 H, CH_2_, *J* = 6.4 Hz), 2.19 (s, 3 H, CH_3_-Ar), 2.45 (d, 2 H, CH_2_, *J* = 6.8 Hz), 2.75 (d, 2 H, CH_2_, *J* = 6.7 Hz), 4.08 (s, 2 H, CH_2_–Cl), 4.12 (t, 2 H, CH_2_–N, *J* = 7.5 Hz), 4.21 (t, 2 H, CH_2_–O, *J* = 7.5 Hz), 5.00 (s, 1 H, acridinedione-H_9_), 6.91–6.97 (m, 4 H, Ar–H). MS (ESI +) *m/z*: Expected for C_28_H_34_ClNO_4_ [M + H]^+^: 484.22, found 484.

##### 2-(9-(3-chlorophenyl)-3,3,6,6-tetramethyl-1,8-dioxo-2,3,4,5,6,7,8,9-octahydroacridin-10(1H)-yl) ethyl 2-chloroacetate (6k)

Compound **(6k)** was obtained as white powder, (2.2 g, 87.2% yield); **mp.** 194–196 °C. IR (KBr) *ν*_max_ (cm^−1^): 3062 (=C–H, *sp*^2^); 2960, 2871 (C–H, *sp*^3^); 1764 (C=O, ester); 1630 (C=O, conjugated ketone, acridinedione); 1569, 1470 (C=C); 1333 (C–N stretching); 1091 (C–O stretching). ^1^H NMR (400 MHz, DMSO-*d*_6_) *δ* (ppm): 0.93 (s, 6 H, 2 CH_3_), 1.03 (s, 6 H, 2 CH_3_), 2.12 (d, 2 H, CH_2_, *J* = 6.2 Hz), 2.18 (d, 2 H, CH_2_, *J* = 6.2 Hz), 2.48 (d, 2 H, CH_2_, *J* = 6.7 Hz), 2.77 (d, 2 H, CH_2_, *J* = 6.7 Hz), 4.14 (t, 2 H, CH_2_-N, *J* = 7.0 Hz), 4.22 (s, 2 H, CH_2_-Cl), 4.23 (t, 2 H, CH_2_–O, *J* = 7.0 Hz), 5.01 (s, 1 H, acridinedione-H_9_), 7.02 (d, 1 H, Ar–H, *J* = 8.2 Hz), 7.12–7.19 (m, 3 H, Ar–H). MS (ESI +) *m/z*: Expected for C_27_H_31_Cl_2_NO_4_ [M + H]^+^: 504.16, found 504.

##### 2-(9-(benzo[d][1,3] dioxol-5-yl)-3,3,6,6-tetramethyl-1,8-dioxo-2,3,4,5,6,7,8,9-octahydroacridin-10(1H)-yl)ethyl 2-chloroacetate (6L)

Compound **(6L)** was obtained as white powder, (2.3 g, 89.5% yield); **mp.** 176–178 °C. IR (KBr) *ν*_max_ (cm^−1^): 3074, 3002 (=C–H, *sp*^2^); 2957, 2880 (C–H, *sp*^3^); 1760 (C=O, ester); 1644 (C=O, conjugated ketone, acridinedione); 1572, 1482 (C=C); 1332 (C–N stretching); 1090 (C–O stretching). ^1^H NMR (400 MHz, DMSO-*d*_6_) *δ* (ppm): 0.95 (s, 6H, 2 CH_3_), 1.02 (s, 6H, 2 CH_3_), 2.11 (d, 2H, CH_2_, *J* = 6.2 Hz), 2.17 (d, 2H, CH_2_, *J* = 6.2 Hz), 2.45 (d, 2H, CH_2_, *J* = 6.6 Hz), 2.76 (d, 2H, CH_2_, *J* = 6.7 Hz), 4.12 (t, 2H, CH_2_–N, *J* = 7.0 Hz), 4.22 (s, 2H, CH_2_-Cl), 4.29 (t, 2H, CH_2_–O, *J* = 7.0 Hz), 4.95 (s, 1H, acridinedione-H_9_), 5.90 (s, 2H, CH_2_, dioxole-H), 6.53–6.66 (m, 3H, Ar–H). ^13^C NMR (100 MHz, DMSO-*d*_6_) *δ* (ppm): 195.84 (2 C=O, acridinedione), 167.47 (C=O, ester), 151.86, 147.18, 145.45, 140.54, 120.26, 114.40, 108.03, 107.96, 101.02, 65.97, 49.91, 42.73, 41.31, 39.68 (under DMSO-*d*_6_), 32.57, 30.90, 29.04 (2 CH_3_), 27.78 (2 CH_3_). MS (ESI +) *m/z*: Expected for C_28_H_32_ClNO_6_ [M + H]^+^: 514.19, found 514.

#### Synthesis of 2-(9-(aryl)-3,3,6,6-tetramethyl-1,8-dioxo-2,3,4,5,6,7,8,9-octahydroacridin-10(1H)-yl) ethyl2-(1,3-dioxoisoindolin-2-yl)acetate (8a-L)

In a 50 mL round flask, phthalimide **7** (0.32 g, 2.2 mmol) was dissolved in *N*,*N*-dimethyl formamide (6 mL) containing potassium carbonate (0.41 g, 3 mmol) as a catalyst, then **6a–L** (2 mmol) was added. The reaction mixture was stirred at room temperature for 5 h at room temperature. The reaction was monitored by TLC. After completion, the reaction mixture was dropped into saline water 8% (100 mL) with stirring untill complete precipitation. The product was filtered off, washed with water, and dried. The crude products were recrystallized from methanol to obtain pure compounds **8a–L**.

##### 2-(9-(4-bromophenyl)-3,3,6,6-tetramethyl-1,8-dioxo-2,3,4,5,6,7,8,9-octahydroacridin-10(1H)-yl) ethyl 2-(1,3-dioxoisoindolin-2-yl)acetate (8a)

Compound **(8a)** was obtained as white powder, (1.1 g, 83.38% yield); **mp.** 215–218 °C. IR (KBr) *ν*_max_ (cm^−1^): 3061 (=C–H, *sp*^2^); 2957, 2870 (C–H, *sp*^3^); 1754 (C=O, ester); 1723 (C=O, phthalimide); 1633 (C=O, conjugated ketone, acridinedione); 1573, 1485, 1468 (C=C); 1330 (C–N stretching); 1089 (C–O stretching). ^1^H NMR (400 MHz, DMSO-*d*_6_) *δ* (ppm): 0.68 (s, 6 H, 2 CH_3_), 0.79 (s, 6 H, 2 CH_3_), 1.90 (d, 2 H, CH_2_, *J* = 6.1 Hz), 1.93 (d, 2 H, CH_2_, *J* = 6.1 Hz), 2.22 (d, 2 H, CH_2_, *J* = 6.5 Hz), 2.50 (d, 2 H, CH_2_, *J* = 6.4 Hz), 3.90 (t, 2 H, CH_2_–N, *J* = 7.1 Hz), 4.17 (t, 2 H, CH_2_–O, *J* = 7.1 Hz), 4.03 (s, 2 H, CH_2_-phthalimide), 4.74 (s, 1H, acridinedione-H_9_), 7.15 (d, 4H, Ar–H, *J* = 8.3 Hz), 7.60–7.72 (m, 4H, Ar–H, phthalimide). MS (ESI +) *m/z*: Expected for C_35_H_35_BrN_2_O_6_ [M + H]^+^: 661.17, found 661.

##### 2-(9-(4-chlorophenyl)-3,3,6,6-tetramethyl-1,8-dioxo-2,3,4,5,6,7,8,9-octahydroacridin-10(1H)-yl) ethyl 2-(1,3-dioxoisoindolin-2-yl) acetate (8b)

Compound **(8b)** was obtained as white powder, (1 g, 81.28% yield); **mp.** 221–223 °C. IR (KBr) *ν*_max_ (cm^−1^): 3062 (=C–H, *sp*^2^); 2958, 2871 (C–H, *sp*^3^); 1755 (C=O, ester); 1722 (C=O, phthalimide); 1633 (C=O, conjugated ketone, acridinedione); 1573, 1487, 1468 (C=C); 1338 (C–N stretching); 1089 (C–O stretching). ^1^H NMR (400 MHz, DMSO-*d*_6_) *δ* (ppm): 0.69 (s, 6 H, 2 CH_3_), 0.80 (s, 6 H, 2 CH_3_), 1.91 (d, 2H, CH_2_, *J* = 6.3 Hz), 1.94 (d, 2H, CH_2_, *J* = 6.3 Hz), 2.23 (d, 2H, CH_2_, *J* = 6.7 Hz), 2.51 (d, 2H, CH_2_, *J* = 6.6 Hz), 3.91 (t, 2H, CH_2_-N, *J* = 7.1 Hz), 4.04 (t, 2H, CH_2_–O, *J* = 7.1 Hz), 4.17 (s, 2H, CH_2_-phthalimide), 4.76 (s, 1H, acridinedione-H_9_), 6.92 (d, 2H, Ar–H, *J* = 8.4 Hz), 7.04 (d, 2H, Ar–H, *J* = 8.4 Hz), 7.68–7.73 (m, 4H, Ar–H, phthalimide). MS (ESI +) *m/z*: Expected for C_35_H_35_ClN_2_O_6_ [M + H]^+^: 615.22, found 615.

##### 2-(9-(4-fluorophenyl)-3,3,6,6-tetramethyl-1,8-dioxo-2,3,4,5,6,7,8,9-octahydroacridin-10(1H)-yl) ethyl 2-(1,3-dioxoisoindolin-2-yl) acetate (8c)

Compound **(8c)** was obtained as white powder, (1.1 g, 91.8% yield); **mp.** 198–200  °C. IR (KBr) *ν*_max_ (cm^−1^): 3064 (=C–H, *sp*^2^); 2958, 2871 (C–H, *sp*^3^); 1756 (C=O, ester); 1723 (C=O, phthalimide); 1633 (C=O, conjugated ketone, acridinedione); 1572, 1504, 1468 (C=C); 1337 (C–N stretching); 1090 (C–O stretching). ^1^H NMR (400 MHz, DMSO-*d*_6_) *δ* (ppm): 0.93 (s, 6 H, 2 CH_3_), 1.05 (s, 6 H, 2 CH_3_), 2.11 (d, 2 H, CH_2_, *J* = 6.1 Hz), 2.18 (d, 2 H, CH_2_, *J* = 6.1 Hz), 2.47 (d, 2 H, CH_2_, *J* = 6.7 Hz), 2.77 (d, 2 H, CH_2_, *J* = 6.7 Hz), 4.16 (t, 2 H, CH_2_-N, *J* = 7.1 Hz), 4.27 (t, 2 H, CH_2_–O, *J* = 7.1 Hz), 4.39 (s, 2 H, CH_2_-phthalimide), 5.02 (s, 1 H, acridinedione-H_9_), 7.06 (d, 2 H, Ar–H, *J* = 8.0 Hz), 7.16 (d, 2 H, Ar–H, *J* = 8.0 Hz), 7.92–7.97 (m, 4 H, Ar–H, phthalimide). MS (ESI +) *m/z*: Expected for C_35_H_35_FN_2_O_6_ [M + H]^+^: 599.25, found 599.

##### 2-(3,3,6,6-tetramethyl-1,8-dioxo-9-phenyl-2,3,4,5,6,7,8,9-octahydroacridin-10(1H)-yl) ethyl 2-(1,3-dioxoisoindolin-2-yl) acetate (8d)

Compound **(8d)** was obtained as white powder, (1 g, 86.1% yield); **mp.** 210–212  °C. IR (KBr) *ν*_max_ (cm^−1^): 3059, 3028 (=C–H, *sp*^2^); 2958, 2871 (C–H, *sp*^3^); 1755 (C=O, ester); 1723 (C=O, phthalimide); 1632 (C=O, conjugated ketone, acridinedione); 1571, 1489, 1468 (C=C); 1336 (C–N stretching); 1089 (C–O stretching). ^1^H NMR (400 MHz, DMSO-*d*_6_) *δ* (ppm):): 0.69 (s, 6 H, 2 CH_3_), 0.80 (s, 6 H, 2 CH_3_), 1.79 (d, 2 H, CH_2_, *J* = 6.2 Hz), 1.90 (d, 2 H, CH_2_, *J* = 6.2 Hz), 2.20 (d, 2 H, CH_2_, *J* = 6.7 Hz), 2.52 (d, 2 H, CH_2_, *J* = 6.7 Hz), 3.91 (t, 2 H, CH_2_-N, *J* = 7.6 Hz), 4.00 (t, 2 H, CH_2_-O, *J* = 7.5 Hz), 4.10 (s, 2 H, CH_2_-phthalimide), 4.79 (s, 1 H, acridinedione-H_9_), 6.75–6.98 (m, 5 H, Ar–H), 7.52–7.71 (m, 4 H, Ar–H, phthalimide). MS (ESI +) m/z: Expected for C_35_H_36_N_2_O_6_ [M + H]^+^: 581.26, found 581.

##### 2-(9-(3,5-dimethylphenyl)-3,3,6,6-tetramethyl-1,8-dioxo-2,3,4,5,6,7,8,9-octahydroacridin-10(1H)-yl) ethyl 2-(1,3-dioxoisoindolin-2-yl) acetate (8e)

Compound **(8e)** was obtained as white powder, (1.1 g, 90.3% yield); **mp.** 189–191  °C. IR (KBr) *ν*_max_ (cm^−1^): 3028 (=C–H, *sp*^2^); 2958, 2870 (C–H, *sp*^3^); 1760 (C=O, ester); 1726 (C=O, phthalimide); 1631 (C=O, conjugated ketone, acridinedione); 1571, 1499, 1468 (C=C); 1336 (C–N stretching); 1086 (C–O stretching). ^1^H NMR (400 MHz, DMSO-*d*_6_) *δ* (ppm): 0.89 (s, 6 H, 2 CH_3_), 1.04 (s, 6 H, 2 CH_3_), 2.04 (d, 2 H, CH_2_, *J* = 6.0 Hz), 2.10 (s, 3 H, CH_3_-Ar), 2.14 (d, 2 H, CH_2_, *J* = 6.0 Hz), 2.50 (d, 2 H, CH_2_, *J* = 6.5 Hz), 2.69 (s, 3 H, CH_3_-Ar), 2.73 (d, 2 H, CH_2_, *J* = 6.5 Hz), 4.18 (t, 2 H, CH_2_-N, *J* = 7.2 Hz), 4.40 (t, 2 H, CH_2_–O, *J* = 7.3 Hz), 4.46 (s, 2 H, CH_2_-phthalimide), 4.94 (s, 1 H, acridinedione-H_9_), 6.72–6.82 (m, 3 H, Ar–H), 7.90–7.98 (m, 4 H, Ar–H, phthalimide). ^13^C NMR (100 MHz, DMSO-*d*_6_) *δ* (ppm): 195.81 (2 C=O, acridinedione), 168.13 (C=O, ester), 167.51 (2 C=O, phthalimide), 151.11, 146.36, 135.46, 134.80, 133.08, 132.94, 131.76, 129.61, 128.83, 126.63, 124.02, 123.43 (Ar–C), 116.35, 65.65, 49.96, 43.08, 39.68 (under DMSO-d6), 39.29 (under DMSO-*d*_6_), 32.54, 29.22, 28.81 (2 CH_3_), 27.23 (2 CH_3_), 21.15, 19.70. MS (ESI +) m/z: Expected for C_37_H_40_N_2_O_6_ [M + H]^+^: 609.29, found 609.

##### 2-(9-([1,1'-biphenyl]-4-yl)-3,3,6,6-tetramethyl-1,8-dioxo-2,3,4,5,6,7,8,9-octahydroacridin-10(1H)-yl) ethyl 2-(1,3-dioxoisoindolin-2-yl) acetate (8f)

Compound **(8f)** was obtained as white powder, (1 g, 76.1% yield); **mp.** 250–252  °C. IR (KBr) *ν*_max_ (cm^−1^): 3059, 3029 (=C–H, *sp*^2^); 2957, 2870 (C–H, *sp*^3^); 1754 (C=O, ester); 1723 (C=O, phthalimide); 1633 (C=O, conjugated ketone, acridinedione); 1573, 1485, 1468 (C=C); 1330 (C–N stretching); 1089 (C–O stretching). ^1^H NMR (400 MHz, DMSO-*d*_6_) *δ* (ppm): 0.95 (s, 6 H, 2 CH_3_), 1.05 (s, 6 H, 2 CH_3_), 2.10 (d, 2 H, CH_2_, *J* = 6.4 Hz), 2.17 (d, 2 H, CH_2_, *J* = 6.4 Hz), 2.52 (d, 2 H, CH_2_, *J* = 6.7 Hz), 2.78 (d, 2 H, CH_2_, *J* = 6.6 Hz), 4.17 (t, 2 H, CH_2_–N, *J* = 7.2 Hz), 4.29 (t, 2 H, CH_2_–O, *J* = 7.3 Hz), 4.45 (s, 2 H, CH_2_-phthalimide), 5.06 (s, 1 H, acridinedione-H_9_), 7.23–7.25 (d, 2 H, Ar–H, phenyl, *J* = 8.0 Hz), 7.32 (t, 1 H, Ar–H, phenyl, *J* = 8.2 Hz), 7.42 (t, 2 H, Ar–H, phenyl, *J* = 8.1 Hz), 7.53 (d, 2 H, Ar–H, phenylene, *J* = 8.3 Hz), 7.64 (d, 2 H, Ar–H, phenylene, *J* = 8.2 Hz),7.89–7.96 (m, 4 H, Ar–H, phthalimide). MS (ESI +) *m/z*: Expected for C_41_H_40_N_2_O_6_ [M + H]^+^: 657.29, found 657.

##### 2-(9-(3-bromophenyl)-3,3,6,6-tetramethyl-1,8-dioxo-2,3,4,5,6,7,8,9-octahydroacridin-10(1H)-yl) ethyl 2-(1,3-dioxoisoindolin-2-yl) acetate (8j)

Compound **(8j)** was obtained as white powder, (1.1 g, 83.3% yield); **mp.** 225–227 °C. IR (KBr) *ν*_max_ (cm^−1^): 3062 (=C–H, *sp*^2^); 2958, 2871 (C–H, *sp*^3^); 1755 (C=O, ester); 1723 (C=O, phthalimide); 1633 (C=O, conjugated ketone, acridinedione); 1571, 1469 (C=C); 1338 (C–N stretching); 1089 (C–O stretching). ^1^H NMR (400 MHz, DMSO-*d*_6_) *δ* (ppm): ): 0.94 (s, 6 H, 2 CH_3_), 1.04 (s, 6 H, 2 CH_3_), 2.13 (d, 2 H, CH_2_, *J* = 6.3 Hz), 2.17 (d, 2 H, CH_2_, *J* = 6.3 Hz), 2.47 (d, 2 H, CH_2_, *J* = 6.5 Hz), 2.76 (d, 2 H, CH_2_, *J* = 6.5 Hz), 4.16 (t, 2 H, CH_2_–N, *J* = 7.0 Hz), 4.27 (t, 2 H, CH_2_–O, *J* = 7.1 Hz), 4.37 (s, 2 H, CH_2_-phthalimide), 4.99 (s, 1 H, acridinedione-H_9_), 7.07–7.09 (d, 1 H, Ar–H), 7.19–7.23 (t, 1 H, Ar–H, *J* = 8.0 Hz), 7.26–7.28 (d, 1 H, Ar–H, *J* = 8.1 Hz), 7.37 (s, 1 H, Ar–H), 7.90–7.97 (m, 4 H, Ar–H, phthalimide). MS (ESI +) m/z: Expected for C_35_H_35_BrN_2_O_6_ [M + H]^+^: 659.17, found 659.

##### 2-(9-(3,4-dimethoxyphenyl)-3,3,6,6-tetramethyl-1,8-dioxo-2,3,4,5,6,7,8,9-octahydroacridin-10(1H)-yl) ethyl 2-(1,3-dioxoisoindolin-2-yl) acetate (8h)

Compound **(8h)** was obtained as white powder, (1.1 g, 85.8% yield); **mp.** 179–181 °C. IR (KBr) *ν*_max_ (cm^−1^): 3066 (=C–H, *sp*^2^); 2953, 2870 (C–H, *sp*^3^); 1753 (C=O, ester); 1720 (C=O, phthalimide); 1649 (C=O, conjugated ketone, acridinedione); 1573, 1514, 1466 (C=C); 1332 (C–N stretching); 1087 (C–O stretching). ^1^H NMR (400 MHz, DMSO-*d*_6_) *δ* (ppm): ): 0.95 (s, 6 H, 2 CH_3_), 1.04 (s, 6 H, 2 CH_3_), 2.11 (d, 2 H, CH_2_, *J* = 6.2 Hz), 2.18 (d, 2 H, CH_2_, *J* = 6.3 Hz), 2.46 (d, 2 H, CH_2_, *J* = 6.7 Hz), 2.75 (d, 2 H, CH_2_, *J* = 6.7 Hz), 3.67 (s, 3 H, OCH_3_), 3.69 (s, 3 H, OCH_3_), 4.13 (t, 2 H, CH_2_–N, *J* = 7.0 Hz), 4.25 (t, 2 H, CH_2_–O, *J* = 7.0 Hz), 4.39 (s, 2 H, CH_2_-phthalimide), 4.97 (s, 1 H, acridinedione-H_9_), 6.57–7.59 (d, 1 H, Ar–H, *J* = 8.4 Hz), 6.80 (s, 1 H, Ar–H), 6.82 (d, 1 H, Ar–H, *J* = 8.5 Hz), 7.90–7.95 (m, 4 H, Ar–H, phthalimide). MS (ESI+) m/z: Expected for C_37_H_40_N_2_O_8_ [M + H]^+^: 641.28, found 641.

##### 2-(9-(4-methoxyphenyl)-3,3,6,6-tetramethyl-1,8-dioxo-2,3,4,5,6,7,8,9-octahydroacridin-10(1H)-yl) ethyl 2-(1,3-dioxoisoindolin-2-yl) acetate (8i)

Compound **(8i)** was obtained as white powder, (1 g, 81.8% yield); **mp.** 211–213 °C. IR (KBr) *ν*_max_ (cm^−1^): 3028 (=C–H, *sp*^2^); 2955, 2871 (C–H, *sp*^3^); 1749 (C=O, ester); 1719 (C=O, phthalimide); 1623 (C=O, conjugated ketone, acridinedione); 1565, 1508, 1469 (C=C); 1337 (C–N stretching); 1088 (C–O stretching). ^1^H NMR (400 MHz, DMSO-*d*_6_) *δ* (ppm): ): 0.93 (s, 6H, 2 CH_3_), 1.04 (s, 6H, 2 CH_3_), 2.09 (d, 2 H, CH_2_, *J* = 6.2 Hz), 2.17 (d, 2 H, CH_2_, *J* = 6.2 Hz), 2.45 (d, 2H, CH_2_, *J* = 6.5 Hz), 2.75 (d, 2H, CH_2_, *J* = 6.4 Hz), 3.69 (s, 3H, OCH_3_), 4.15 (t, 2H, CH_2_–N, *J* = 7.2 Hz), 4.25 (t, 2H, CH_2_-O, *J* = 7.2 Hz), 4.40 (s, 2H, CH_2_-phthalimide), 4.96 (s, 1H, acridinedione-H_9_), 6.78 (d, 2 H, Ar–H, *J* = 8.0 Hz), 7.06 (d, 2H, Ar–H, *J* = 8.0 Hz), 7.90–7.97 (m, 4H, Ar–H, phthalimide). ^13^C NMR (100 MHz, DMSO-*d*_6_) *δ* (ppm): 195.68 (2 C=O, acridinedione), 168.05 (C=O, ester), 167.56 (2 C=O, phthalimide), 157.70 (C–OCH_3_), 151.43, 138.84, 135.44, 134.81, 133.08, 131.77, 128.61, 124.01, 123.42, 114.67 (Ar–C), 113.57, 65.93, 55.26 (OCH_3_), 49.95, 42.75, 39.65 (under DMSO-*d*_6_), 39.20 (under DMSO-*d*_6_), 32.57, 30.78, 29.23 (2 CH_3_), 27.56 (2 CH_3_). MS (ESI+) *m/z*: Expected for C_36_H_38_N_2_O_7_ [M + H]^+^: 611.27, found 611.

##### 2-(3,3,6,6-tetramethyl-1,8-dioxo-9-(p-tolyl)-2,3,4,5,6,7,8,9-octahydroacridin-10(1H)-yl) ethyl 2-(1,3-dioxoisoindolin-2-yl) acetate (8j)

Compound **(8j)** was obtained as white powder, (1 g, 84% yield); **mp.** 208–210  °C. IR (KBr) *ν*_max_ (cm^−1^): 3028 (=C–H, *sp*^2^); 2957, 2870 (C–H, *sp*^3^); 1755 (C=O, ester); 1723 (C=O, phthalimide); 1632 (C=O, conjugated ketone, acridinedione); 1572, 1509, 1468 (C=C); 1337 (C–N stretching); 1089 (C–O stretching). ^1^H NMR (400 MHz, DMSO-*d*_6_) *δ* (ppm): ): 0.92 (s, 6 H, 2 CH_3_), 1.04 (s, 6 H, 2 CH_3_), 2.09 (d, 2 H, CH_2_, *J* = 6.4 Hz), 2.17 (d, 2 H, CH_2_, *J* = 6.4 Hz) , 2.21 (s, 3 H, CH_3_-Ar), 2.45 (d, 2 H, CH_2_, *J* = 6.9 Hz), 2.75 (d, 2 H, CH_2_, *J* = 6.8 Hz), 4.15 (t, 2 H, CH_2_-N, *J* = 7.2 Hz), 4.24 (t, 2 H, CH_2_-O, *J* = 7.2 Hz), 4.35 (s, 2 H, CH_2_-phthalimide), 4.98 (s, 1 H, acridinedione-H_9_), 7.01 (m, 4 H, Ar–H), 7.91–7.98 (m, 4 H, Ar–H, phthalimide). ^13^C NMR (100 MHz, DMSO-*d*_6_) *δ* (ppm): 195.66 (2 C=O, acridinedione), 168.04 (C=O, ester), 167.54 (2 C=O, phthalimide), 151.61, 143.55, 135.45, 134.97, 134.82, 133.08, 131.78, 128.84, 127.47, 124.02, 123.43 (Ar–C), 114.56, 65.91, 49.94, 42.76, 39.64 (under DMSO-*d*_6_), 39.17 (under DMSO-*d*_6_), 32.59, 31.09, 29.20 (2 CH_3_), 27.60 (2 CH_3_), 21.07. MS (ESI+) m/z: Expected for C_36_H_38_N_2_O_6_ [M + H]^+^: 595.27, found 595.

##### 2-(9-(3-chlorophenyl)-3,3,6,6-tetramethyl-1,8-dioxo-2,3,4,5,6,7,8,9-octahydroacridin-10(1H)-yl) ethyl 2-(1,3-dioxoisoindolin-2-yl) acetate (8k)

Compound **(8k)** was obtained as white powder, (1.1 g, 89.4% yield); **mp.** 198–200 °C. IR (KBr) *ν*_max_ (cm^−1^): 3062 (=C–H, *sp*^2^); 2958, 2871 (C–H, *sp*^3^); 1755 (C=O, ester); 1723 (C=O, phthalimide); 1633 (C=O, conjugated ketone, acridinedione); 1571, 1469 (C=C); 1338 (C–N stretching); 1089 (C–O stretching). ^1^H NMR (400 MHz, DMSO-*d*_6_) *δ* (ppm): ): 0.94 (s, 6 H, 2 CH_3_), 1.04 (s, 6 H, 2 CH_3_), 2.13 (d, 2 H, CH_2_, *J* = 6.6 Hz), 2.19 (d, 2 H, CH_2_, *J* = 6.4 Hz), 2.47 (d, 2 H, CH_2_, *J* = 6.7 Hz), 2.77 (d, 2 H, CH_2_, *J* = 6.5 Hz), 4.16 (t, 2 H, CH_2_-N, *J* = 7.0 Hz), 4.27 (t, 2 H, CH_2_-O, *J* = 7.1 Hz), 4.36 (s, 2 H, CH_2_-phthalimide), 5.01 (s, 1 H, acridinedione-H_9_), 7.05 (d, 1 H, Ar–H, *J* = 8.0 Hz), 7.15 (d, 1 H, Ar–H, *J* = 8.2 Hz), 7.21 (s, 1 H, Ar–H), 7.27 (t, 1 H, Ar–H, *J* = 8.2 Hz), 7.90–7.97 (m, 4 H, Ar–H, phthalimide). MS (ESI +) m/z: Expected for C_35_H_35_ClN_2_O_6_ [M + H]^+^: 615.22, found 615.

##### 2-(9-(benzo[d][1,3] dioxol-5-yl)-3,3,6,6-tetramethyl-1,8-dioxo-2,3,4,5,6,7,8,9-octahydroacridin-10(1H)-yl)ethyl 2-(1,3-dioxoisoindolin-2-yl)acetate (8L)

Compound **8L** was obtained as white powder, (1.1 g, 88% yield); **mp.** 179–181 °C. IR (KBr) *ν*_max_ (cm^−1^): 3028 (=C–H, *sp*^2^); 2958, 2872 (C–H, *sp*^3^); 1755 (C=O, ester); 1722 (C=O, phthalimide); 1632 (C=O, conjugated ketone, acridinedione); 1572, 1502, 1485 (C=C); 1337 (C–N stretching); 1090 (C–O stretching). ^1^H NMR (400 MHz, DMSO-*d*_6_) *δ* (ppm): ): 0.94 (s, 6 H, 2 CH_3_), 1.03 (s, 6 H, 2 CH_3_), 2.12 (d, 2 H, CH_2_, *J* = 6.2 Hz), 2.17 (d, 2 H, CH_2_, *J* = 6.4 Hz), 2.45 (d, 2 H, CH_2_, *J* = 6.3 Hz), 2.74 (d, 2 H, CH_2_, *J* = 6.5 Hz), 4.14 (t, 2 H, CH_2_-N, *J* = 7.1 Hz), 4.25 (t, 2 H, CH_2_-O, *J* = 7.2 Hz), 4.39 (s, 2 H, CH_2_-phthalimde), 4.94 (s, 1 H, acridinedione-H_9_), 5.91 (s, 2 H, CH_2_, dioxole-H), 6.58 (d, 1 H, Ar–H, *J* = 8.5 Hz), 6.70 (s, 1 H, Ar–H), 6.76 (d, 1 H, Ar–H, *J* = 8.2 Hz), 7.90–7.97 (m, 4 H, Ar–H, phthalimide). ^13^C NMR (100 MHz, DMSO-*d*_6_) *δ* (ppm): 195.74 (2 C=O, acridinedione), 168.01 (C=O, ester), 167.54 (2 C=O, phthalimide), 151.65, 147.10, 145.50, 140.71, 135.43, 134.82, 133.08, 131.78, 124.01, 123.43, 120.14, 108.51, 108.09 (Ar–C), 114.55, 101.00, 65.77, 49.91, 42.87, 39.64 (under DMSO-d6), 39.14 (under DMSO-*d*_6_), 32.63, 31.26, 29.07 (2 CH_3_), 27.71 (2 CH_3_). MS (ESI +) *m/z*: Expected for C_36_H_36_N_2_O_8_ [M + H]^+^: 625.25, found 625.

#### Another method for the synthesis of 2-(9-(aryl)-3,3,6,6-tetramethyl-1,8-dioxo-2,3,4,5,6,7,8,9-octahydroacridin-10(1H)-yl) ethyl2-(1,3-dioxoisoindolin-2-yl) acetate (8a–L)

i. A mixture of equivalent amounts of phthalic anhydride **9** (0.05 mol) and glycine **10** (0.05 mol) were refluxed in toluene (40 mL) for 3 h. Toluene was removed under vacuum, followed by addition of water (80 mL) and concentrated HCl (1.5 mL). The mixture was stirred well, filtered off, and recrystallized from ethanol to give 2-(1,3-dioxoisoindolin-2yl) acetic acid **(11)**^46^. White powder, yield 95%, mp. 192–193 °C [Lit. mp. 191–192 °C].
ii. In 50 mL round-bottomed flask, thionyl chloride **12** (0.255 mmol) was added to 2-(1,3-dioxoisoindolin-2yl) acetic acid **(11)** (0.250 mmol) in ethyl acetate (20 mL) and refluxed for 1 h at 70–80 °C, the solvent was removed under vacuum to give phthaloylglycine chloride **13**^47^.
iii. In a 20 mL round-bottomed flask, *N*-phthaloylglycine chloride **13** (0.2 mmol) was added to compounds **4a–L** (0.125 mmol) including triethylamine (0.1 mmol) in dimethyl formamide (5 mL) then the reaction mixture was stirred at room temperature for 3 h. The progression of the reaction was monitored by TLC. After completion of the reaction, the mixture was dropped into water (100 mL) while stirring until complete precipitation. The product was filtered off, washed with water, and dried. The crude products were purified by crystallization in methanol to afford compounds **8a–L**.

### In vitro cytotoxicity assay

The cell lines were obtained from American Type Culture Collection (ATCC). With the usage of the Thiazolyl Blue Tetrazolium Bromide (MTT) method, various concentrations of the compounds (**8c, 8f, 8h, 8i**, and **8L**) were evaluated for their cytotoxicity against the HSF, H460, A431, A549, and MDA-MB-231 cell lines^48^. In brief HSF, H460, A431, A549 and MDA-MB-231 cell lines cells (10  × 10^3^) were cultured in 96 well plate for overnight at 37 °C, 5% CO_2_ and 88% humidity. The total volume of used DMEM supplemented medium and the synthesized compounds supernatant was 200 µL with final concentrations of 25, 50, 100, 150 and 200 µg/ml. The plate was incubated at 37 °C, 5% CO_2_ for 48 h. After incubation, debris and dead cells were removed by washing three times with fresh medium. Twenty mL of MTT solution (5 mg/ml of MTT in PBS buffer) was added to each well and shook for 5 min at 150 rpm to thoroughly mix the MTT into the media. The cells were incubated at 37 °C and 5% CO_2_ for 3–5 h to metabolize MTT by viable cells. 200 µL dimethyl sulfoxide (DMSO) was added to each well and shook again for 5 min at 150 rpm, and then the viability of the cells was calculated by measuring the optical density at 630 nm subtracted from optical density at 570 nm ^49^. The relative cell viability (%) was determined as the given formula:

$$ {\text{The}}\;{\text{relative}}\;{\text{cell}}\;{\text{viability}}\;\left( \% \right) = \left[ {{\text{OD}}_{{\text{S}}} /{\text{OD}}_{{\text{C}}} } \right] \times {1}00 $$

whereas OD_S_ is the mean optical density of sample, OD_C_ is the mean OD of control group. Results were displayed viability (%) as function of concentrations using Graph Pad Prism software version 7. Tumor-selectivity index (TS value) was determined by dividing the mean IC_50_ against normal cells by the mean IC_50_ against tumor cells. Thus obtained TS values seem to reflect the in vivo antitumor activity even though these cells were different types of cells (epithelial oral squamous cell carcinoma cell lines versus mesenchymal normal oral cells). In addition, the effect of the most potent antitumor compounds (**8f**) on the morphology of the tested cancer cells at their IC_50_ values was visualized under the phase-contrast microscopy (Olympus, Germany) in comparison with untreated (control) cells.

### The effect of compounds (8c, 8f, 8h, 8i, and 8L) on the expression of some tumor-regulating genes

In this study, the impact of potent antitumor compounds (**8c, 8f, 8h, 8i**, and **8L**) on the expression levels of four tumor regulating genes, namely Topoisomerase II beta (TOP2B), mitogen-activated protein kinase (MAP K) (p38), tumor protein interacted with the mouse double minute 2 homolog (p53), and Epidermal growth factor receptor (EGFR), was investigated in skin cancer cells (A431). Evaluation the relative changes in mRNA expression levels of four key genes by performing quantitative real-time PCR (qRT-PCR) before and after treatment for 2 days with IC_50_ concentrations of compounds (**8c, 8f, 8h, 8i**, and **8L**). Total RNA was extracted from the cells using the Gene JET RNA Purification Kit (Thermo Scientific, USA), followed by cDNA synthesis using the cDNA Synthesis Kit (Thermo Scientific, USA) protocol. SYBR green kit and specific primers (Forward/Reverse) were used for qRT-PCR: 5′-TCCGATCAGGAAGGCTAGAGTT-3’/5’-TCGGTCTCCTAAAAGCAGGC-3′ for p38, 5′-TAACAGTTCCTGCATGGGCGGC-3′/5′-AGGACAGGCACAAACACGCACC-3′ for p53, 5’-CTGCGTATTTCCATTCATC-3’/5’-CCTTGGGTCAGGTTTAGAG-3’ for TOP2B, and 5’-GGCTTTACTGCTGTACCTCC-3’/5’-CAAATGCTTTCTCCGCTCT-3’ for EGFR. The expression levels of the tested genes were determined by using the 2^−ΔΔCT^ equation to assess any changes in upregulation and/or downregulation of gene expression^50^.

### Docking studies

The binding orientations and interactions of the promising anticancer compounds (**8c**, **8f, 8h, 8i and 8L**) with four tumor-regulating proteins, (TOP2B, p38, p53, and EGFR) were simulated using the Molecular Operating Environment (MOE 2019) software. The three-dimensional (3D) structures of the selected proteins TOP2B, p38, p53, and EGFR are identified by their respective PDB IDs: 3QX3, 3HEG, 3JZK, and 1M17, respectively. Water molecules and repeating chains were removed. Protons were added, and the protein's energy was decreased. After that, the ligand pocket was isolated. The downloaded structure's validity was established by re-docking the downloaded ligand into the isolated ligand pocket. The obtained root mean square deviation (RMSD) was found to be less than 1.5. The potent anticancer compounds (**8c**, **8f, 8h, 8i,** and** 8L**) were prepared for docking by the MOE's chemical structure creation process. Afterwards, protons were added to the 3D structure. Force Field MMFF94x was then employed to further minimize the energy. The prepared structures were added to the built database. Recently synthesized compounds were docked using MOE, and their binding energies and mechanisms of binding were described^34^.

### Statistical analysis

The experiments were conducted in triplicate (n = 3), and the results are presented as the mean ± standard error of the mean (SEM). The statistical significance of the data was assessed using the one-way analysis of variance (ANOVA) followed by Tukey's post-hoc test for multiple comparisons. The statistical analysis was performed using the Graph Pad Prism software version 7. Differences between groups were considered statistically significant at p values < 0.05.

### Supplementary Information

Supplementary Information.

## Data Availability

All data generated or analyzed during this study are included in this published article and its supplementary information files.
