# Supplementary material for: Synthesis and cytotoxicity evaluation of novel 1,8-acridinedione derivatives bearing phthalimide moiety as potential antitumor agents
Source: Sci Rep. 2023 Sep 12;13:15093. doi: 10.1038/s41598-023-41970-0 (PMC10497682; doi:10.1038/s41598-023-41970-0)
Supplement: Supplementary file 1 — Supplementary Information. [file 41598_2023_41970_MOESM1_ESM.pdf]

Synthesis and Cytotoxicity Evaluation of Novel 1,8-Acridinedione Derivatives Bearing Phthalimide Moiety as Potential Antitumor Agents

Hassan A. Khatab [1,2], Sherif F. Hammad [1,3], Esmail M. El-Fakharany [4], Ahmed I. Hashem [2], Eman A. E. El-Helw [2,*]

[1] PharmD Program, Egypt-Japan University of Science and Technology (E-JUST), Alexandria, Egypt

[2] Chemistry Department, Faculty of Science, Ain Shams University, Cairo 11566, Egypt

[3] Department of Pharmaceutical Chemistry, Faculty of Pharmacy, Helwan University, Cairo, Egypt

[4] Protein Research Department, Genetic Engineering and Biotechnology Research Institute GEBRI, City of Scientific Research and Technological Applications, New Borg El Arab, Alexandria 21934, Egypt.

**E*-mail: eman.abdelrahman@sci.asu.edu.eg

Supporting information:

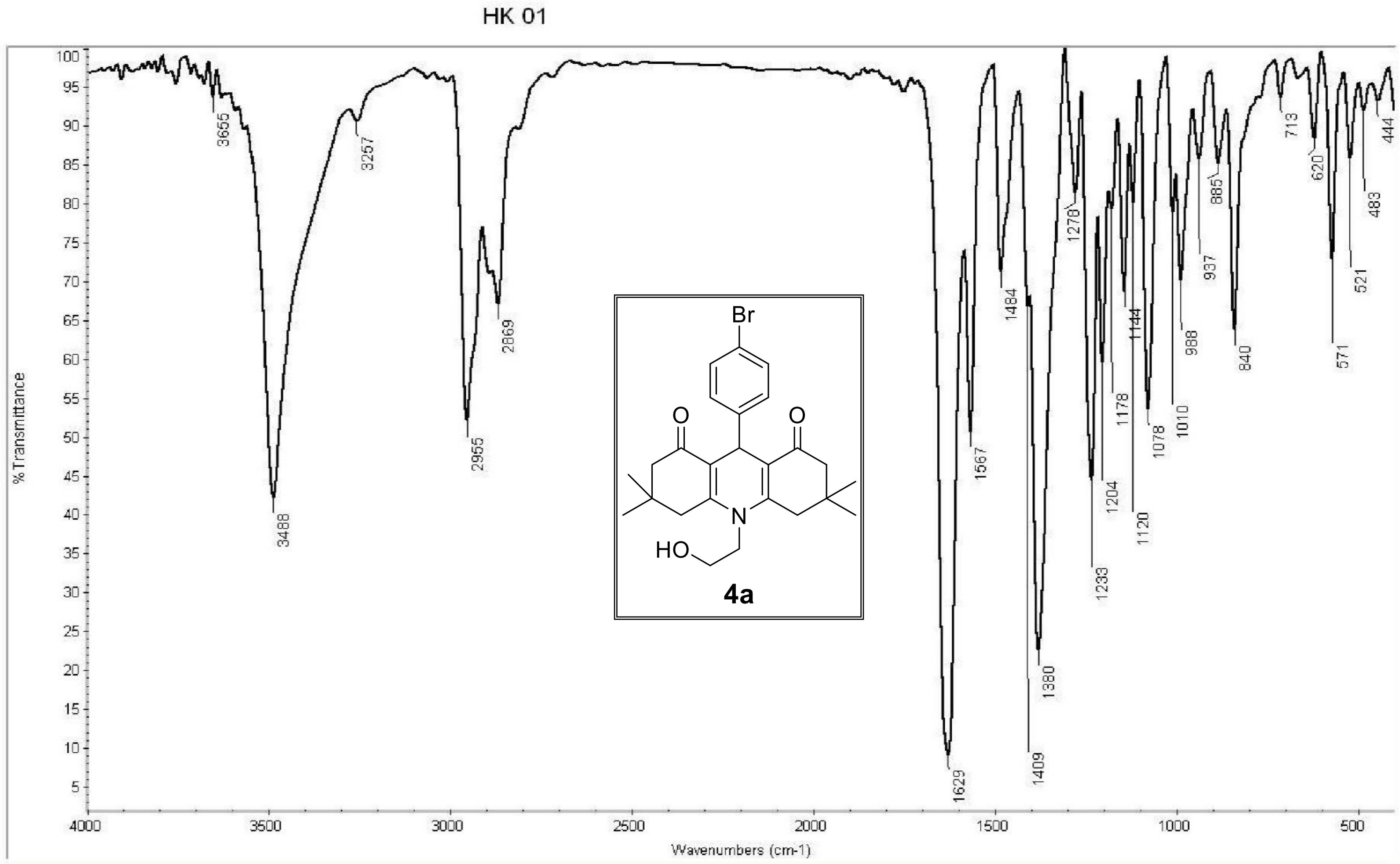

IR Spectrum of compound **4a**

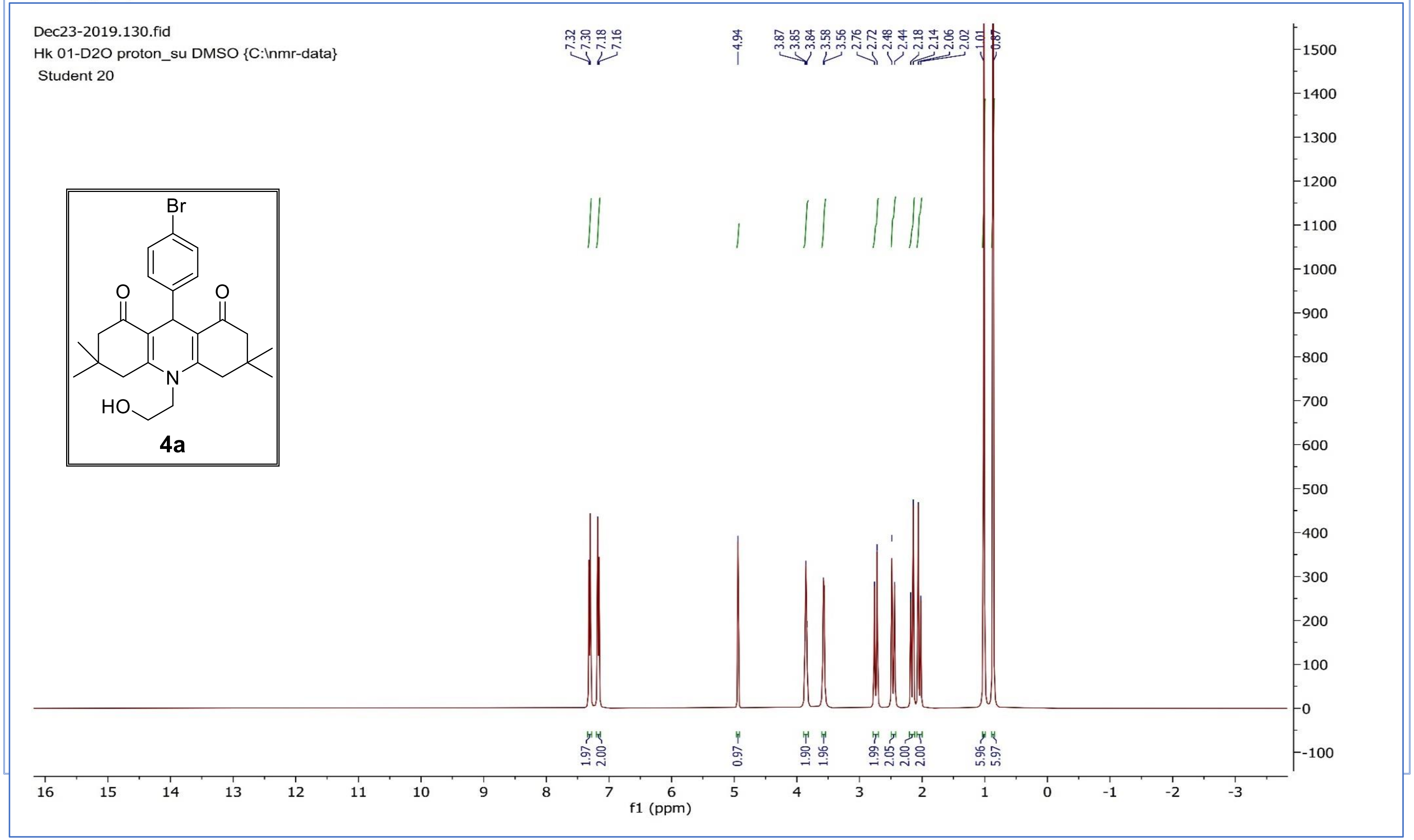

^{1}H NMR Spectrum of compound **4a**

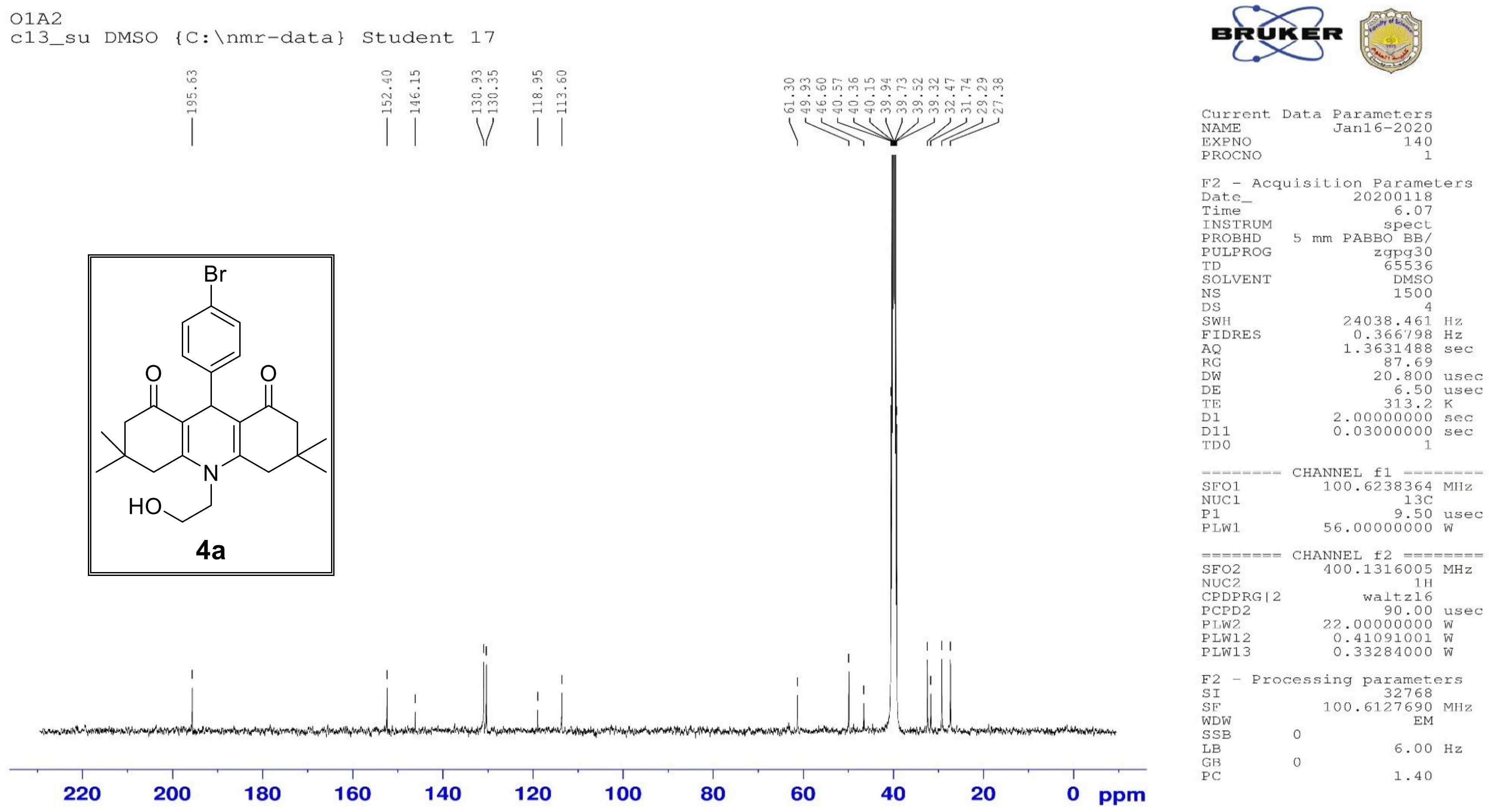

^{13}C NMR Spectrum of compound **4a**

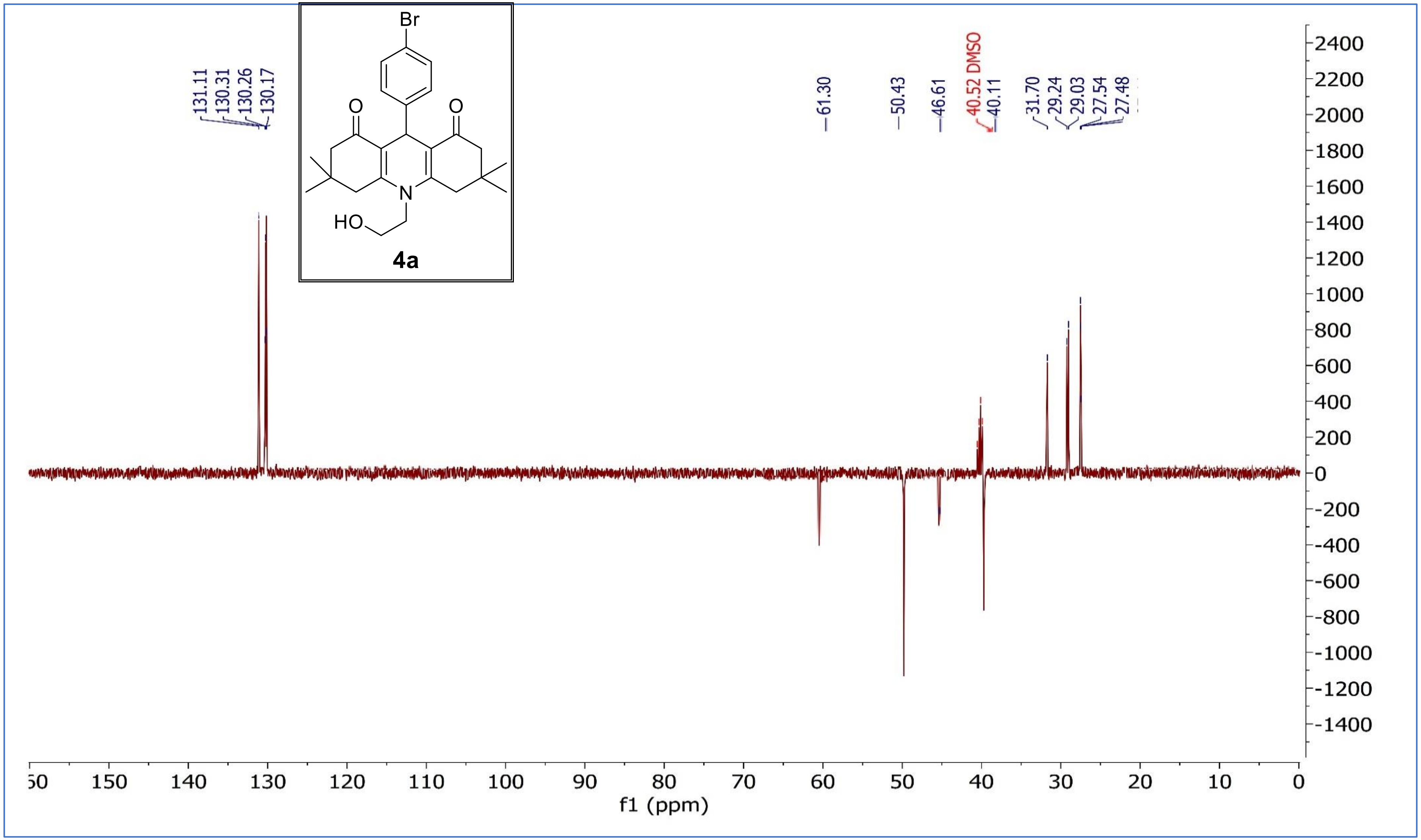

DEPT ^{13}C NMR Spectrum of compound **4a**

Sample Information

Acquired by	: System Administrator
Date Acquired	: 01/12/2021 11:06:43 ص
Sample Type	: Unknown
Sample Name	: D01
Sample ID	:
Dilution Factor	: 1
Tray#	: 1
Vial#	: 1
Injection Volume	: 10
Data File	: S_1.lcd
Method File	: Method_MS_only.lcm
Original Method File	: Method_MS_only.lcm
Report Format File	: DEFAULT.lsr
Tuning File	: default.lct
Processed by	: System Administrator
Date Processed	: 05/12/2021 09:35:50 ص

MS Chromatogram

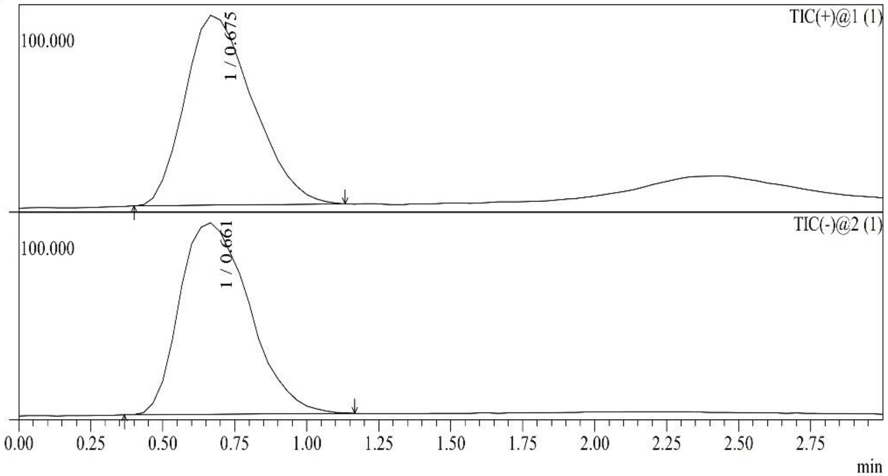

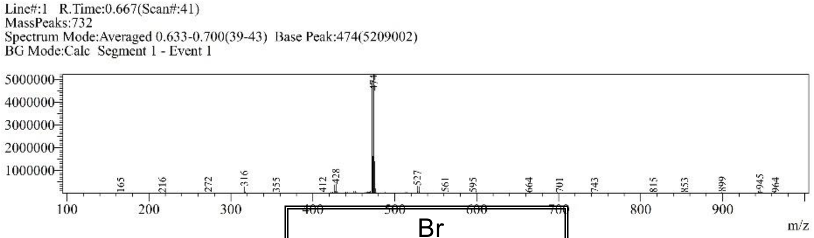

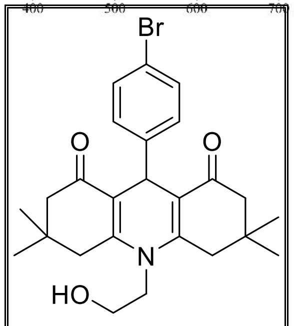

4a

MASS Peak Table ALL MC

Peak#	Ret. Time	m/z	Area	Area%	Mark	A/H	Event#
1	0.675	TIC	309874370	100.000		16.460	1-1
2	0.661	TIC	294652688	100.000		16.821	1-2
Total			604527058	200.000			

LC-MS Spectrum of compound **4a**

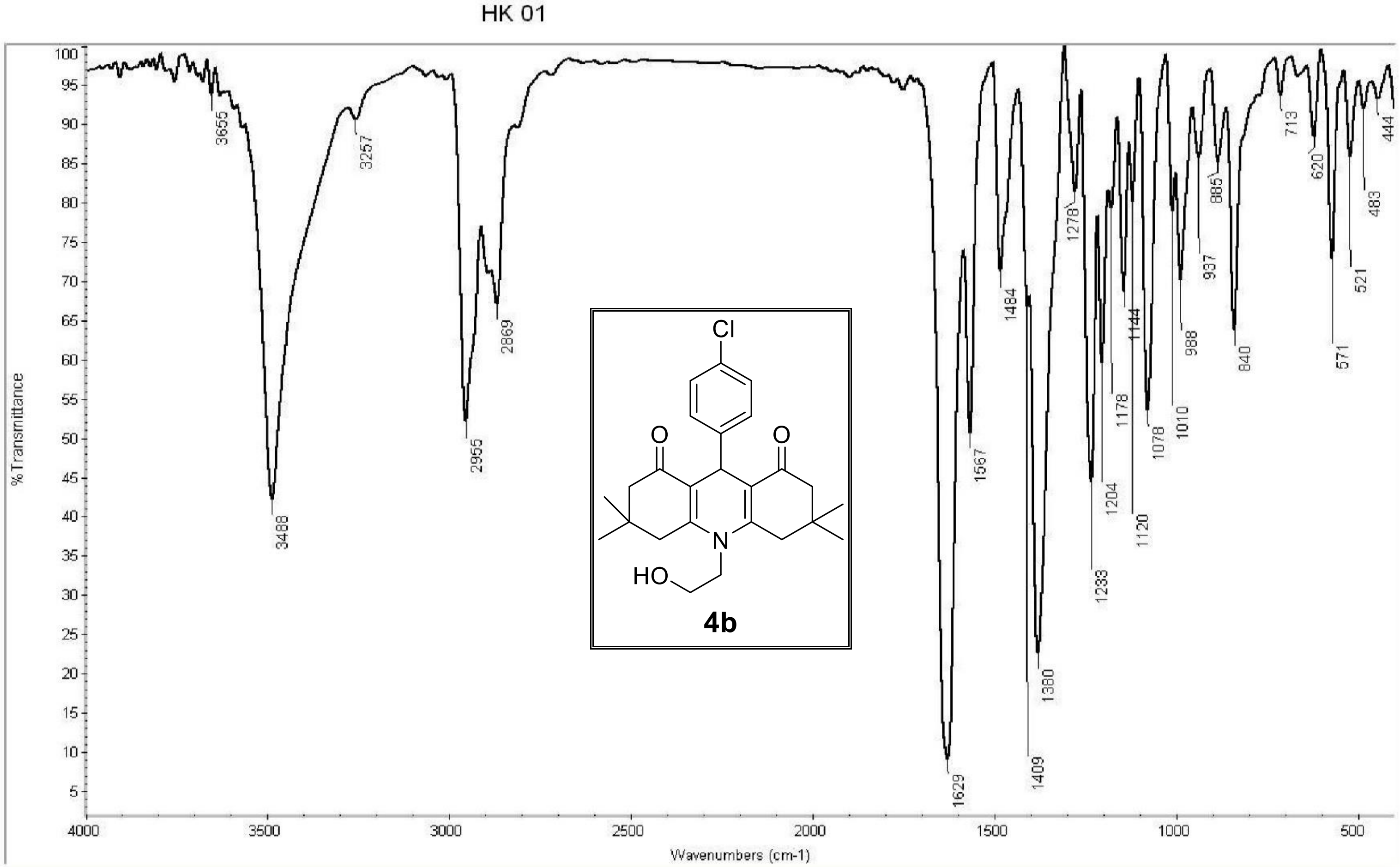

IR Spectrum of compound **4b**

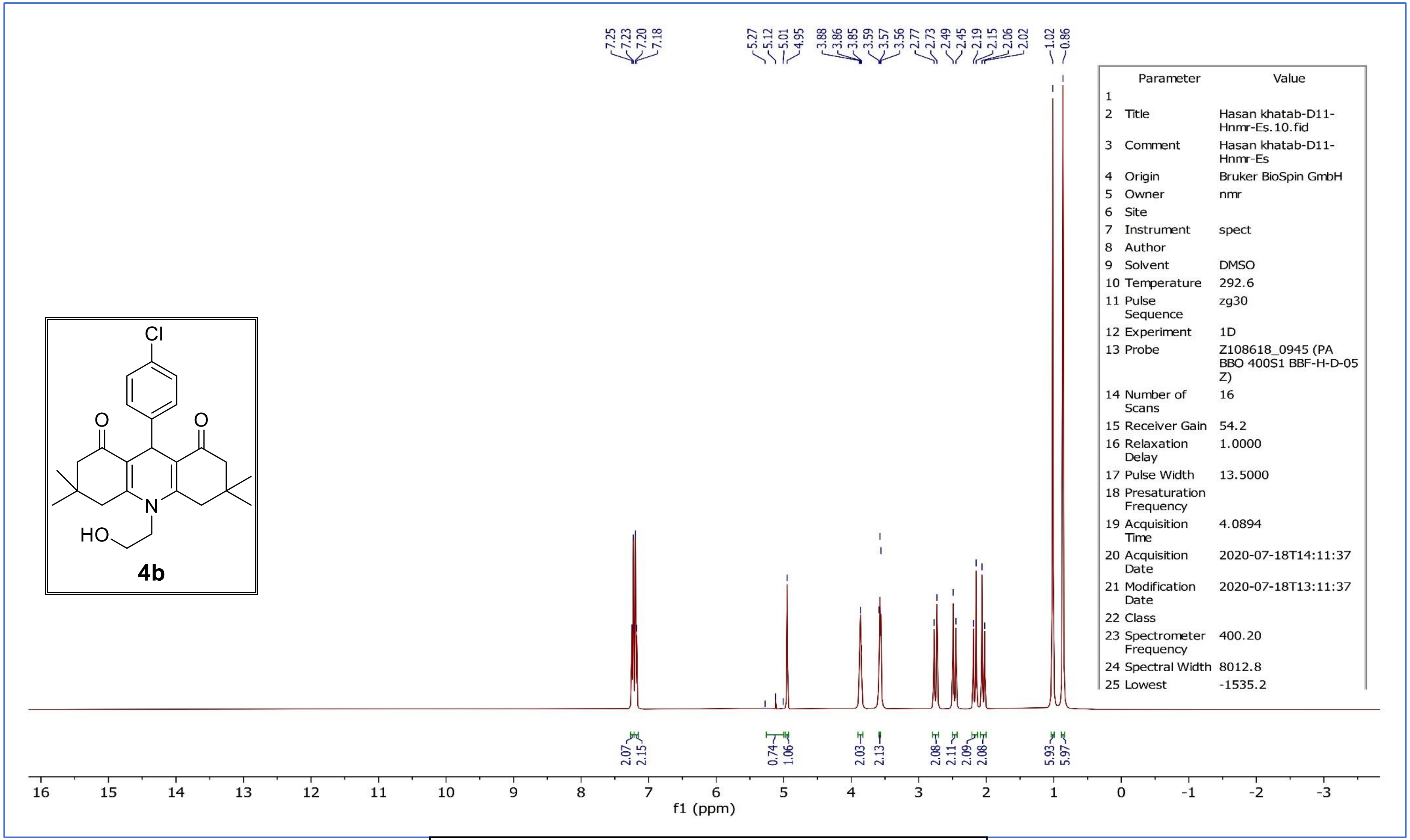

^{1}H NMR Spectrum of compound **4b**

Sample Information

Acquired by	: System Administrator
Date Acquired	: 01/12/2021 11:32:11 ص
Sample Type	: Unknown
Sample Name	: D02
Sample ID	:
Dilution Factor	: 1
Tray#	: 1
Vial#	: 2
Injection Volume	: 10
Data File	: S_2.lcd
Method File	: Method_MS_only.lcm
Original Method File	: Method_MS_only.lcm
Report Format File	: DEFAULT.lsr
Tuning File	: default.lct
Processed by	: System Administrator
Date Processed	: 05/12/2021 09:36:11 ص

MS Chromatogram

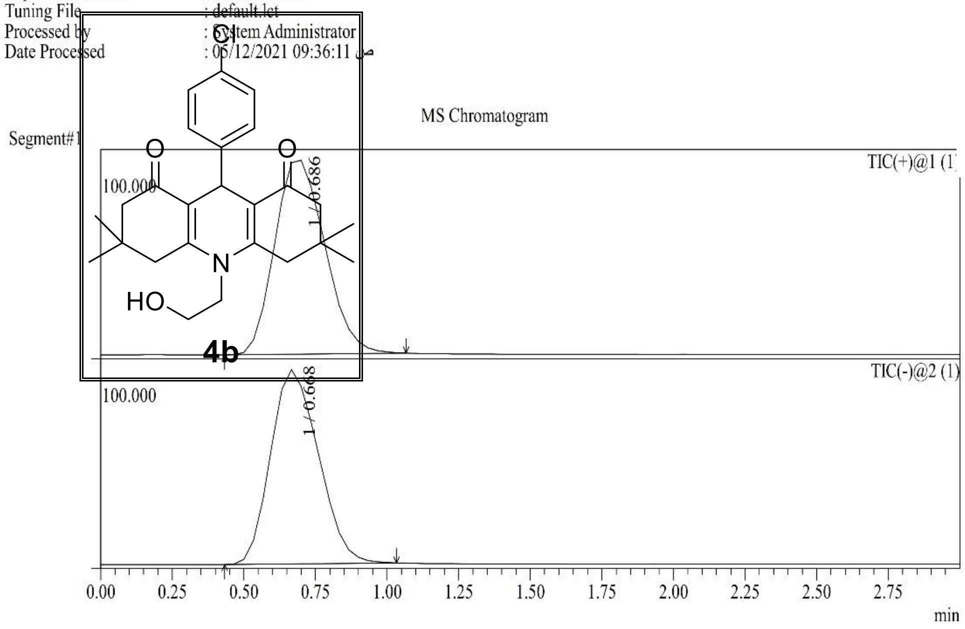

MASS Peak Table ALL MC

Peak#	Ret. Time	m/z	Area	Area%	Mark	A/H	Event#
1	0.686	TIC	335794518	100.000		12.198	1-1
2	0.668	TIC	870200438	100.000		11.581	1-2
Total			1205994956	200.000			

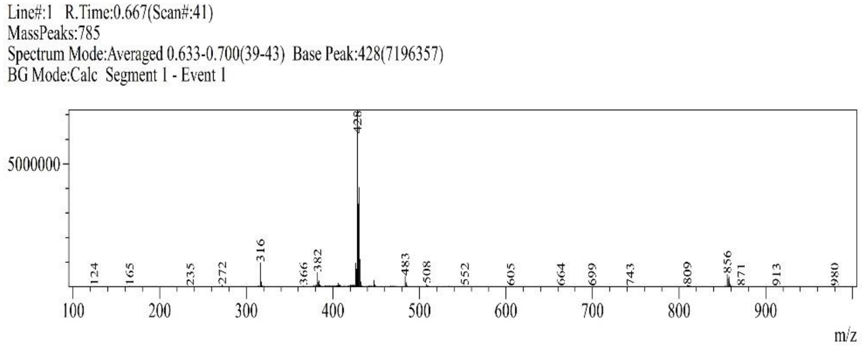

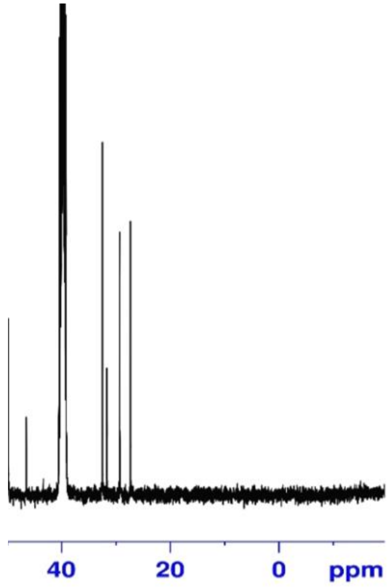

NS	1200	
DS	4	
SWH	24038.461	Hz
FIDRES	0.733596	Hz
AQ	1.3631488	sec
RG	197.77	
DW	20.800	usec
DE	6.50	usec
TE	293.3	K
D1	2.00000000	sec
D11	0.03000000	sec
TD0	1	
SFO1	100.6404331	MHz
NUC1	13C	
P1	10.00	usec
PLW1	47.00000000	W
SFO2	400.2016008	MHz
NUC2	1H	
CPDPRG[2	waltz16	
PCPD2	90.00	usec
PLW2	13.00000000	W
PLW12	0.29249999	W
PLW13	0.14713000	W

F2 - Processing parameters

SI	32768	
SF	100.6303700	MHz
WDW	EM	
SSB	0	
LB	1.00	Hz
GB	0	
PC	1.40	

LC-MS Spectrum of compound **4b**

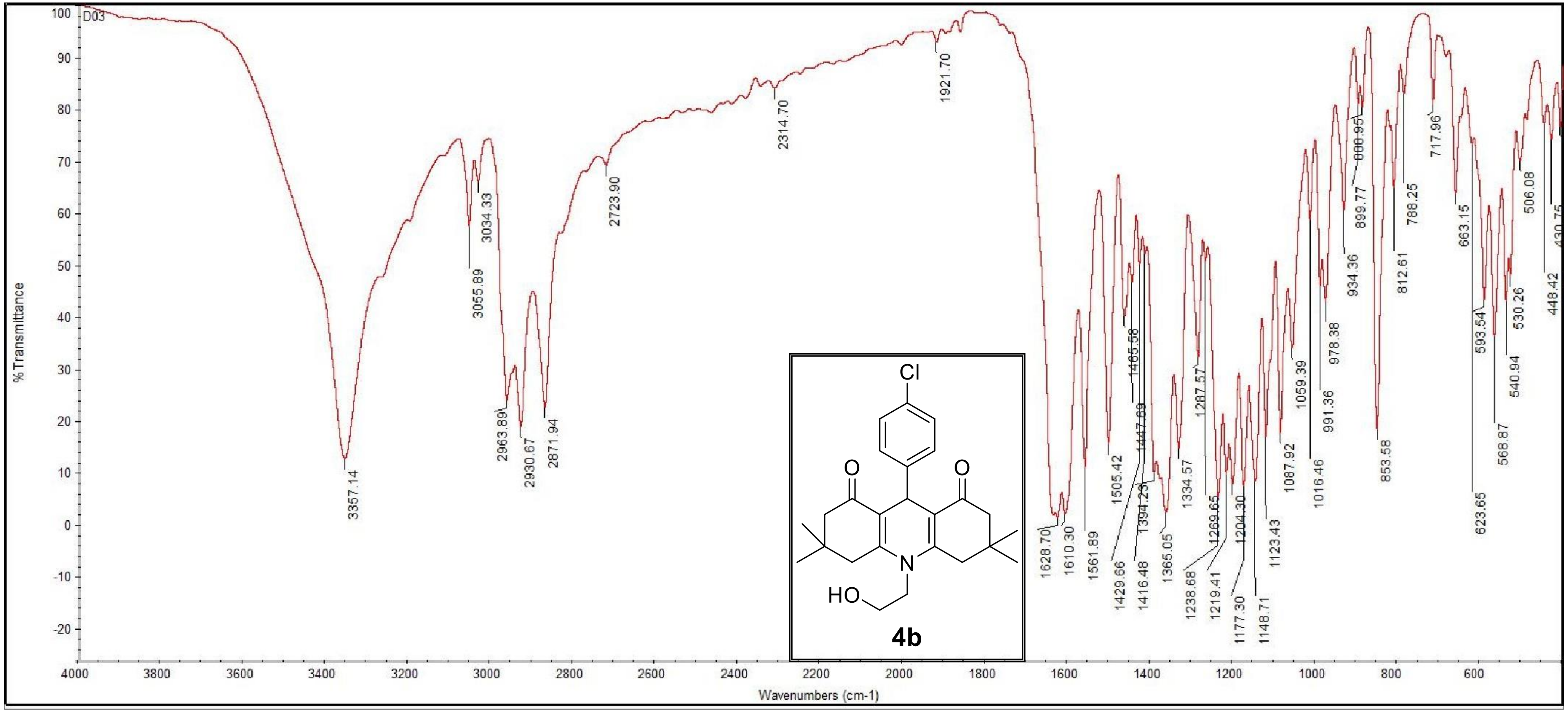

^{1}H NMR Spectrum of compound **4b**

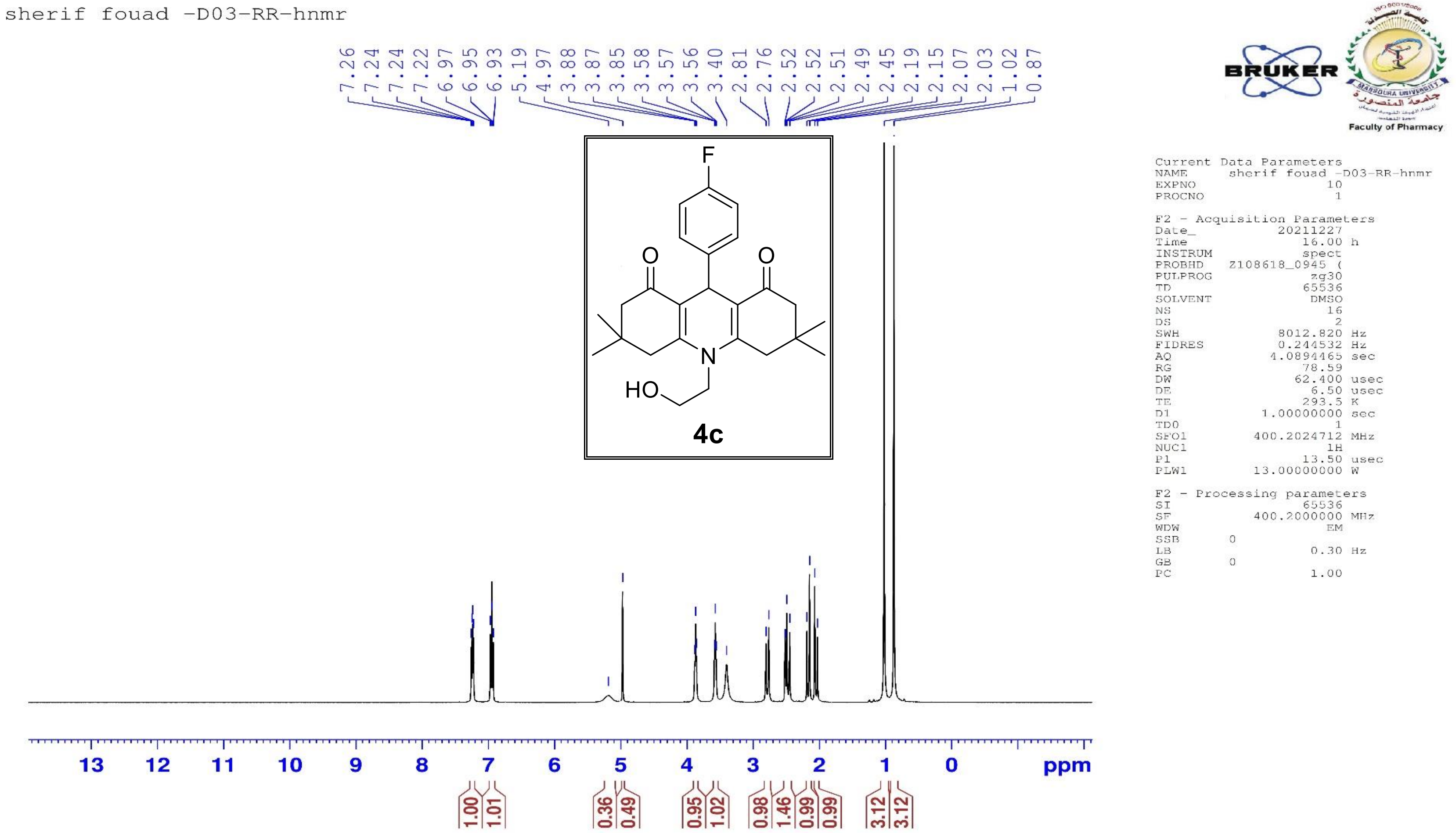
sherif fouad -D03-RR-hnmr
7.26 7.24 7.24 7.22 6.97 6.95 6.93 5.19 4.97 3.88 3.87 3.85 3.58 3.57 3.56 3.40 2.81 2.76 2.52 2.52 2.51 2.49 2.45 2.19 2.15 2.07 2.03 1.02 0.87
BRUKER
Faculty of Pharmacy
F
O
O
N
HO
4c
Current Data Parameters
NAME sherif fouad -D03-RR-hnmr
EXPNO 10
PROCNO 1
F2 - Acquisition Parameters
Date_ 20211227
Time 16.00 h
INSTRUM spect
PROBHD Z108618_0945 (
PULPROG zg30
TD 65536
SOLVENT DMSO
NS 16
DS 2
SWH 8012.820 Hz
FIDRES 0.244532 Hz
AQ 4.0894465 sec
RG 78.59
DW 62.400 usec
DE 6.50 usec
TE 293.5 K
D1 1.00000000 sec
TD0 1
SFO1 400.2024712 MHz
NUC1 1H
P1 13.50 usec
PLW1 13.00000000 W
F2 - Processing parameters
SI 65536
SF 400.2000000 MHz
WDW EM
SSB 0
LB 0.30 Hz
GB 0
PC 1.00
13 12 11 10 9 8 7 6 5 4 3 2 1 0 ppm
1.00 1.01 0.36 0.49 0.95 1.02 0.98 1.46 0.99 0.99 3.12 3.12

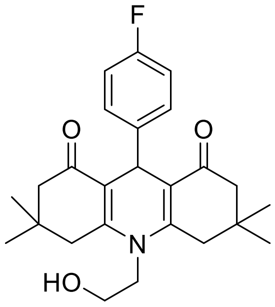

4c

Sample Information

Acquired by	: System Administrator
Date Acquired	: 01/12/2021 11:57:41 ص
Sample Type	: Unknown
Sample Name	: D03
Sample ID	:
Dilution Factor	: 1
Tray#	: 1
Vial#	: 3
Injection Volume	: 10
Data File	: S_3.lcd
Method File	: Method_MS_only.lcm
Original Method File	: Method_MS_only.lcm
Report Format File	: DEFAULT.lsr
Tuning File	: default.lct
Processed by	: System Administrator
Date Processed	: 05/12/2021 09:36:30 ص

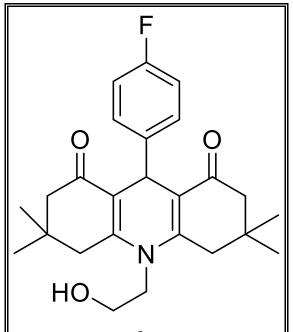

4c

MS Chromatogram

Segment#1

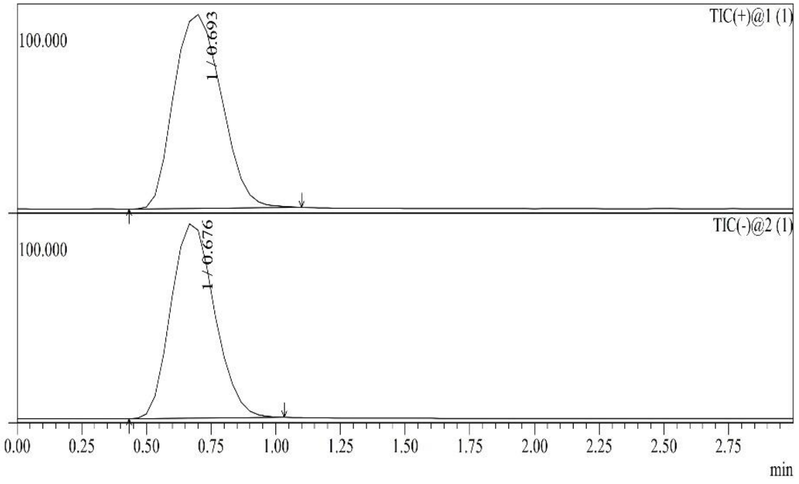

MASS Peak Table ALL MC

Peak#	Ret. Time	m/z	Area	Area%	Mark	A/H	Event#
1	0.693	TIC	292764122	100.000		12.815	1-1
2	0.676	TIC	703420534	100.000		11.750	1-2
Total			996184656	200.000			

Line#:1 R.Time:0.667(Scan#:41)
MassPeaks:833
Spectrum Mode:Averaged 0.633-0.700(39-43) Base Peak:412(7394241)
BG Mode:Calc Segment 1 - Event 1

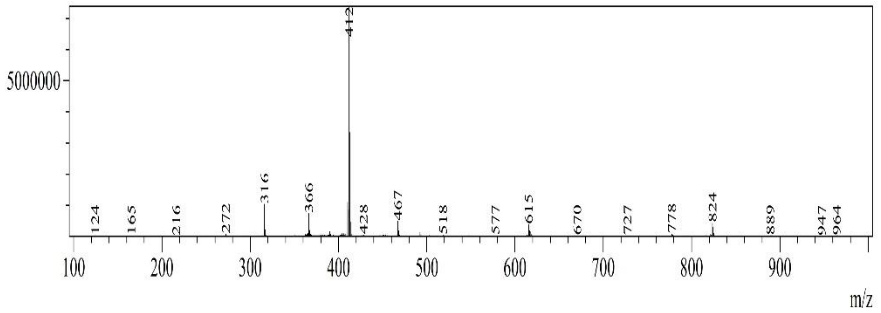

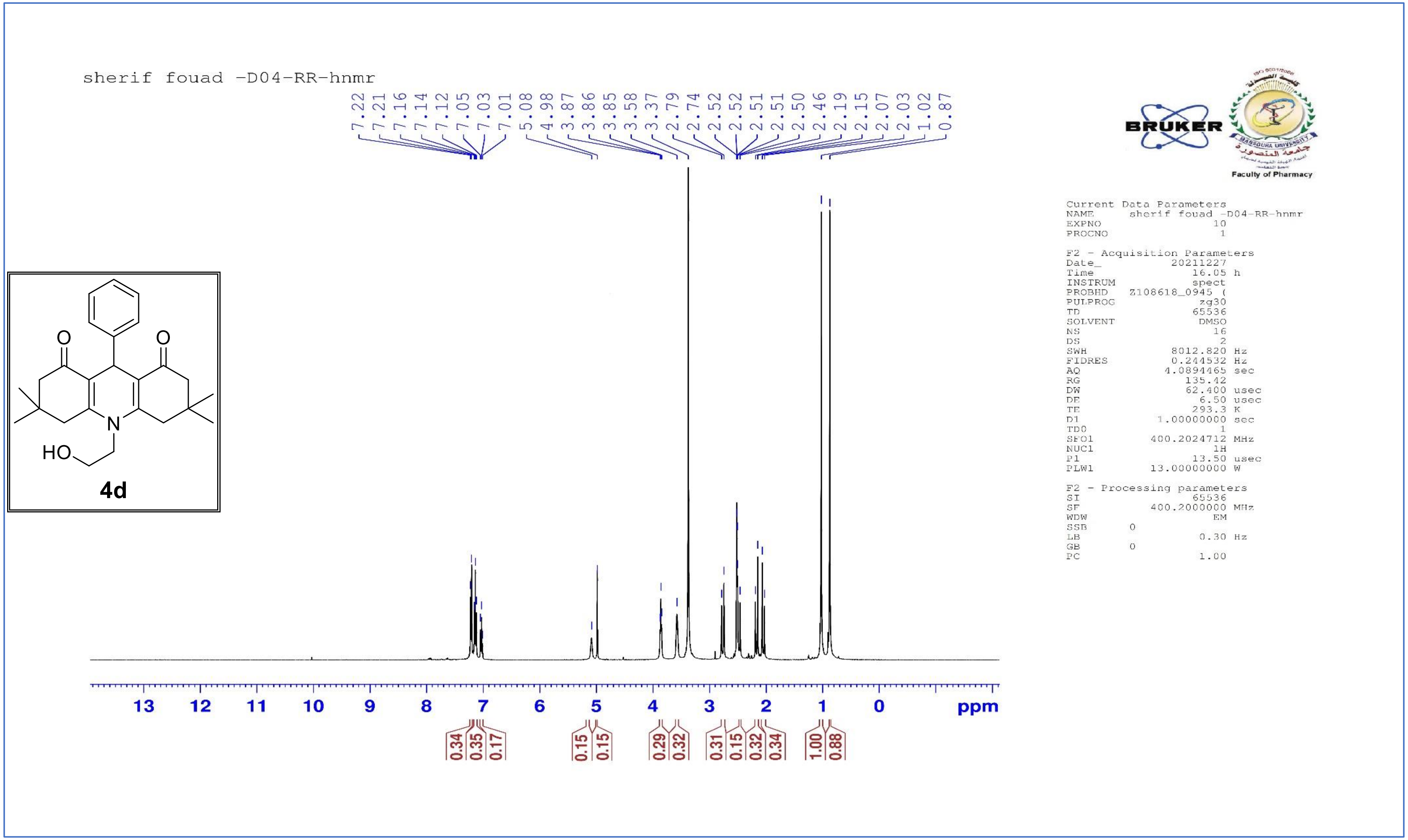

^{1}H NMR Spectrum of compound **4d**

Sample Information

Acquired by	: System Administrator
Date Acquired	: 01/12/2021 12:23:10 م
Sample Type	: Unknown
Sample Name	: D04
Sample ID	:
Dilution Factor	: 1
Tray#	: 1
Vial#	: 4
Injection Volume	: 10
Data File	: S_4.lcd
Method File	: Method_MS_only.lcm
Original Method File	: Method_MS_only.lcm
Report Format File	: DEFAULT.lsr
Tuning File	: default.lct
Processed by	: System Administrator
Date Processed	: 05/12/2021 09:36:48 ص

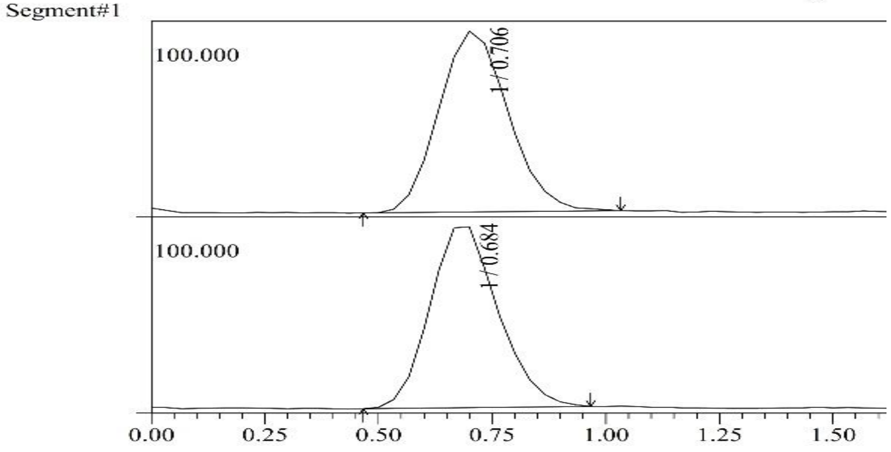

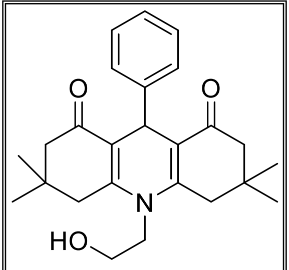

4d

MASS Peak Table ALL M

Peak#	Ret. Time	m/z	Area	Area%	Mark
1	0.706	TIC	17605141	100.000	
2	0.684	TIC	31134830	100.000	
Total			48739971	200.000	

Line#:1 R.Time:0.700(Scan#:43)
MassPeaks:635
Spectrum Mode:Averaged 0.667-0.733(41-45) Base Peak:394(701808)
BG Mode:Calc Segment 1 - Event 1

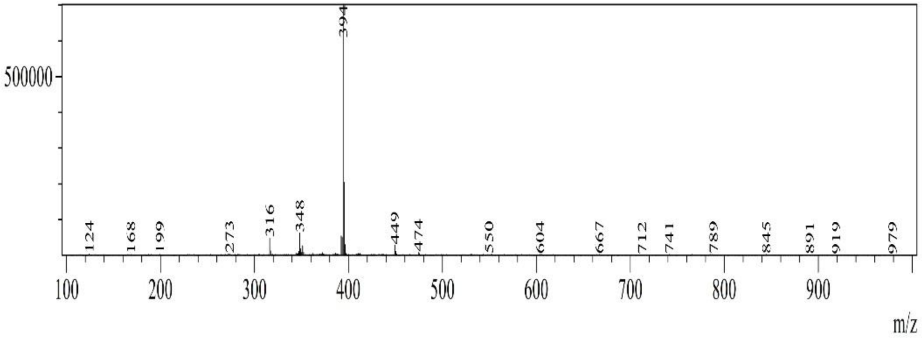

LC-MS Spectrum of compound **4d**

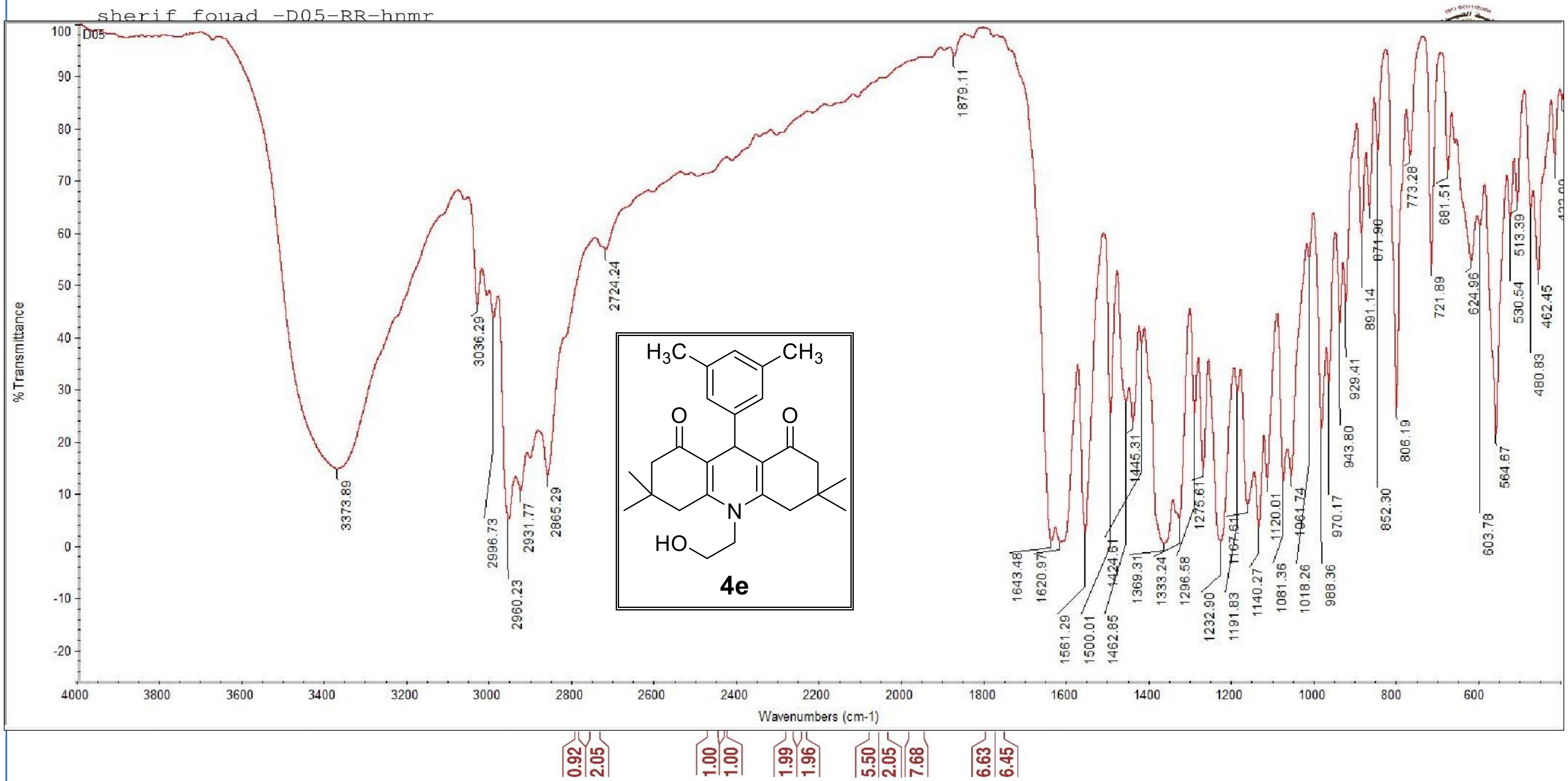

IR Spectrum of compound **4e**

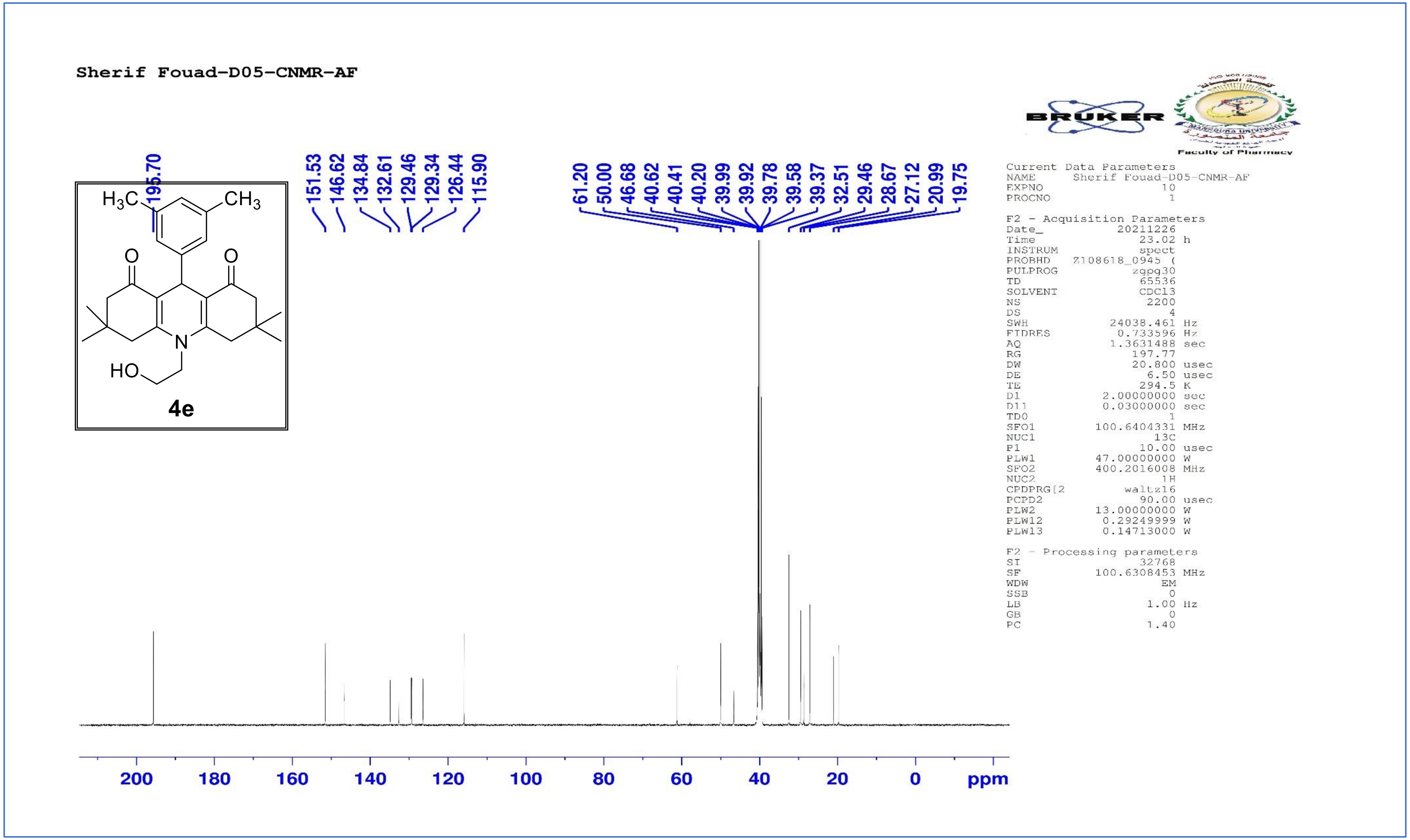

^{13}C NMR Spectrum of compound **4e**

Sample Information

Acquired by	: System Administrator
Date Acquired	: 01/12/2021 12:48:39 م
Sample Type	: Unknown
Sample Name	: D05
Sample ID	:
Dilution Factor	: 1
Tray#	: 1
Vial#	: 5
Injection Volume	: 10
Data File	: S_5.lcd
Method File	: Method_MS_only.lcm
Original Method File	: Method_MS_only.lcm
Report Format File	: DEFAULT.lsr
Tuning File	: default.lct
Processed by	: System Administrator
Date Processed	: 05/12/2021 09:37:10 ص

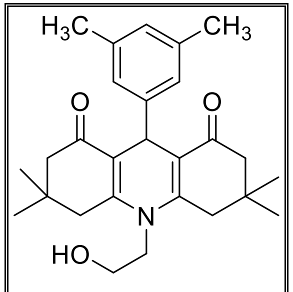

4e

MS Chromatogram

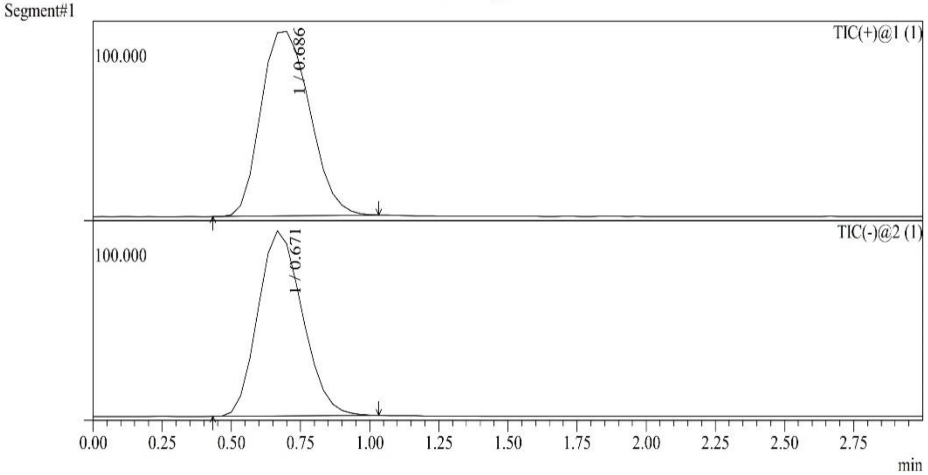

Line#:1 R.Time:0.667(Scan#:41)
MassPeaks:765
Spectrum Mode:Averaged 0.633-0.700(39-43) Base Peak:422(6593788)
BG Mode:Calc Segment 1 - Event 1

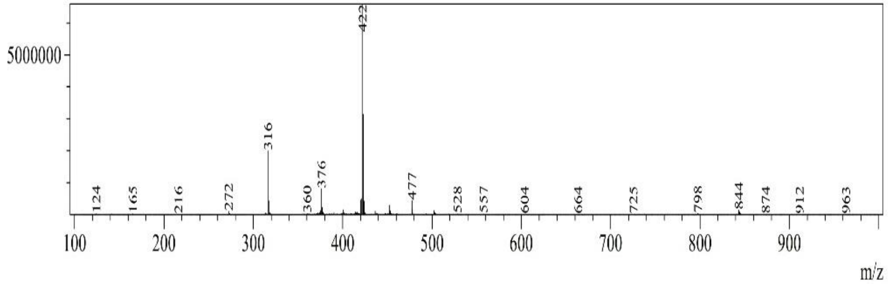

MASS Peak Table ALL MC

Peak#	Ret. Time	m/z	Area	Area%	Mark	A/H	Event#
1	0.686	TIC	241554508	100.000		12.300	1-1
2	0.671	TIC	532422788	100.000		11.185	1-2
Total			773977296	200.000			

LC-MS Spectrum of compound **4e**

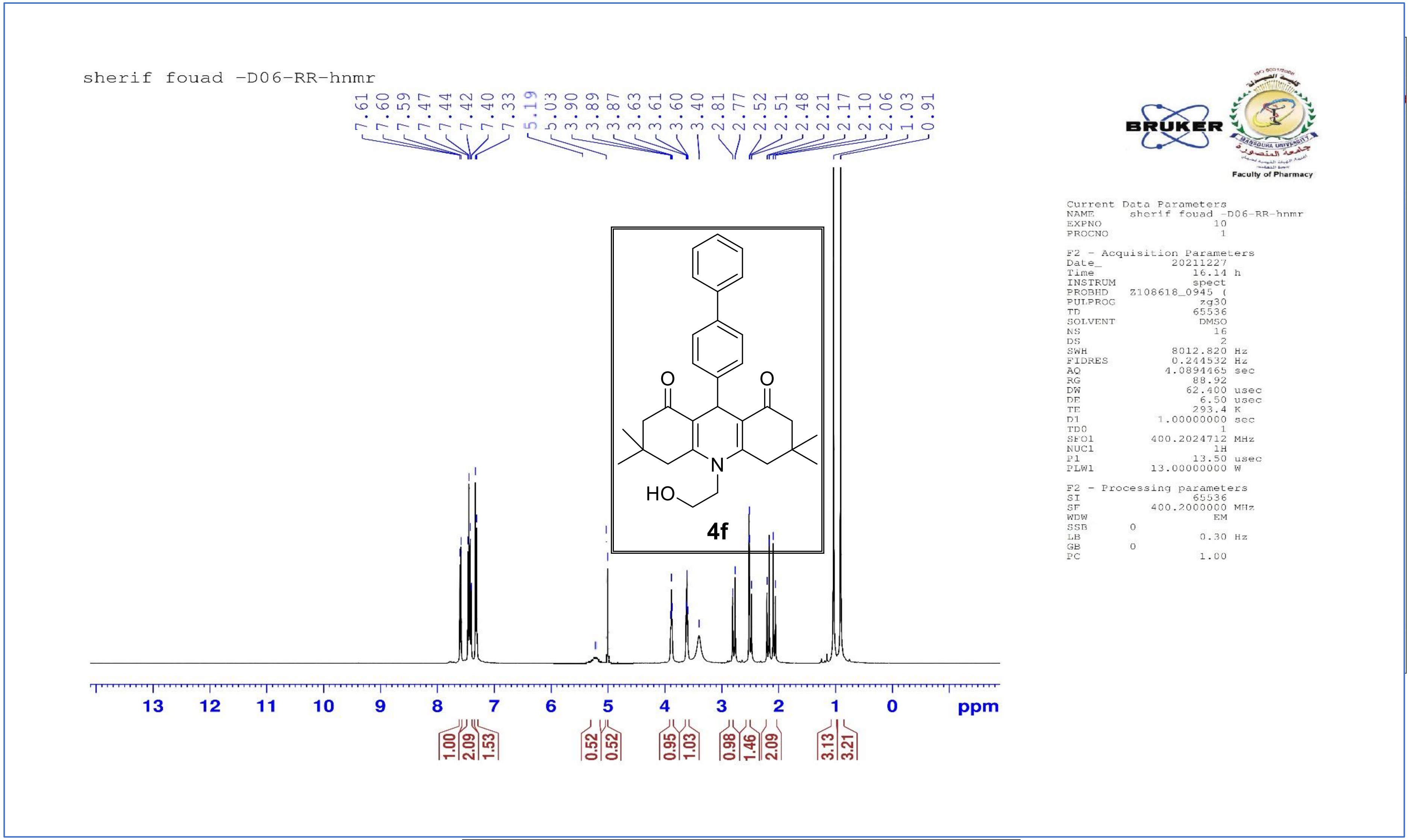

^{1}H NMR Spectrum of compound **4f**

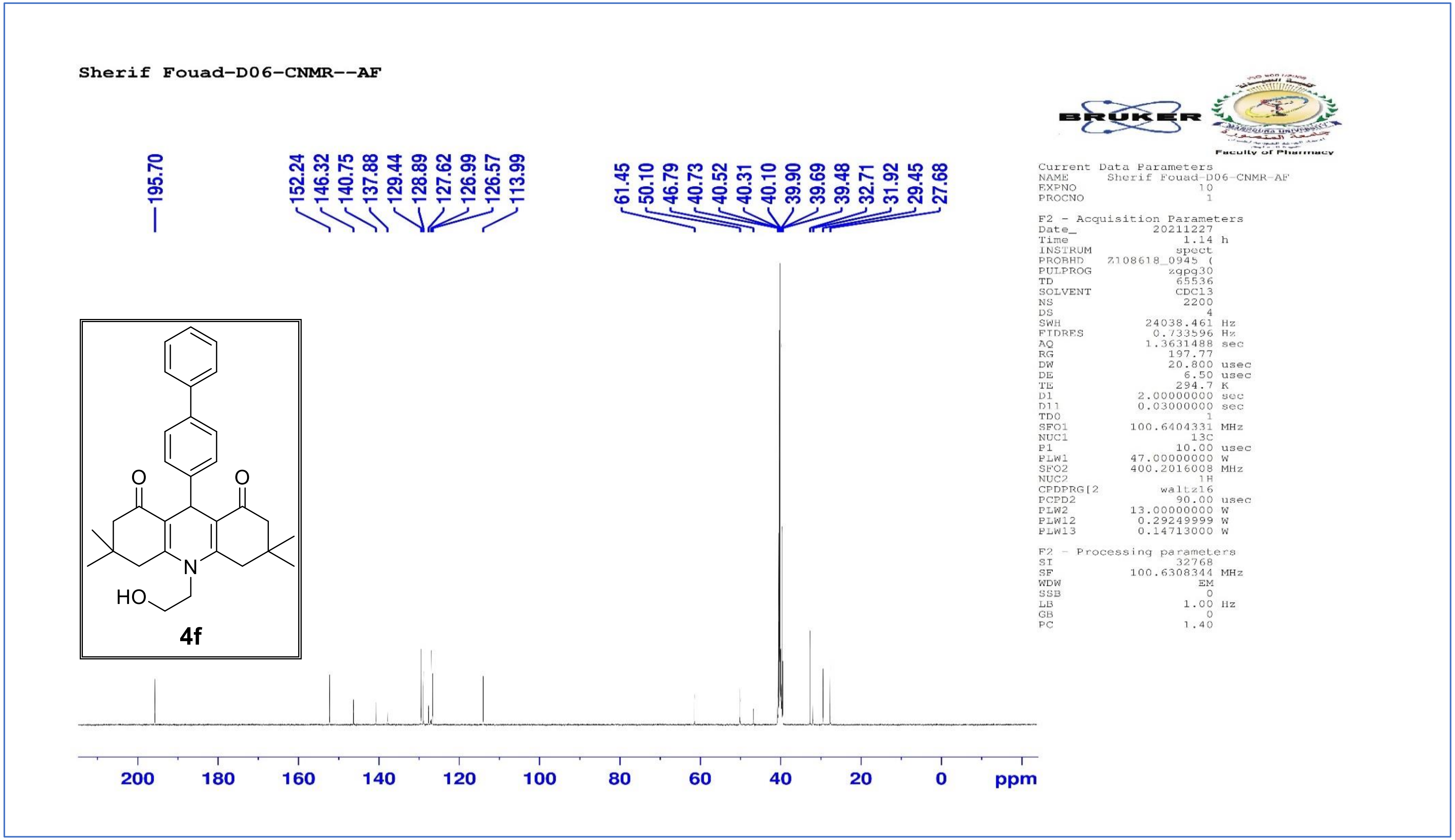

^{13}C NMR Spectrum of compound **4f**

Sample Information

Acquired by	: System Administrator
Date Acquired	: 01/12/2021 01:06:33 م
Sample Type	: Unknown
Sample Name	: D06
Sample ID	:
Dilution Factor	: 1
Tray#	: 1
Vial#	: 6
Injection Volume	: 10
Data File	: S_6.lcd
Method File	: Method_MS_only.lcm
Original Method File	: Method_MS_only.lcm
Report Format File	: DEFAULT.lsr
Tuning File	: default.lct
Processed by	: System Administrator
Date Processed	: 05/12/2021 09:37:43 ص

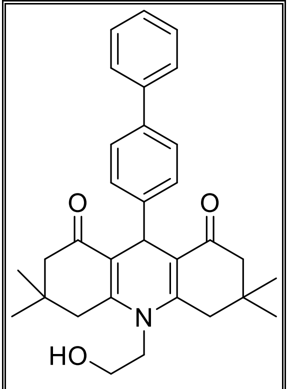

4f

MS Chromatogram

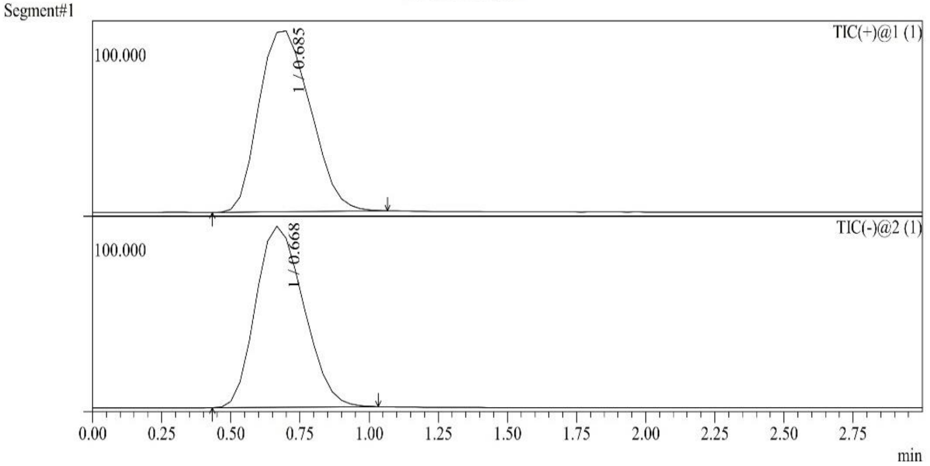

Line#:1 R.Time:0.667(Scan#:41)
MassPeaks:805
Spectrum Mode:Averaged 0.633-0.700(39-43) Base Peak:470(7699472)
BG Mode:Calc Segment 1 - Event 1

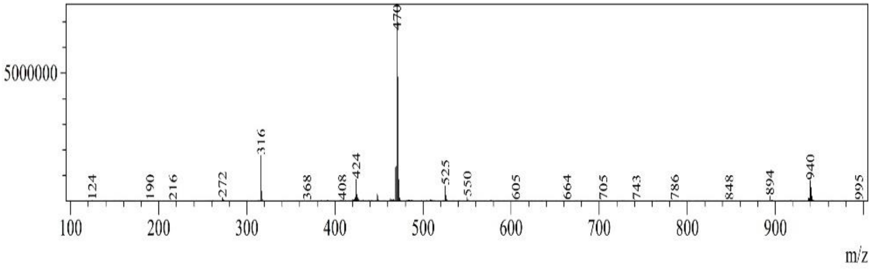

MASS Peak Table ALL MC

Peak#	Ret. Time	m/z	Area	Area%	Mark	A/H	Event#
1	0.685	TIC	352135962	100.000		12.977	1-1
2	0.668	TIC	826085904	100.000		12.088	1-2
Total			1178221866	200.000			

LC-MS Spectrum of compound **4f**

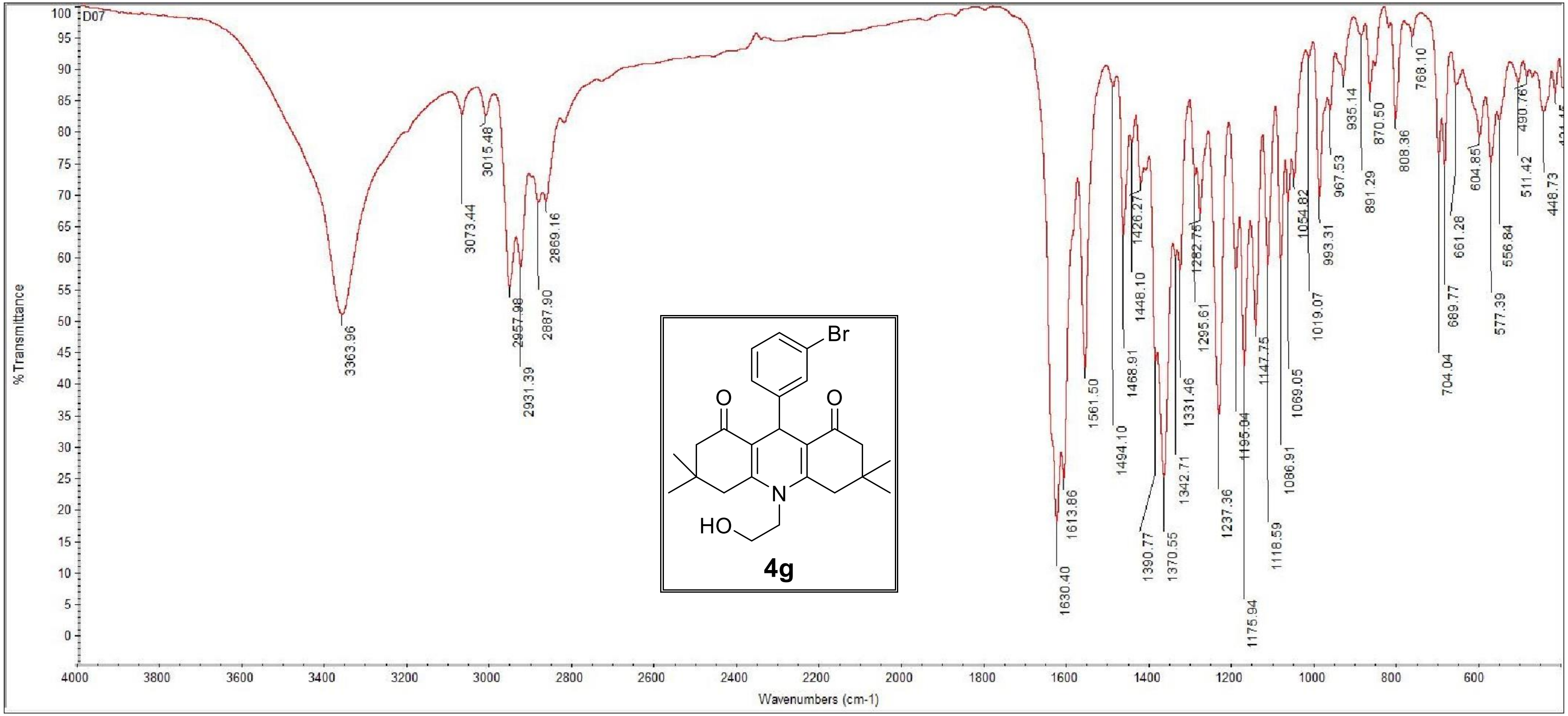
D07
% Transmittance
Wavenumbers (cm-1)
3363.96
3073.44
3015.48
2957.98
2931.39
2887.90
2869.16
1630.40
1613.86
1561.50
1494.10
1468.91
1448.10
1426.27
1390.77
1370.55
1342.71
1331.46
1295.61
1282.75
1237.36
1195.04
1175.94
1147.75
1118.59
1086.91
1069.05
1054.82
1019.07
993.31
967.53
935.14
891.29
870.50
808.36
768.10
704.04
689.77
661.28
604.85
577.39
556.84
511.42
490.76
448.73
Br
O
O
N
HO
4g

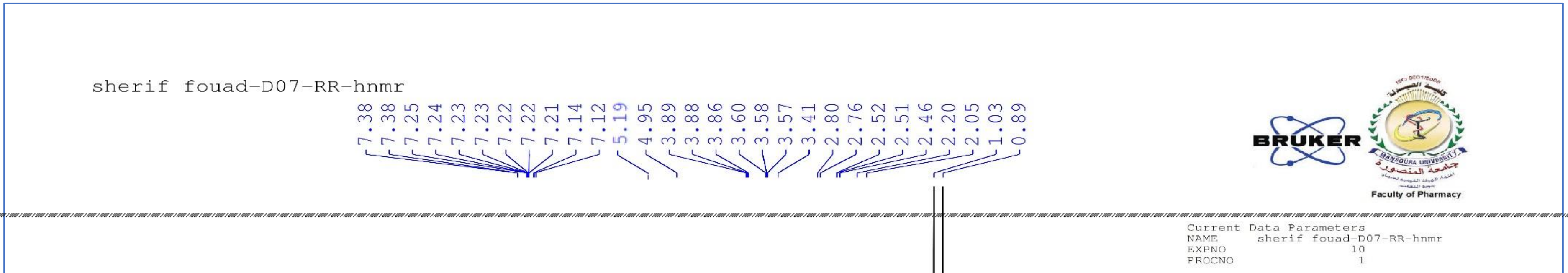
sherif fouad-D07-RR-hnmr
7.38 7.38 7.25 7.24 7.23 7.23 7.22 7.22 7.21 7.14 7.12 5.19 4.95 3.89 3.88 3.86 3.60 3.58 3.57 3.41 2.80 2.76 2.52 2.51 2.46 2.20 2.05 1.03 0.89
BRUKER
Faculty of Pharmacy
Current Data Parameters
NAME sherif fouad-D07-RR-hnmr
EXPNO 10
PROCNO 1

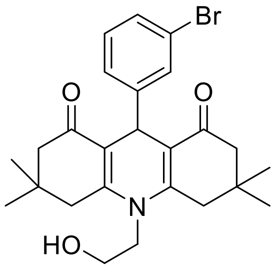
Br
O
O
N
HO

4g

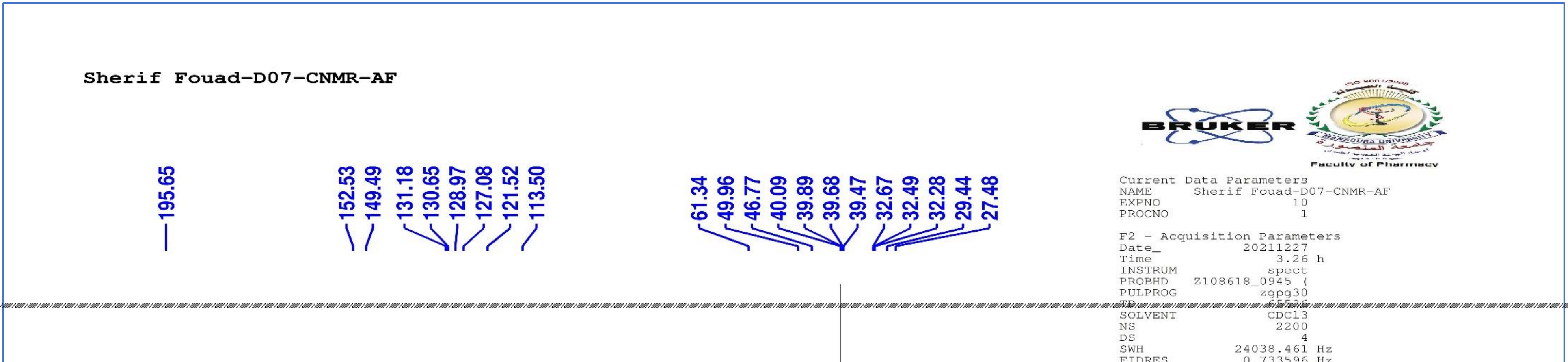
Sherif Fouad-D07-CNMR-AF
195.65
152.53
149.49
131.18
130.65
128.97
127.08
121.52
113.50
61.34
49.96
46.77
40.09
39.89
39.68
39.47
32.67
32.49
32.28
29.44
27.48
BRUKER
Faculty of Pharmacy
Current Data Parameters
NAME Sherif Fouad-D07-CNMR-AF
EXPNO 10
PROCNO 1
F2 - Acquisition Parameters
Date_ 20211227
Time 3.26 h
INSTRUM spect
PROBHD Z108618_0945 (
PULPROG zgpg30
SOLVENT CDCl3
NS 2200
DS 4
SWH 24038.461 Hz

Sample Information

Acquired by	: System Administrator
Date Acquired	: 01/12/2021 01:12:04 م
Sample Type	: Unknown
Sample Name	: D07
Sample ID	:
Dilution Factor	: 1
Tray#	: 1
Vial#	: 7
Injection Volume	: 10
Data File	: S_7.lcd
Method File	: Method_MS_only.lcm
Original Method File	: Method_MS_only.lcm
Report Format File	: DEFAULT.lsr
Tuning File	: default.lct
Processed by	: System Administrator
Date Processed	: 05/12/2021 09:38:14 ص

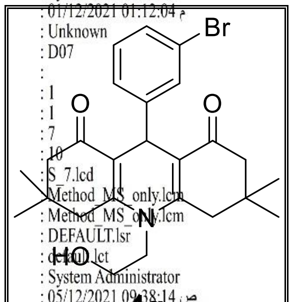

4g

MS Chromatogram

Segment#1

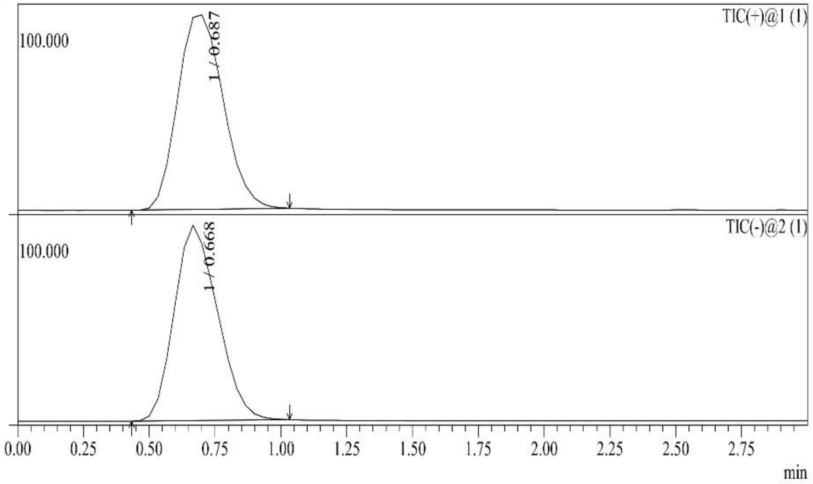

MASS Peak Table ALL MC

Peak#	Ret. Time	m/z	Area	Area%	Mark	A/H	Event#
1	0.687	TIC	312177216	100.000		12.064	1-1
2	0.668	TIC	784001042	100.000		11.398	1-2
Total			1096178258	200.000			

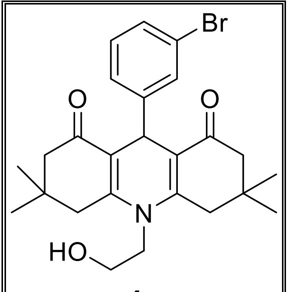

4g

Line#:1 R.Time:0.667(Scan#:41)
MassPeaks:801
Spectrum Mode:Averaged 0.633-0.700(39-43) Base Peak:472(6141759)
BG Mode:Calc Segment 1 - Event 1

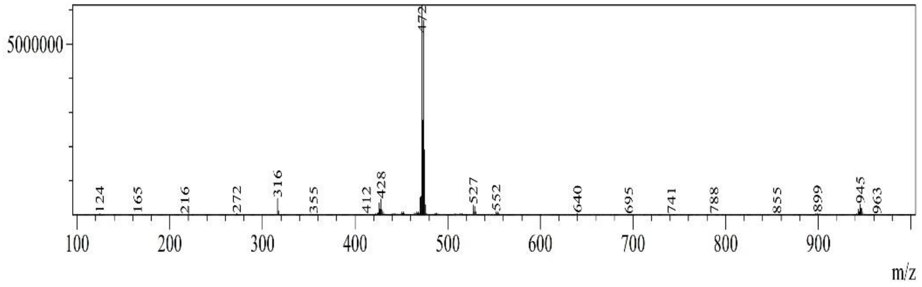

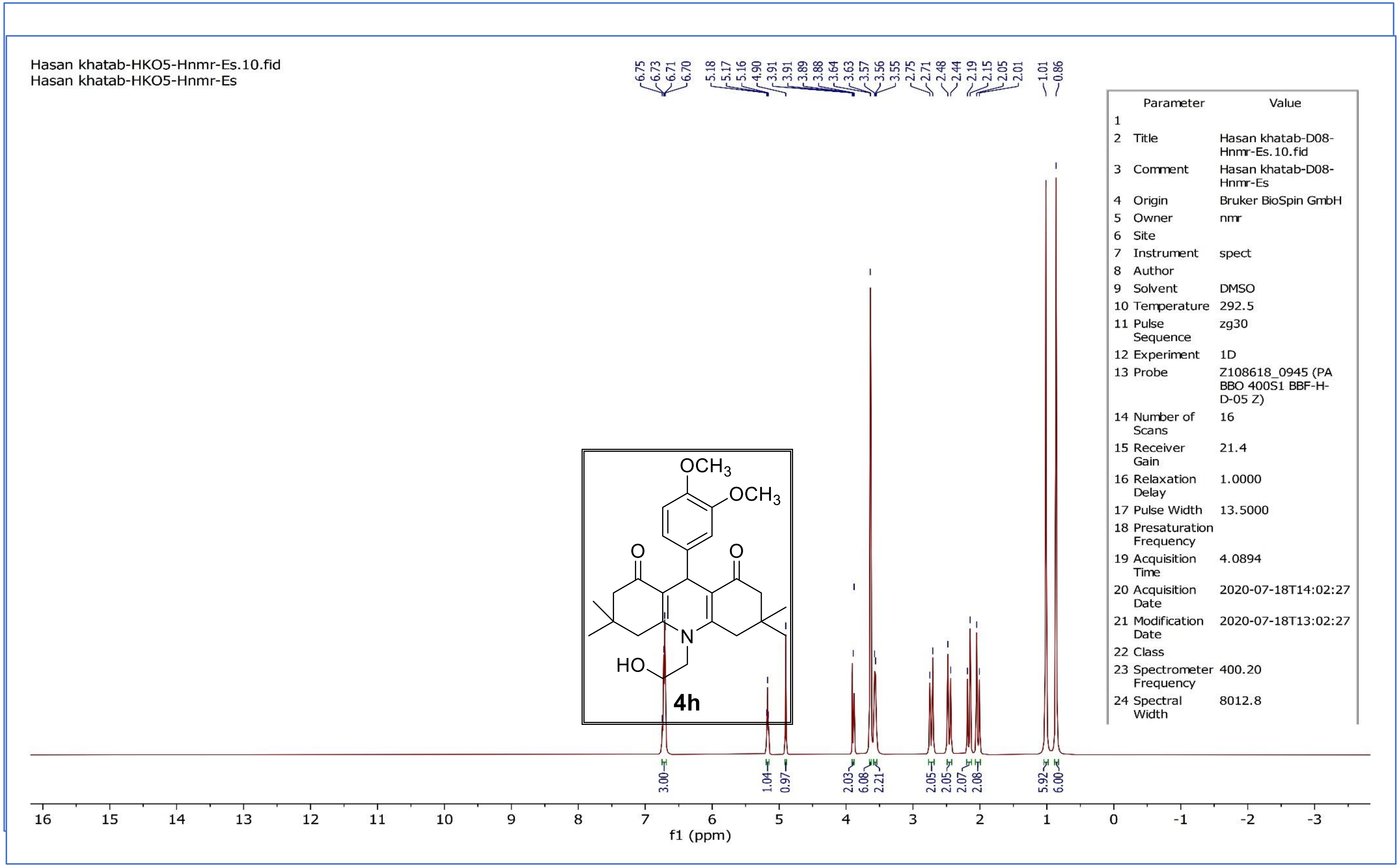
Hasan khatab-HKO5-Hnmr-Es.10.fid
Hasan khatab-HKO5-Hnmr-Es
6.75 6.73 6.71 6.70 5.18 5.17 5.16 4.90 3.91 3.91 3.89 3.88 3.64 3.63 3.57 3.56 3.55 2.75 2.71 2.48 2.44 2.19 2.15 2.05 2.01 1.01 0.86
Parameter Value
1
2 Title Hasan khatab-D08-Hnmr-Es.10.fid
3 Comment Hasan khatab-D08-Hnmr-Es
4 Origin Bruker BioSpin GmbH
5 Owner nmr
6 Site
7 Instrument spect
8 Author
9 Solvent DMSO
10 Temperature 292.5
11 Pulse Sequence zg30
12 Experiment 1D
13 Probe Z108618_0945 (PA BBO 400S1 BBF-H-D-05 Z)
14 Number of Scans 16
15 Receiver Gain 21.4
16 Relaxation Delay 1.0000
17 Pulse Width 13.5000
18 Presaturation Frequency
19 Acquisition Time 4.0894
20 Acquisition Date 2020-07-18T14:02:27
21 Modification Date 2020-07-18T13:02:27
22 Class
23 Spectrometer Frequency 400.20
24 Spectral Width 8012.8
OCH3
OCH3
O
O
N
HO
4h
3.00 1.04 0.97 2.03 6.08 2.21 2.05 2.05 2.07 2.08 5.92 6.00
16 15 14 13 12 11 10 9 8 7 6 5 4 3 2 1 0 -1 -2 -3
f1 (ppm)

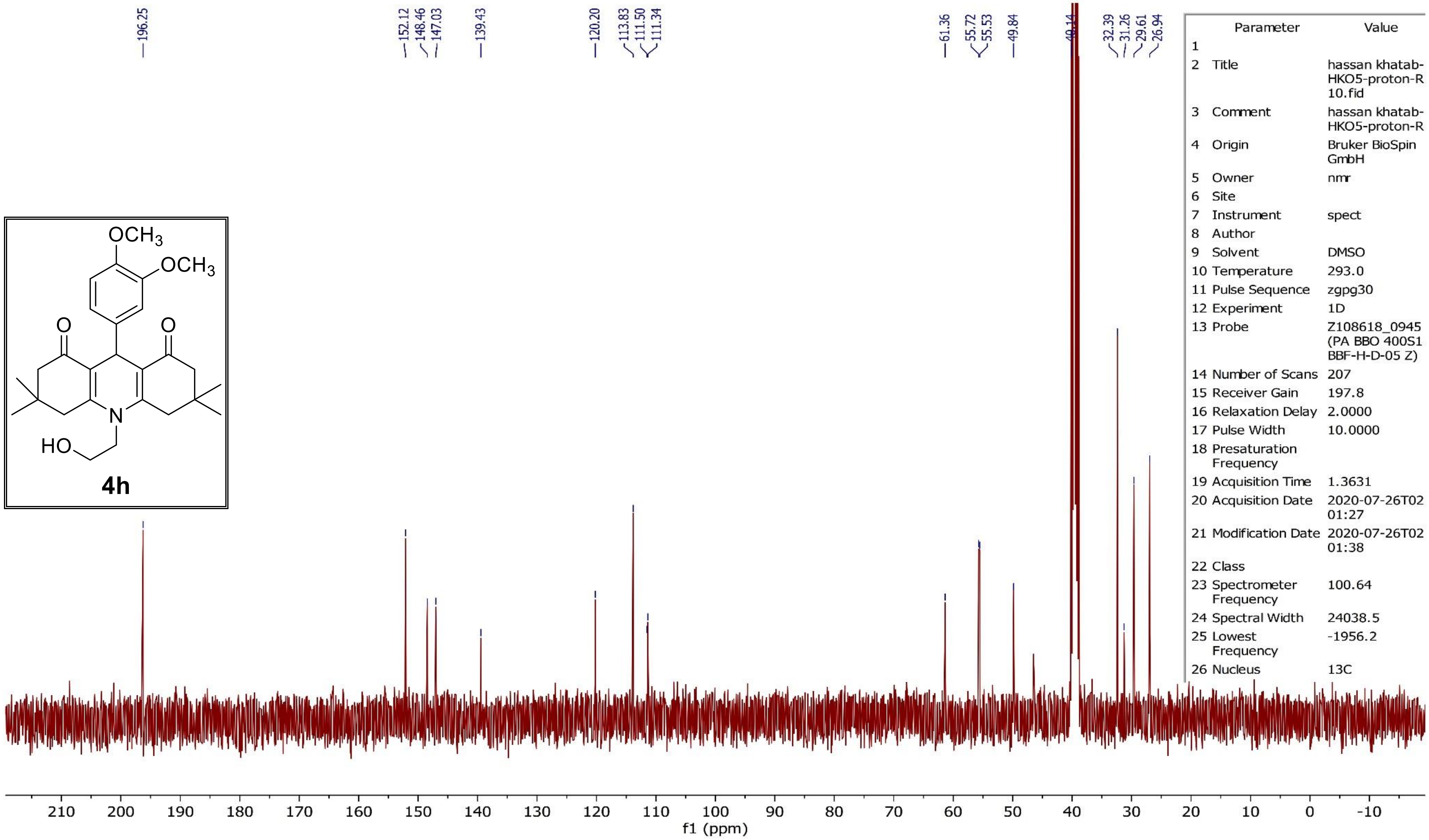

Parameter		Value
1		
2	Title	hassan khatab-HKO5-proton-R 10.fid
3	Comment	hassan khatab-HKO5-proton-R
4	Origin	Bruker BioSpin GmbH
5	Owner	nmr
6	Site	
7	Instrument	spect
8	Author	
9	Solvent	DMSO
10	Temperature	293.0
11	Pulse Sequence	zgpg30
12	Experiment	1D
13	Probe	Z108618_0945 (PA BBO 400S1 BBF-H-D-05 Z)
14	Number of Scans	207
15	Receiver Gain	197.8
16	Relaxation Delay	2.0000
17	Pulse Width	10.0000
18	Presaturation Frequency	
19	Acquisition Time	1.3631
20	Acquisition Date	2020-07-26T02 01:27
21	Modification Date	2020-07-26T02 01:38
22	Class	
23	Spectrometer Frequency	100.64
24	Spectral Width	24038.5
25	Lowest Frequency	-1956.2
26	Nucleus	13C

Sample Information

Acquired by	: System Administrator
Date Acquired	: 01/12/2021 01:17:35 م
Sample Type	: Unknown
Sample Name	: D08
Sample ID	:
Dilution Factor	: 1
Tray#	: 1
Vial#	: 8
Injection Volume	: 10
Data File	: [illegible]_8.lcd
Method File	: Method_MS_only.lcm
Original Method File	: Method_MS_only.lcm
Report Format File	: DEFAULT.lsr
Tuning File	: default.lct
Processed by	: System Administrator
Date Processed	: 05/12/2021 09:38:39 ص

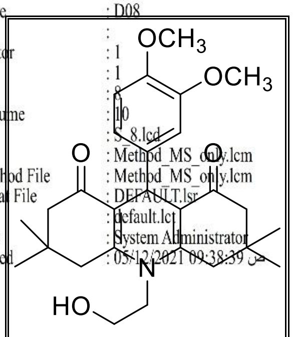

4h

MS Chromatogram

Segment#1

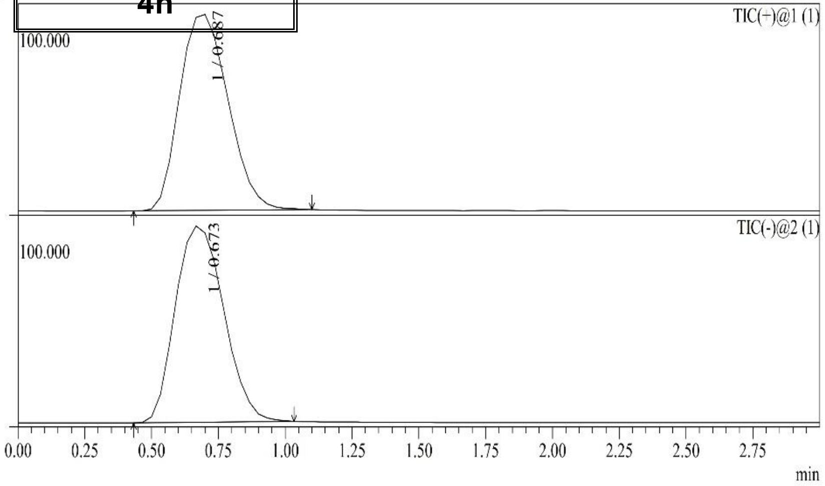

MASS Peak Table ALL MC

Peak#	Ret. Time	m/z	Area	Area%	Mark	A/H	Event#
1	0.687	TIC	351210358	100.000		12.637	1-1
2	0.673	TIC	789936400	100.000		12.630	1-2
Total			1141146758	200.000			

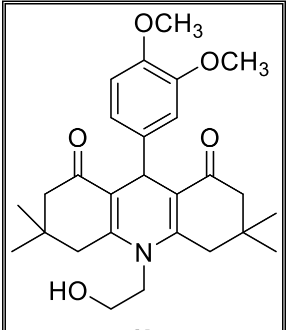

4h

Line#:1 R.Time:0.667(Scan#:41)
MassPeaks:843
Spectrum Mode:Averaged 0.633-0.700(39-43) Base Peak:454(6440031)
BG Mode:Calc Segment 1 - Event 1

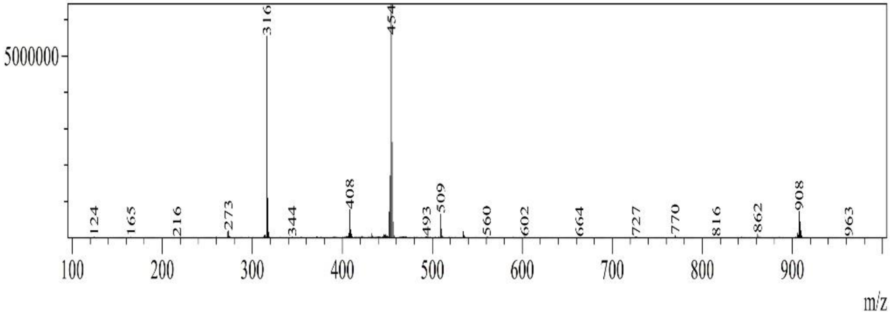

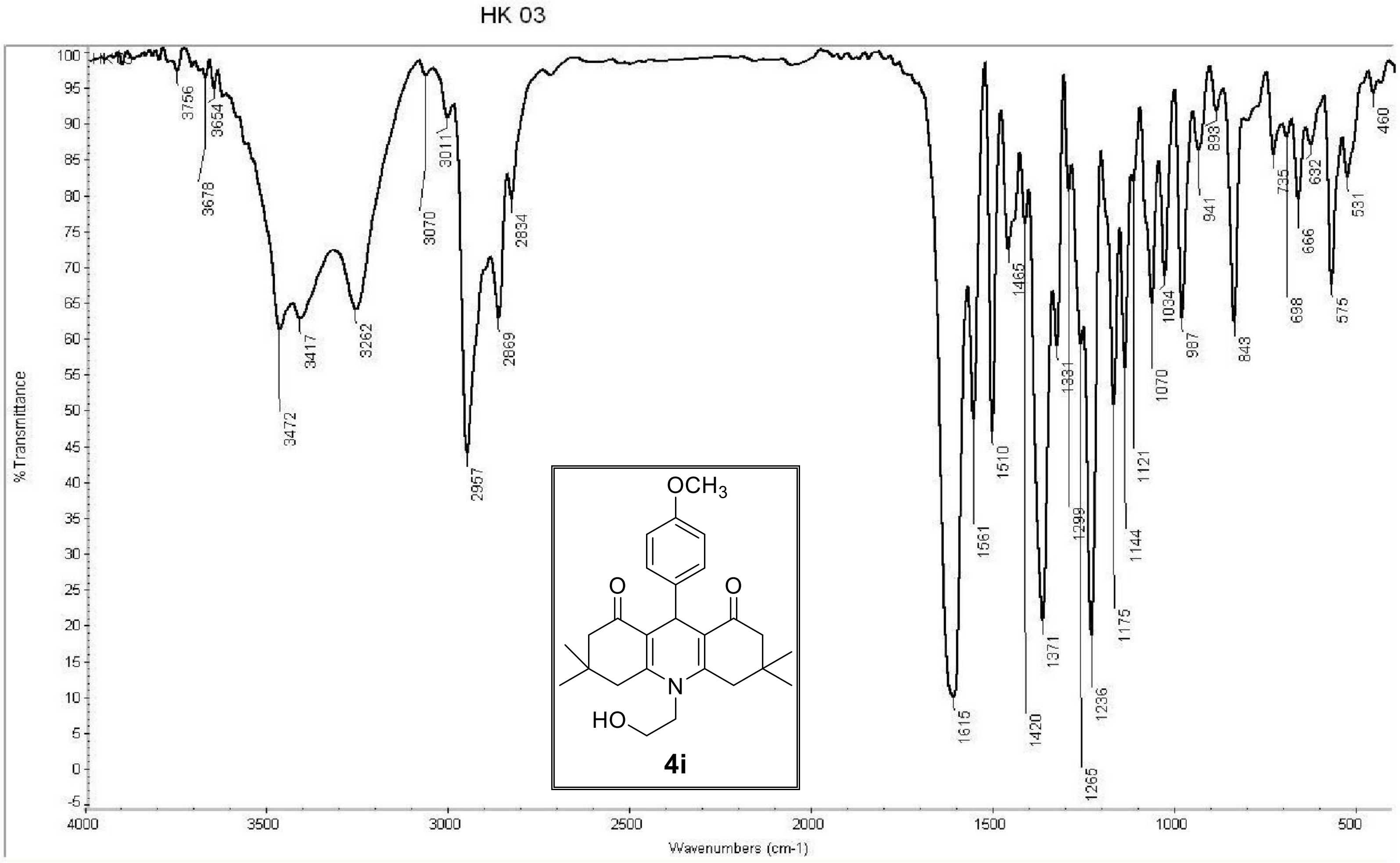

HK 03
% Transmittance
Wavenumbers (cm-1)
3756
3654
3678
3472
3417
3262
3070
3011
2957
2869
2834
1615
1561
1510
1465
1420
1371
1331
1299
1265
1236
1175
1144
1121
1070
1034
987
941
893
843
735
698
666
632
575
531
460
OCH3
O
O
N
HO
4i

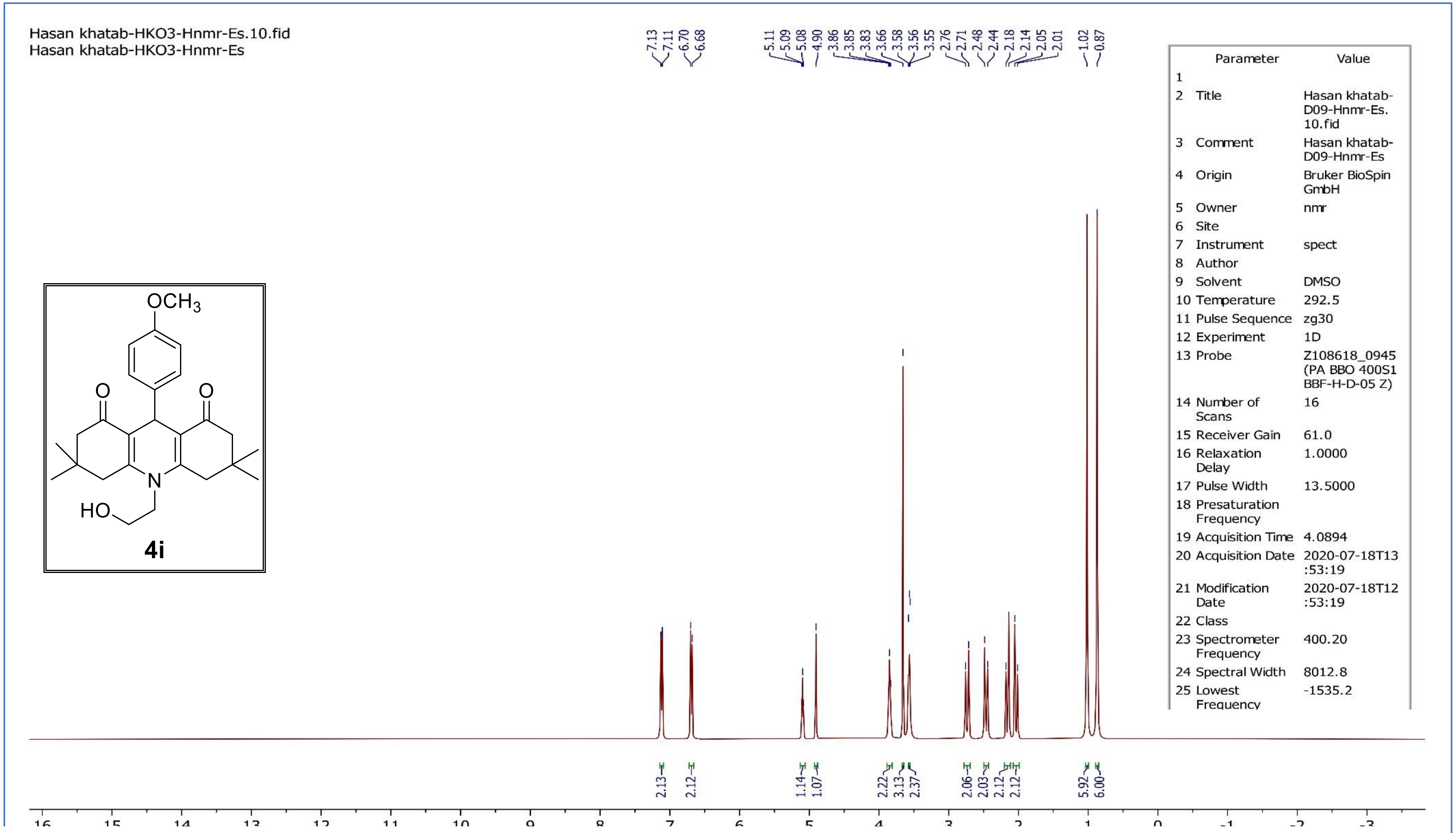
Hasan khatab-HKO3-Hnmr-Es.10.fid
Hasan khatab-HKO3-Hnmr-Es
7.13 7.11 6.70 6.68 5.11 5.09 5.08 4.90 3.86 3.85 3.83 3.66 3.58 3.56 3.55 2.76 2.71 2.48 2.44 2.18 2.14 2.05 2.01 1.02 0.87
OCH3
O
O
N
HO
4i
2.13 2.12 1.14 1.07 2.22 3.13 2.37 2.06 2.03 2.12 2.12 5.92 6.00
Parameter Value
1
2 Title Hasan khatab-D09-Hnmr-Es.10.fid
3 Comment Hasan khatab-D09-Hnmr-Es
4 Origin Bruker BioSpin GmbH
5 Owner nmr
6 Site
7 Instrument spect
8 Author
9 Solvent DMSO
10 Temperature 292.5
11 Pulse Sequence zg30
12 Experiment 1D
13 Probe Z108618_0945 (PA BBO 400S1 BBF-H-D-05 Z)
14 Number of Scans 16
15 Receiver Gain 61.0
16 Relaxation Delay 1.0000
17 Pulse Width 13.5000
18 Presaturation Frequency
19 Acquisition Time 4.0894
20 Acquisition Date 2020-07-18T13:53:19
21 Modification Date 2020-07-18T12:53:19
22 Class
23 Spectrometer Frequency 400.20
24 Spectral Width 8012.8
25 Lowest Frequency -1535.2

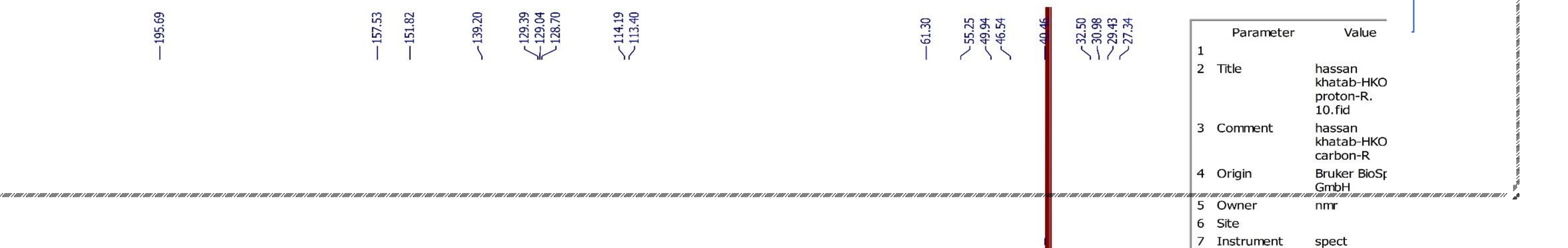
195.69 157.53 151.82 139.20 129.39 129.04 128.70 114.19 113.40 61.30 55.25 49.94 46.54 32.50 30.98 29.43 27.34
Parameter Value
1
2 Title hassan khatab-HKO proton-R.10.fid
3 Comment hassan khatab-HKO carbon-R
4 Origin Bruker BioSp GmbH
5 Owner nmr
6 Site
7 Instrument spect

Sample Information

Acquired by : System Administrator
Date Acquired : 07/03/2022 02:40:57 م
Sample Type : Unknown
Sample Name : D09
Sample ID :
Dilution Factor : 1
Tray# : 1
Vial# : 27
Injection Volume : 10
Data File : [illegible]7.lcd
Method File : Method_MS_only.lcm
Original Method File : Method_MS_only.lcm
Report Format File : MS Spectrum.lsr
Tuning File : default.lct
Processed by : System Administrator
Date Processed : 10/03/2022 11:53:58 ص

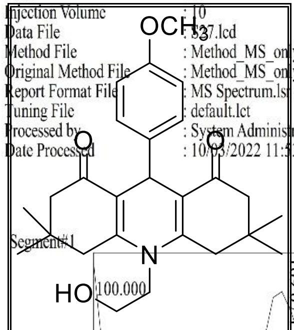

4i

MS Chromatogram

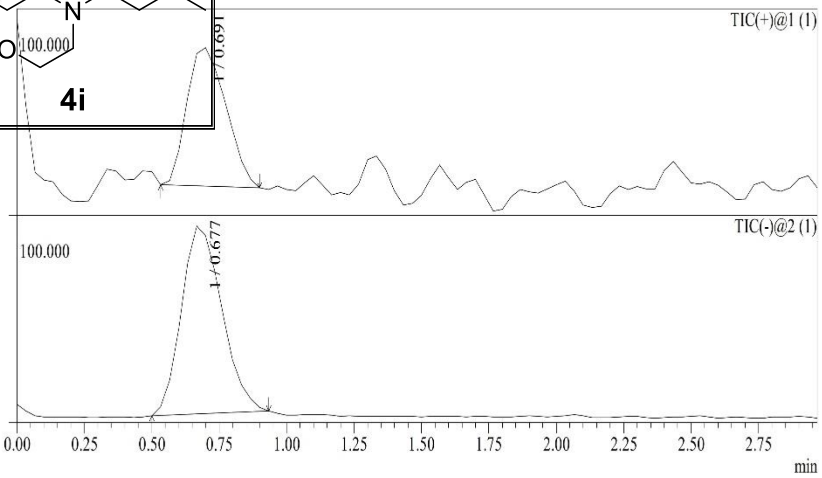

MASS Peak Table ALL MC

Peak#	Ret. Time	m/z	Area	Area%	Mark	A/H	Event#
1	0.691	TIC	64311781	100.000	M	10.057	1-1
2	0.677	TIC	31566970	100.000	M	10.468	1-2
Total			95878751	200.000			

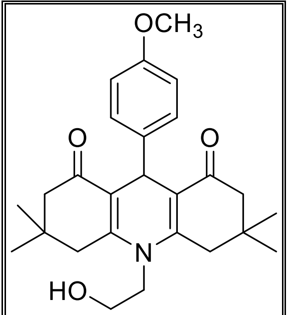

4i

Line#:1 R.Time:0.667(Scan#:41)
MassPeaks:584
Spectrum Mode:Averaged 0.633-0.700(39-43) Base Peak:424(1633750)
BG Mode:Calc Segment 1 - Event 1

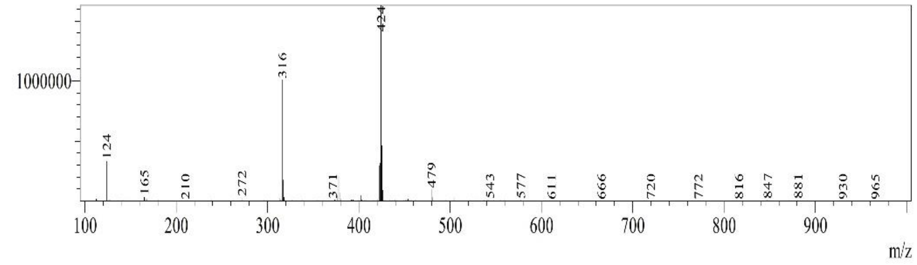

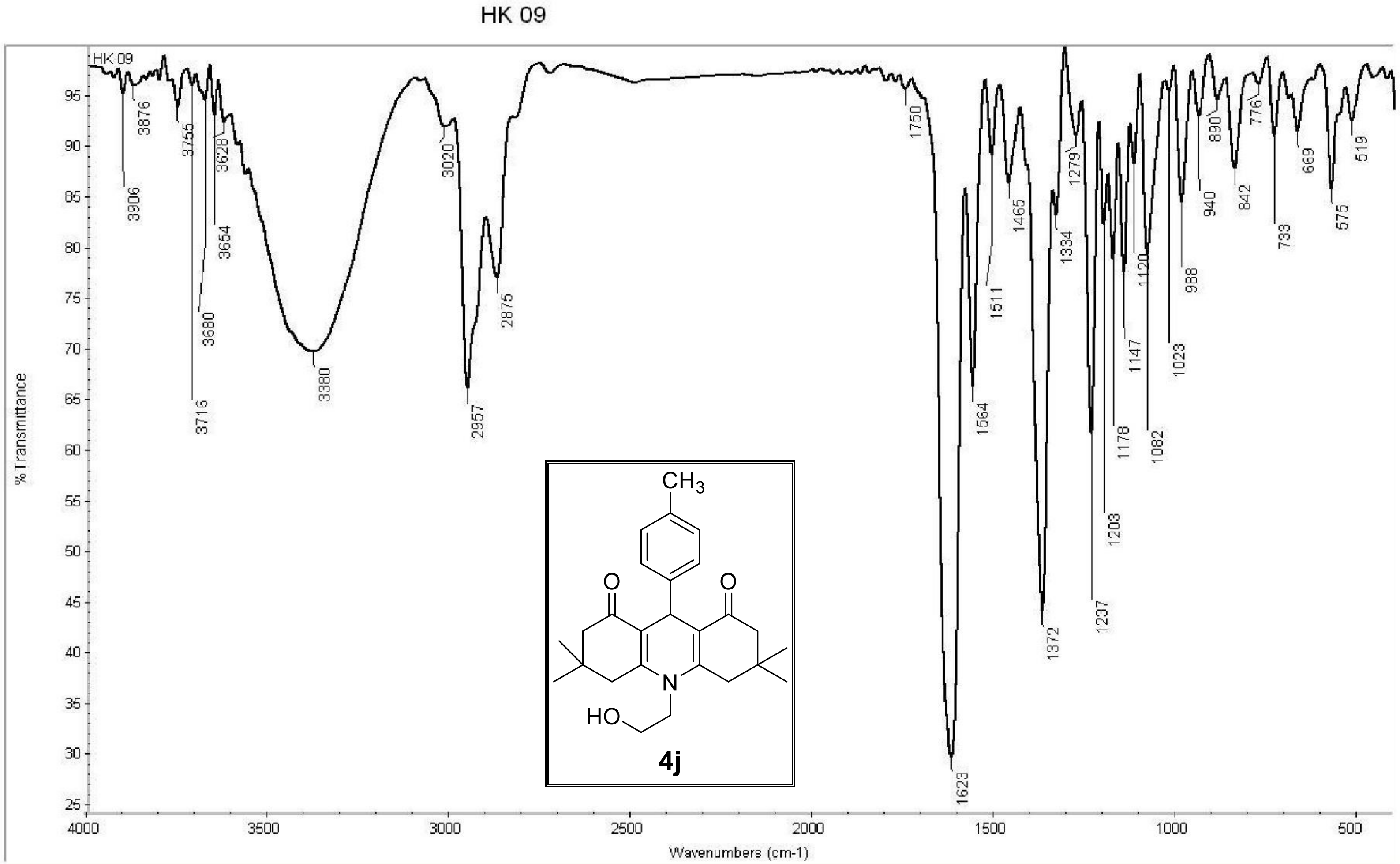
HK 09
%Transmittance
Wavenumbers (cm-1)
3906
3876
3755
3716
3680
3654
3628
3380
3020
2957
2875
1750
1623
1564
1511
1465
1372
1334
1279
1237
1203
1178
1147
1120
1082
1023
988
940
890
842
776
733
669
575
519
CH3
O
O
N
HO
4j

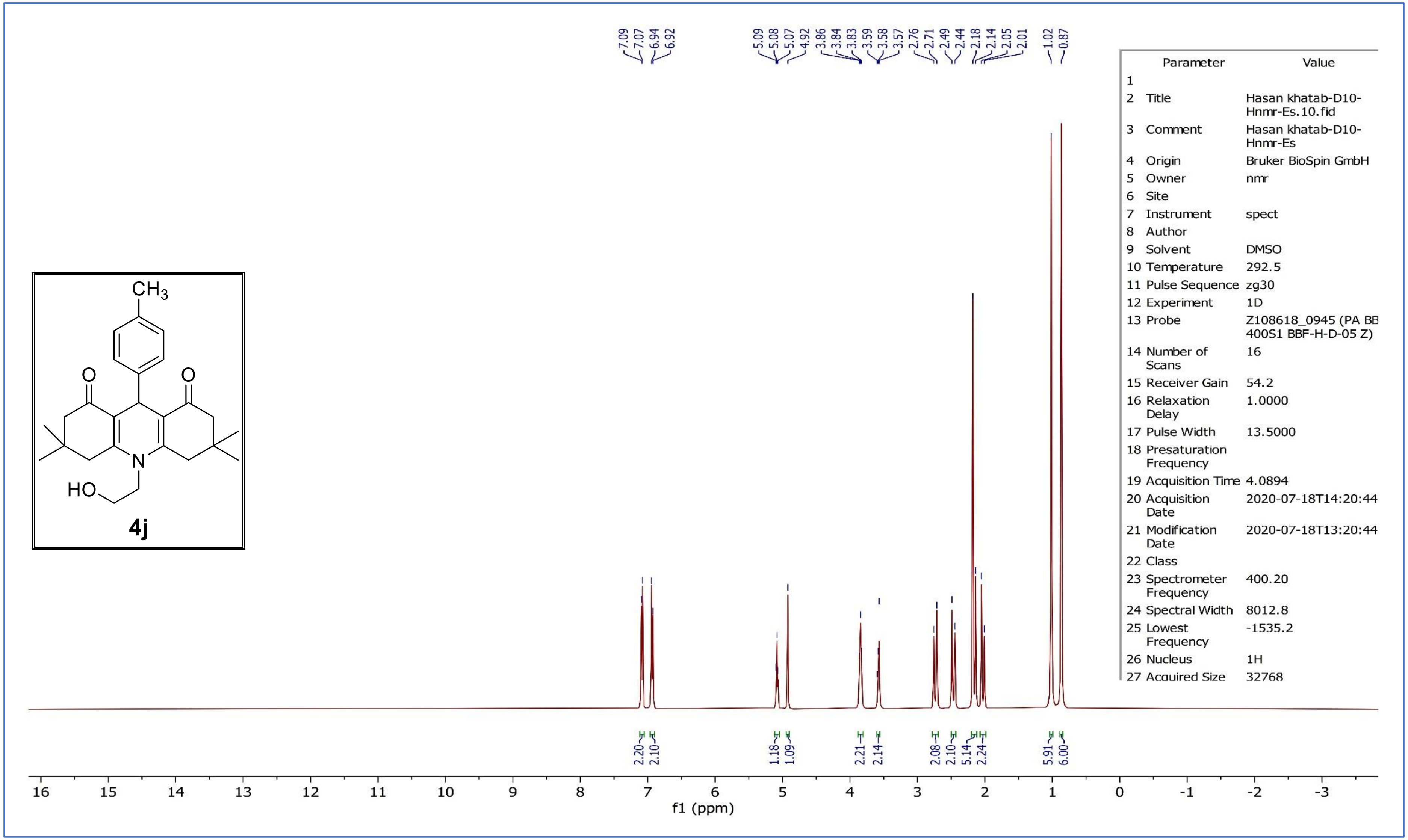
CH3
O
O
N
HO
4j
7.09
7.07
6.94
6.92
5.09
5.08
5.07
4.92
3.86
3.84
3.83
3.59
3.58
3.57
2.76
2.71
2.49
2.44
2.18
2.14
2.05
2.01
1.02
0.87
2.20
2.10
1.18
1.09
2.21
2.14
2.08
2.10
5.14
2.24
5.91
6.00
16 15 14 13 12 11 10 9 8 7 6 5 4 3 2 1 0 -1 -2 -3
f1 (ppm)
Parameter Value
1
2 Title Hasan khatab-D10-Hnmr-Es.10.fid
3 Comment Hasan khatab-D10-Hnmr-Es
4 Origin Bruker BioSpin GmbH
5 Owner nmr
6 Site
7 Instrument spect
8 Author
9 Solvent DMSO
10 Temperature 292.5
11 Pulse Sequence zg30
12 Experiment 1D
13 Probe Z108618_0945 (PA BB 400S1 BBF-H-D-05 Z)
14 Number of Scans 16
15 Receiver Gain 54.2
16 Relaxation Delay 1.0000
17 Pulse Width 13.5000
18 Presaturation Frequency
19 Acquisition Time 4.0894
20 Acquisition Date 2020-07-18T14:20:44
21 Modification Date 2020-07-18T13:20:44
22 Class
23 Spectrometer Frequency 400.20
24 Spectral Width 8012.8
25 Lowest Frequency -1535.2
26 Nucleus 1H
27 Acquired Size 32768

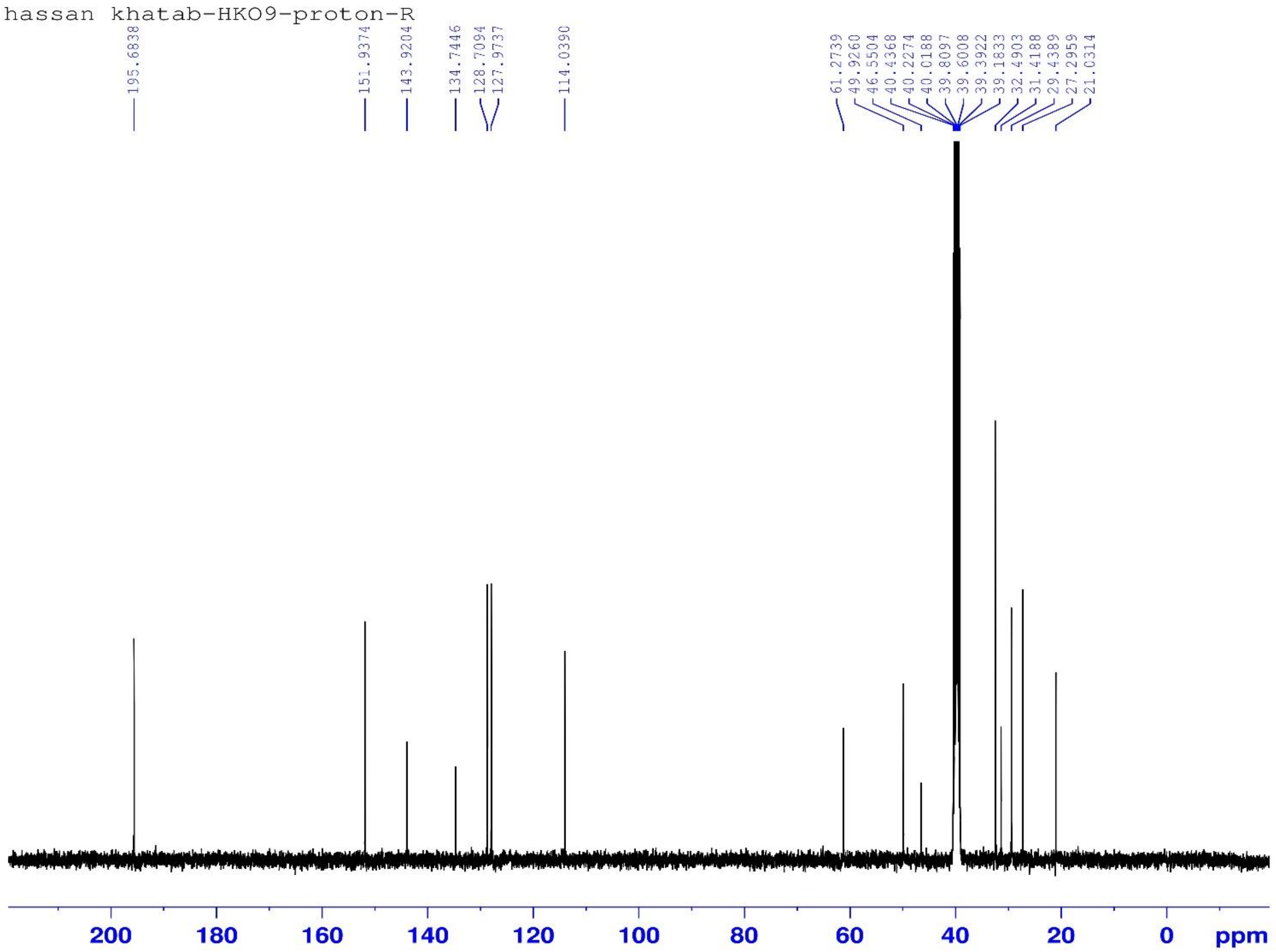

Current Data Parameters

NAME	hassan khatab-HKO9-proton-R
EXPNO	10
PROCNO	1

F2 - Acquisition Parameters

Date_	20200726	
Time	15.41	h
INSTRUM	spect	
PROBHD	Z108618_0945 (	
PULPROG	zgpg30	
TD	65536	
SOLVENT	DMSO	
NS	1200	
DS	4	
SWH	24038.461	Hz
FIDRES	0.733596	Hz
AQ	1.3631488	sec
RG	197.77	
DW	20.800	usec
DE	6.50	usec
TE	292.7	K
D1	2.00000000	sec
D11	0.03000000	sec
TD0	1	
SFO1	100.6404331	MHz
NUC1	13C	
P1	10.00	usec
PLW1	47.00000000	W
SFO2	400.2016008	MHz
NUC2	1H	
CPDPRG[2	waltz16	
PCPD2	90.00	usec
PLW2	13.00000000	W
PLW12	0.29249999	W
PLW13	0.14713000	W

F2 - Processing parameters

SI	32768	
SF	100.6303700	MHz
WDW	EM	
SSB	0	
LB	1.00	Hz
GB	0	
PC	1.40	

Sample Information

Acquired by	: System Administrator
Date Acquired	: 01/12/2021 01:28:36 م
Sample Type	: Unknown
Sample Name	: D10
Sample ID	:
Dilution Factor	: 1
Tray#	: 1
Vial#	: 10
Injection Volume	: 10
Data File	: S_10.lcd
Method File	: Method_MS_only.lcm
Original Method File	: Method_MS_only.lcm
Report Format File	: DEFAULT.lsr
Tuning File	: default.lct
Processed by	: System Administrator
Date Processed	: 05/12/2021 09:39:52 ص

MS Chromatogram

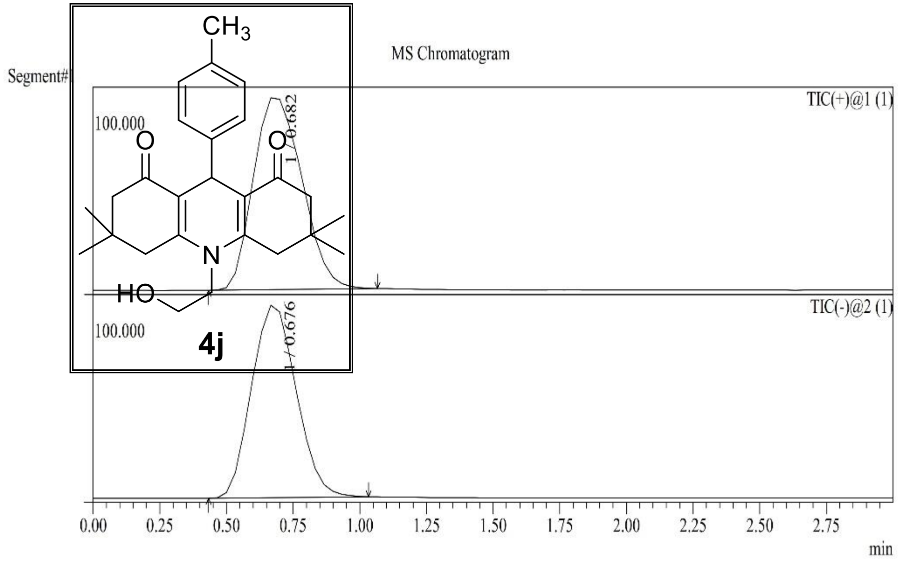

Line#:1 R.Time:0.667(Scan#:41)
MassPeaks:830
Spectrum Mode:Averaged 0.633-0.700(39-43) Base Peak:408(7789424)
BG Mode:Calc Segment 1 - Event 1

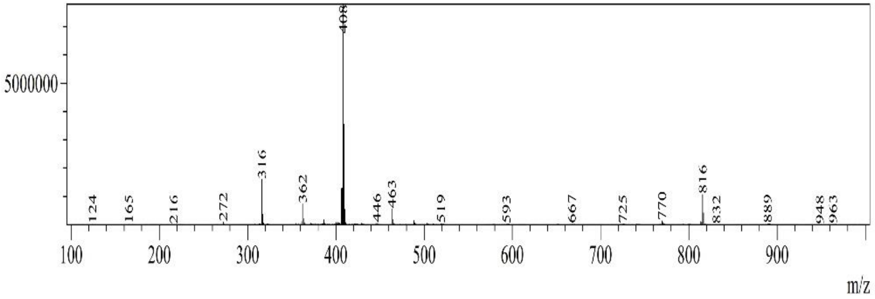

MASS Peak Table ALL MC

Peak#	Ret. Time	m/z	Area	Area%	Mark	A/H	Event#
1	0.682	TIC	324097339	100.000		12.703	1-1
2	0.676	TIC	663610466	100.000		11.673	1-2
Total			987707805	200.000			

LC-MS Spectrum of compound **4j**

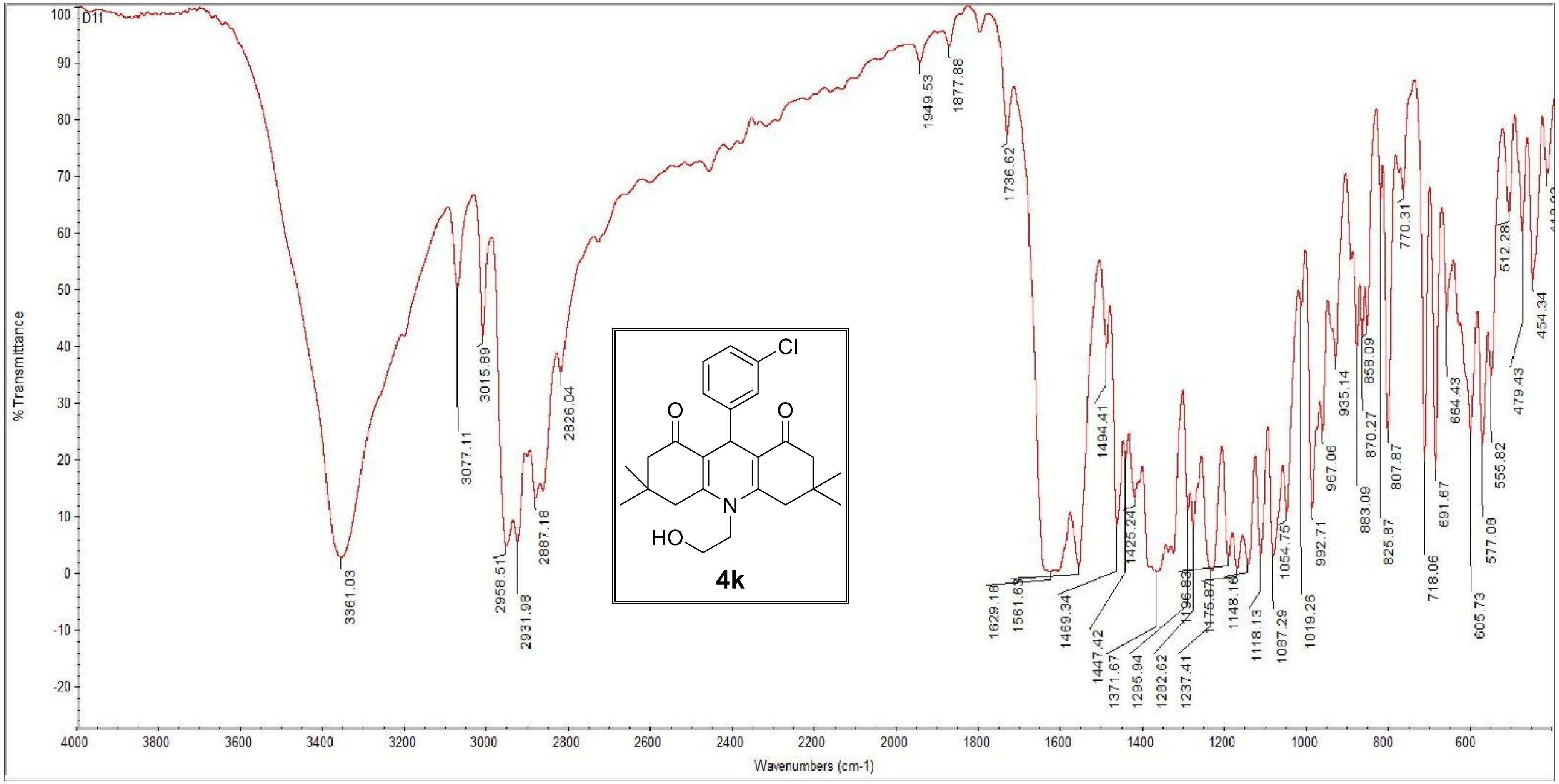

IR Spectrum of compound **4k**

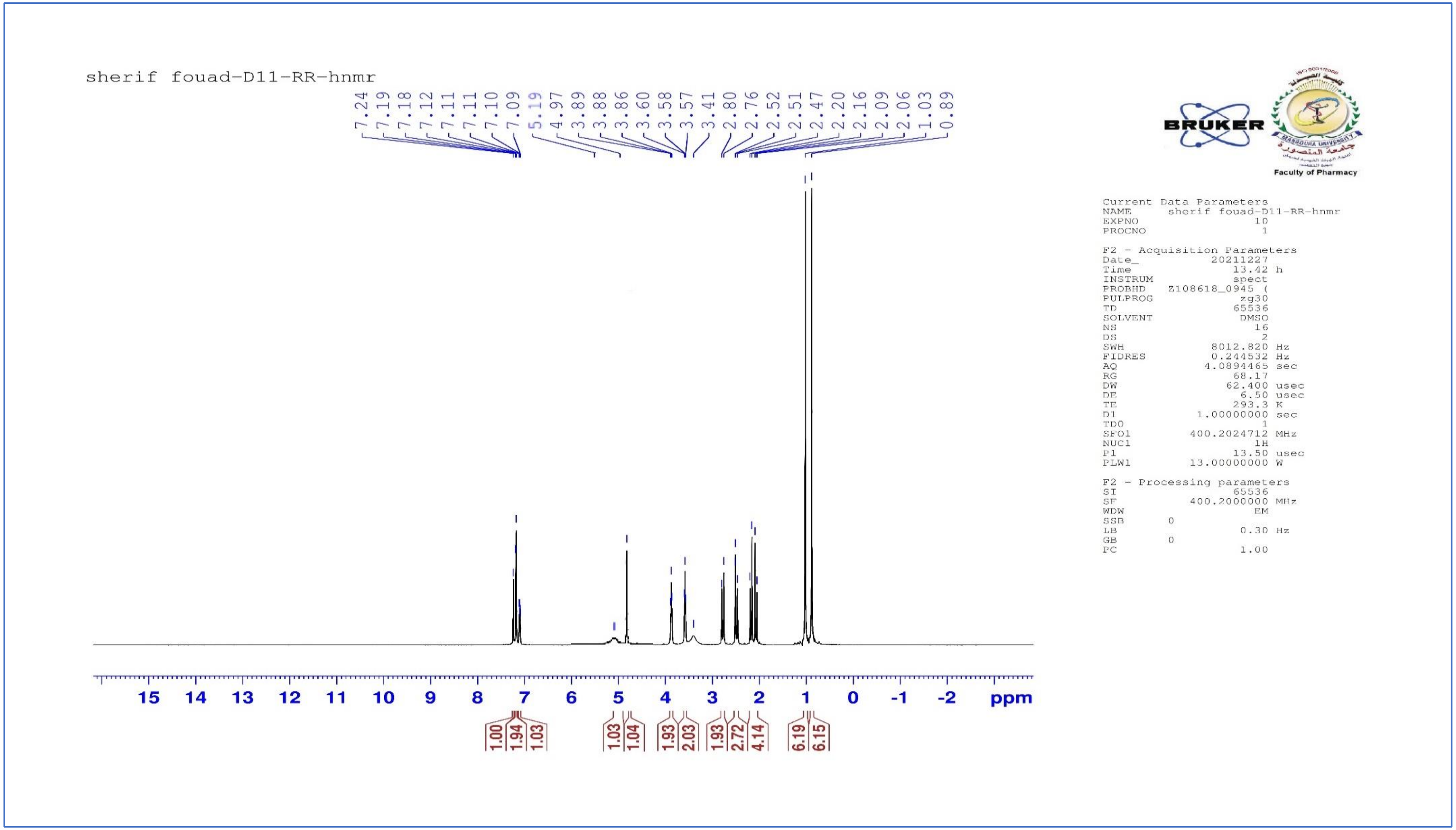

^{1}H NMR Spectrum of compound **4k**

Sample Information

Acquired by : System Administrator
Date Acquired : 01/12/2021 01:34:08 م
Sample Type : Unknown
Sample Name : D11
Sample ID :
Dilution Factor : 1
Tray# : 1
Vial# : 11
Injection Volume : 10
Data File : S_11.lcd
Method File : Method_MS_only.lcm
Original Method File : Method_MS_only.lcm
Report Format File : DEFAULT.lsr
Tuning File : default.lct
Processed by : System Administrator
Date Processed : 05/12/2021 09:40:13 ص

MS Chromatogram

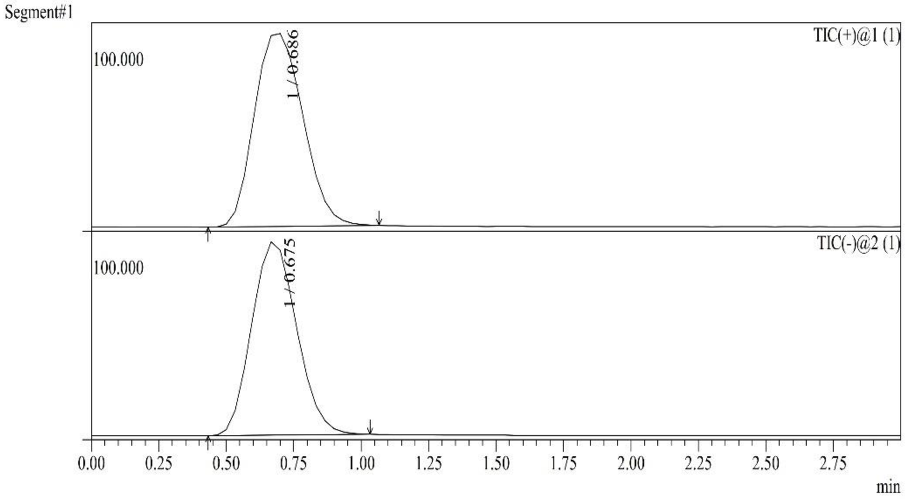

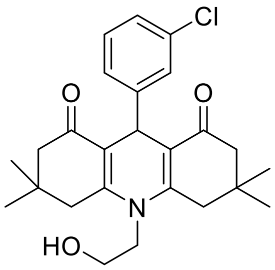

4k

Line#:1 R.Time:0.667(Scan#:41)
MassPeaks:835
Spectrum Mode:Averaged 0.633-0.700(39-43) Base Peak:428(6820534)
BG Mode:Calc Segment 1 - Event 1

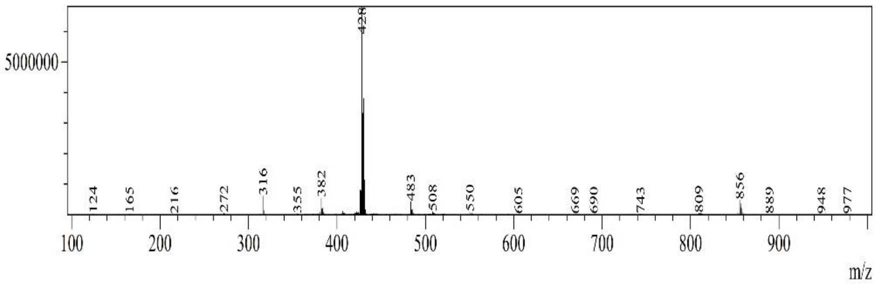

MASS Peak Table ALL MC

Peak#	Ret. Time	m/z	Area	Area%	Mark	A/H	Event#
1	0.686	TIC	319126426	100.000		12.579	1-1
2	0.675	TIC	776951728	100.000		11.624	1-2
Total			1096078154	200.000			

LC-MS Spectrum of compound **4k**

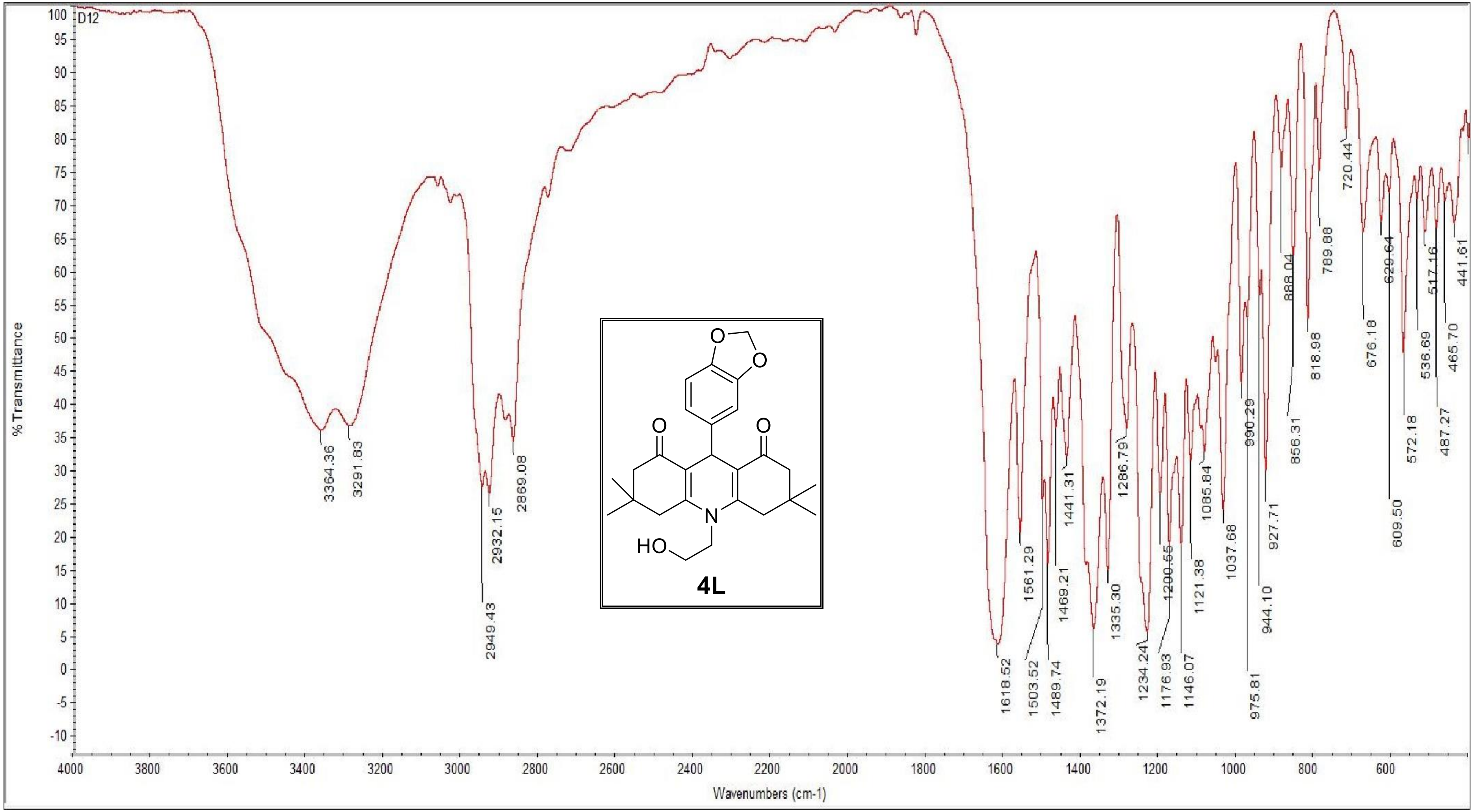

IR Spectrum of compound **4L**

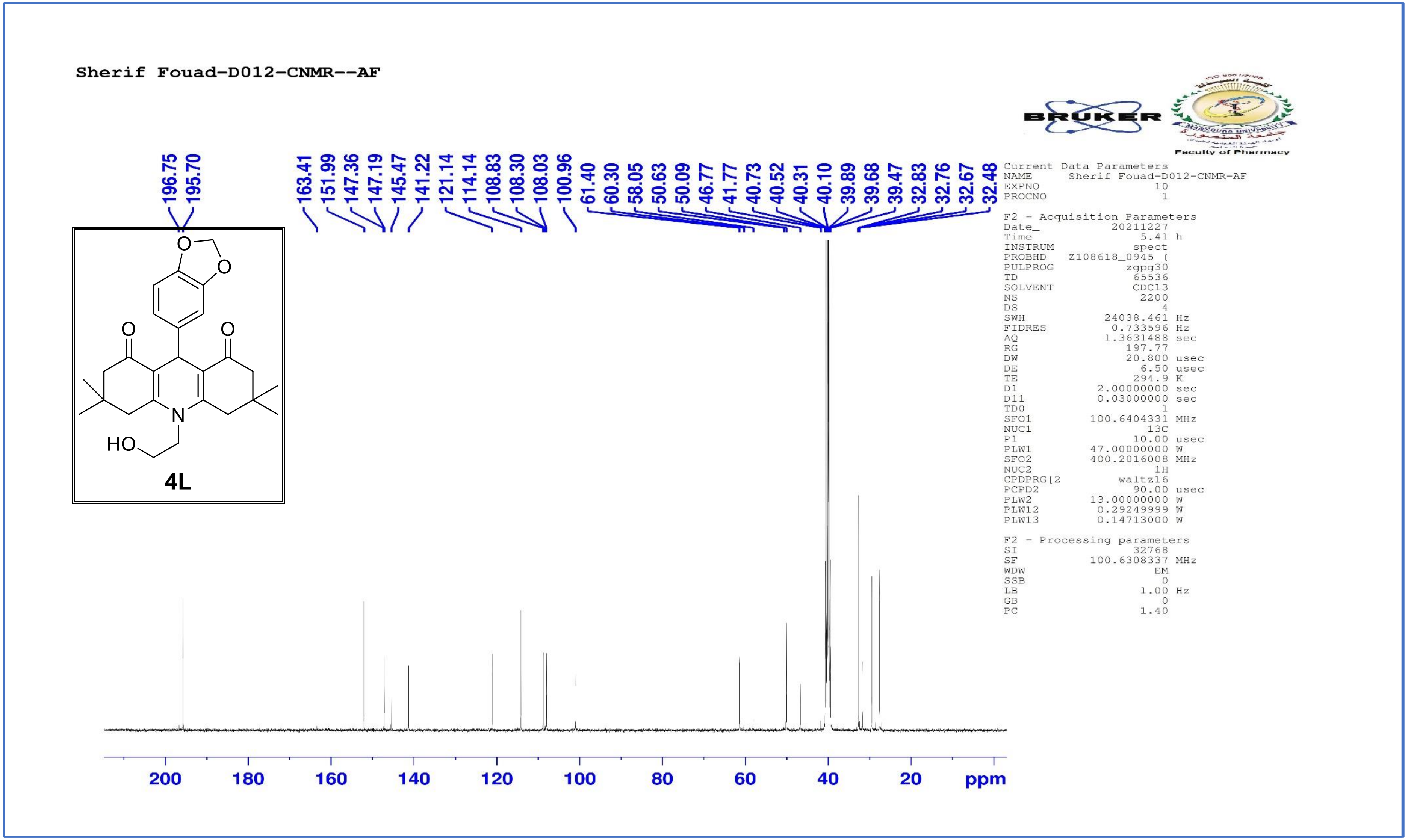

^{13}C NMR Spectrum of compound **4L**

Sample Information

Acquired by	: System Administrator
Date Acquired	: 01/12/2021 01:39:39 م
Sample Type	: Unknown
Sample Name	: D12
Sample ID	:
Dilution Factor	: 1
Tray#	: 1
Vial#	: 12
Injection Volume	: 10
Data File	: S_12.lcd
Method File	: Method_MS_only.lcm
Original Method File	: Method_MS_only.lcm
Report Format File	: DEFAULT.lsr
Tuning File	: default.lt
Processed by	: System Administrator
Date Processed	: 05/12/2021 09:40:53 ص

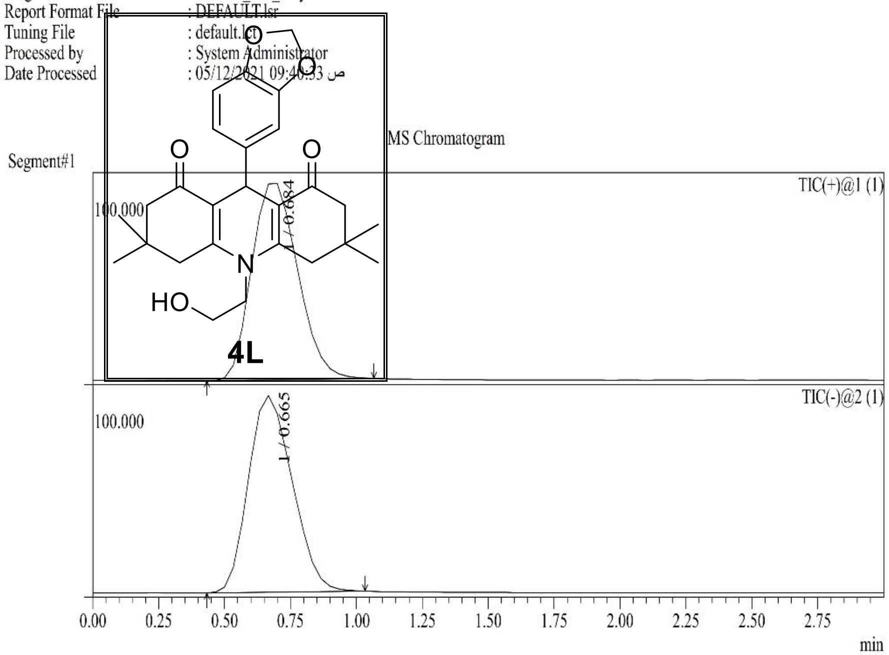

Line#:1 R.Time:0.667(Scan#:41)
MassPeaks:881
Spectrum Mode:Averaged 0.633-0.700(39-43) Base Peak:438(6505764)
BG Mode:Calc Segment 1 - Event 1

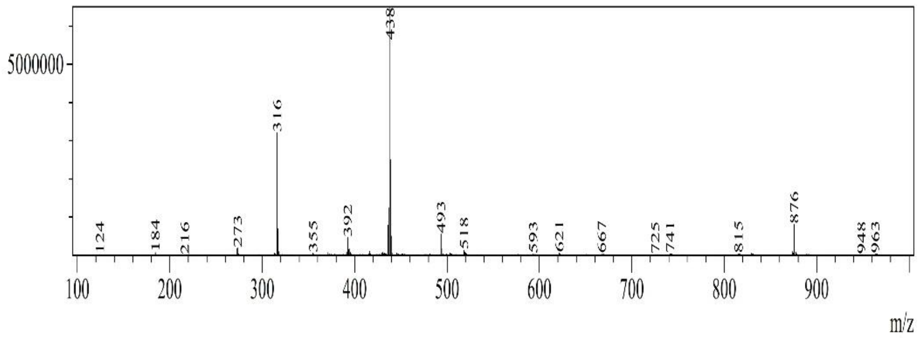

MASS Peak Table ALL MC

Peak#	Ret. Time	m/z	Area	Area%	Mark	A/H	Event#
1	0.684	TIC	301373471	100.000		12.181	1-1
2	0.665	TIC	633517178	100.000		11.599	1-2
Total			934890649	200.000			

LC-MS Spectrum of compound **4L**

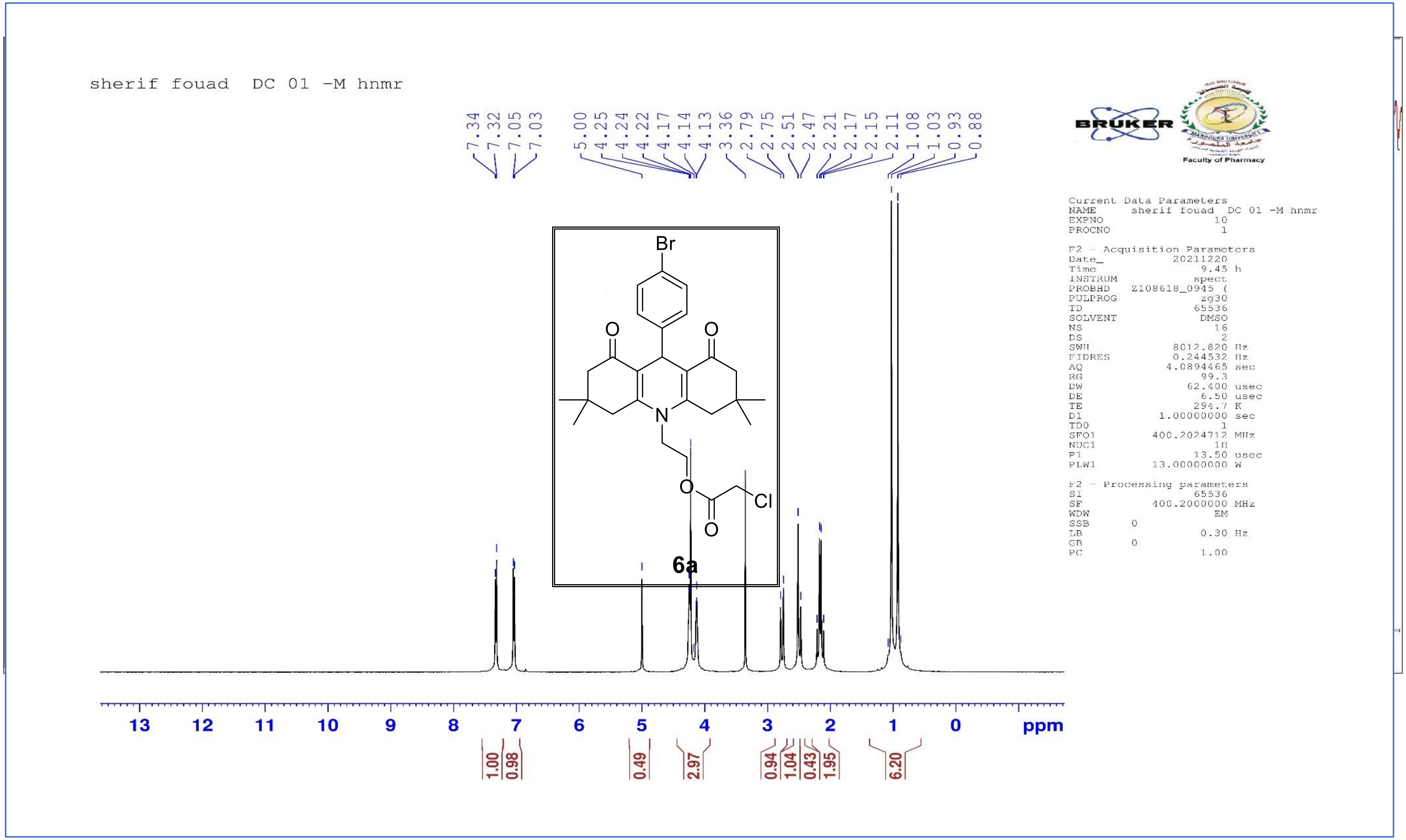

^{1}H NMR Spectrum of compound **6a**

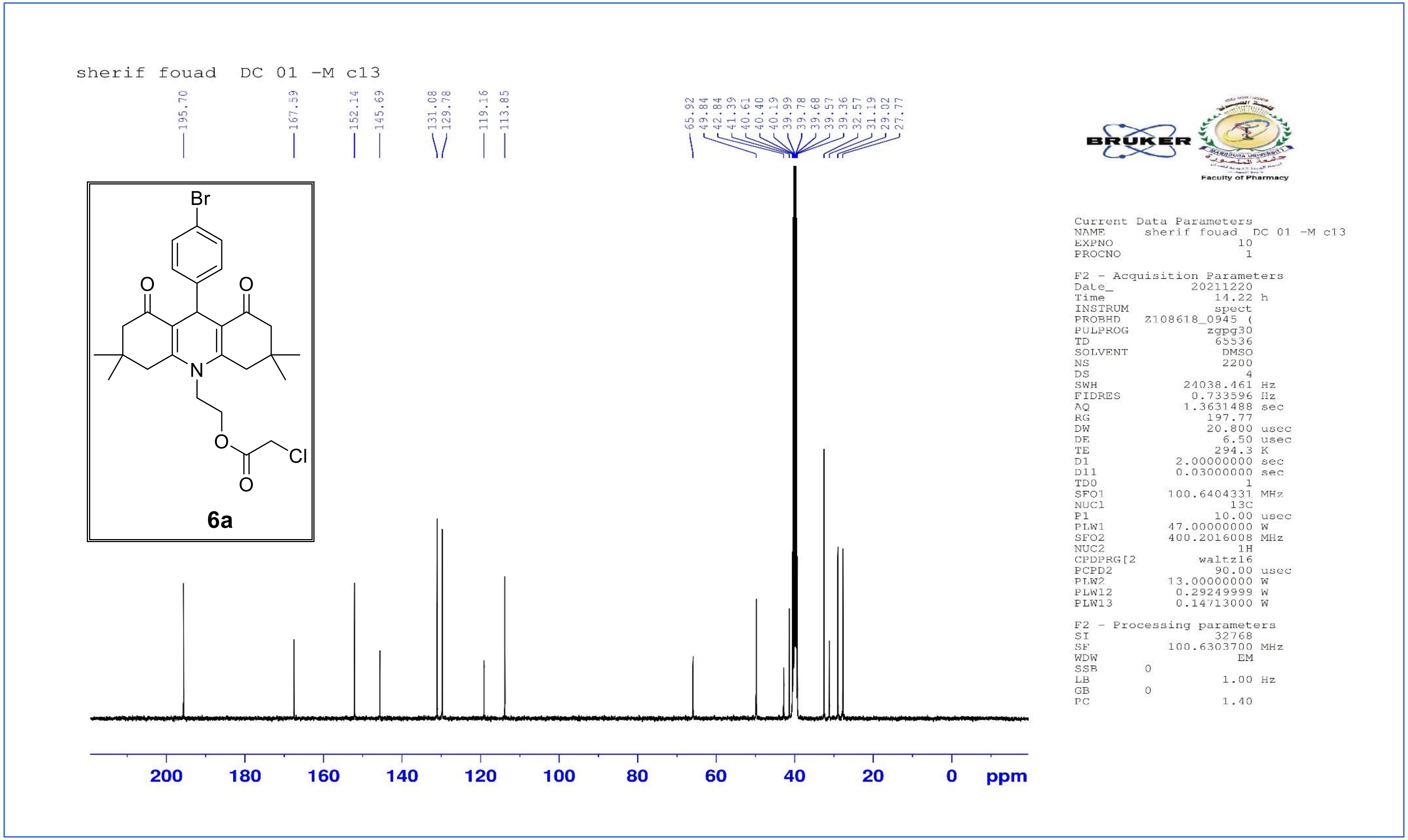

^{13}C NMR Spectrum of compound **6a**

Sample Information

Acquired by	: System Administrator
Date Acquired	: 01/12/2021 01:45:08 م
Sample Type	: Unknown
Sample Name	: DC01
Sample ID	:
Dilution Factor	: 1
Tray#	: 1
Vial#	: 13
Injection Volume	: 10
Data File	: S_13.lcd
Method File	: Method_MS_only.lcm
Original Method File	: Method_MS_only.lcm
Report Format File	: DEFAULT.lsr
Tuning File	: default.lct
Processed by	: System Administrator
Date Processed	: 05/12/2021 09:40:52 ص

MS Chromatogram

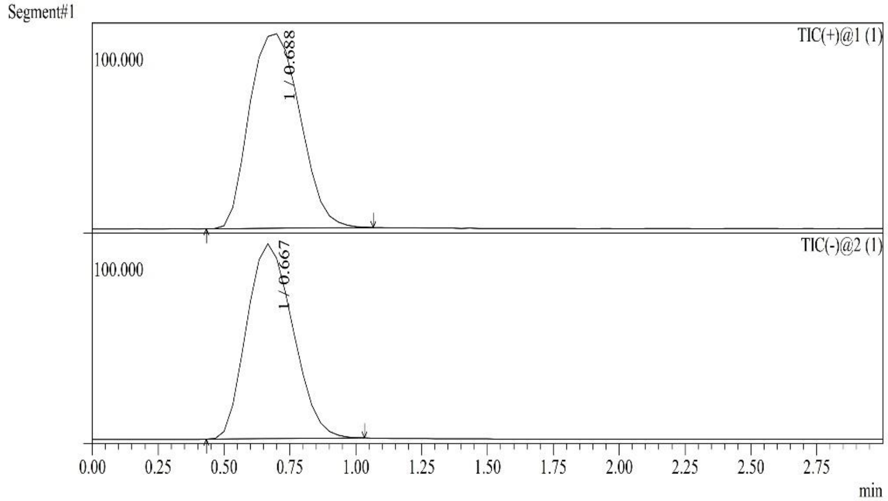

MASS Peak Table ALL MC

Peak#	Ret. Time	m/z	Area	Area%	Mark	A/H	Event#
1	0.688	TIC	414692556	100.000		13.370	1-1
2	0.667	TIC	992831844	100.000		12.232	1-2
Total			1407524400	200.000			

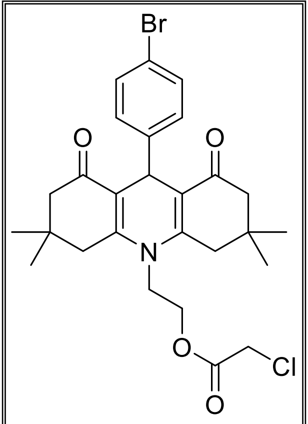

6a

Line#:1 R.Time:0.667(Scan#:41)
MassPeaks:759
Spectrum Mode:Averaged 0.633-0.700(39-43) Base Peak:550(7149911)
BG Mode:Calc Segment 1 - Event 1

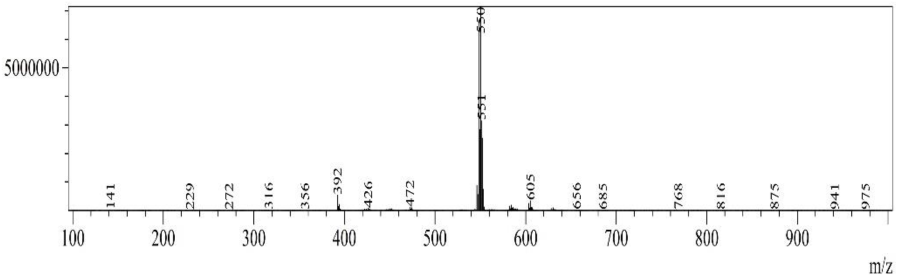

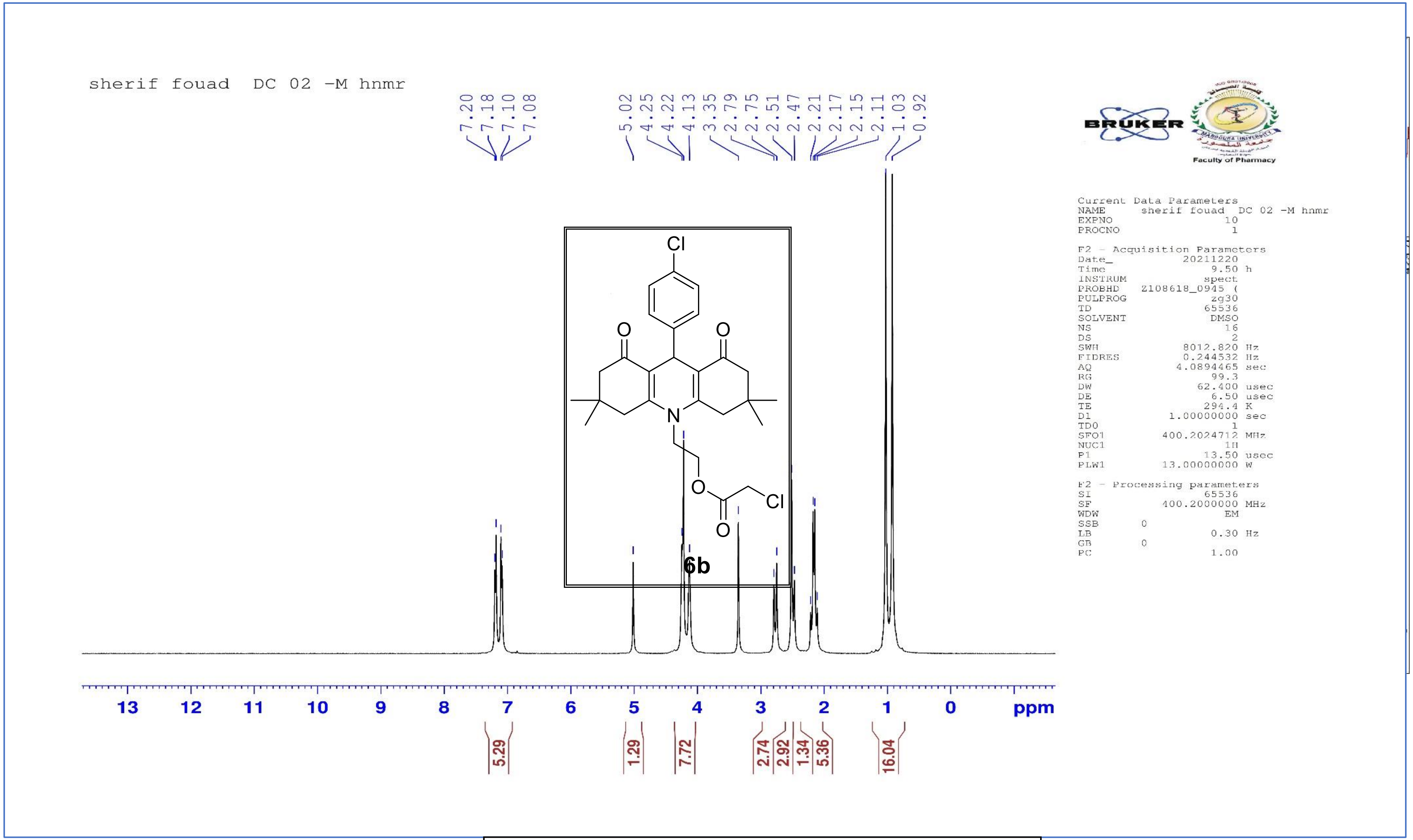

^{1}H NMR Spectrum of compound **6b**

Sample Information

Acquired by	: System Administrator
Date Acquired	: 01/12/2021 01:50:38 م
Sample Type	: Unknown
Sample Name	: DC02
Sample ID	:
Dilution Factor	: 1
Tray#	: 1
Vial#	: 14
Injection Volume	: 10
Data File	: S_14.lcd
Method File	: Method_MS_only.lcm
Original Method File	: Method_MS_only.lcm
Report Format File	: DEFAULT.lsr
Tuning File	: default.lct
Processed by	: System Administrator
Date Processed	: 05/12/2021 09:41:17 ص

MS Chromatogram

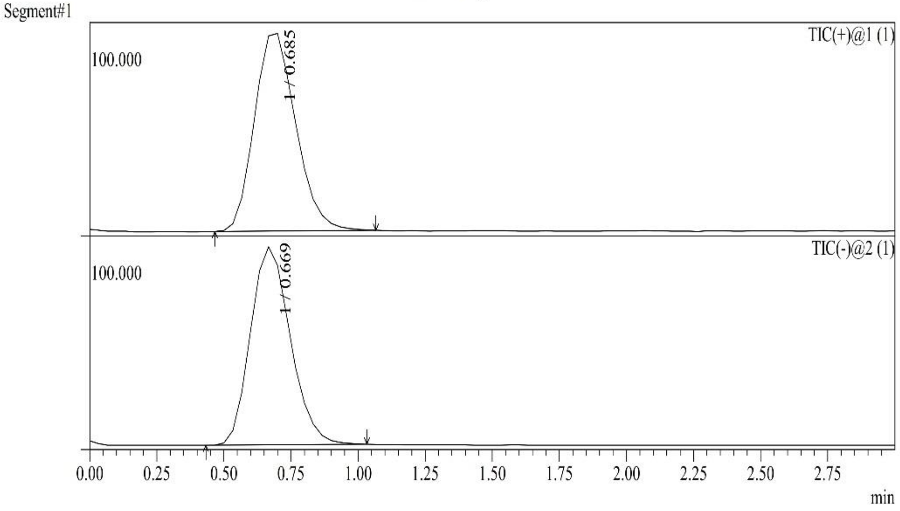

MASS Peak Table ALL MC

Peak#	Ret. Time	m/z	Area	Area%	Mark	A/H	Event#
1	0.685	TIC	119431444	100.000		10.767	1-1
2	0.669	TIC	267850348	100.000		10.332	1-2
Total			387281792	200.000			

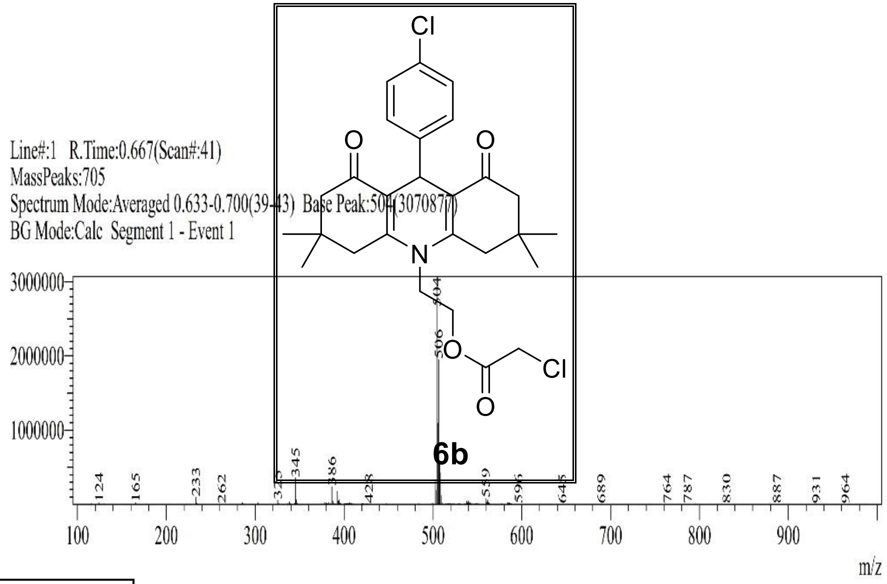

LC-MS Spectrum of compound **6b**

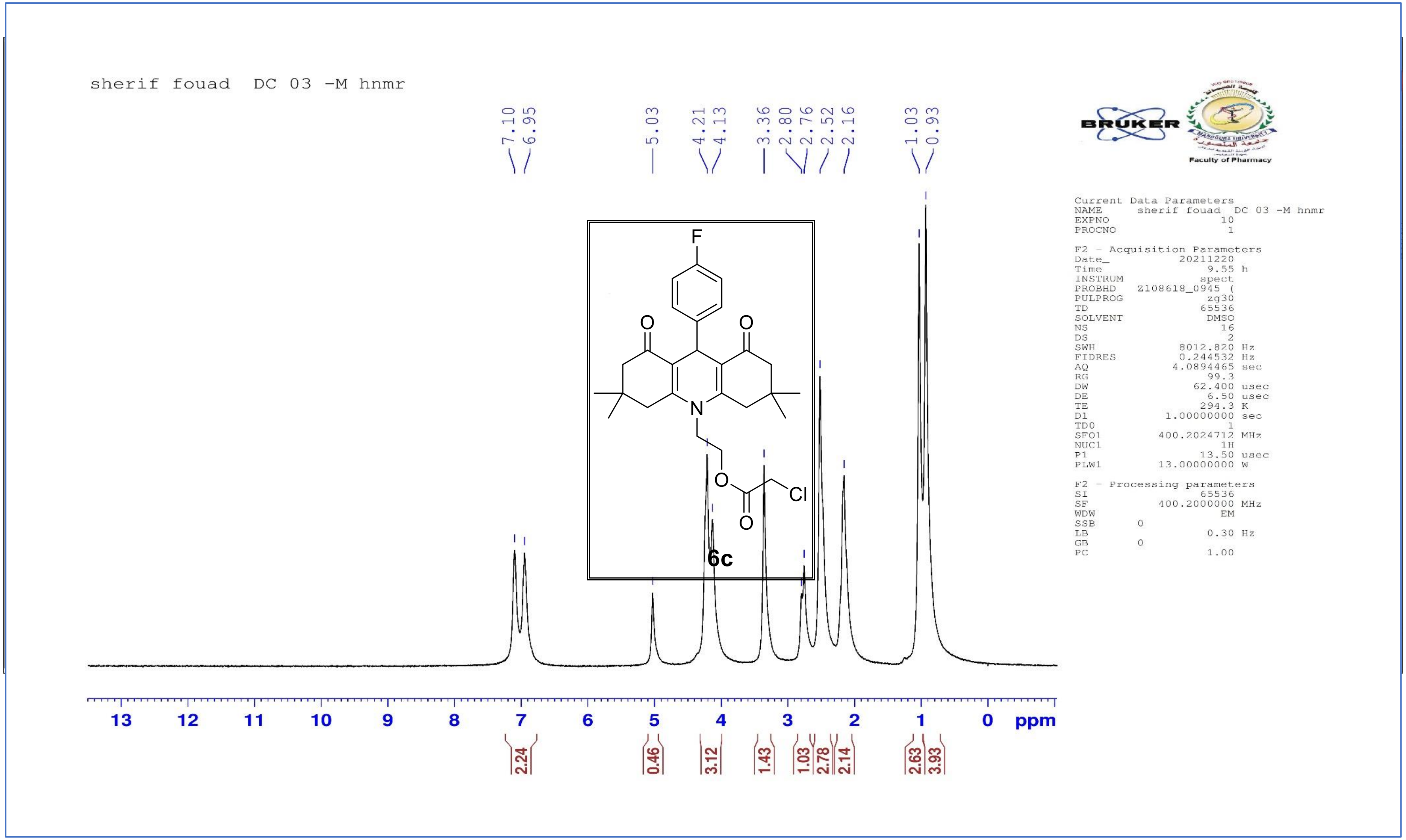

^{1}H NMR Spectrum of compound **6c**

Sample Information

Acquired by	: System Administrator
Date Acquired	: 01/12/2021 01:56:09 م
Sample Type	: Unknown
Sample Name	: DC03
Sample ID	:
Dilution Factor	: 1
Tray#	: 1
Vial#	: 15
Injection Volume	: 10
Data File	: S_15.lcd
Method File	: Method_MS_only.lcm
Original Method File	: Method_MS_only.lcm
Report Format File	: DEFAULT.lsr
Tuning File	: default.lct
Processed by	: System Administrator
Date Processed	: 05/12/2021 09:41:36 ص

MS Chromatogram

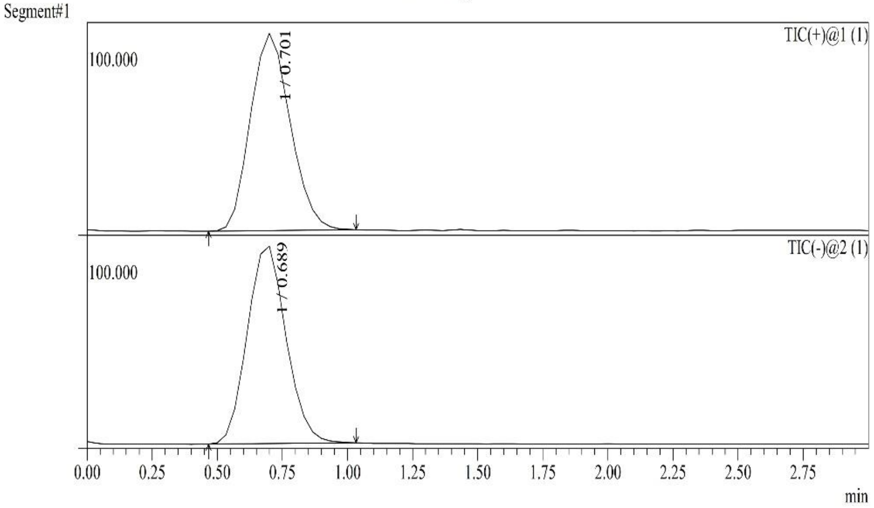

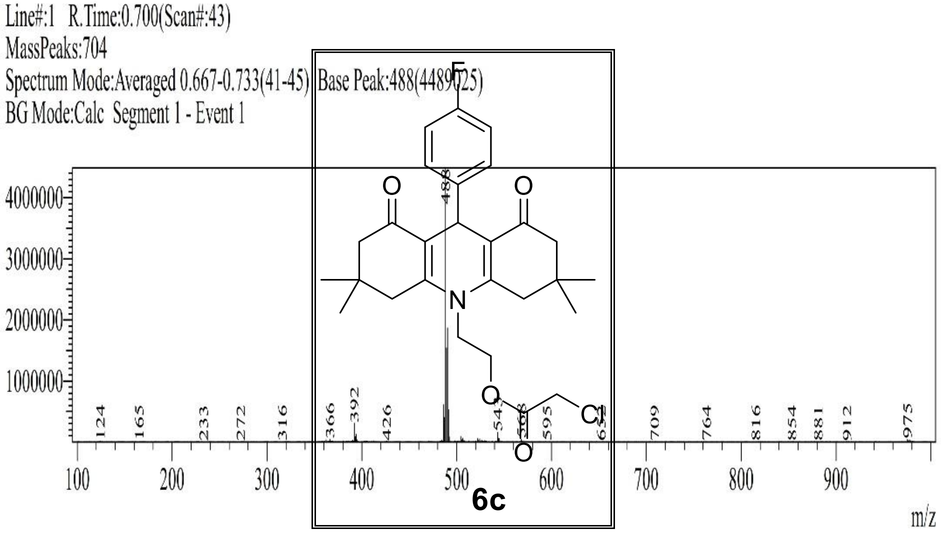

MASS Peak Table ALL MC

Peak#	Ret. Time	m/z	Area	Area%	Mark	A/H	Event#
1	0.701	TIC	136195145	100.000		10.639	1-1
2	0.689	TIC	287109947	100.000		10.399	1-2
Total			423305092	200.000			

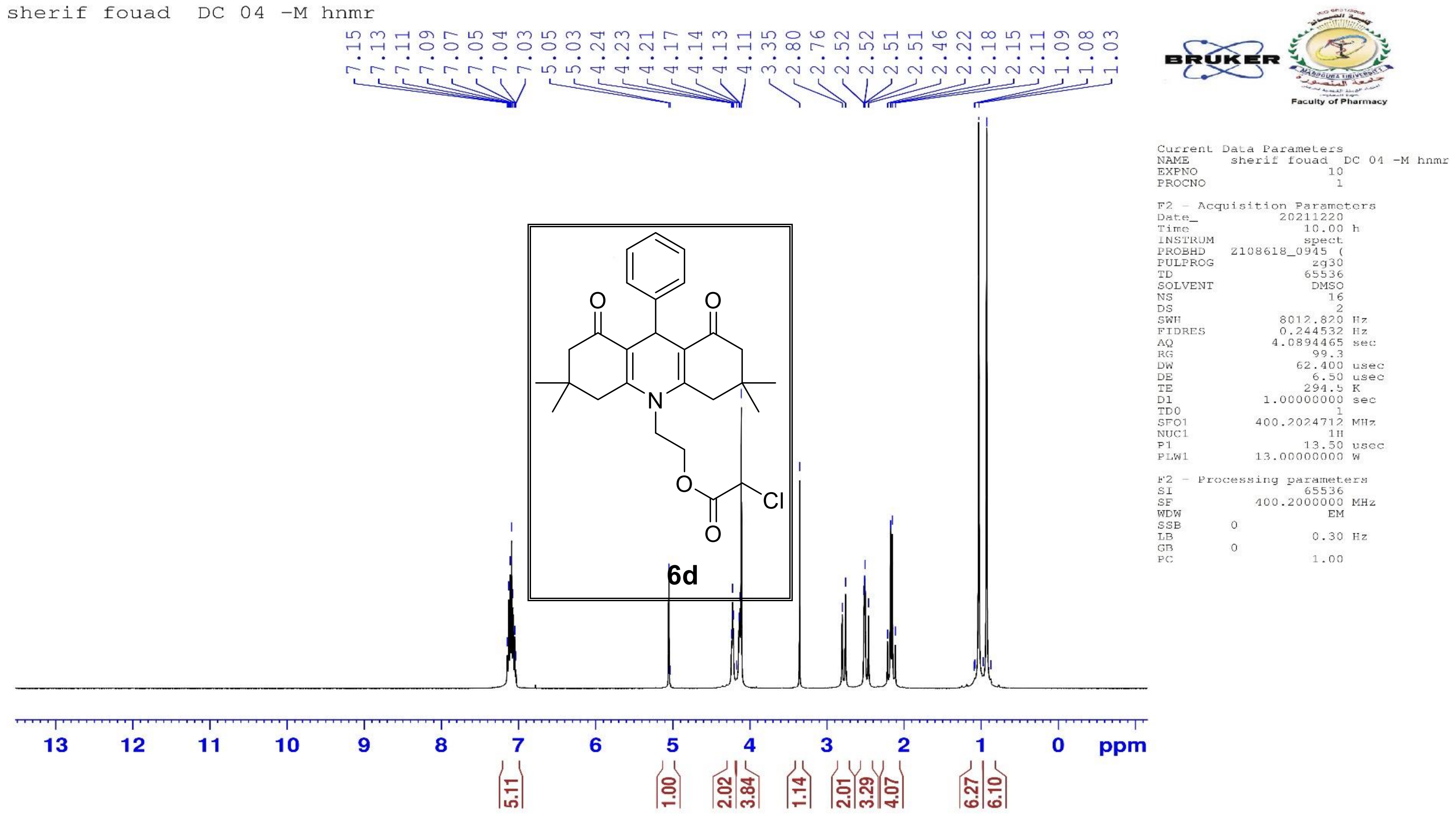
sherif fouad DC 04 -M hnmr
7.15 7.13 7.11 7.09 7.07 7.05 7.04 7.03 5.05 5.03 4.24 4.23 4.21 4.17 4.14 4.13 4.11 3.35 2.80 2.76 2.52 2.52 2.51 2.51 2.46 2.22 2.18 2.15 2.11 1.09 1.08 1.03
BRUKER
Faculty of Pharmacy
Current Data Parameters
NAME sherif fouad DC 04 -M hnmr
EXPNO 10
PROCNO 1
F2 - Acquisition Parameters
Date_ 20211220
Time 10.00 h
INSTRUM spect
PROBHD Z108618_0945 (
PULPROG zg30
TD 65536
SOLVENT DMSO
NS 16
DS 2
SWH 8012.820 Hz
FIDRES 0.244532 Hz
AQ 4.0894465 sec
RG 99.3
DW 62.400 usec
DE 6.50 usec
TE 294.5 K
D1 1.00000000 sec
TD0 1
SFO1 400.2024712 MHz
NUC1 1H
P1 13.50 usec
PLW1 13.00000000 W
F2 - Processing parameters
SI 65536
SF 400.2000000 MHz
WDW EM
SSB 0
LB 0.30 Hz
GB 0
PC 1.00
O
O
N
O
O
Cl
6d
13 12 11 10 9 8 7 6 5 4 3 2 1 0 ppm
5.11 1.00 2.02 3.84 1.14 2.01 3.29 4.07 6.27 6.10

Sample Information

Acquired by	: System Administrator
Date Acquired	: 01/12/2021 02:01:40 م
Sample Type	: Unknown
Sample Name	: DC04
Sample ID	:
Dilution Factor	: 1
Tray#	: 1
Vial#	: 16
Injection Volume	: 10
Data File	: S_16.lcd
Method File	: Method_MS_only.lcm
Original Method File	: Method_MS_only.lcm
Report Format File	: DEFAULT.lsr
Tuning File	: default.lct
Processed by	: System Administrator
Date Processed	: 05/12/2021 09:42:13 ص

MS Chromatogram

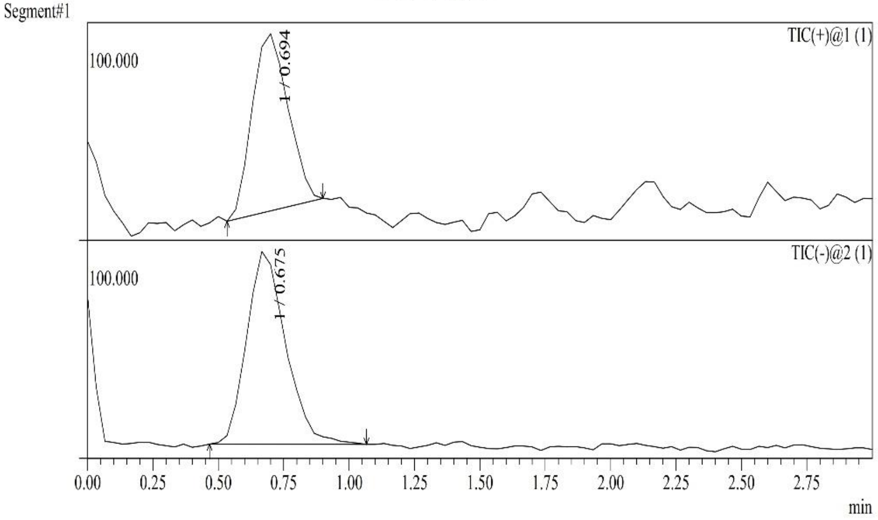

MASS Peak Table ALL MC

Peak#	Ret. Time	m/z	Area	Area%	Mark	A/H	Event#
1	0.694	TIC	2323188	100.000		9.720	1-1
2	0.675	TIC	4881636	100.000		10.388	1-2
Total			7204824	200.000			

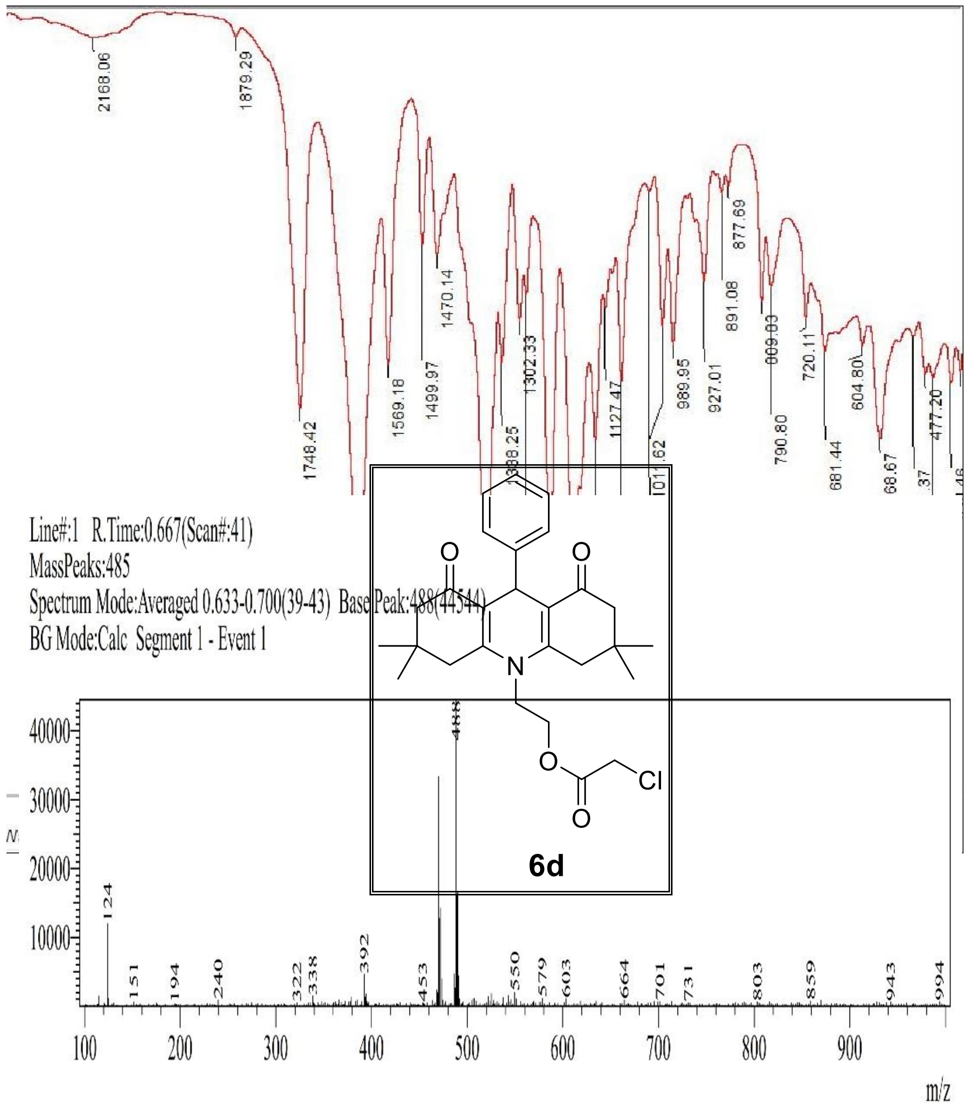

sherif fouad DC 05 -M hnmr

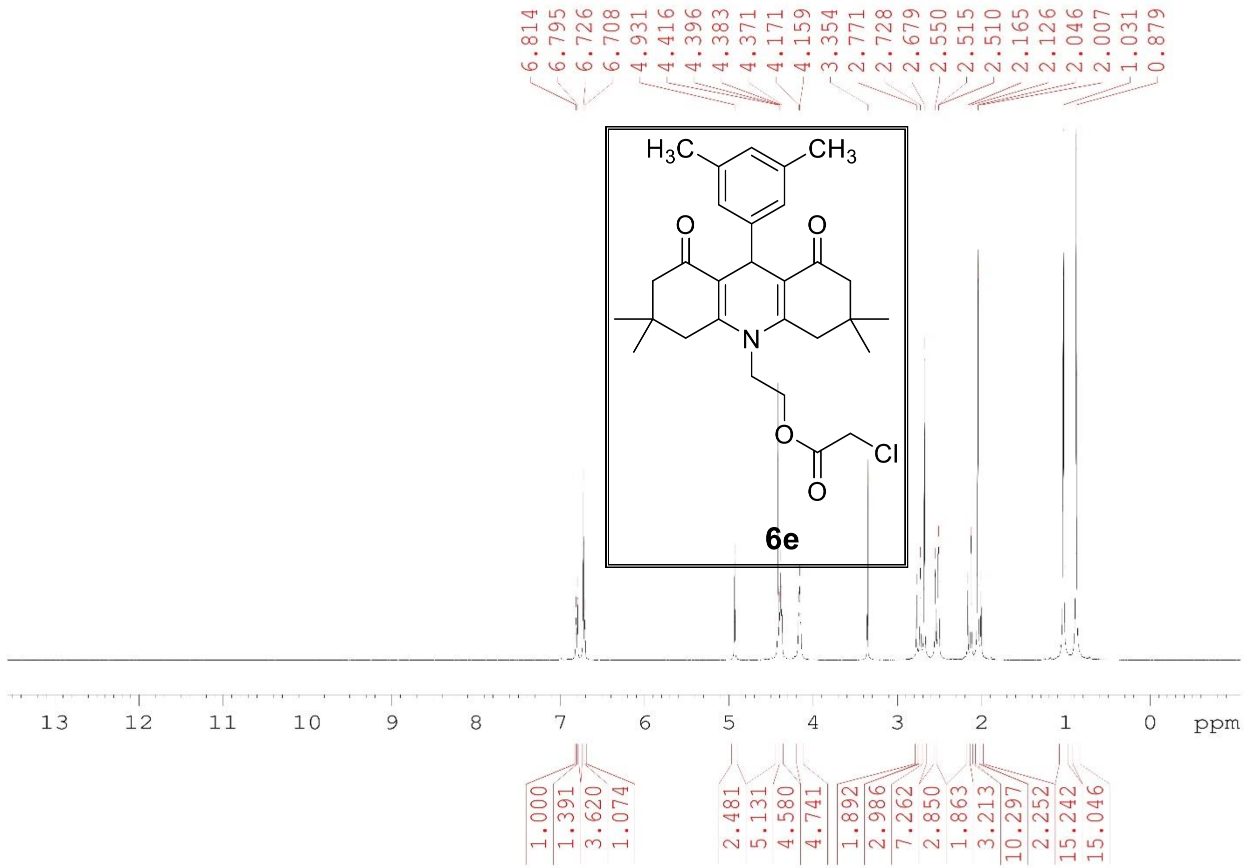

Current Data Parameters
NAME sherif fouad DC 05 -M hnmr
EXPNO 10
PROCNO 1

F2 - Acquisition Parameters
Date_ 20211220
Time 10.05 h
INSTRUM spect
PROBHD Z108618_0945 (
PULPROG zg30
TD 65536
SOLVENT DMSO
NS 16
DS 2
SWH 8012.820 Hz
FIDRES 0.244532 Hz
AQ 4.0894465 sec
RG 99.3
DW 62.400 usec
DE 6.50 usec
TE 294.4 K
D1 1.00000000 sec
TD0 1
SFO1 400.2024712 MHz
NUC1 1H
P1 13.50 usec
PLW1 13.00000000 W

F2 - Processing parameters
SI 65536
SF 400.2000000 MHz
WDW EM
SSB 0
LB 0.30 Hz
GB 0
PC 1.00

Sample Information

Acquired by	: System Administrator
Date Acquired	: 01/12/2021 02:07:11 م
Sample Type	: Unknown
Sample Name	: DC05
Sample ID	:
Dilution Factor	: 1
Tray#	: 1
Vial#	: 17
Injection Volume	: 10
Data File	: S_17.lcd
Method File	: Method_MS_only.lcm
Original Method File	: Method_MS_only.lcm
Report Format File	: DEFAULT.lsr
Tuning File	: default.lct
Processed by	: System Administrator
Date Processed	: 05/12/2021 09:42:37 ص

MS Chromatogram

Segment#1

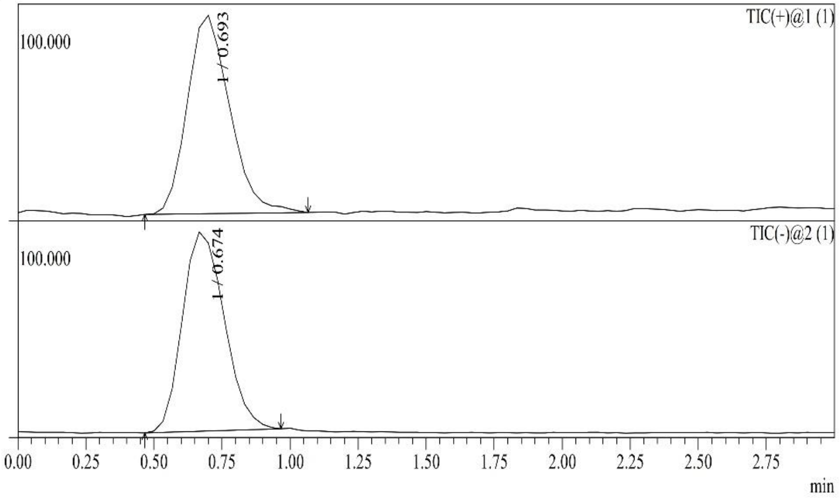

MASS Peak Table ALL MC

Peak#	Ret. Time	m/z	Area	Area%	Mark	A/H	Event#
1	0.693	TIC	20742958	100.000		10.848	1-1
2	0.674	TIC	39102276	100.000		10.735	1-2
Total			59845234	200.000			

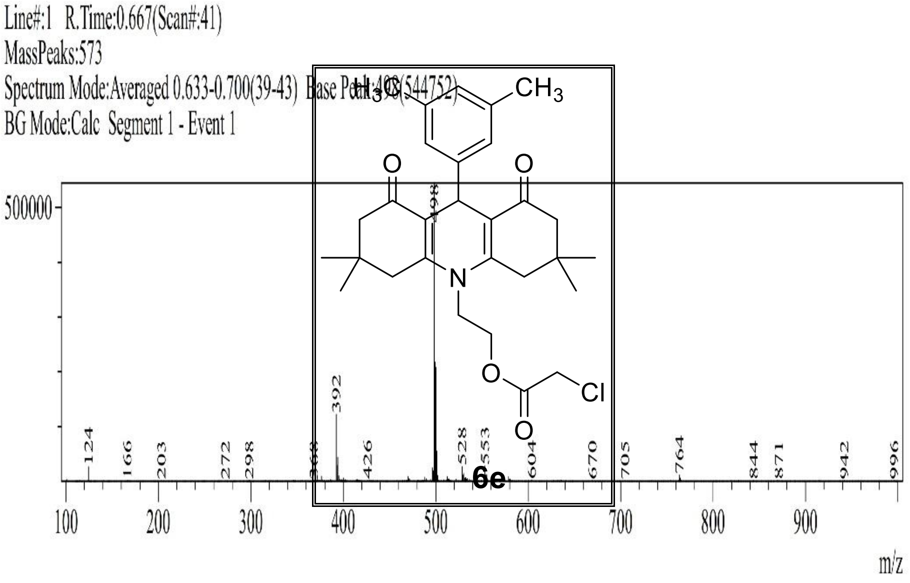

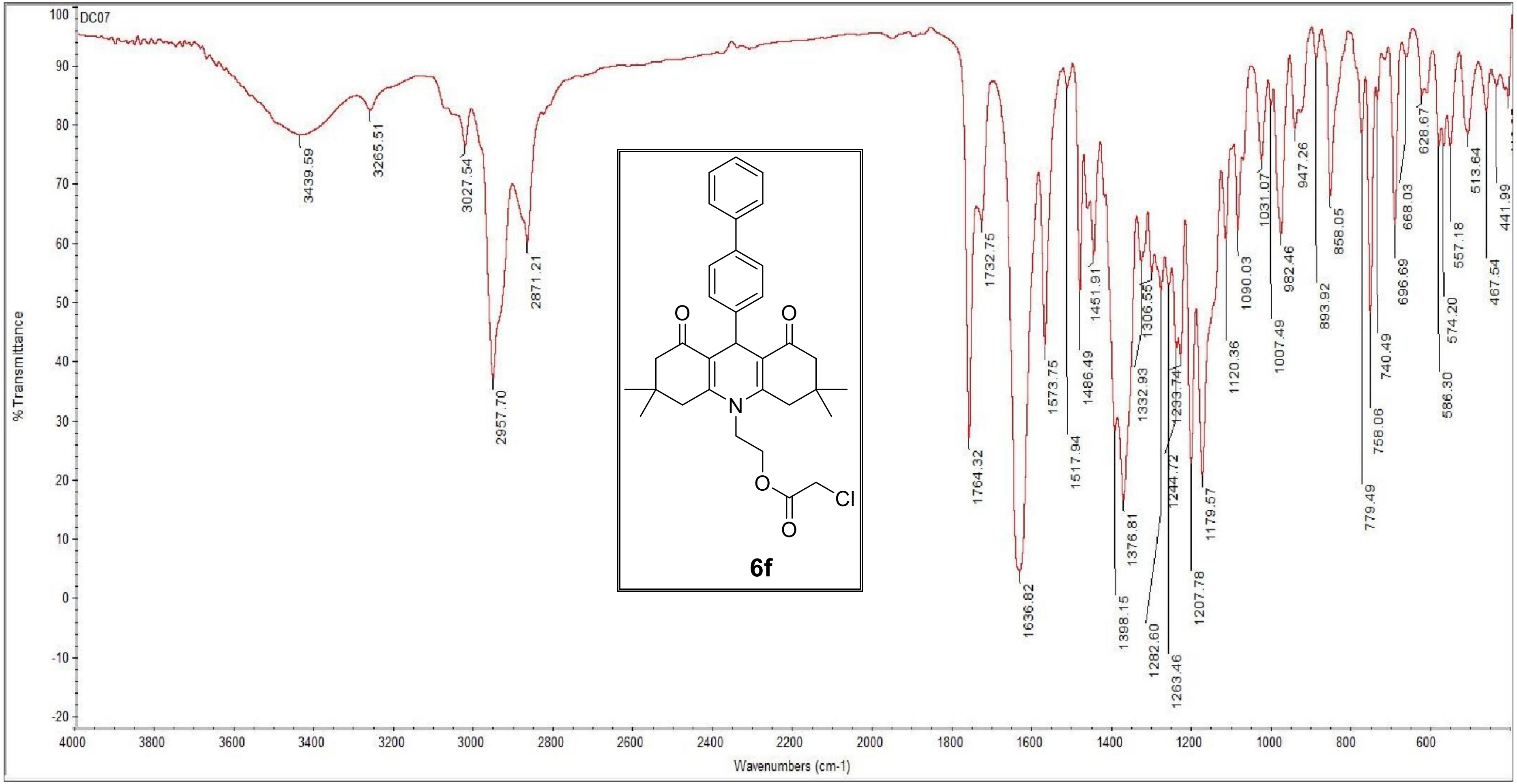

IR Spectrum of compound **6f**

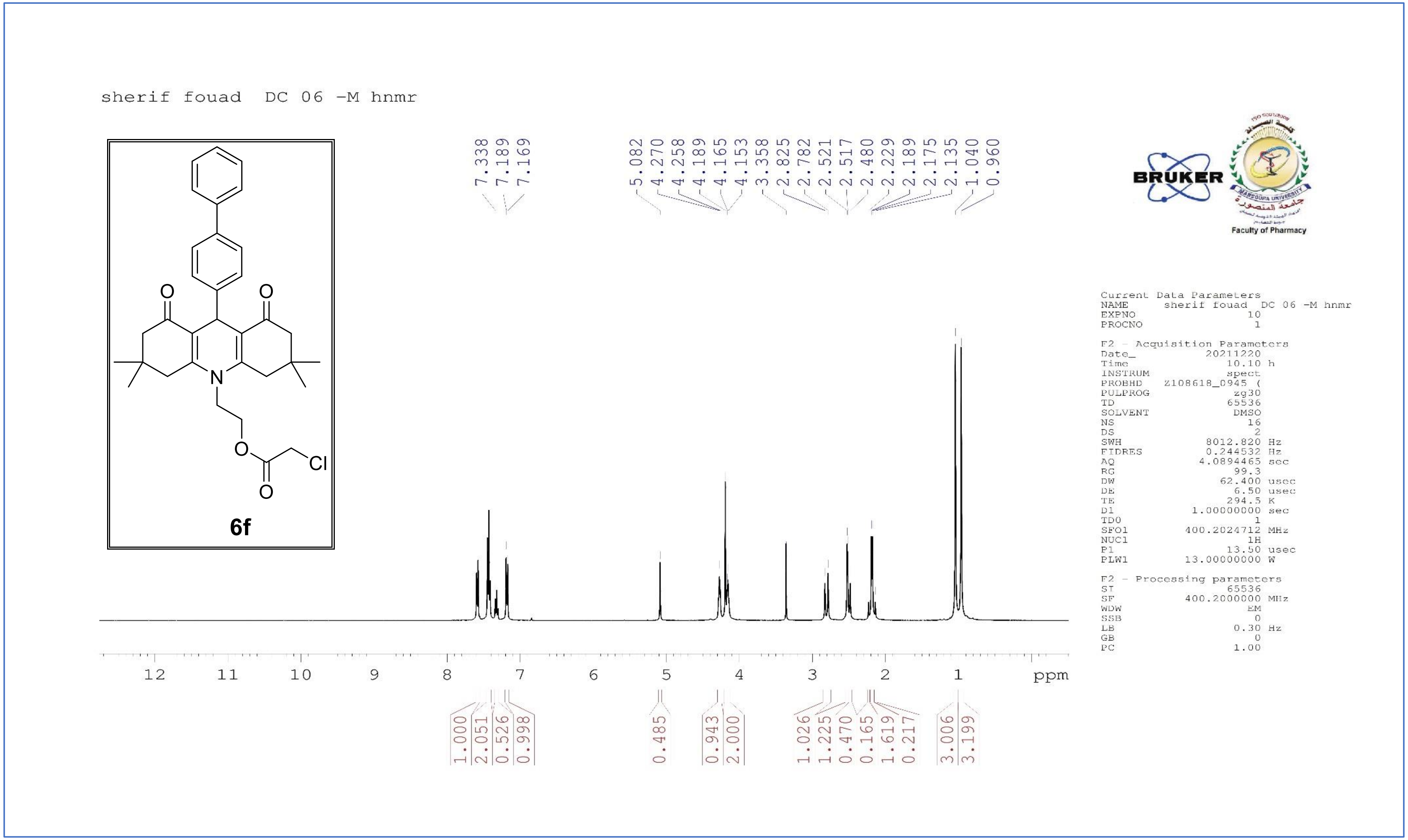

^{1}H NMR Spectrum of compound **6f**

Sample Information

Acquired by	: System Administrator
Date Acquired	: 01/12/2021 02:12:42 م
Sample Type	: Unknown
Sample Name	: DC06
Sample ID	:
Dilution Factor	: 1
Tray#	: 1
Vial#	: 18
Injection Volume	: 10
Data File	: S_18.lcd
Method File	: Method_MS_only.lcm
Original Method File	: Method_MS_only.lcm
Report Format File	: DEFAULT.lsr
Tuning File	: default.lct
Processed by	: System Administrator
Date Processed	: 05/12/2021 09:42:56 ص

MS Chromatogram

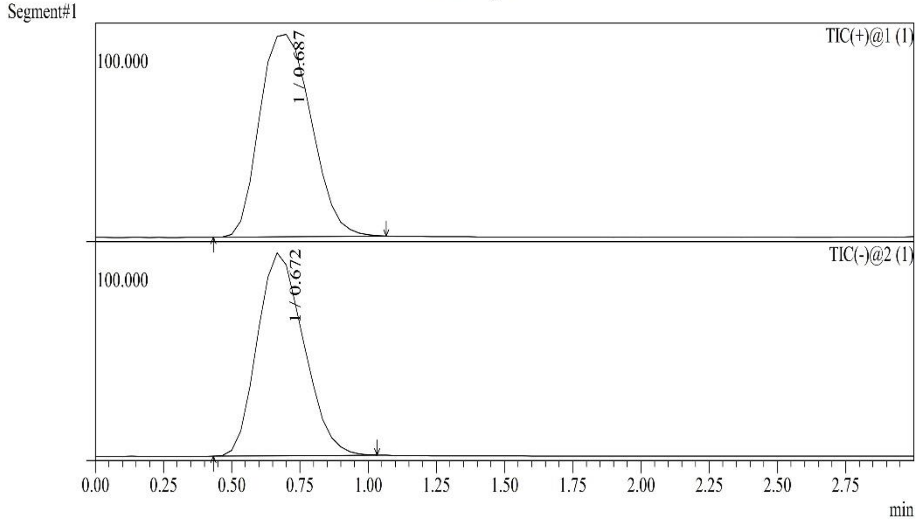

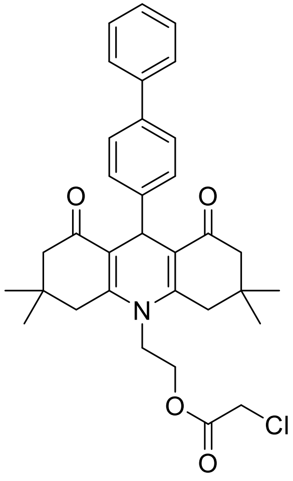

6f

Line#:1 R.Time:0.667(Scan#:41)
MassPeaks:791
Spectrum Mode:Averaged 0.633-0.700(39-43) Base Peak:546(7397153)
BG Mode:Calc Segment 1 - Event 1

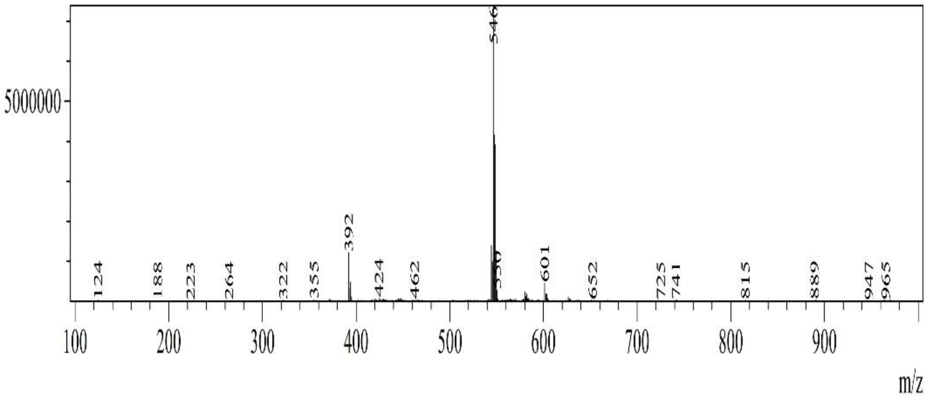

MASS Peak Table ALL MC

Peak#	Ret. Time	m/z	Area	Area%	Mark	A/H	Event#
1	0.687	TIC	363319854	100.000		13.168	1-1
2	0.672	TIC	710728098	100.000		11.849	1-2
Total			1074047952	200.000			

LC-MS Spectrum of compound **6f**

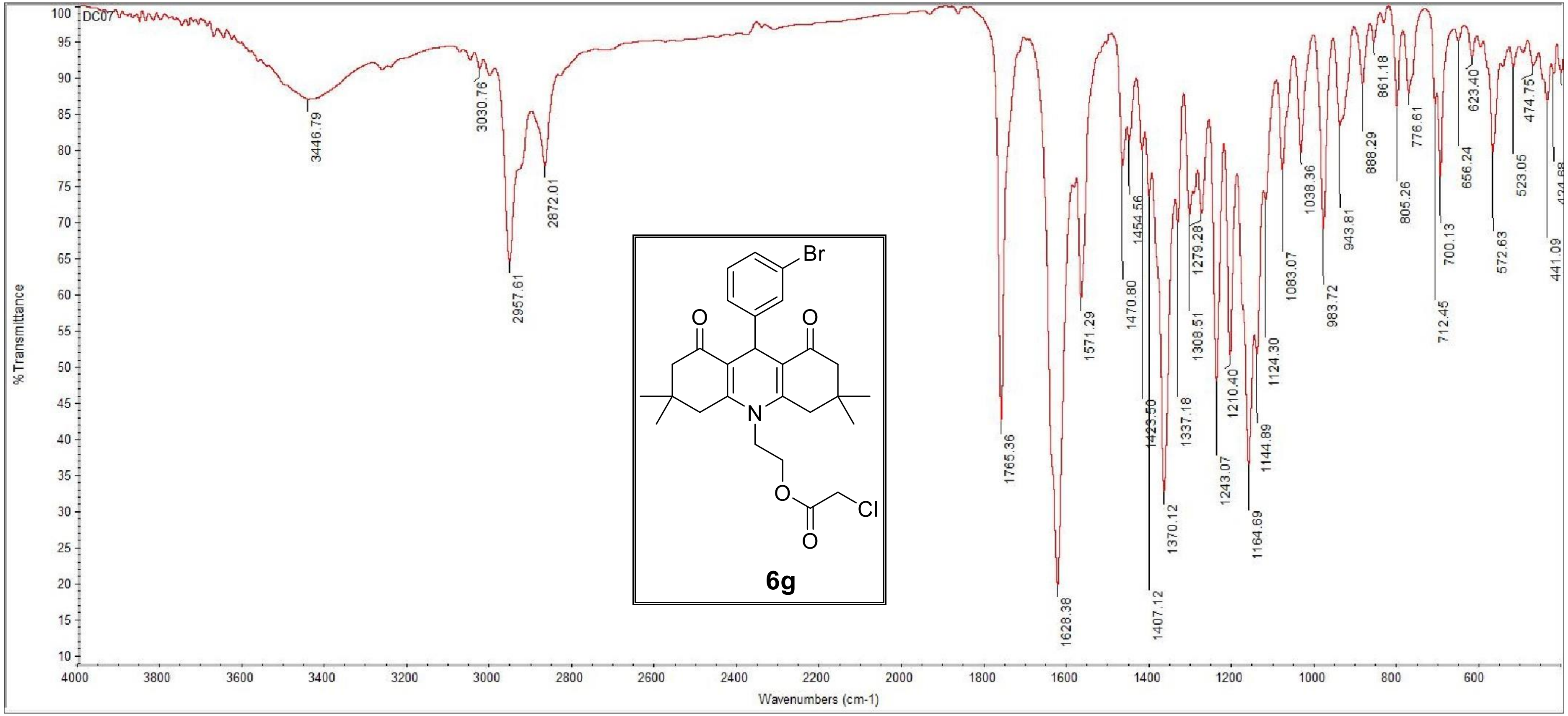
DC07
% Transmittance
Wavenumbers (cm-1)
3446.79
3030.76
2957.61
2872.01
1765.36
1628.38
1571.29
1470.80
1454.56
1423.50
1407.12
1370.12
1337.18
1308.51
1279.28
1243.07
1210.40
1164.69
1144.89
1124.30
1083.07
1038.36
983.72
943.81
888.29
861.18
805.26
776.61
712.45
700.13
656.24
623.40
572.63
523.05
474.75
441.09
Br
O
N
Cl
6g

sherif fouad DC 07 -M hnmr

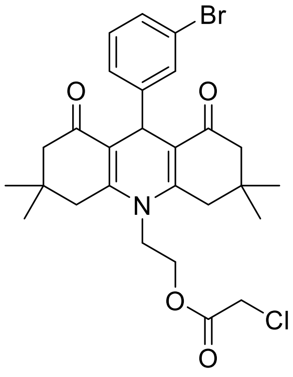

6g

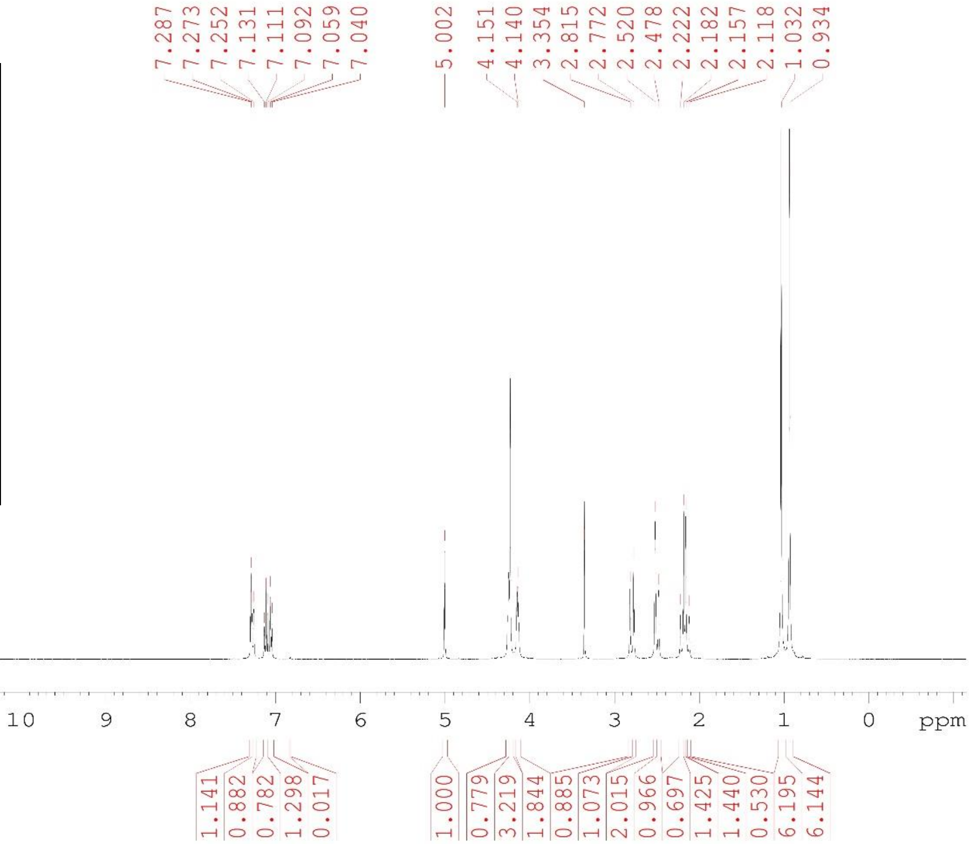

BRUKER

Faculty of Pharmacy

```
Current Data Parameters
NAME       sherif fouad  DC 07 -M hnmr
EXPNO                 10
PROCNO                 1

F2 - Acquisition Parameters
Date_           20211220
Time               10.14 h
INSTRUM            spect
PROBHD    Z108618_0945 (
PULPROG             zg30
TD                 65536
SOLVENT             DMSO
NS                    16
DS                     2
SWH             8012.820 Hz
FIDRES          0.244532 Hz
AQ             4.0894465 sec
RG                 88.92
DW                62.400 usec
DE                  6.50 usec
TE                 294.6 K
D1            1.00000000 sec
TD0                    1
SFO1         400.2024712 MHz
NUC1                  1H
P1                 13.50 usec
PLW1         13.00000000 W

F2 - Processing parameters
SI                 65536
SF           400.2000000 MHz
WDW                   EM
SSB                    0
LB                  0.30 Hz
GB                     0
PC                  1.00
```

Sample Information

Acquired by	: System Administrator
Date Acquired	: 01/12/2021 02:18:13 م
Sample Type	: Unknown
Sample Name	: DC07
Sample ID	:
Dilution Factor	: 1
Tray#	: 1
Vial#	: 19
Injection Volume	: 10
Data File	: S_19.lcd
Method File	: Method_MS_only.lcm
Original Method File	: Method_MS_only.lcm
Report Format File	: DEFAULT.lsr
Tuning File	: default.lct
Processed by	: System Administrator
Date Processed	: 05/12/2021 09:43:13 ص

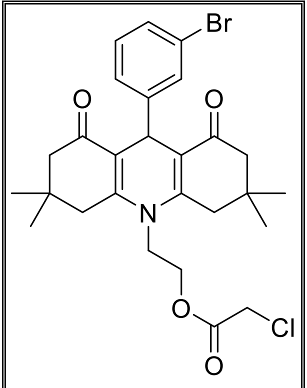

6g

MS Chromatogram

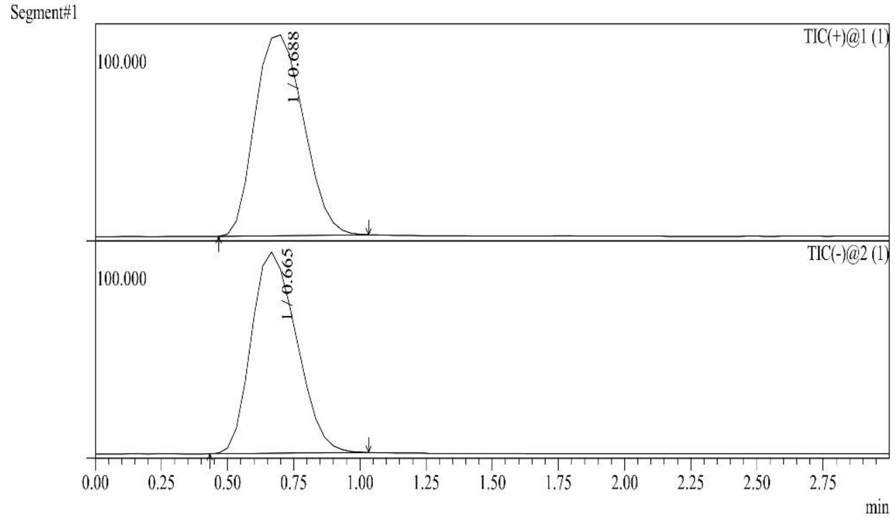

Line#:1 R.Time:0.667(Scan#:41)
MassPeaks:790
Spectrum Mode:Averaged 0.633-0.700(39-43) Base Peak:550(7057434)
BG Mode:Calc Segment 1 - Event 1

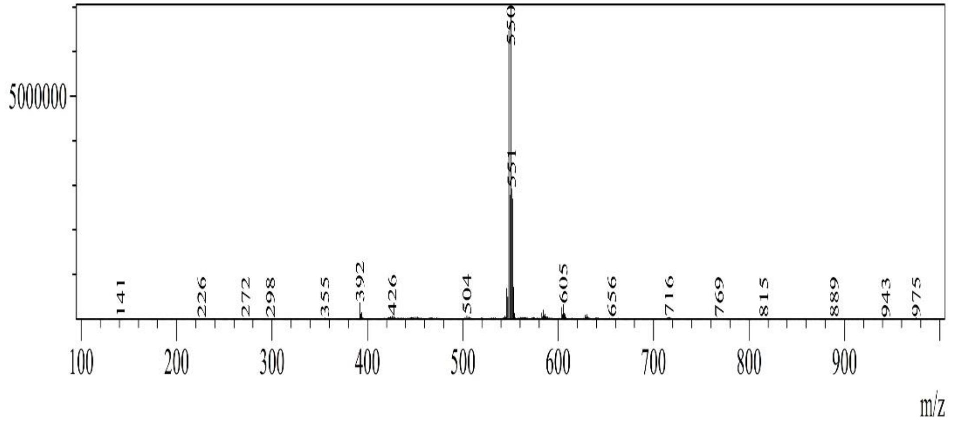

MASS Peak Table ALL MC

Peak#	Ret. Time	m/z	Area	Area%	Mark	A/H	Event#
1	0.688	TIC	388441923	100.000		12.782	1-1
2	0.665	TIC	873071410	100.000		11.905	1-2
Total			1261513333	200.000			

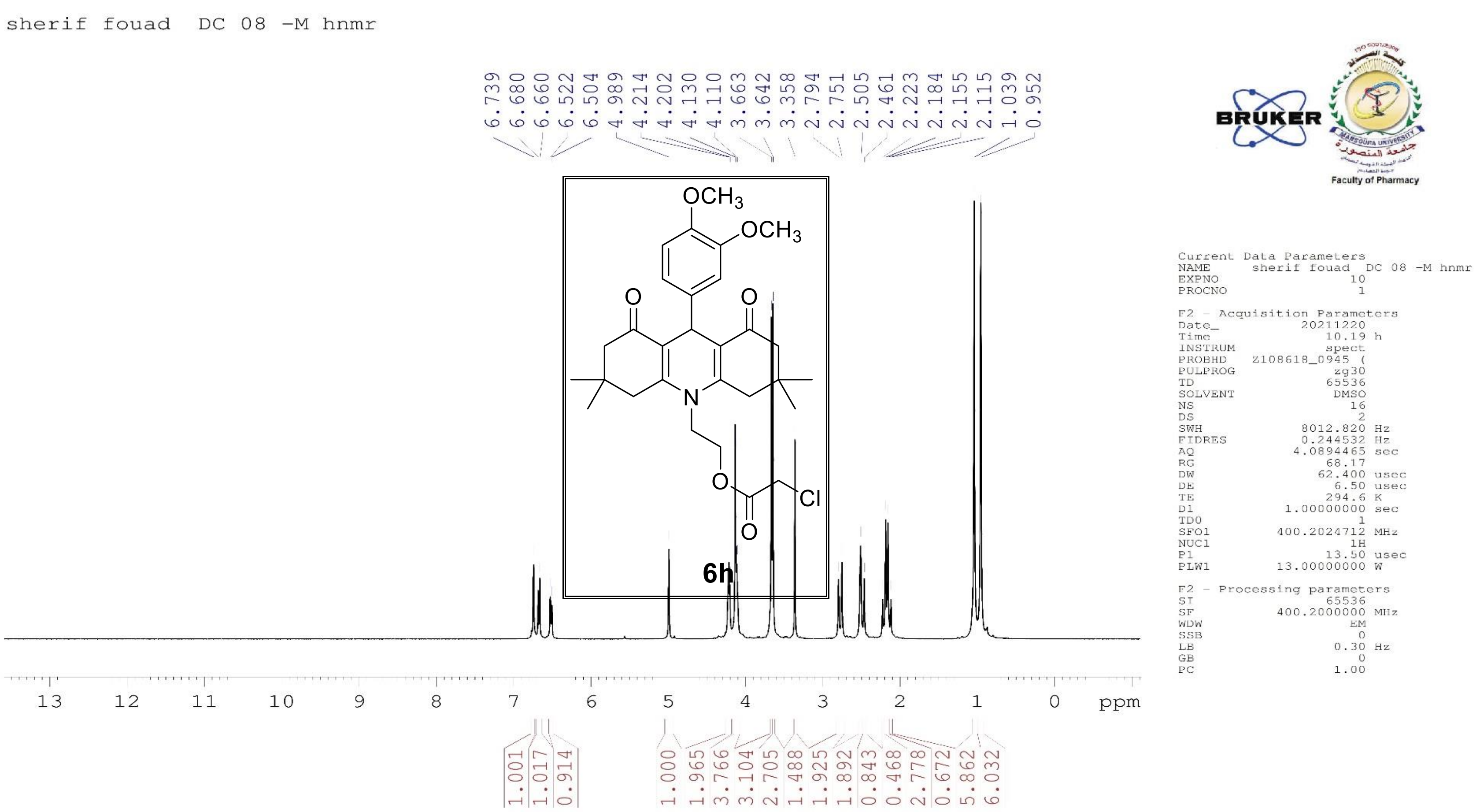
sherif fouad DC 08 -M hnmr
6.739 6.680 6.660 6.522 6.504 4.989 4.214 4.202 4.130 4.110 3.663 3.642 3.358 2.794 2.751 2.505 2.461 2.223 2.184 2.155 2.115 1.039 0.952
OCH3
OCH3
O
O
N
O
O
Cl
6h
13 12 11 10 9 8 7 6 5 4 3 2 1 0 ppm
1.001 1.017 0.914 1.000 1.965 3.766 3.104 2.705 1.488 1.925 1.892 0.843 0.468 2.778 0.672 5.862 6.032
BRUKER
Faculty of Pharmacy
Current Data Parameters
NAME sherif fouad DC 08 -M hnmr
EXPNO 10
PROCNO 1
F2 - Acquisition Parameters
Date_ 20211220
Time 10.19 h
INSTRUM spect
PROBHD Z108618_0945 (
PULPROG zg30
TD 65536
SOLVENT DMSO
NS 16
DS 2
SWH 8012.820 Hz
FIDRES 0.244532 Hz
AQ 4.0894465 sec
RG 68.17
DW 62.400 usec
DE 6.50 usec
TE 294.6 K
D1 1.00000000 sec
TD0 1
SFO1 400.2024712 MHz
NUC1 1H
P1 13.50 usec
PLW1 13.00000000 W
F2 - Processing parameters
SI 65536
SF 400.2000000 MHz
WDW EM
SSB 0
LB 0.30 Hz
GB 0
PC 1.00

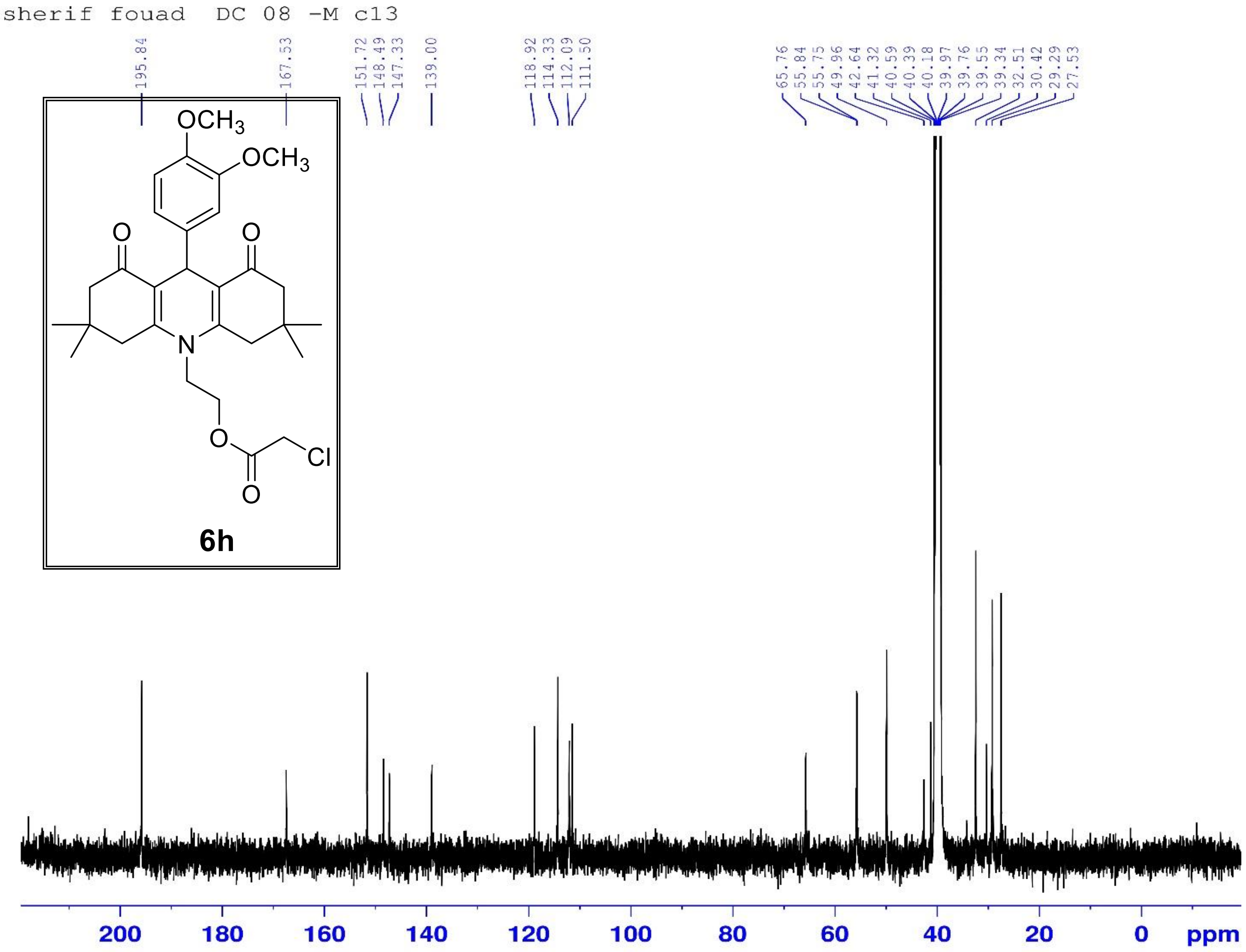

Faculty of Pharmacy

```
Current Data Parameters
NAME        sherif fouad  DC 08 -M c13
EXPNO                   10
PROCNO                   1

F2 - Acquisition Parameters
Date_             20211220
Time                 23.03 h
INSTRUM              spect
PROBHD      Z108618_0945 (
PULPROG             zgpg30
TD                   65536
SOLVENT               DMSO
NS                    2200
DS                       4
SWH              24038.461 Hz
FIDRES            0.733596 Hz
AQ               1.3631488 sec
RG                  197.77
DW                  20.800 usec
DE                    6.50 usec
TE                   294.7 K
D1              2.00000000 sec
D11             0.03000000 sec
TD0                      1
SFO1           100.6404331 MHz
NUC1                   13C
P1                   10.00 usec
PLW1           47.00000000 W
SFO2           400.2016008 MHz
NUC2                    1H
CPDPRG[2           waltz16
PCPD2                90.00 usec
PLW2           13.00000000 W
PLW12           0.29249999 W
PLW13           0.14713000 W

F2 - Processing parameters
SI                   32768
SF             100.6303700 MHz
WDW                     EM
SSB         0
LB                    1.00 Hz
GB          0
PC                    1.40
```

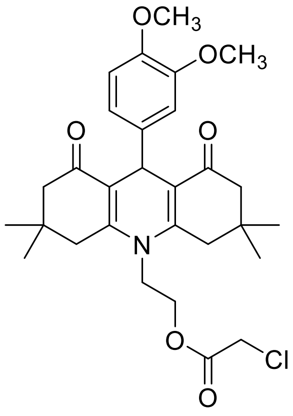

6h

Sample Information

Acquired by	: System Administrator
Date Acquired	: 01/12/2021 02:23:44 م
Sample Type	: Unknown
Sample Name	: DC08
Sample ID	:
Dilution Factor	: 1
Tray#	: 1
Vial#	: 20
Injection Volume	: 10
Data File	: S_20.lcd
Method File	: Method_MS_only.lcm
Original Method File	: Method_MS_only.lcm
Report Format File	: DEFAULT.lsr
Tuning File	: default.lct
Processed by	: System Administrator
Date Processed	: 05/12/2021 09:43:33 ص

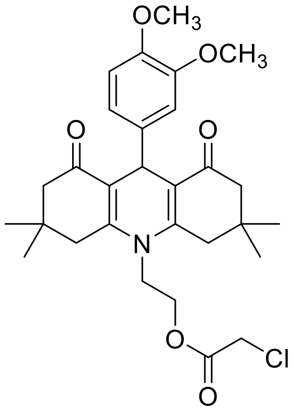

6h

MS Chromatogram

Segment#1

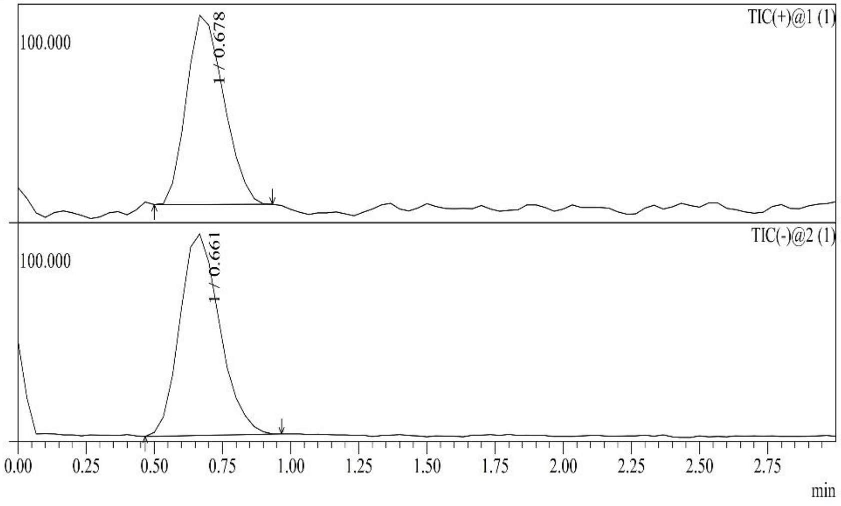

Line#:1 R.Time:0.667(Scan#:41)
MassPeaks:504
Spectrum Mode:Averaged 0.633-0.700(39-43) Base Peak:530(159918)
BG Mode:Calc Segment 1 - Event 1

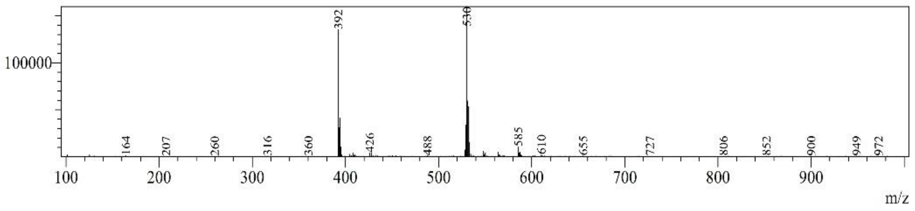

MASS Peak Table ALL MC

Peak#	Ret. Time	m/z	Area	Area%	Mark	A/H	Event#
1	0.678	TIC	6704953	100.000		9.570	1-1
2	0.661	TIC	15254379	100.000		10.240	1-2
Total			21959332	200.000			

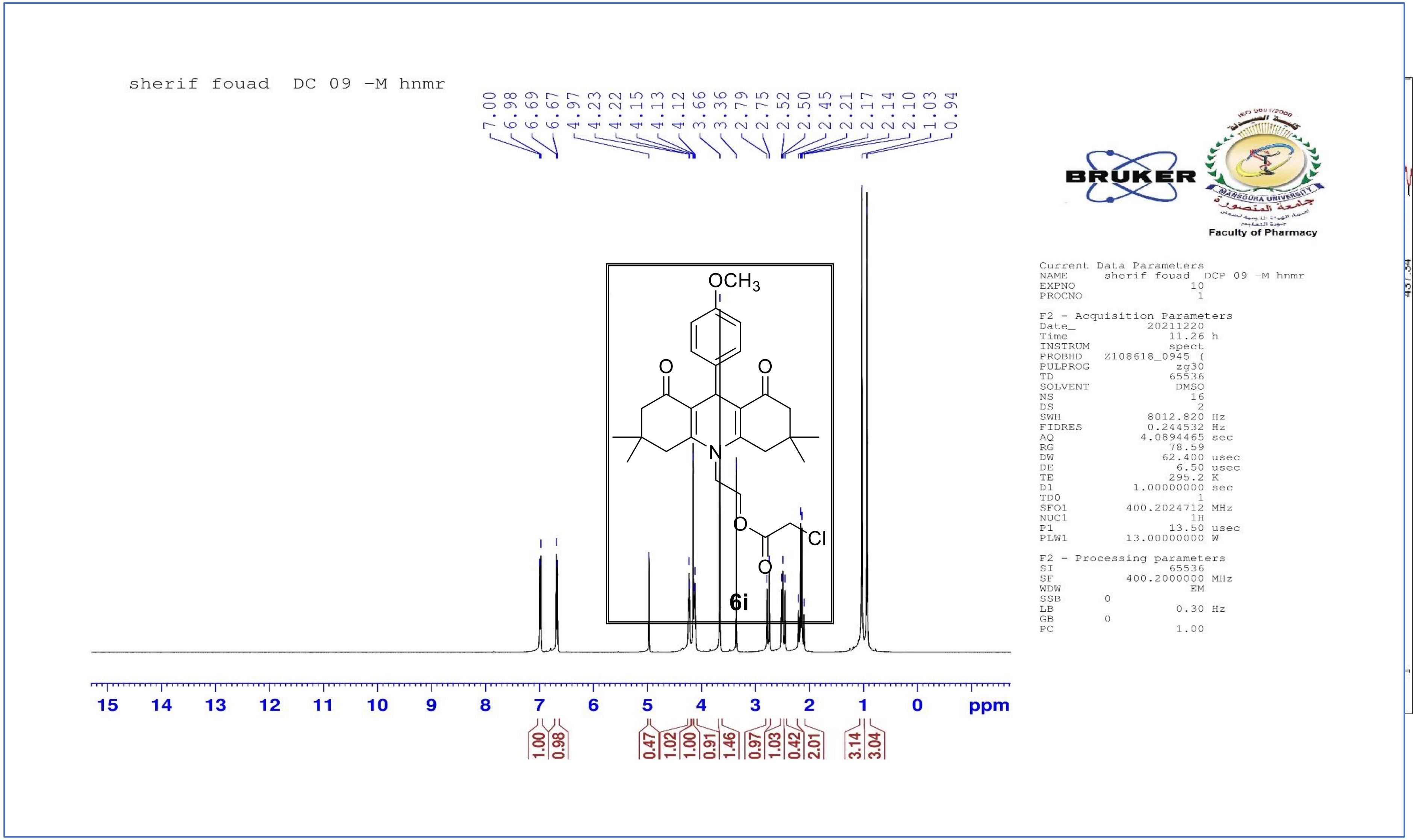

sherif fouad DC 09 -M hnmr
7.00
6.98
6.69
6.67
4.97
4.23
4.22
4.15
4.13
4.12
3.66
3.36
2.79
2.75
2.52
2.50
2.45
2.21
2.17
2.14
2.10
1.03
0.94
OCH3
O
O
N
O
Cl
O
6i
BRUKER
Faculty of Pharmacy
MANSOURA UNIVERSITY
Current Data Parameters
NAME sherif fouad DCP 09 -M hnmr
EXPNO 10
PROCNO 1
F2 - Acquisition Parameters
Date_ 20211220
Time 11.26 h
INSTRUM spect
PROBHD Z108618_0945 (
PULPROG zg30
TD 65536
SOLVENT DMSO
NS 16
DS 2
SWH 8012.820 Hz
FIDRES 0.244532 Hz
AQ 4.0894465 sec
RG 78.59
DW 62.400 usec
DE 6.50 usec
TE 295.2 K
D1 1.00000000 sec
TD0 1
SFO1 400.2024712 MHz
NUC1 1H
P1 13.50 usec
PLW1 13.00000000 W
F2 - Processing parameters
SI 65536
SF 400.2000000 MHz
WDW EM
SSB 0
LB 0.30 Hz
GB 0
PC 1.00
15 14 13 12 11 10 9 8 7 6 5 4 3 2 1 0 ppm
1.00
0.98
0.47
1.02
1.00
0.91
1.46
0.97
1.03
0.42
2.01
3.14
3.04

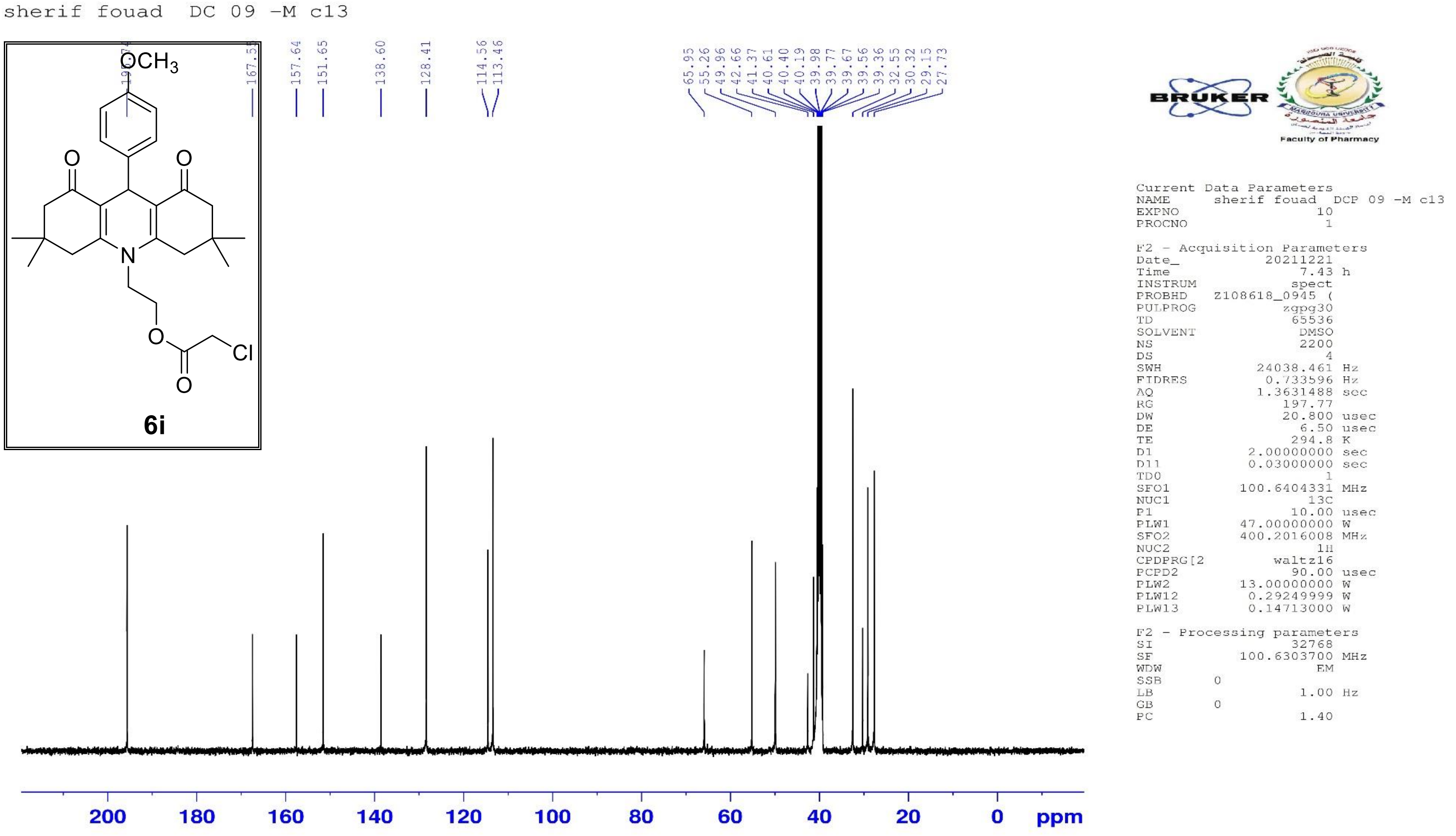

sherif fouad DC 09 -M c13
OCH3
O
O
N
O
Cl
O
6i
167.55
157.64
151.65
138.60
128.41
114.56
113.46
65.95
55.26
49.96
42.66
41.37
40.61
40.40
40.19
39.98
39.77
39.67
39.56
39.36
32.55
30.32
29.15
27.73
BRUKER
Faculty of Pharmacy
Current Data Parameters
NAME sherif fouad DCP 09 -M c13
EXPNO 10
PROCNO 1
F2 - Acquisition Parameters
Date_ 20211221
Time 7.43 h
INSTRUM spect
PROBHD Z108618_0945 (
PULPROG zgpg30
TD 65536
SOLVENT DMSO
NS 2200
DS 4
SWH 24038.461 Hz
FIDRES 0.733596 Hz
AQ 1.3631488 sec
RG 197.77
DW 20.800 usec
DE 6.50 usec
TE 294.8 K
D1 2.00000000 sec
D11 0.03000000 sec
TD0 1
SFO1 100.6404331 MHz
NUC1 13C
P1 10.00 usec
PLW1 47.00000000 W
SFO2 400.2016008 MHz
NUC2 1H
CPDPRG[2 waltz16
PCPD2 90.00 usec
PLW2 13.00000000 W
PLW12 0.29249999 W
PLW13 0.14713000 W
F2 - Processing parameters
SI 32768
SF 100.6303700 MHz
WDW EM
SSB 0
LB 1.00 Hz
GB 0
PC 1.40
200 180 160 140 120 100 80 60 40 20 0 ppm

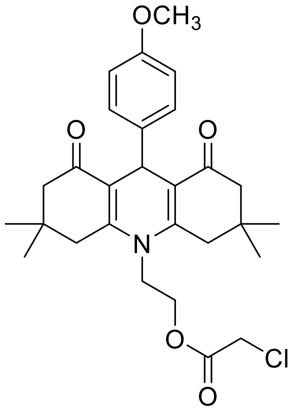

6i

Sample Information

Acquired by	: System Administrator
Date Acquired	: 01/12/2021 02:29:15 م
Sample Type	: Unknown
Sample Name	: DC09
Sample ID	:
Dilution Factor	: 1
Tray#	: 1
Vial#	: 21
Injection Volume	: 10
Data File	: S_21.lcd
Method File	: Method_MS_only.lcm
Original Method File	: Method_MS_only.lcm
Report Format File	: DEFAULT.lsr
Tuning File	: default.lct
Processed by	: System Administrator
Date Processed	: 05/12/2021 09:43:53 ص

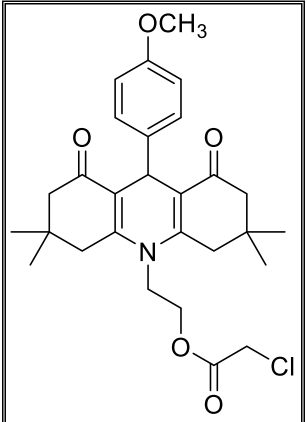

6i

MS Chromatogram

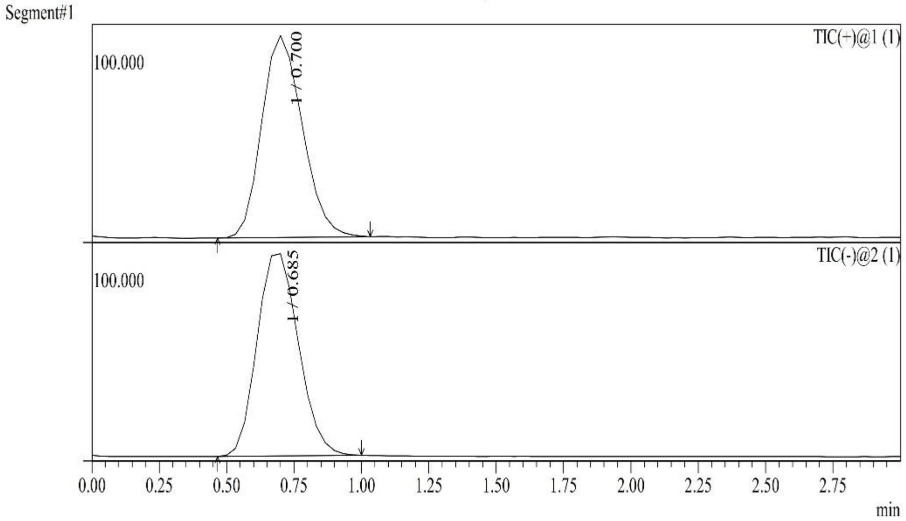

Line#:1 R.Time:0.667(Scan#:41)
MassPeaks:712
Spectrum Mode:Averaged 0.633-0.700(39-43) Base Peak:500(2380906)
BG Mode:Calc Segment 1 - Event 1

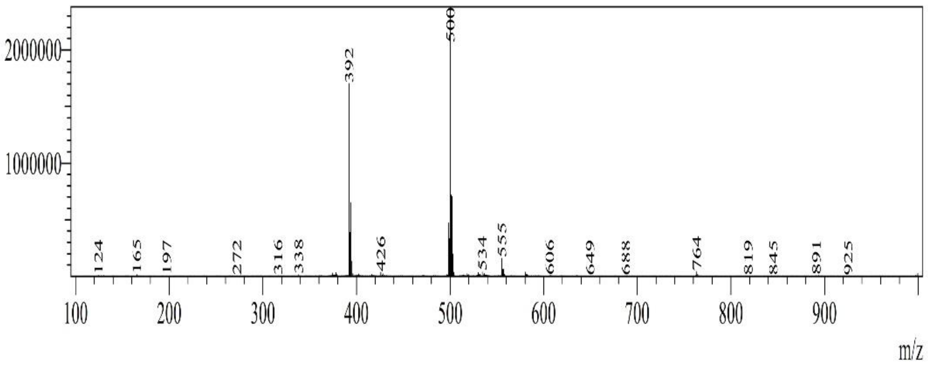

MASS Peak Table ALL MC

Peak#	Ret. Time	m/z	Area	Area%	Mark	A/H	Event#
1	0.700	TIC	115796959	100.000		10.484	1-1
2	0.685	TIC	224239102	100.000		10.727	1-2
Total			340036061	200.000			

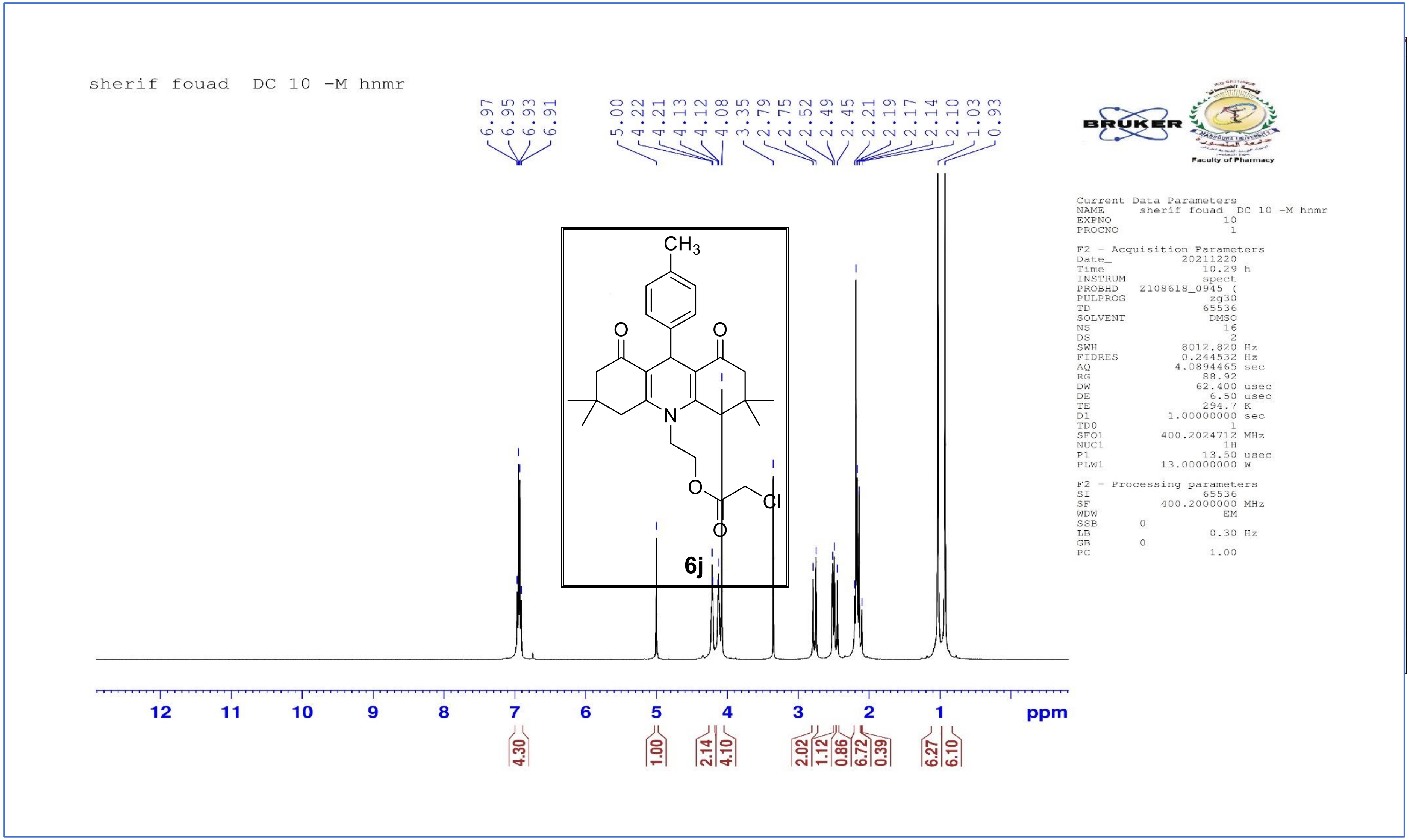
sherif fouad DC 10 -M hnmr
6.97 6.95 6.93 6.91 5.00 4.22 4.21 4.13 4.12 4.08 3.35 2.79 2.75 2.52 2.49 2.45 2.21 2.19 2.17 2.14 2.10 1.03 0.93
6j
Current Data Parameters
NAME sherif fouad DC 10 -M hnmr
EXPNO 10
PROCNO 1
F2 - Acquisition Parameters
Date_ 20211220
Time 10.29 h
INSTRUM spect
PULPROG zg30
TD 65536
SOLVENT DMSO
NS 16
DS 2
SWH 8012.820 Hz
FIDRES 0.244532 Hz
AQ 4.0894465 sec
RG 88.92
DW 62.400 usec
DE 6.50 usec
TE 294.7 K
D1 1.00000000 sec
TD0 1
SFO1 400.2024712 MHz
NUC1 1H
P1 13.50 usec
PLW1 13.00000000 W
F2 - Processing parameters
SI 65536
SF 400.2000000 MHz
WDW EM
SSB 0
LB 0.30 Hz
GB 0
PC 1.00
ppm
4.30 1.00 2.14 4.10 2.02 1.12 0.86 6.72 0.39 6.27 6.10

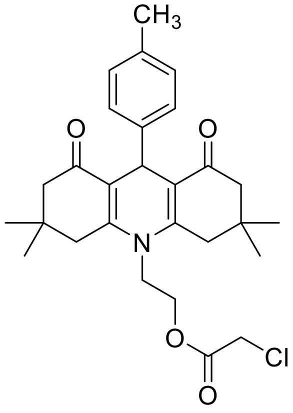

6j

Sample Information

Acquired by	: System Administrator
Date Acquired	: 01/12/2021 02:34:53 م
Sample Type	: Unknown
Sample Name	: DC10
Sample ID	:
Dilution Factor	: 1
Tray#	: 1
Vial#	: 22
Injection Volume	: 10
Data File	: S_22.lcd
Method File	: Method_MS_only.lcm
Original Method File	: Method_MS_only.lcm
Report Format File	: DEFAULT.lsr
Tuning File	: default.lct
Processed by	: System Administrator
Date Processed	: 05/12/2021 09:44:17 ص

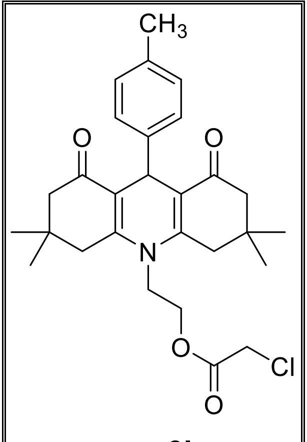
6j

MS Chromatogram

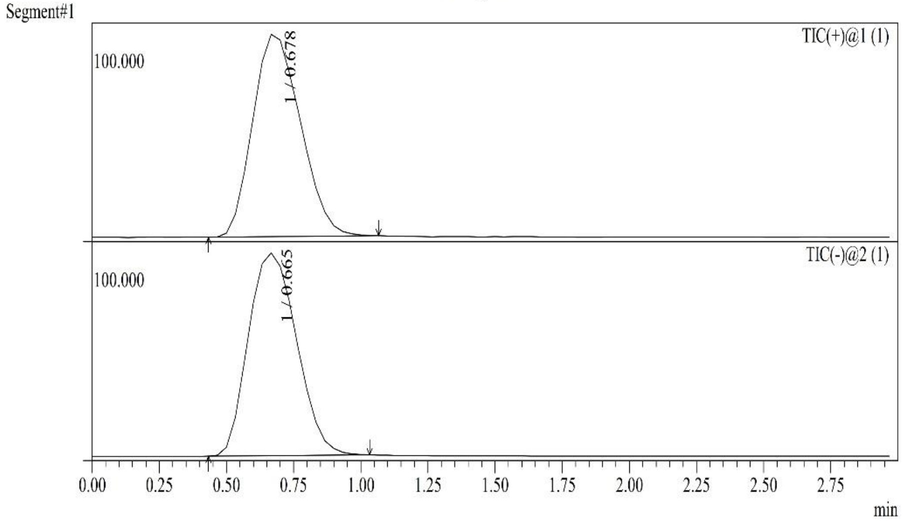

Line#:1 R.Time:0.667(Scan#:41)
MassPeaks:751
Spectrum Mode:Averaged 0.633-0.700(39-43) Base Peak:484(8063666)
BG Mode:Calc Segment 1 - Event 1

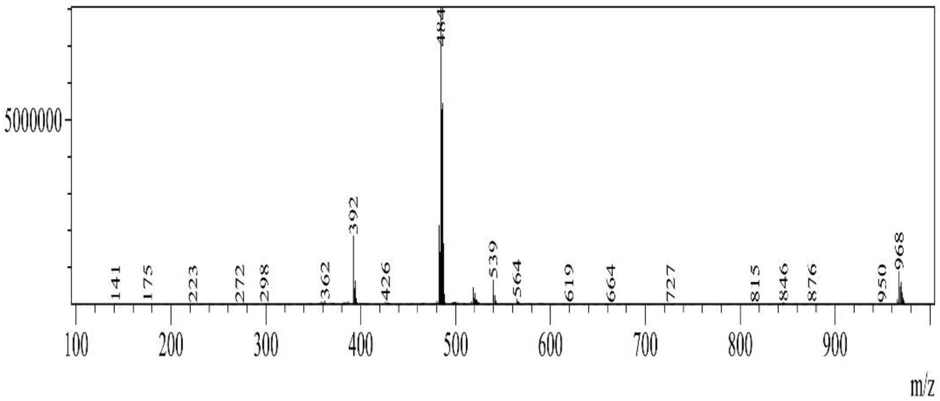

MASS Peak Table ALL MC

Peak#	Ret. Time	m/z	Area	Area%	Mark	A/H	Event#
1	0.678	TIC	460152311	100.000		12.377	1-1
2	0.665	TIC	860282320	100.000		12.513	1-2
Total			1320434631	200.000			

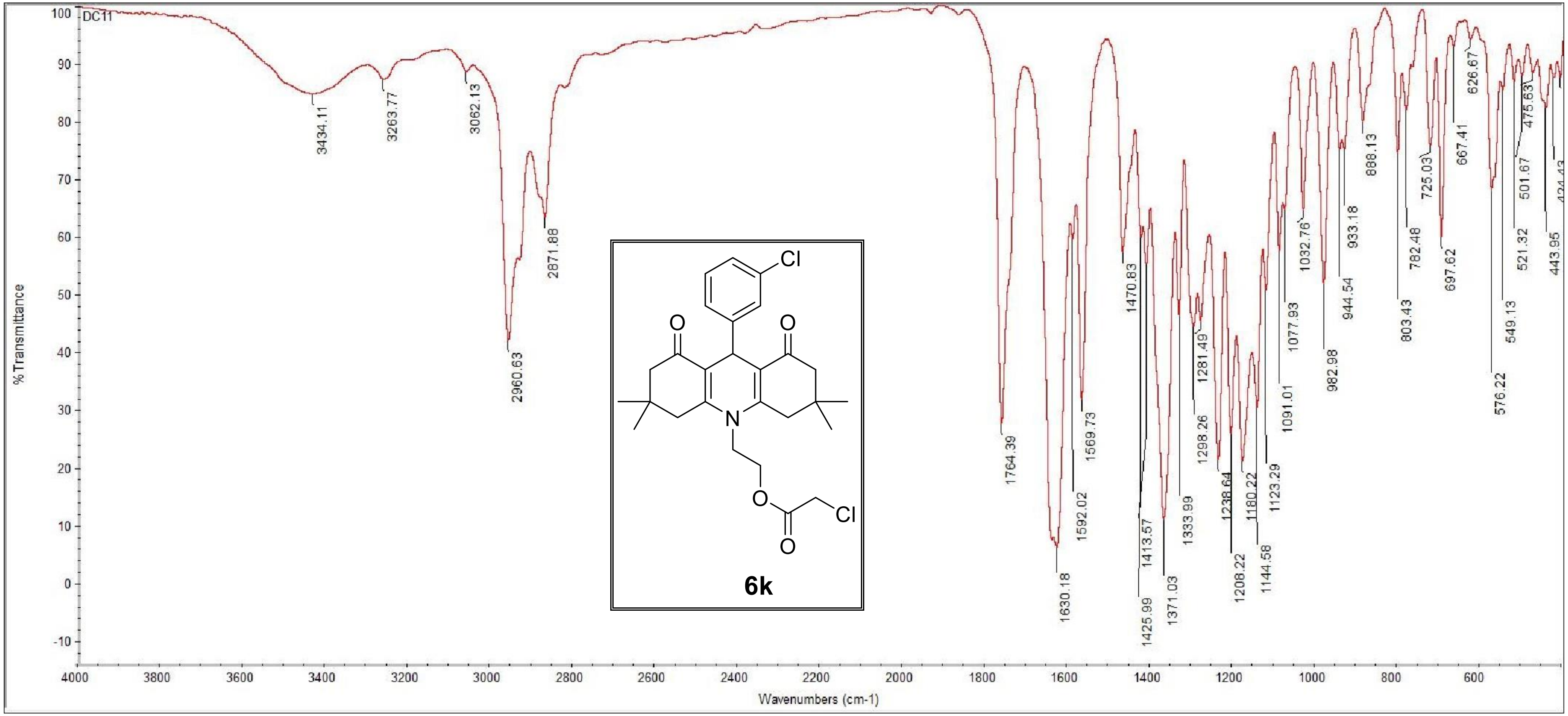
DC11
% Transmittance
Wavenumbers (cm-1)
3434.11
3263.77
3062.13
2960.63
2871.88
1764.39
1630.18
1592.02
1569.73
1470.83
1425.99
1413.57
1371.03
1333.99
1298.26
1281.49
1238.64
1208.22
1180.22
1144.58
1123.29
1091.01
1077.93
1032.76
982.98
944.54
933.18
888.13
803.43
782.48
725.03
697.62
667.41
626.67
576.22
549.13
521.32
501.67
475.63
443.95
Cl
O
O
N
O
O
Cl
6k

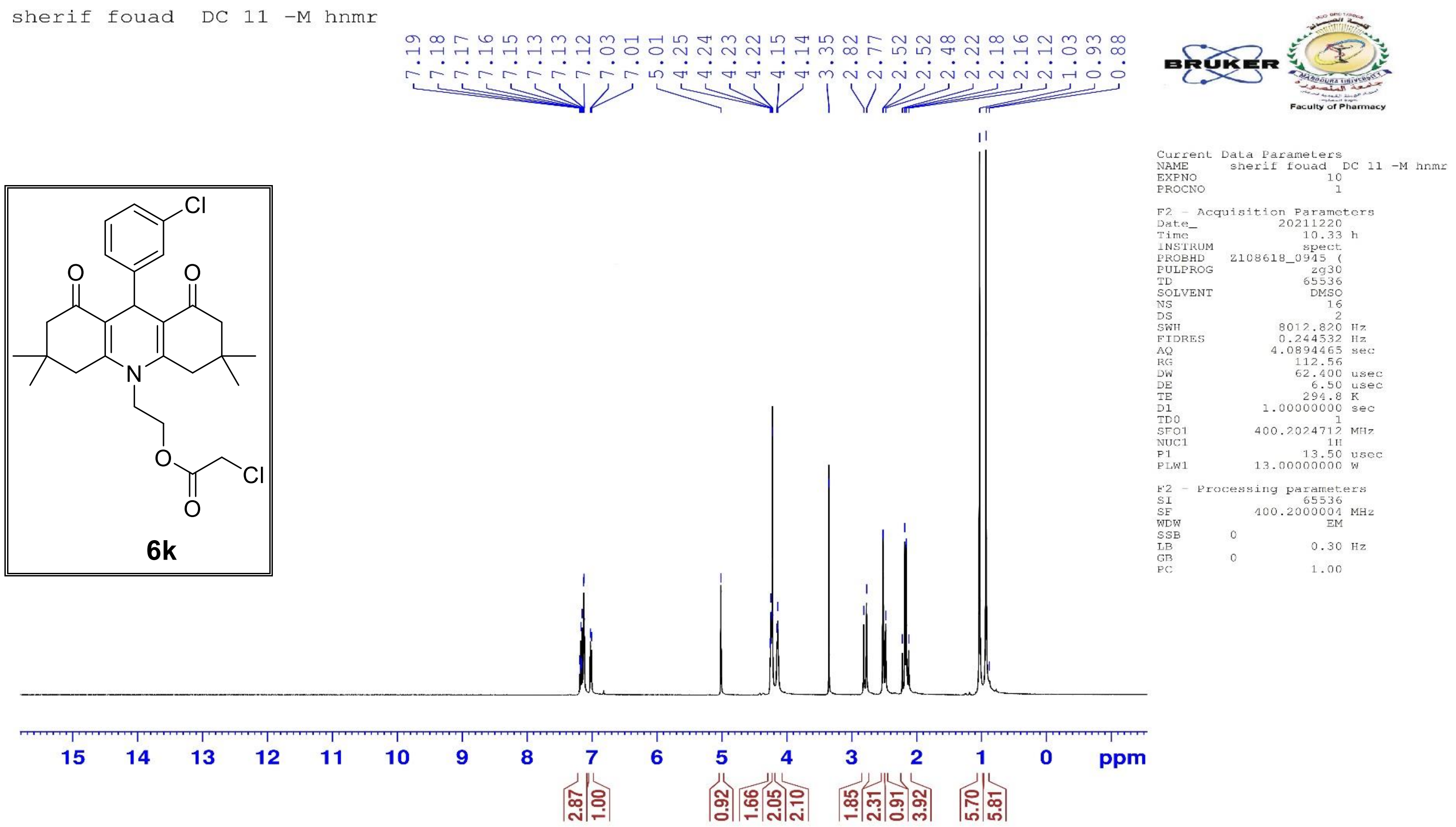
sherif fouad DC 11 -M hnmr
7.19 7.18 7.17 7.16 7.15 7.13 7.13 7.12 7.03 7.01 5.01 4.25 4.24 4.23 4.22 4.15 4.14 3.35 2.82 2.77 2.52 2.52 2.48 2.22 2.18 2.16 2.12 1.03 0.93 0.88
BRUKER
Faculty of Pharmacy
Current Data Parameters
NAME sherif fouad DC 11 -M hnmr
EXPNO 10
PROCNO 1
F2 - Acquisition Parameters
Date_ 20211220
Time 10.33 h
INSTRUM spect
PROBHD Z108618_0945 (
PULPROG zg30
TD 65536
SOLVENT DMSO
NS 16
DS 2
SWH 8012.820 Hz
FIDRES 0.244532 Hz
AQ 4.0894465 sec
RG 112.56
DW 62.400 usec
DE 6.50 usec
TE 294.8 K
D1 1.00000000 sec
TD0 1
SFO1 400.2024712 MHz
NUC1 1H
P1 13.50 usec
PLW1 13.00000000 W
F2 - Processing parameters
SI 65536
SF 400.2000004 MHz
WDW EM
SSB 0
LB 0.30 Hz
GB 0
PC 1.00
Cl
O
O
N
O
O
Cl
6k
15 14 13 12 11 10 9 8 7 6 5 4 3 2 1 0 ppm
2.87 1.00 0.92 1.66 2.05 2.10 1.85 2.31 0.91 3.92 5.70 5.81

Sample Information

Acquired by	: System Administrator
Date Acquired	: 01/12/2021 02:38:24 م
Sample Type	: Unknown
Sample Name	: DC11
Sample ID	:
Dilution Factor	: 1
Tray#	: 1
Vial#	: 23
Injection Volume	: 10
Data File	: S_23.lcd
Method File	: Method_MS_only.lcm
Original Method File	: Method_MS_only.lcm
Report Format File	: DEFAULT.lsr
Tuning File	: default.lct
Processed by	: System Administrator
Date Processed	: 05/12/2021 09:45:29 ص

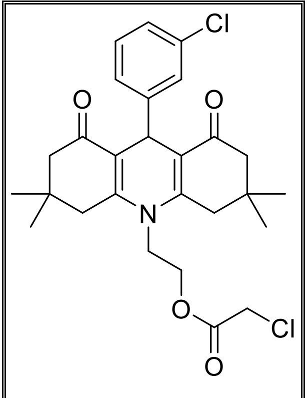

6k

MS Chromatogram

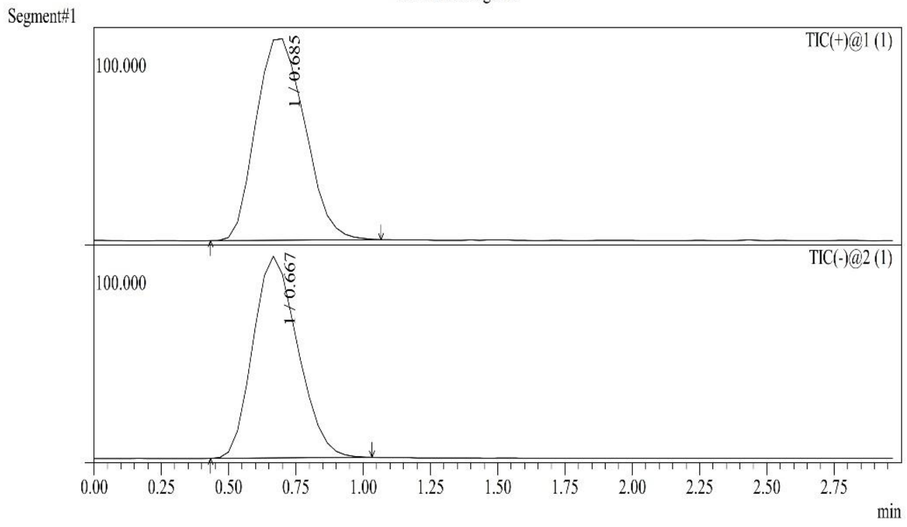

Line#:1 R.Time:0.667(Scan#:41)
MassPeaks:791
Spectrum Mode:Averaged 0.633-0.700(39-43) Base Peak:504(7177175)
BG Mode:Calc Segment 1 - Event 1

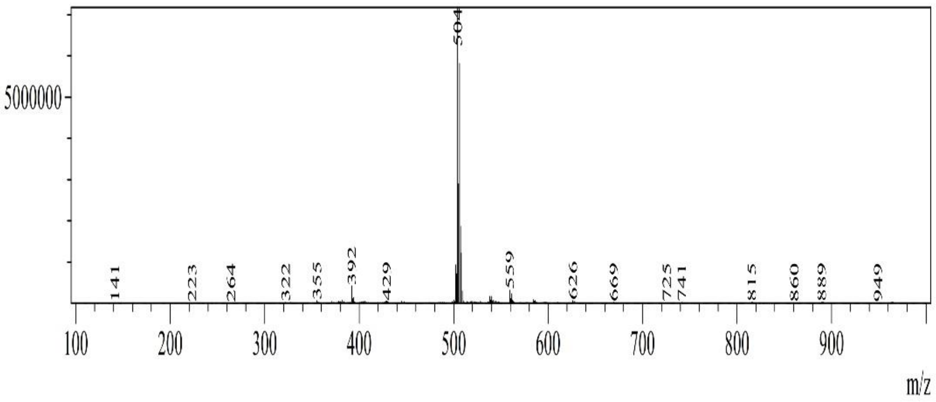

MASS Peak Table ALL MC

Peak#	Ret. Time	m/z	Area	Area%	Mark	A/H	Event#
1	0.685	TIC	346999723	100.000		12.662	1-1
2	0.667	TIC	715646784	100.000		11.567	1-2
Total			1062646507	200.000			

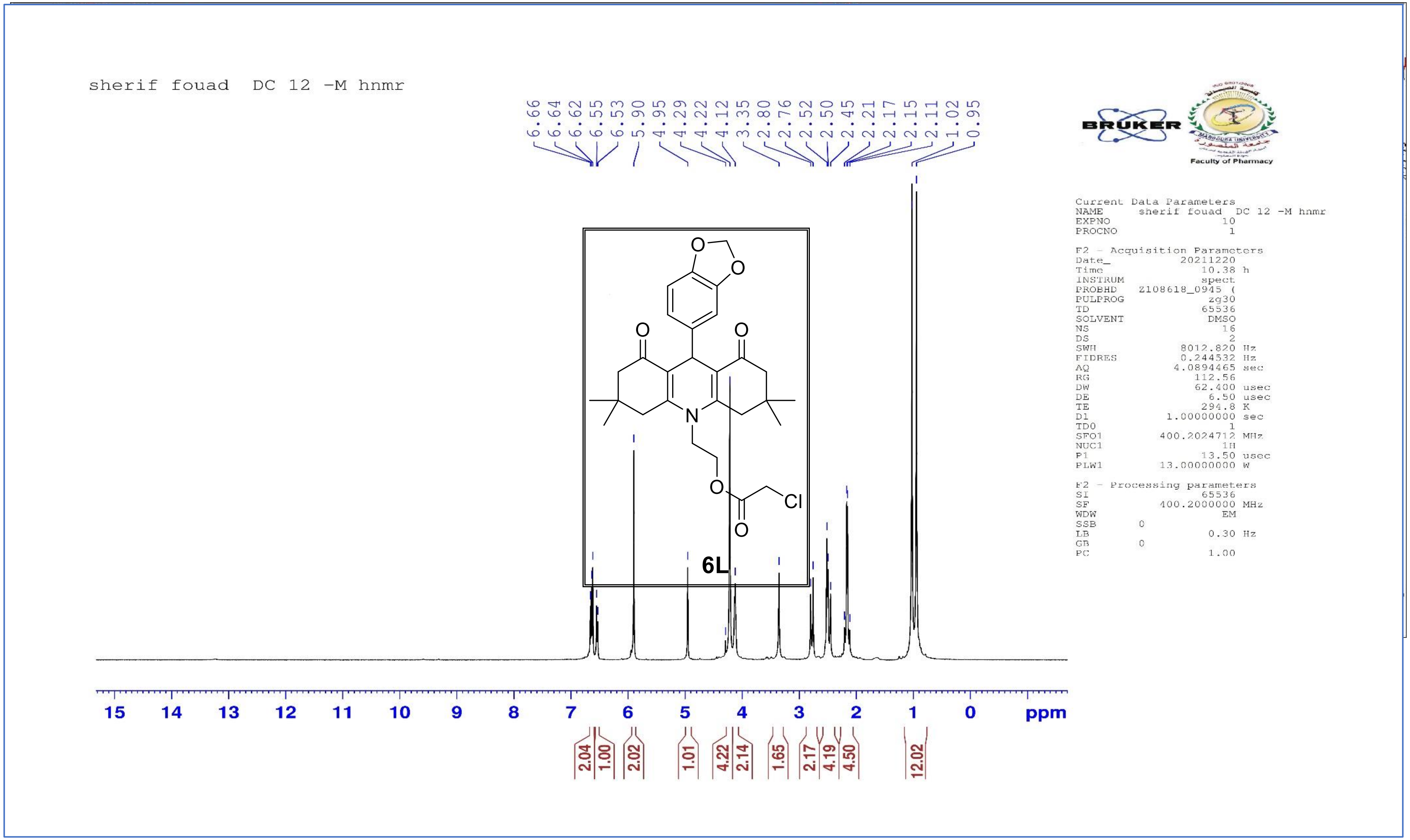
sherif fouad DC 12 -M hnmr
6.66
6.64
6.62
6.55
6.53
5.90
4.95
4.29
4.22
4.12
3.35
2.80
2.76
2.52
2.50
2.45
2.21
2.17
2.15
2.11
1.02
0.95
BRUKER
Faculty of Pharmacy
Current Data Parameters
NAME sherif fouad DC 12 -M hnmr
EXPNO 10
PROCNO 1
F2 - Acquisition Parameters
Date_ 20211220
Time 10.38 h
INSTRUM spect
PROBHD Z108618_0945 (
PULPROG zg30
TD 65536
SOLVENT DMSO
NS 16
DS 2
SWH 8012.820 Hz
FIDRES 0.244532 Hz
AQ 4.0894465 sec
RG 112.56
DW 62.400 usec
DE 6.50 usec
TE 294.8 K
D1 1.00000000 sec
TD0 1
SFO1 400.2024712 MHz
NUC1 1H
P1 13.50 usec
PLW1 13.00000000 W
F2 - Processing parameters
SI 65536
SF 400.2000000 MHz
WDW EM
SSB 0
LB 0.30 Hz
GB 0
PC 1.00
O
O
O
O
N
O
O
Cl
O
6L
15
14
13
12
11
10
9
8
7
6
5
4
3
2
1
0
ppm
2.04
1.00
2.02
1.01
4.22
2.14
1.65
2.17
4.19
4.50
12.02

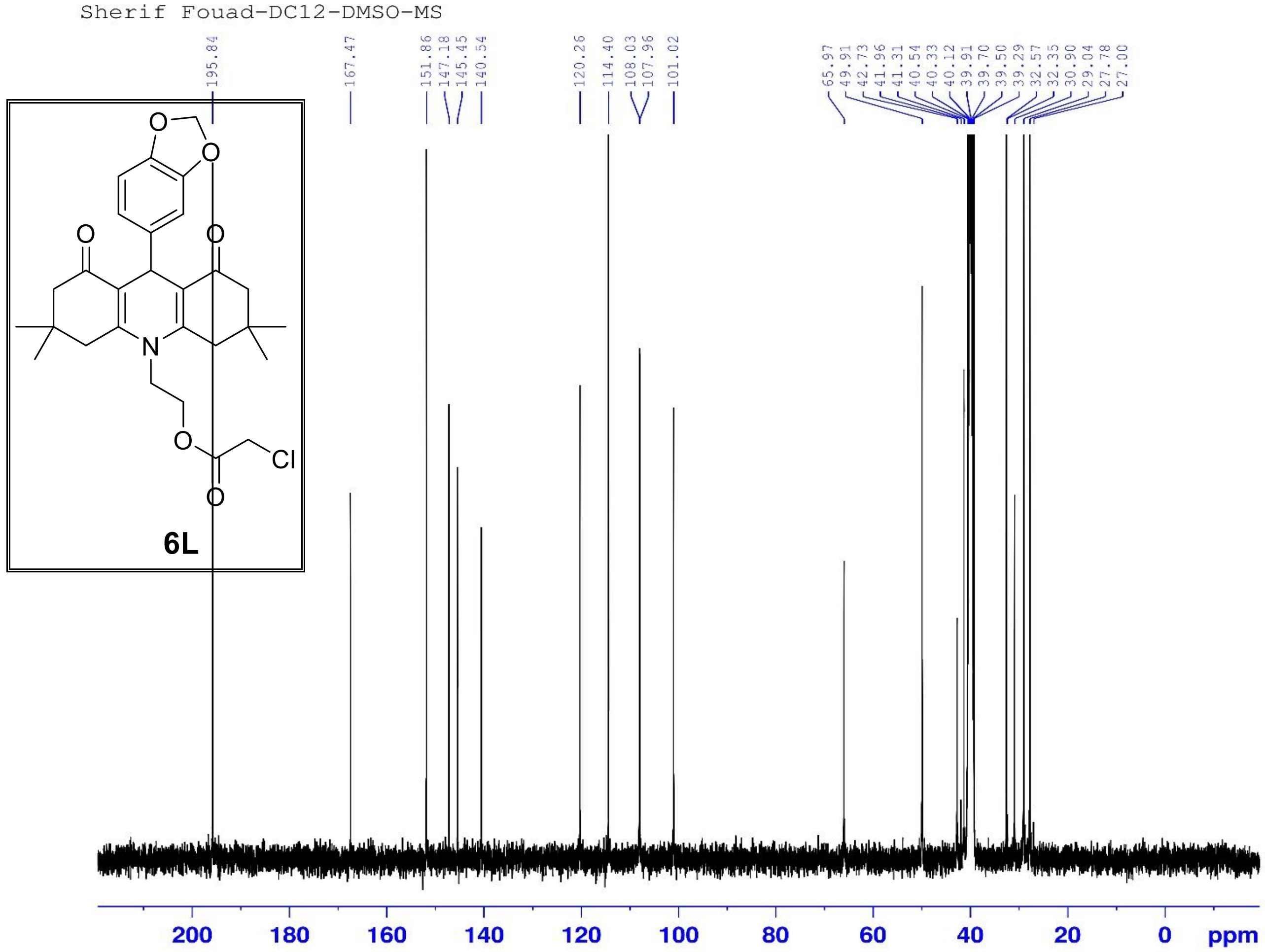


```
Current Data Parameters
NAME      Sherif Fouad-DC12-DMSO-MS
EXPNO                    10
PROCNO                    1

F2 - Acquisition Parameters
Date_              20220107
Time                   5.04 h
INSTRUM               spect
PROBHD    Z108618_0945 (
PULPROG              zgpg30
TD                    65536
SOLVENT                DMSO
NS                     2200
DS                        4
SWH               24038.461 Hz
FIDRES             0.733596 Hz
AQ                1.3631488 sec
RG                   197.77
DW                   20.800 usec
DE                     6.50 usec
TE                    296.6 K
D1               2.00000000 sec
D11              0.03000000 sec
TD0                       1
SFO1            100.6404331 MHz
NUC1                    13C
P1                    10.00 usec
PLW1            47.00000000 W
SFO2            400.2016008 MHz
NUC2                     1H
CPDPRG[2            waltz16
PCPD2                 90.00 usec
PLW2            13.00000000 W
PLW12            0.29249999 W
PLW13            0.14713000 W

F2 - Processing parameters
SI                    32768
SF              100.6303700 MHz
WDW                      EM
SSB      0
LB                     1.00 Hz
GB       0
PC                     1.40
```

Faculty of Pharmacy

Sample Information

Acquired by	: System Administrator
Date Acquired	: 01/12/2021 02:41:54 م
Sample Type	: Unknown
Sample Name	: DC12
Sample ID	:
Dilution Factor	: 1
Tray#	: 1
Vial#	: 24
Injection Volume	: 10
Data File	: S_24.lcd
Method File	: Method_MS_only.lcm
Original Method File	: Method_MS_only.lcm
Report Format File	: DEFAULT.lsr
Tuning File	: default.lct
Processed by	: System Administrator
Date Processed	: 05/12/2021 09:45:48 ص

MS Chromatogram

Segment#1

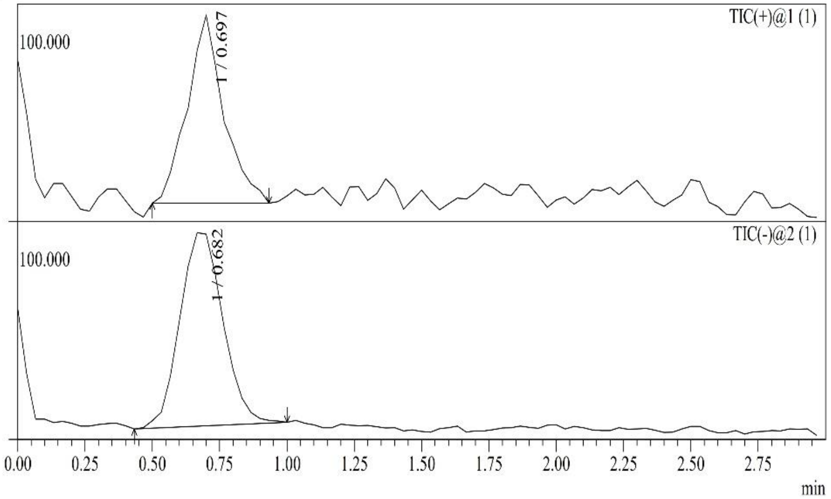

MASS Peak Table ALL MC

Peak#	Ret. Time	m/z	Area	Area%	Mark	A/H	Event#
1	0.697	TIC	2662750	100.000		9.425	1-1
2	0.682	TIC	4759751	100.000		11.168	1-2
Total			7422501	200.000			

Line#:1 R.Time:0.667(Scan#:41)
MassPeaks:485
Spectrum Mode:Averaged 0.633-0.700(39-43) Base Peak:514(55277)
BG Mode:Calc Segment 1 - Event 1

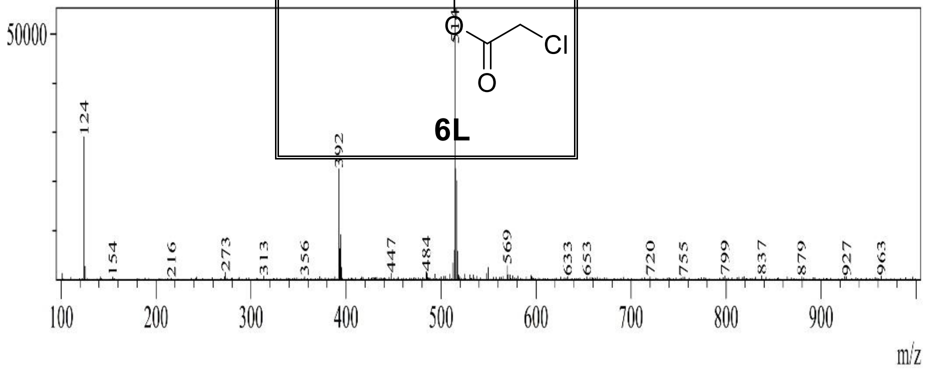

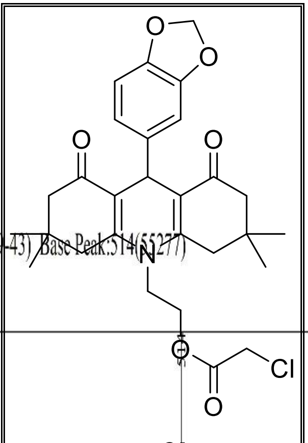

6L

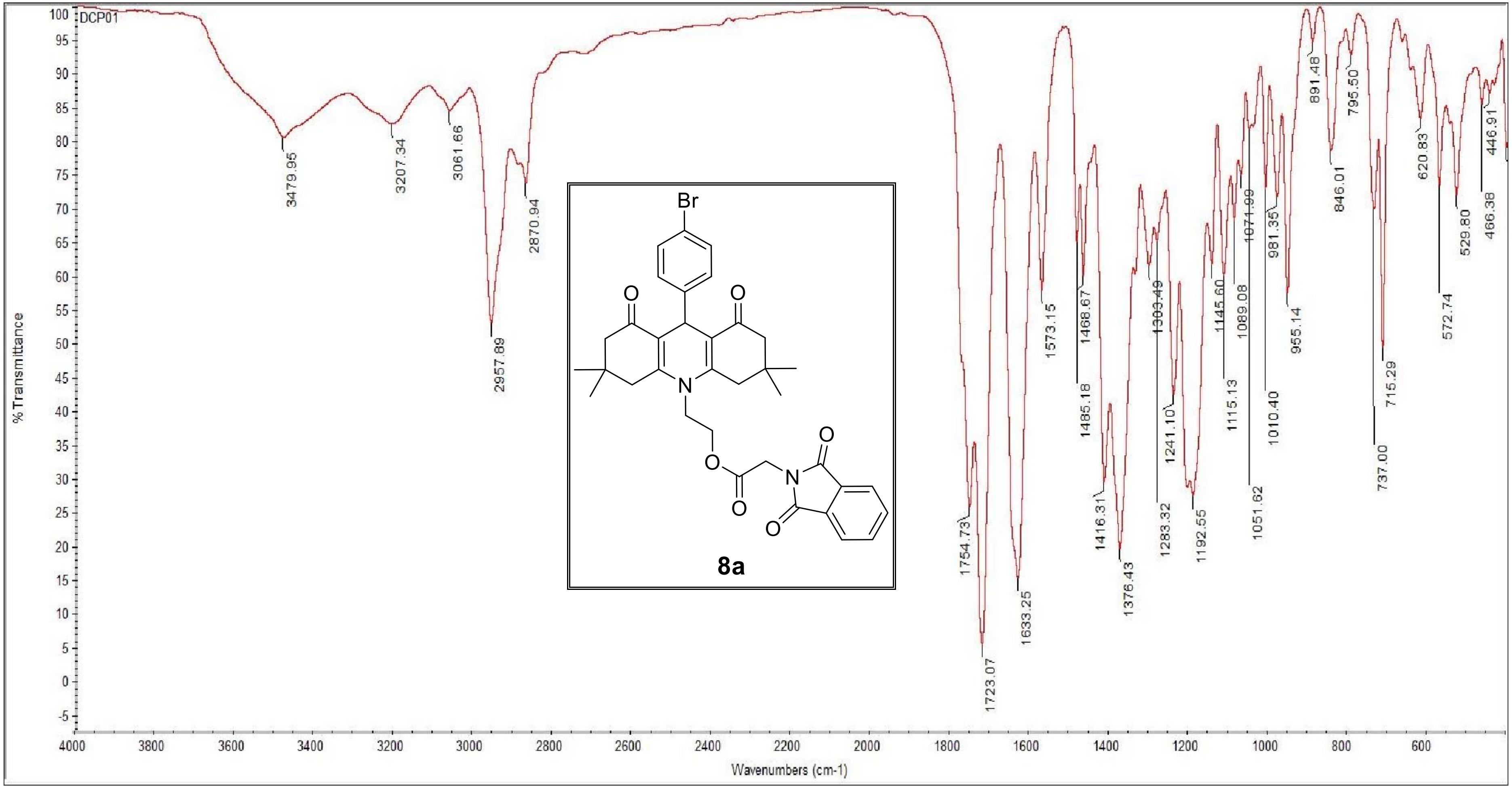

IR Spectrum of compound **8a**

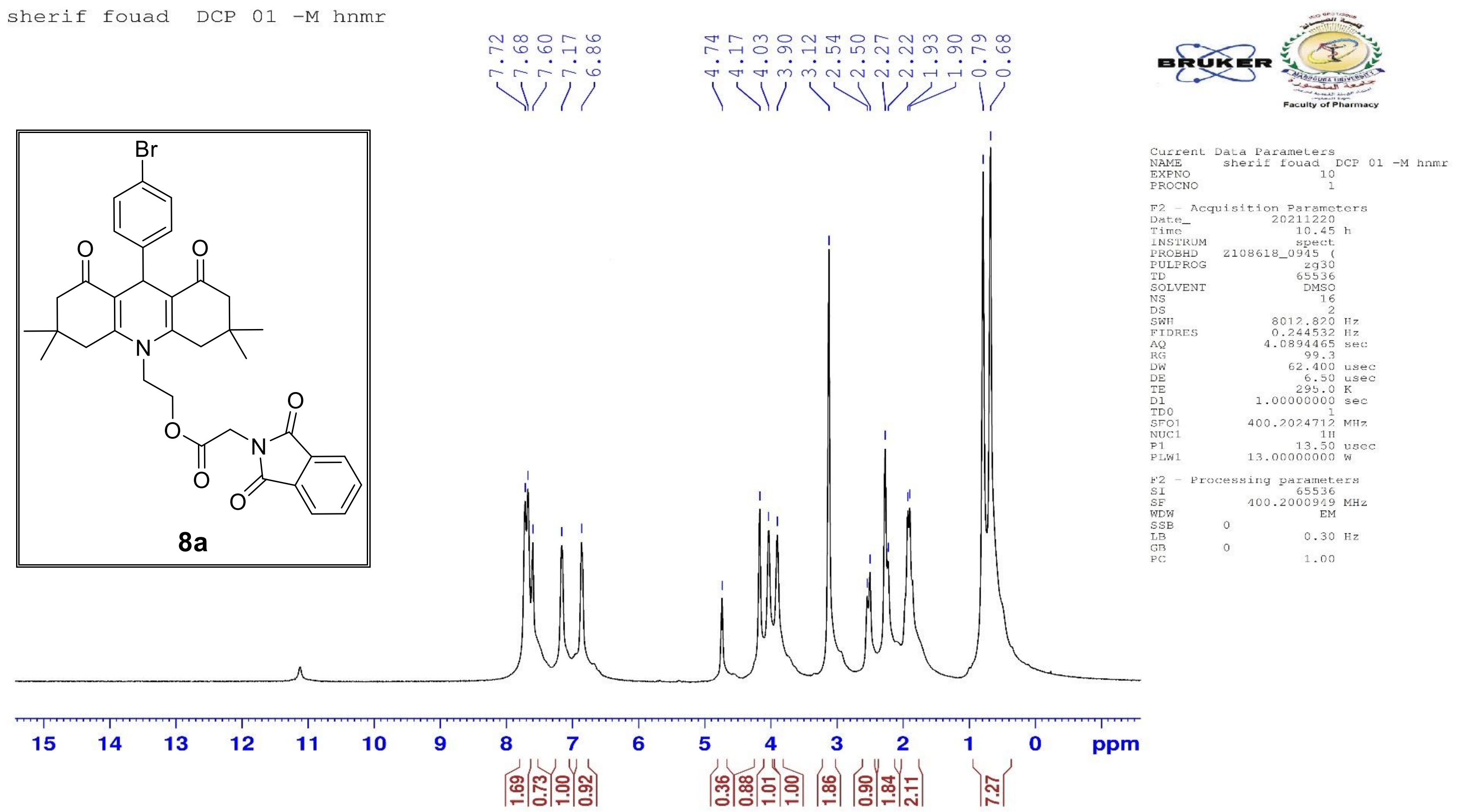

^{1}H NMR Spectrum of compound **8a**

Sample Information

Acquired by	: System Administrator
Date Acquired	: 01/12/2021 02:45:24 م
Sample Type	: Unknown
Sample Name	: DCP01
Sample ID	:
Dilution Factor	: 1
Tray#	: 1
Vial#	: 25
Injection Volume	: 10
Data File	: S_25.lcd
Method File	: Method_MS_only.lcm
Original Method File	: Method_MS_only.lcm
Report Format File	: DEFAULT.lsr
Tuning File	: default.lct
Processed by	: System Administrator
Date Processed	: 05/12/2021 09:46:07 ص

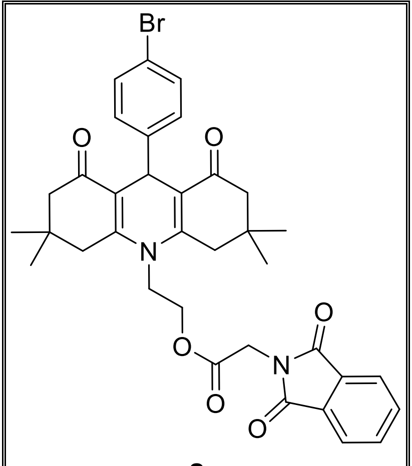

8a

MS Chromatogram

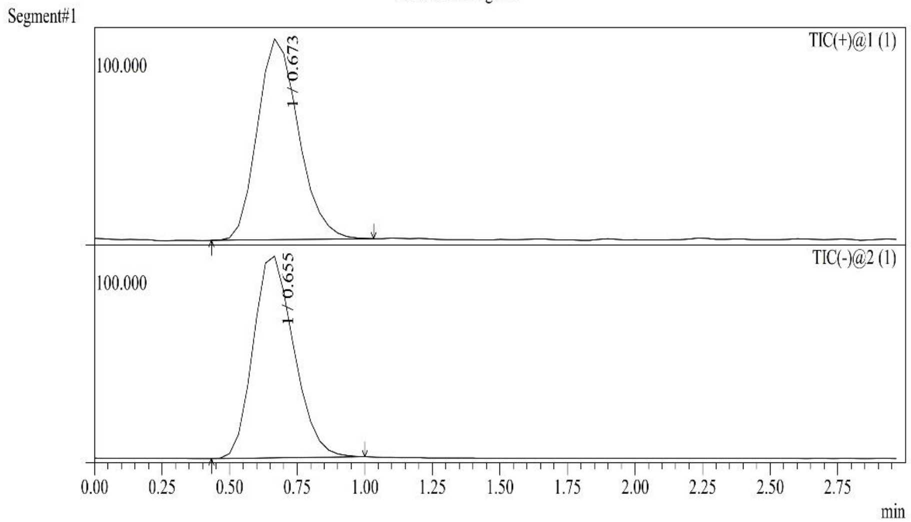

MASS Peak Table ALL MC

Peak#	Ret. Time	m/z	Area	Area%	Mark	A/H	Event#
1	0.673	TIC	90708328	100.000		10.579	1-1
2	0.655	TIC	209106952	100.000		10.464	1-2
Total			299815280	200.000			

Line#:1 R.Time:0.667(Scan#:41)
MassPeaks:763
Spectrum Mode:Averaged 0.633-0.700(39-43) Base Peak:661(1928534)
BG Mode:Calc Segment 1 - Event 1

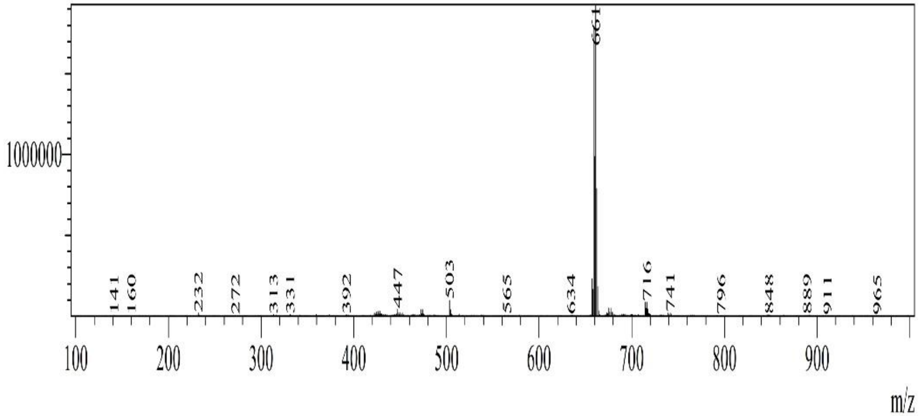

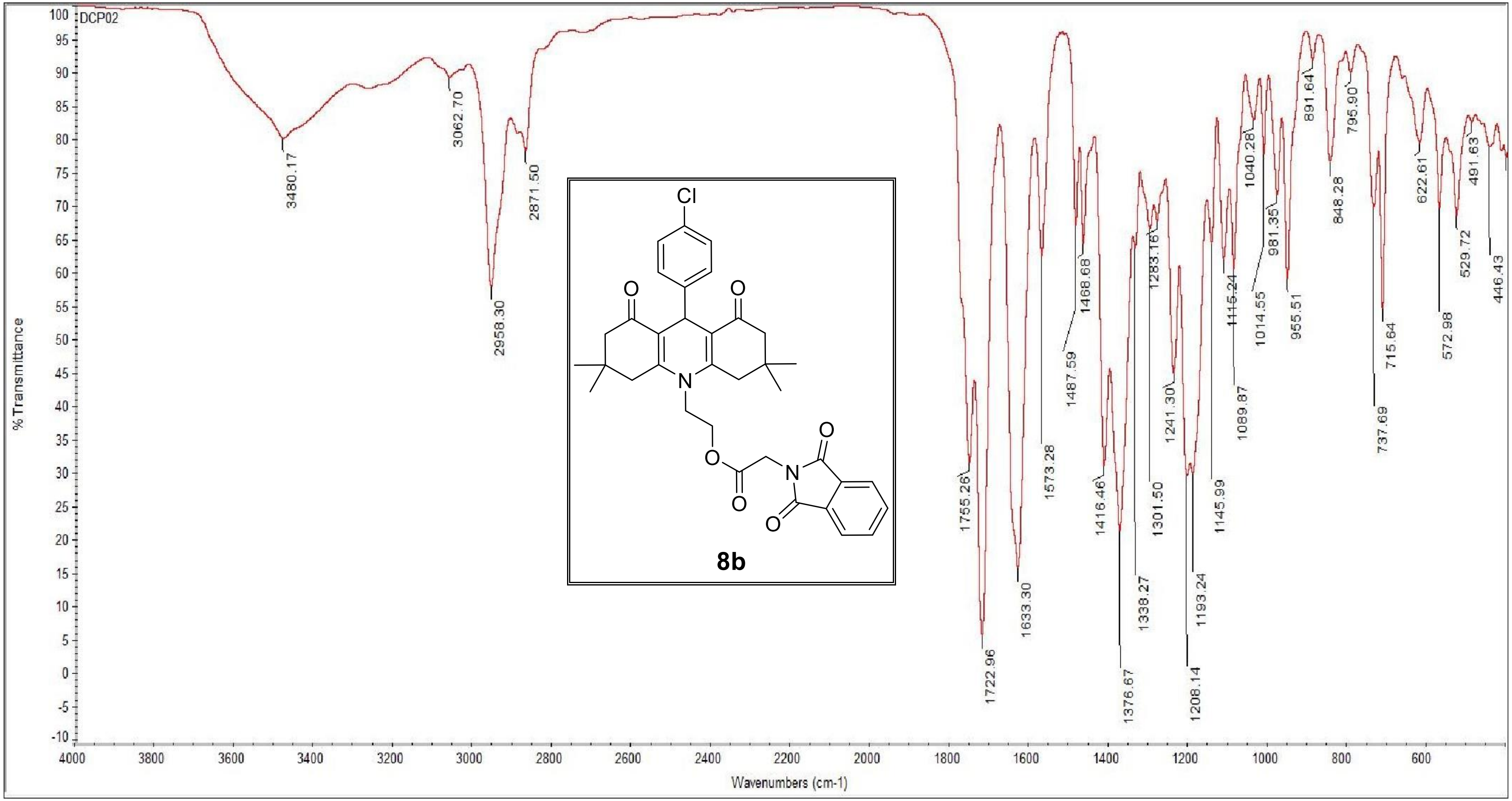

IR Spectrum of compound **8b**

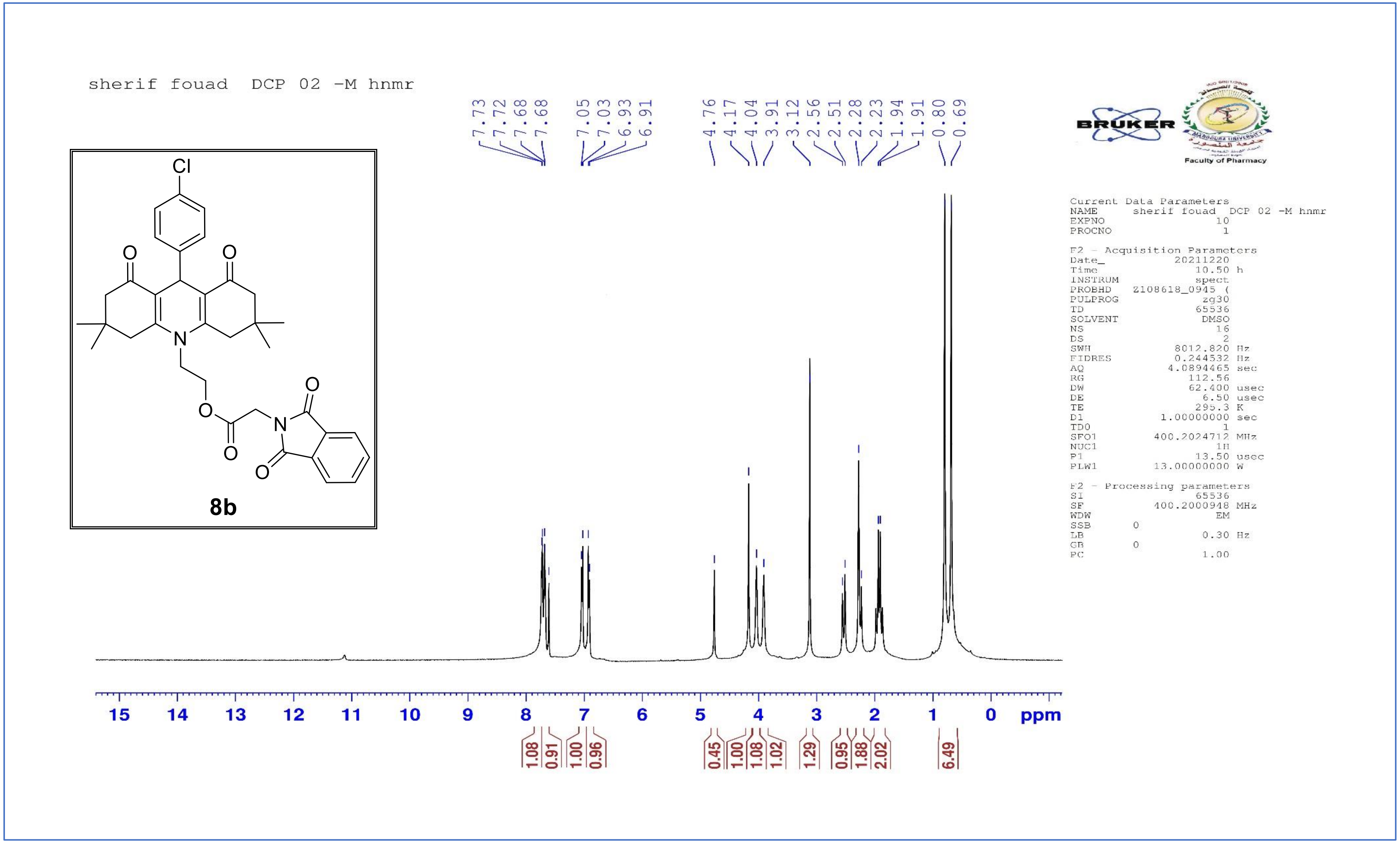
sherif fouad DCP 02 -M hnmr
7.73
7.72
7.68
7.68
7.05
7.03
6.93
6.91
4.76
4.17
4.04
3.91
3.12
2.56
2.51
2.28
2.23
1.94
1.91
0.80
0.69
BRUKER
Faculty of Pharmacy
Current Data Parameters
NAME sherif fouad DCP 02 -M hnmr
EXPNO 10
PROCNO 1
F2 - Acquisition Parameters
Date_ 20211220
Time 10.50 h
INSTRUM spect
PROBHD Z108618_0945 (
PULPROG zg30
TD 65536
SOLVENT DMSO
NS 16
DS 2
SWH 8012.820 Hz
FIDRES 0.244532 Hz
AQ 4.0894465 sec
RG 112.56
DW 62.400 usec
DE 6.50 usec
TE 295.3 K
D1 1.00000000 sec
TD0 1
SFO1 400.2024712 MHz
NUC1 1H
P1 13.50 usec
PLW1 13.00000000 W
F2 - Processing parameters
SI 65536
SF 400.2000948 MHz
WDW EM
SSB 0
LB 0.30 Hz
GB 0
PC 1.00
Cl
O
O
N
O
O
N
O
O
8b
15
14
13
12
11
10
9
8
7
6
5
4
3
2
1
0
ppm
1.08
0.91
1.00
0.96
0.45
1.00
1.08
1.02
1.29
0.95
1.88
2.02
6.49

Sample Information

Acquired by	: System Administrator
Date Acquired	: 01/12/2021 02:48:55 م
Sample Type	: Unknown
Sample Name	: DCP02
Sample ID	:
Dilution Factor	: 1
Tray#	: 1
Vial#	: 26
Injection Volume	: 10
Data File	: S_26.lcd
Method File	: Method_MS_only.lcm
Original Method File	: Method_MS_only.lcm
Report Format File	: DEFAULT.lsr
Tuning File	: default.lct
Processed by	: System Administrator
Date Processed	: 05/12/2021 09:46:26 ص

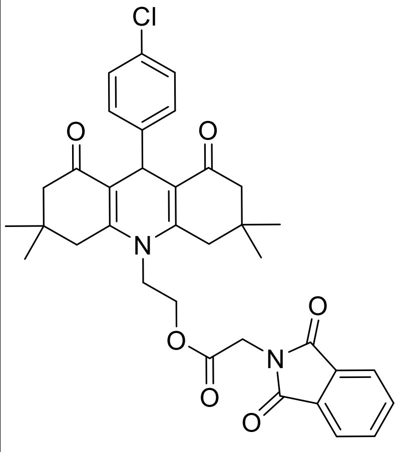

8b

MS Chromatogram

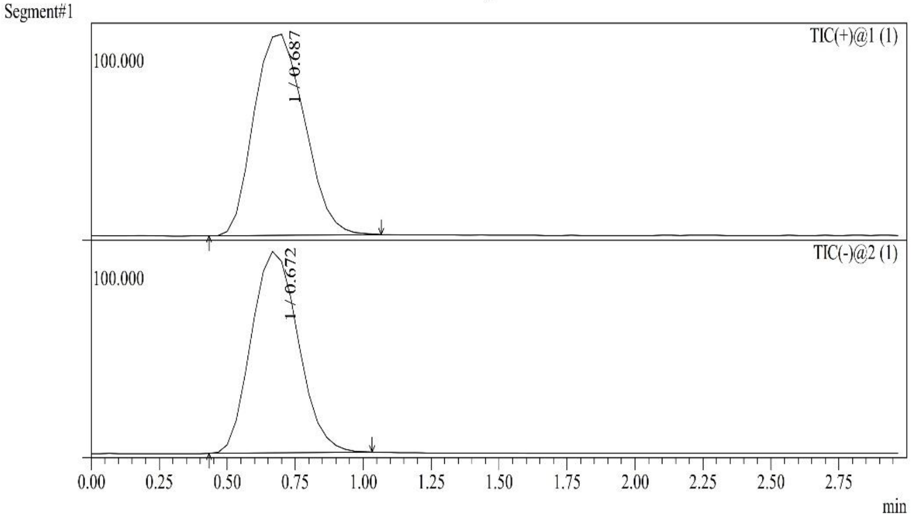

Line#:1 R.Time:0.667(Scan#:41)
MassPeaks:789
Spectrum Mode:Averaged 0.633-0.700(39-43) Base Peak:615(7440788)
BG Mode:Calc Segment 1 - Event 1

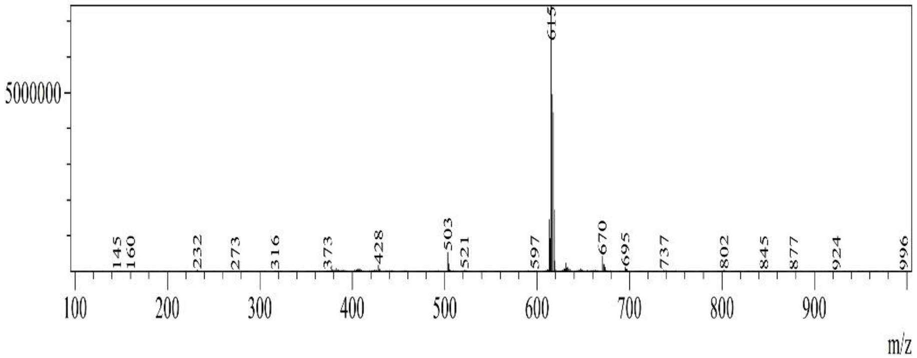

MASS Peak Table ALL MC

Peak#	Ret. Time	m/z	Area	Area%	Mark	A/H	Event#
1	0.687	TIC	370774521	100.000		12.961	1-1
2	0.672	TIC	831251186	100.000		12.011	1-2
Total			1202025707	200.000			

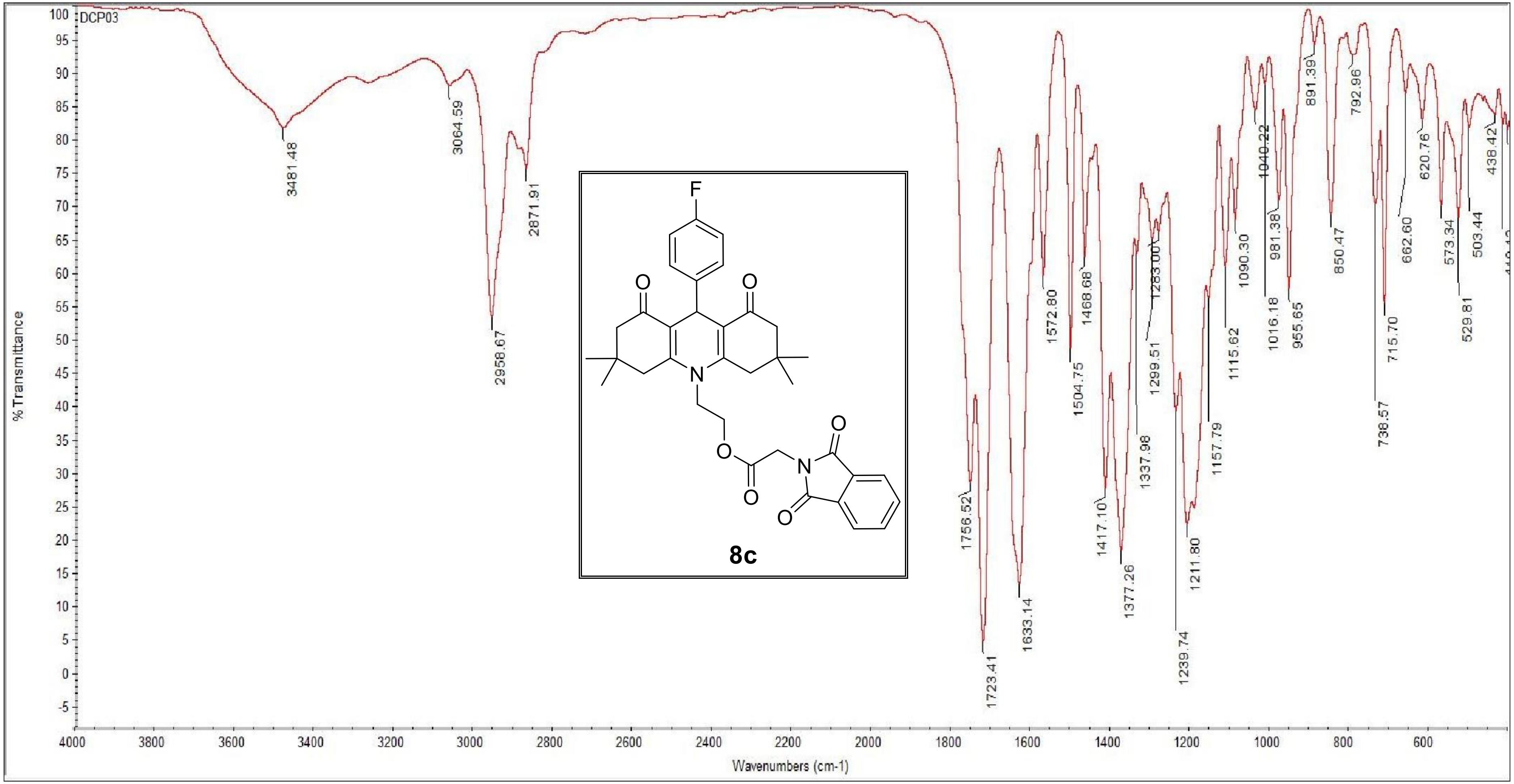

IR Spectrum of compound **8c**

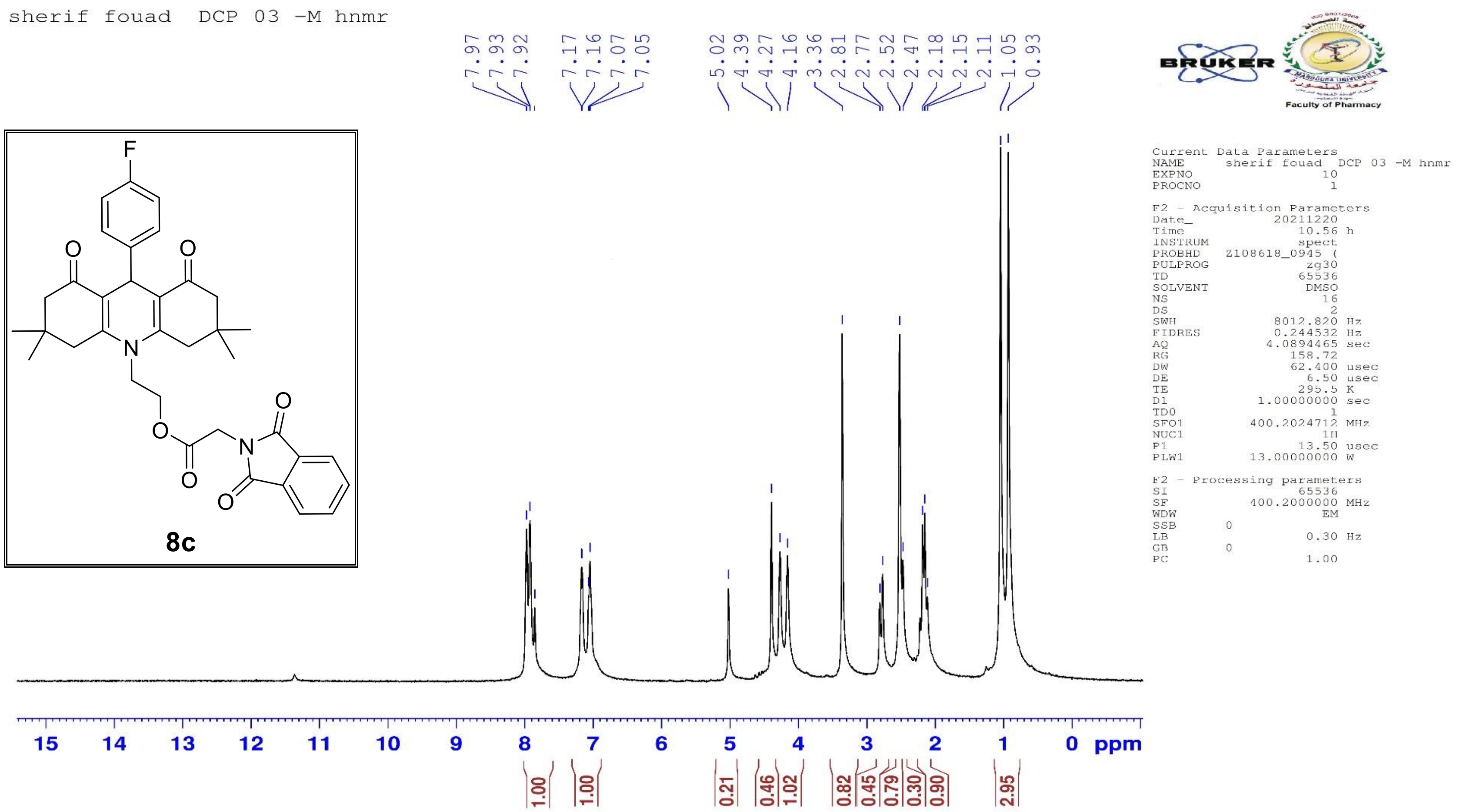
sherif fouad DCP 03 -M hnmr
7.97
7.93
7.92
7.17
7.16
7.07
7.05
5.02
4.39
4.27
4.16
3.36
2.81
2.77
2.52
2.47
2.18
2.15
2.11
1.05
0.93
BRUKER
Faculty of Pharmacy
F
O
O
N
O
O
N
O
O
8c
Current Data Parameters
NAME sherif fouad DCP 03 -M hnmr
EXPNO 10
PROCNO 1
F2 - Acquisition Parameters
Date_ 20211220
Time 10.56 h
INSTRUM spect
PROBHD Z108618_0945 (
PULPROG zg30
TD 65536
SOLVENT DMSO
NS 16
DS 2
SWH 8012.820 Hz
FIDRES 0.244532 Hz
AQ 4.0894465 sec
RG 158.72
DW 62.400 usec
DE 6.50 usec
TE 295.5 K
D1 1.00000000 sec
TD0 1
SFO1 400.2024712 MHz
NUC1 1H
P1 13.50 usec
PLW1 13.00000000 W
F2 - Processing parameters
SI 65536
SF 400.2000000 MHz
WDW EM
SSB 0
LB 0.30 Hz
GB 0
PC 1.00
15 14 13 12 11 10 9 8 7 6 5 4 3 2 1 0 ppm
1.00
1.00
0.21
0.46
1.02
0.82
0.45
0.79
0.30
0.90
2.95

Sample Information

Acquired by	: System Administrator
Date Acquired	: 01/12/2021 02:52:26 م
Sample Type	: Unknown
Sample Name	: DCP03
Sample ID	:
Dilution Factor	: 1
Tray#	: 1
Vial#	: 27
Injection Volume	: 10
Data File	: S_27.lcd
Method File	: Method_MS_only.lcm
Original Method File	: Method_MS_only.lcm
Report Format File	: DEFAULT.lsr
Tuning File	: default.lct
Processed by	: System Administrator
Date Processed	: 05/12/2021 09:47:00 ص

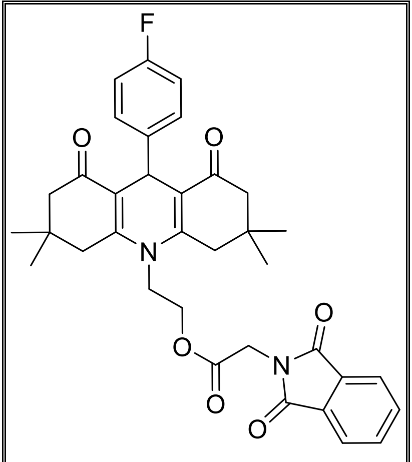

8c

MS Chromatogram

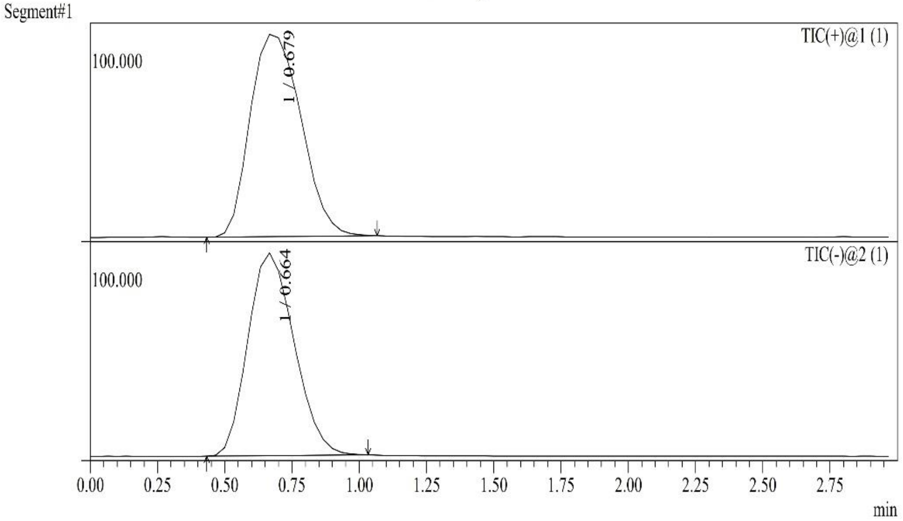

MASS Peak Table ALL MC

Peak#	Ret. Time	m/z	Area	Area%	Mark	A/H	Event#
1	0.679	TIC	365646172	100.000		13.185	1-1
2	0.664	TIC	802812558	100.000		11.984	1-2
Total			1168458730	200.000			

Line#:1 R.Time:0.667(Scan#:41)
MassPeaks:832
Spectrum Mode:Averaged 0.633-0.700(39-43) Base Peak:599(7973040)
BG Mode:Calc Segment 1 - Event 1

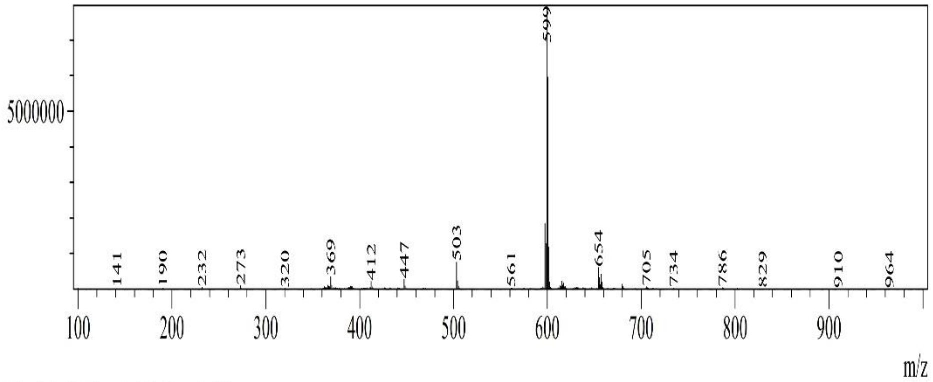

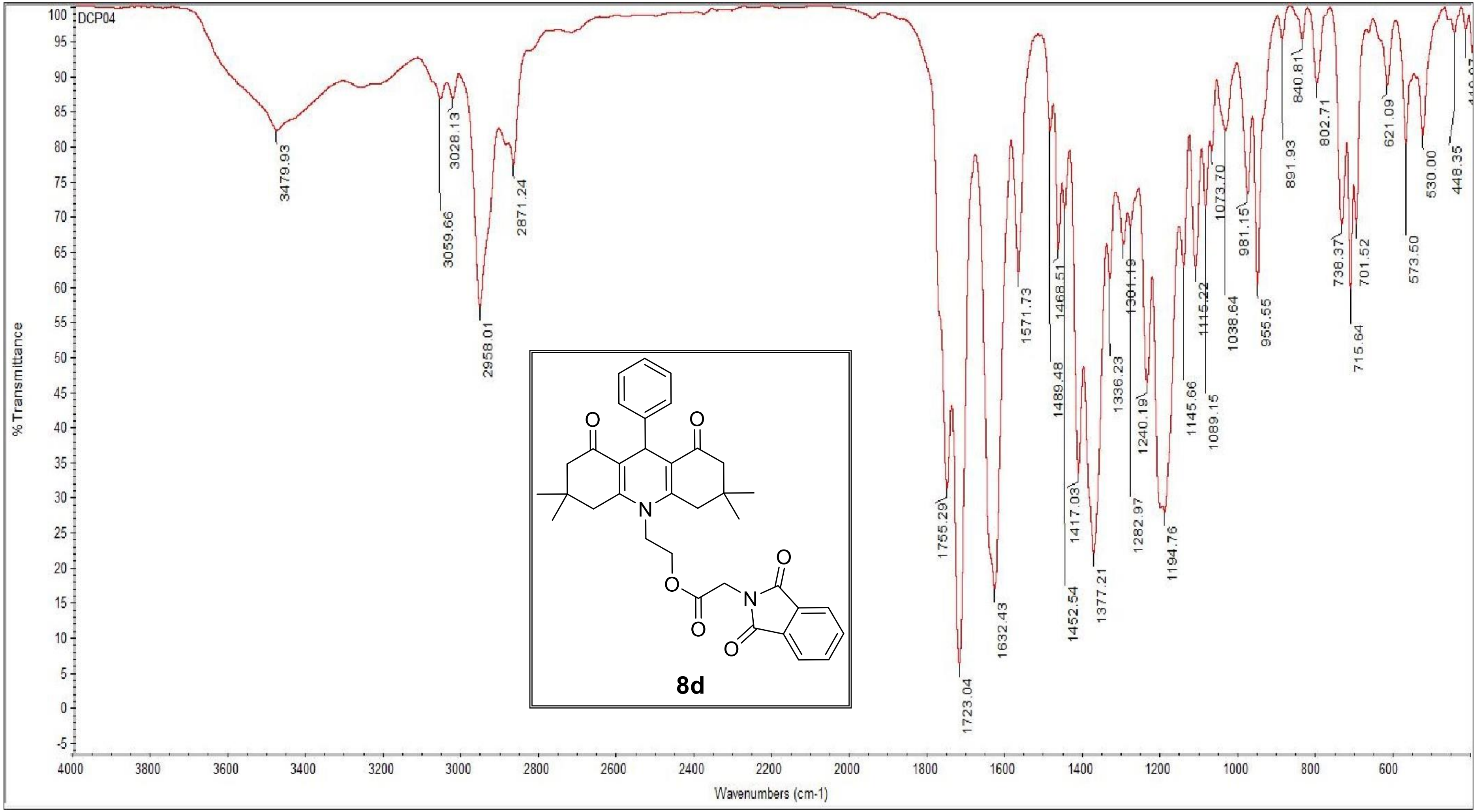

IR Spectrum of compound **8d**

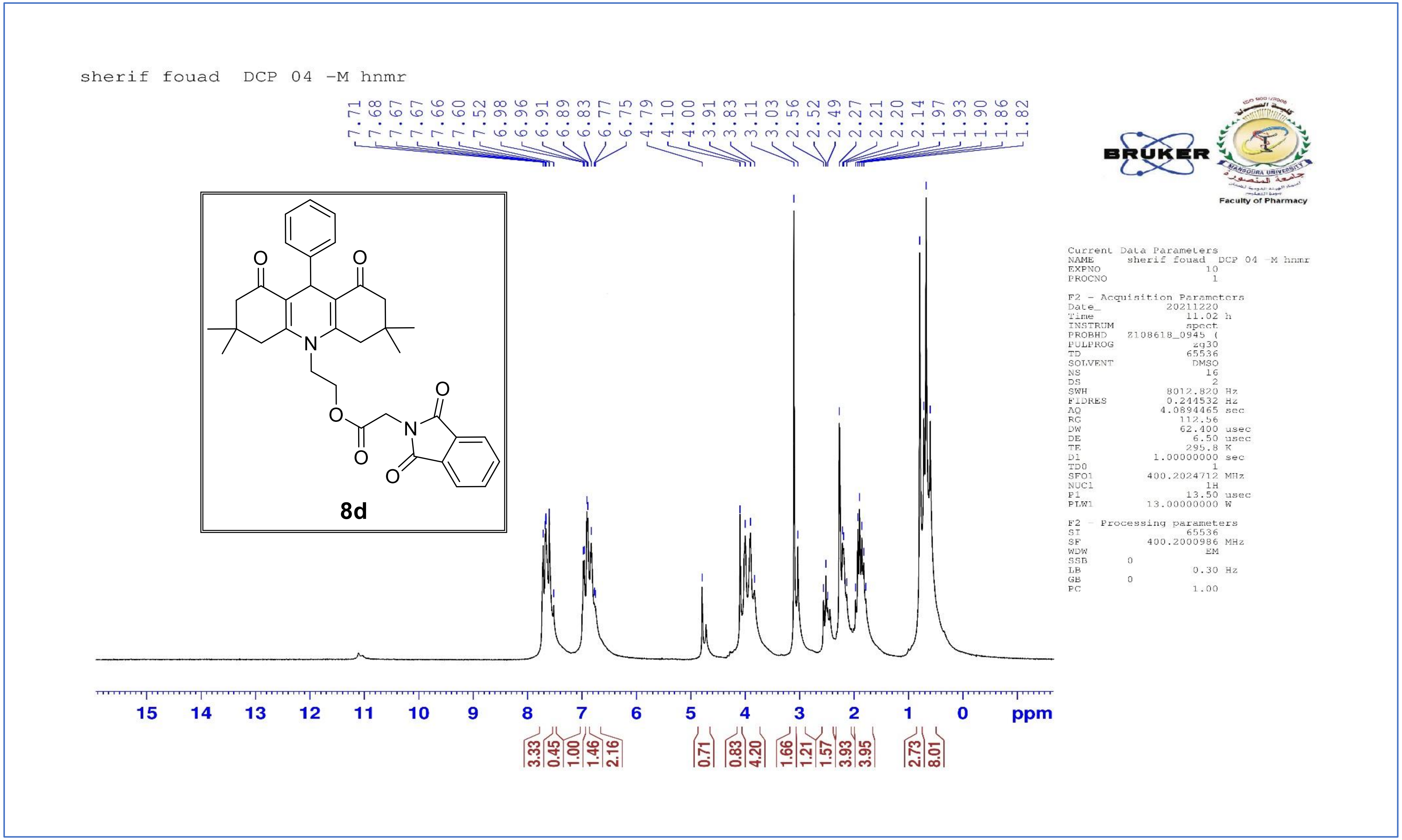
sherif fouad DCP 04 -M hnmr
7.71
7.68
7.67
7.67
7.66
7.60
7.52
6.98
6.96
6.91
6.89
6.83
6.77
6.75
4.79
4.10
4.00
3.91
3.83
3.11
3.03
2.56
2.52
2.49
2.27
2.21
2.20
2.14
1.97
1.93
1.90
1.86
1.82
BRUKER
Faculty of Pharmacy
MANSOURA UNIVERSITY
O
O
N
O
O
N
O
O
8d
Current Data Parameters
NAME sherif fouad DCP 04 -M hnmr
EXPNO 10
PROCNO 1
F2 - Acquisition Parameters
Date_ 20211220
Time 11.02 h
INSTRUM spect
PROBHD Z108618_0945 (
PULPROG zg30
TD 65536
SOLVENT DMSO
NS 16
DS 2
SWH 8012.820 Hz
FIDRES 0.244532 Hz
AQ 4.0894465 sec
RG 112.56
DW 62.400 usec
DE 6.50 usec
TE 295.8 K
D1 1.00000000 sec
TD0 1
SFO1 400.2024712 MHz
NUC1 1H
P1 13.50 usec
PLW1 13.00000000 W
F2 - Processing parameters
SI 65536
SF 400.2000986 MHz
WDW EM
SSB 0
LB 0.30 Hz
GB 0
PC 1.00
15 14 13 12 11 10 9 8 7 6 5 4 3 2 1 0 ppm
3.33
0.45
1.00
1.46
2.16
0.71
0.83
4.20
1.66
1.21
1.57
3.93
3.95
2.73
8.01

Sample Information

Acquired by	: System Administrator
Date Acquired	: 01/12/2021 02:55:57 م
Sample Type	: Unknown
Sample Name	: DCP04
Sample ID	:
Dilution Factor	: 1
Tray#	: 1
Vial#	: 28
Injection Volume	: 10
Data File	: S_28.lcd
Method File	: Method_MS_only.lcm
Original Method File	: Method_MS_only.lcm
Report Format File	: DEFAULT.lsr
Tuning File	: default.lct
Processed by	: System Administrator
Date Processed	: 05/12/2021 09:47:18 ص

MS Chromatogram

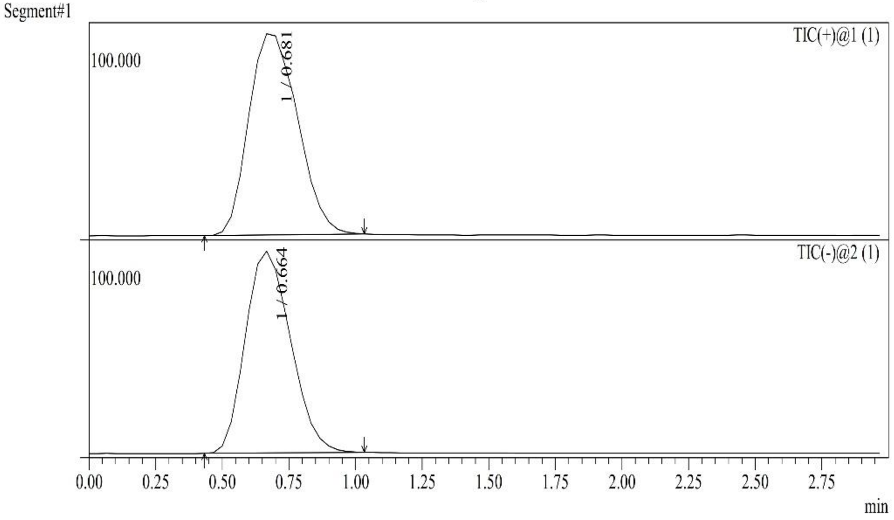

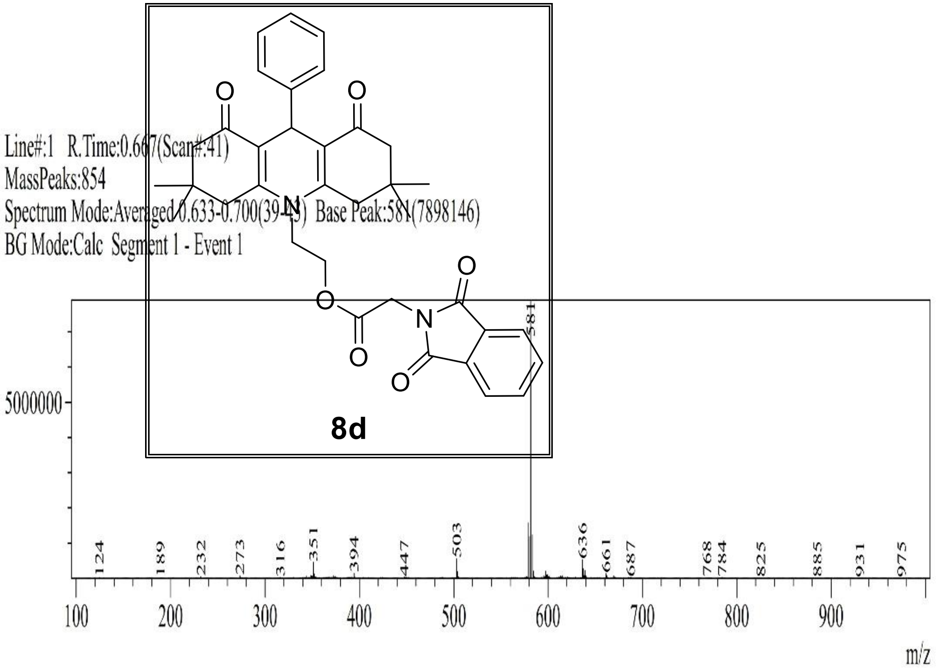

MASS Peak Table ALL MC

Peak#	Ret. Time	m/z	Area	Area%	Mark	A/H	Event#
1	0.681	TIC	331163350	100.000		12.776	1-1
2	0.664	TIC	672375708	100.000		11.832	1-2
Total			1003539058	200.000			

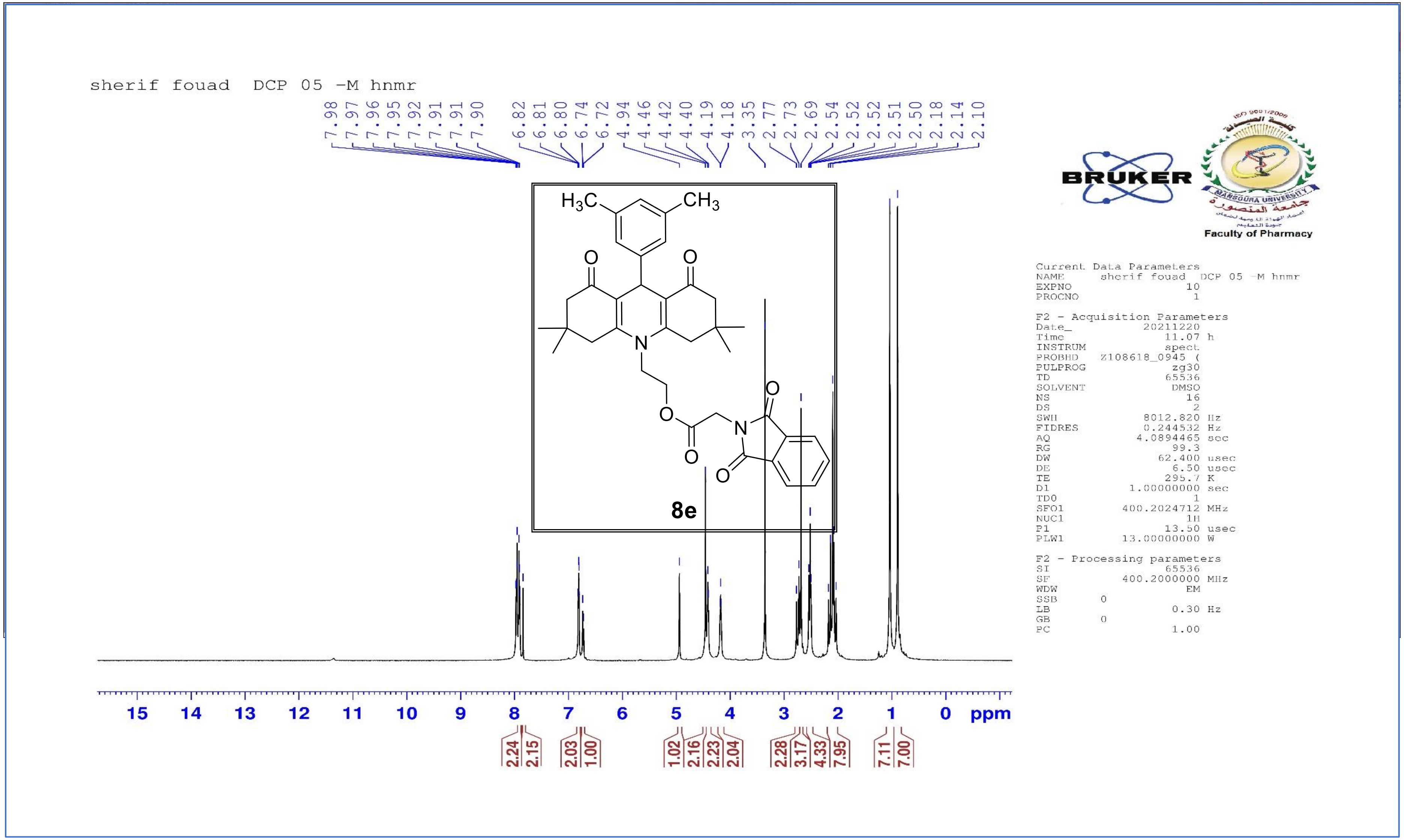
sherif fouad DCP 05 -M hnmr
7.98 7.97 7.96 7.95 7.92 7.91 7.91 7.90 6.82 6.81 6.80 6.74 6.72 4.94 4.46 4.42 4.40 4.19 4.18 3.35 2.77 2.73 2.69 2.54 2.52 2.52 2.51 2.50 2.18 2.14 2.10
H3C
CH3
O
N
8e
BRUKER
Faculty of Pharmacy
Current Data Parameters
NAME sherif fouad DCP 05 -M hnmr
EXPNO 10
PROCNO 1
F2 - Acquisition Parameters
Date_ 20211220
Time 11.07 h
INSTRUM spect
PROBHD Z108618_0945 (
PULPROG zg30
TD 65536
SOLVENT DMSO
NS 16
DS 2
SWH 8012.820 Hz
FIDRES 0.244532 Hz
AQ 4.0894465 sec
RG 99.3
DW 62.400 usec
DE 6.50 usec
TE 295.7 K
D1 1.00000000 sec
TD0 1
SFO1 400.2024712 MHz
NUC1 1H
P1 13.50 usec
PLW1 13.00000000 W
F2 - Processing parameters
SI 65536
SF 400.2000000 MHz
WDW EM
SSB 0
LB 0.30 Hz
GB 0
PC 1.00
15 14 13 12 11 10 9 8 7 6 5 4 3 2 1 0 ppm
2.24 2.15 2.03 1.00 1.02 2.16 2.23 2.04 2.28 3.17 4.33 7.95 7.11 7.00

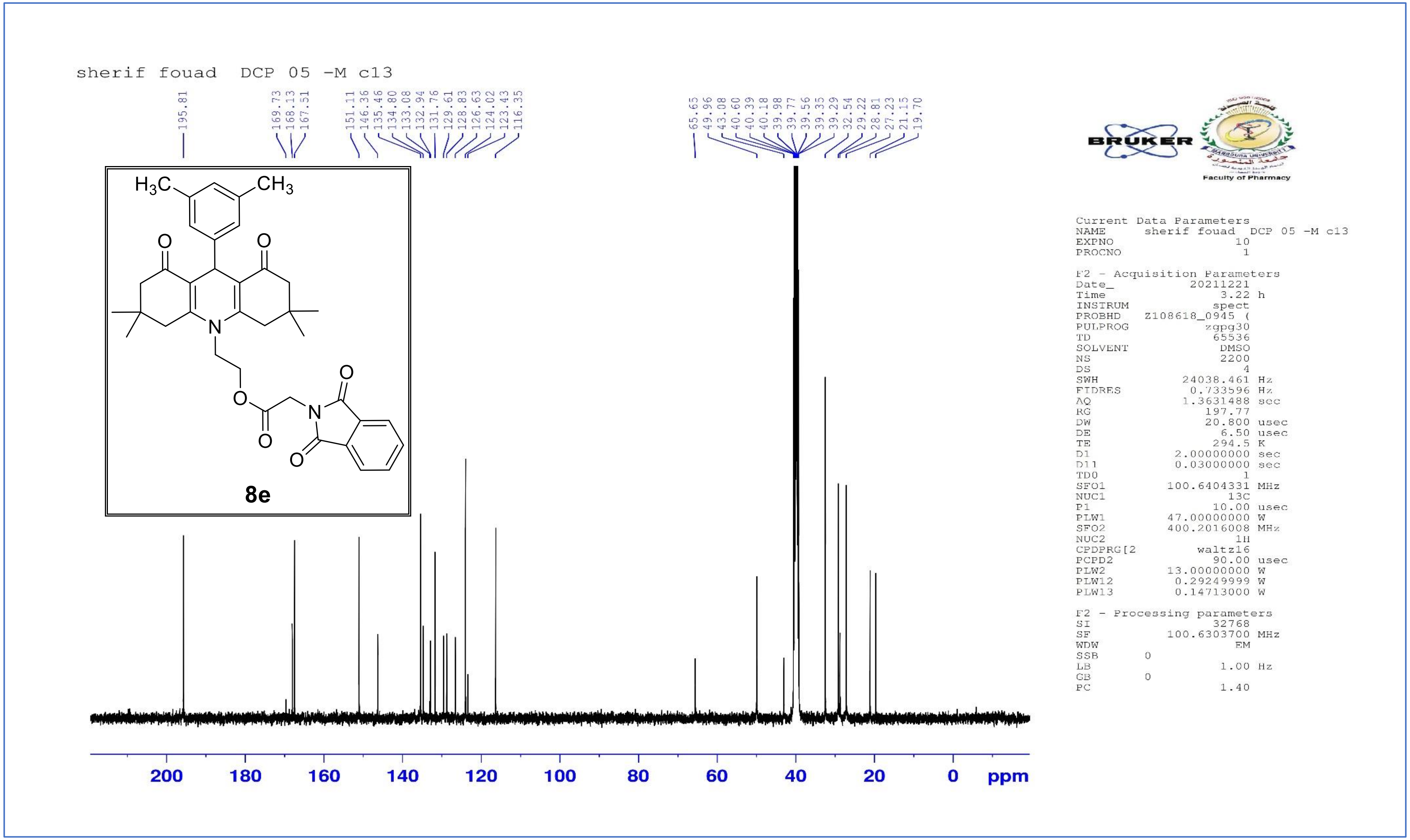
sherif fouad DCP 05 -M c13
195.81
169.73 168.13 167.51
151.11 146.36 135.46 134.80 133.08 132.94 131.76 129.61 128.83 126.63 124.02 123.43 116.35
65.65 49.96 43.08 40.60 40.39 40.18 39.98 39.77 39.56 39.35 39.29 32.54 29.22 28.81 27.23 21.15 19.70
8e
BRUKER
Faculty of Pharmacy
Current Data Parameters
NAME sherif fouad DCP 05 -M c13
EXPNO 10
PROCNO 1
F2 - Acquisition Parameters
Date_ 20211221
Time 3.22 h
INSTRUM spect
PROBHD Z108618_0945 (
PULPROG zgpg30
TD 65536
SOLVENT DMSO
NS 2200
DS 4
SWH 24038.461 Hz
FIDRES 0.733596 Hz
AQ 1.3631488 sec
RG 197.77
DW 20.800 usec
DE 6.50 usec
TE 294.5 K
D1 2.00000000 sec
D11 0.03000000 sec
TD0 1
SFO1 100.6404331 MHz
NUC1 13C
P1 10.00 usec
PLW1 47.00000000 W
SFO2 400.2016008 MHz
NUC2 1H
CPDPRG[2 waltz16
PCPD2 90.00 usec
PLW2 13.00000000 W
PLW12 0.29249999 W
PLW13 0.14713000 W
F2 - Processing parameters
SI 32768
SF 100.6303700 MHz
WDW EM
SSB 0
LB 1.00 Hz
GB 0
PC 1.40
200 180 160 140 120 100 80 60 40 20 0 ppm

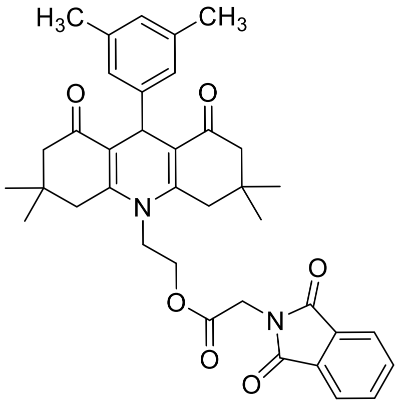

8e

Sample Information

Acquired by	: System Administrator
Date Acquired	: 01/12/2021 02:59:28 م
Sample Type	: Unknown
Sample Name	: DCP05
Sample ID	:
Dilution Factor	: 1
Tray#	: 1
Vial#	: 29
Injection Volume	: 10
Data File	: S_29.lcd
Method File	: Method_MS_only.lcm
Original Method File	: Method_MS_only.lcm
Report Format File	: DEFAULT.lsr
Tuning File	: default.lct
Processed by	: System Administrator
Date Processed	: 05/12/2021 09:47:36 ص

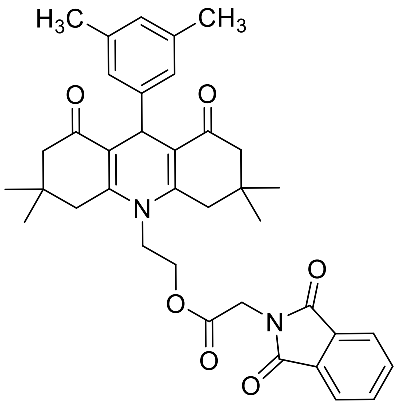

8e

MS Chromatogram

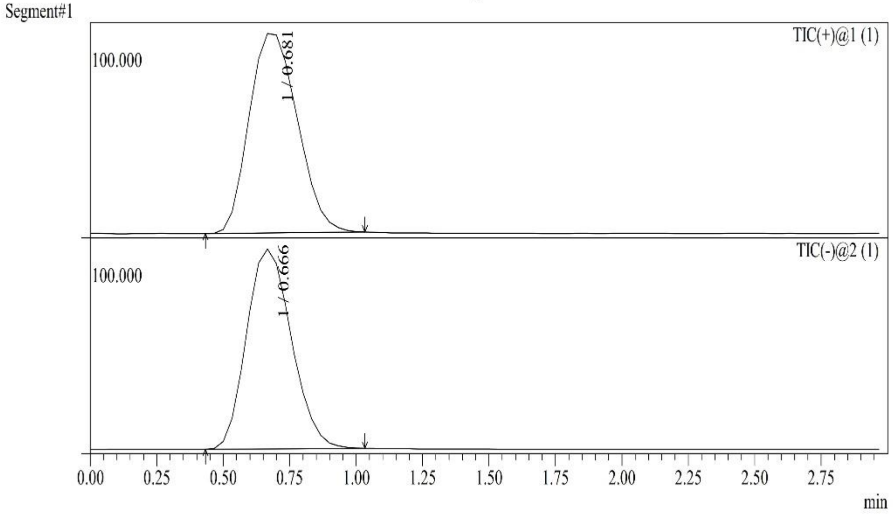

Line#:1 R.Time:0.667(Scan#:41)
MassPeaks:829
Spectrum Mode:Averaged 0.633-0.700(39-43) Base Peak:609(7178844)
BG Mode:Calc Segment 1 - Event 1

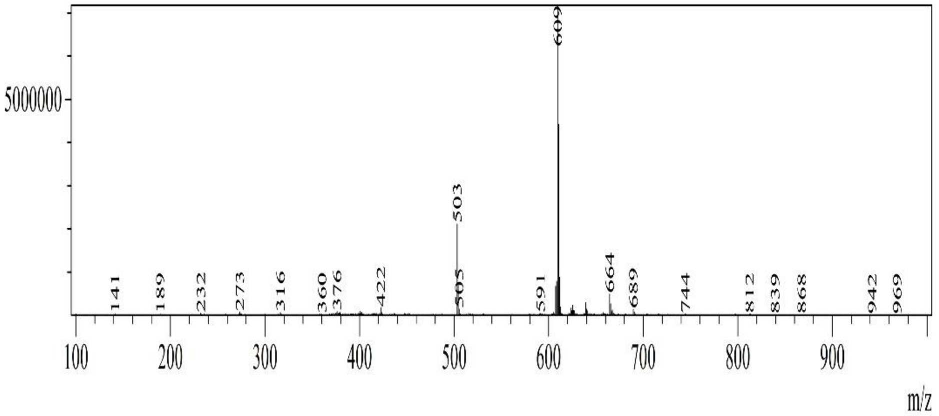

MASS Peak Table ALL MC

Peak#	Ret. Time	m/z	Area	Area%	Mark	A/H	Event#
1	0.681	TIC	306224904	100.000		12.646	1-1
2	0.666	TIC	603607758	100.000		11.767	1-2
Total			909832662	200.000			

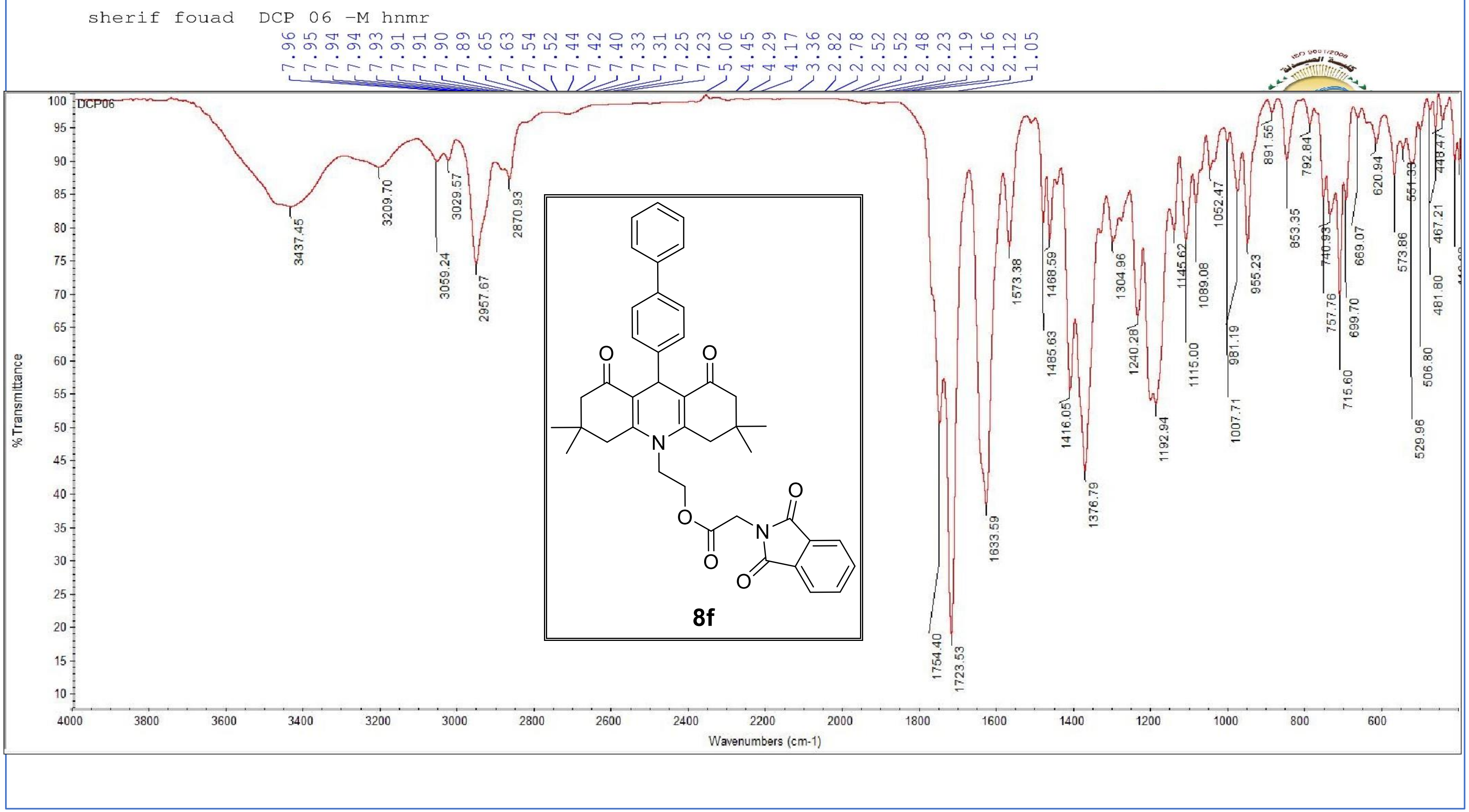
sherif fouad DCP 06 -M hnmr
7.96 7.95 7.94 7.94 7.93 7.91 7.91 7.90 7.89 7.65 7.63 7.54 7.52 7.44 7.42 7.40 7.33 7.31 7.25 7.23 5.06 4.45 4.29 4.17 3.36 2.82 2.78 2.52 2.52 2.48 2.23 2.19 2.16 2.12 1.05
DCP06
%Transmittance
Wavenumbers (cm-1)
3437.45
3209.70
3059.24
3029.57
2957.67
2870.93
1754.40
1723.53
1633.59
1573.38
1485.63
1468.59
1416.05
1376.79
1304.96
1240.28
1192.94
1145.62
1115.00
1089.08
1052.47
1007.71
981.19
955.23
891.55
853.35
792.84
757.76
740.93
715.60
699.70
669.07
620.94
573.86
551.33
529.96
506.80
481.80
467.21
448.47
8f

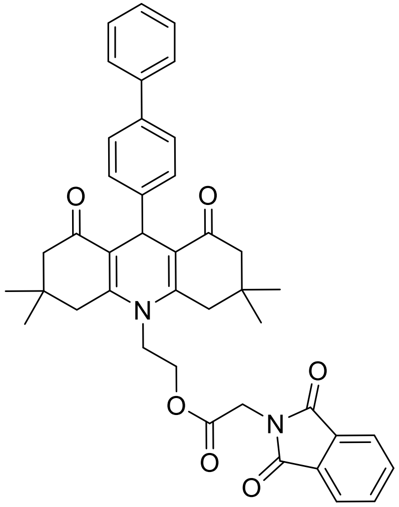

8f

Sample Information

Acquired by	: System Administrator
Date Acquired	: 01/12/2021 03:03:01 م
Sample Type	: Unknown
Sample Name	: DCP06
Sample ID	:
Dilution Factor	: 1
Tray#	: 1
Vial#	: 30
Injection Volume	: 10
Data File	: S_30.lcd
Method File	: Method_MS_only.lcm
Original Method File	: Method_MS_only.lcm
Report Format File	: DEFAULT.lsr
Tuning File	: default.lct
Processed by	: System Administrator
Date Processed	: 05/12/2021 09:47:54 ص

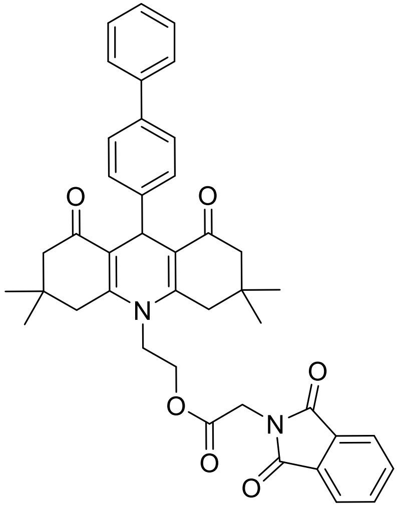

8f

MS Chromatogram

Segment#1

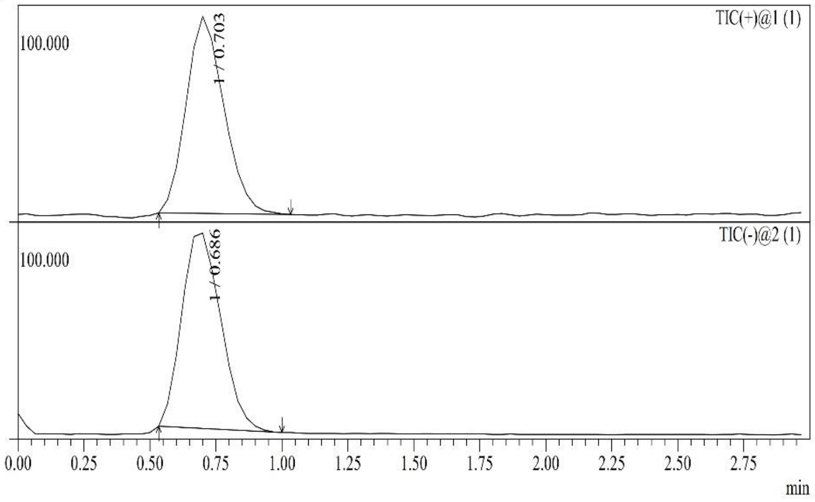

MASS Peak Table ALL MC

Peak#	Ret. Time	m/z	Area	Area%	Mark	A/H	Event#
1	0.703	TIC	31307879	100.000		9.918	1-1
2	0.686	TIC	51790092	100.000		10.318	1-2
Total			83097971	200.000			

Line#:1 R.Time:0.700(Scan#:43)
MassPeaks:581
Spectrum Mode:Averaged 0.667-0.733(41-45) Base Peak:657(1261863)
BG Mode:Calc Segment 1 - Event 1

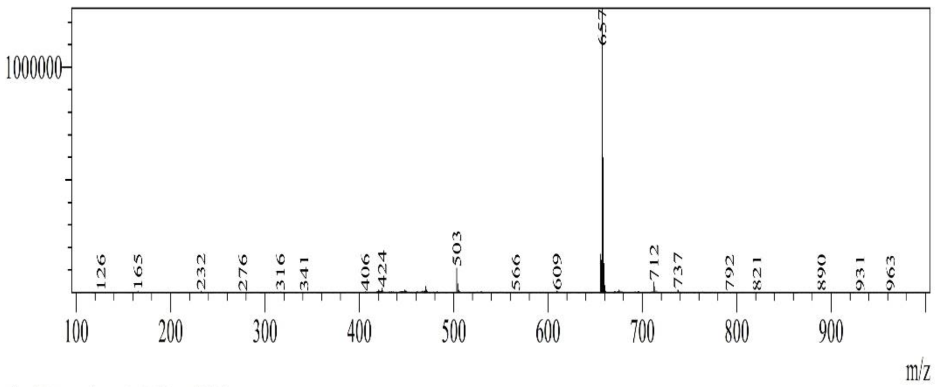

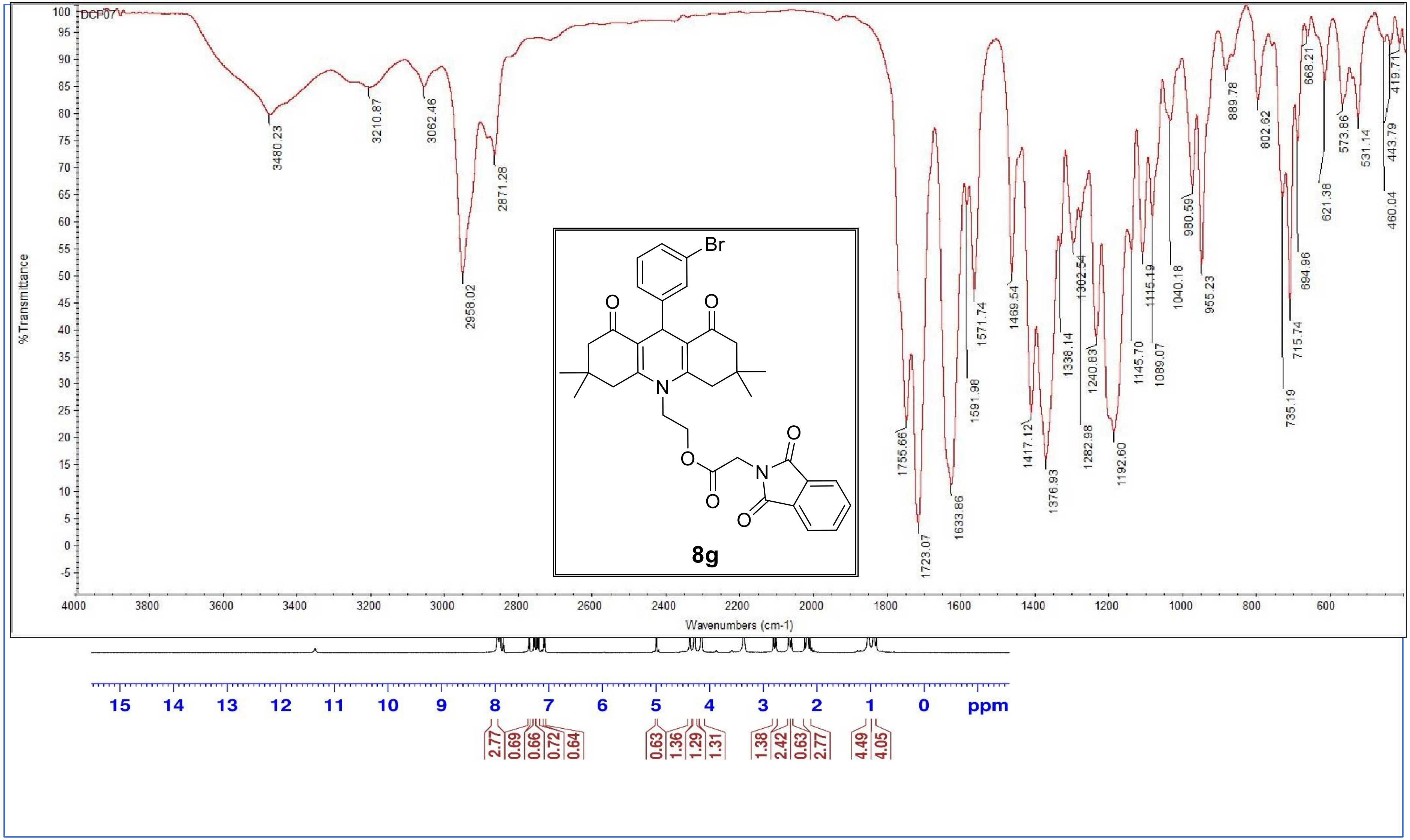
DCP07
% Transmittance
Wavenumbers (cm-1)
3480.23
3210.87
3062.46
2958.02
2871.28
1755.66
1723.07
1633.86
1591.98
1571.74
1469.54
1417.12
1376.93
1338.14
1302.54
1282.98
1240.83
1192.60
1145.70
1115.19
1089.07
1040.18
980.59
955.23
889.78
802.62
735.19
715.74
694.96
668.21
621.38
573.86
531.14
460.04
443.79
419.71
Br
N
8g
ppm
2.77
0.69
0.66
0.72
0.64
0.63
1.36
1.29
1.31
1.38
2.42
0.63
2.77
4.49
4.05

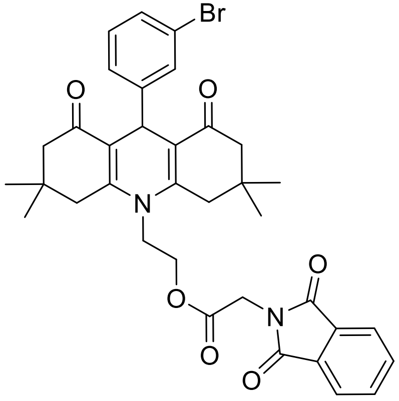

8g

Sample Information

Acquired by	: System Administrator
Date Acquired	: 01/12/2021 03:06:32 م
Sample Type	: Unknown
Sample Name	: DCP07
Sample ID	:
Dilution Factor	: 1
Tray#	: 1
Vial#	: 31
Injection Volume	: 10
Data File	: S_31.lcd
Method File	: Method_MS_only.lcm
Original Method File	: Method_MS_only.lcm
Report Format File	: DEFAULT.lsr
Tuning File	: default.lct
Processed by	: System Administrator
Date Processed	: 05/12/2021 09:48:14 ص

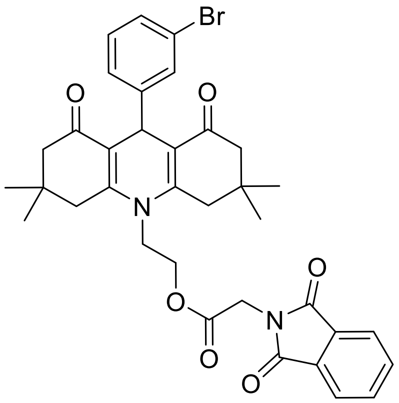

8g

MS Chromatogram

Segment#1

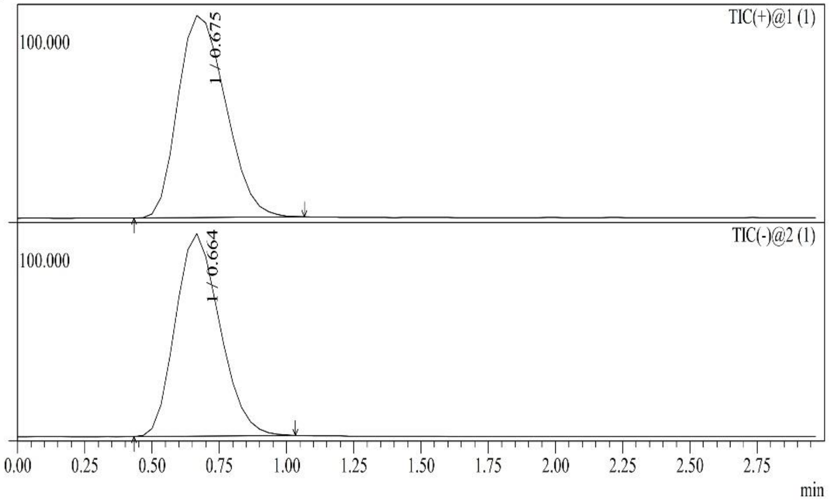

Line#:1 R.Time:0.667(Scan#:41)
MassPeaks:859
Spectrum Mode:Averaged 0.633-0.700(39-43) Base Peak:659(5908199)
BG Mode:Calc Segment 1 - Event 1

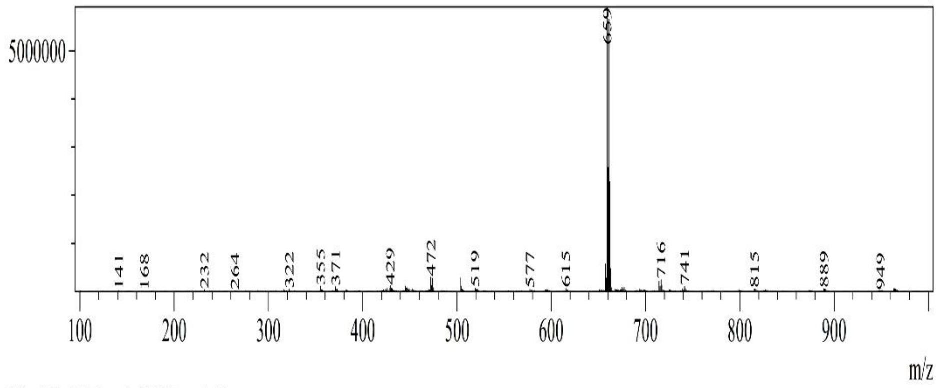

MASS Peak Table ALL MC

Peak#	Ret. Time	m/z	Area	Area%	Mark	A/H	Event#
1	0.675	TIC	334861747	100.000		12.372	1-1
2	0.664	TIC	622431378	100.000		11.491	1-2
Total			957293125	200.000			

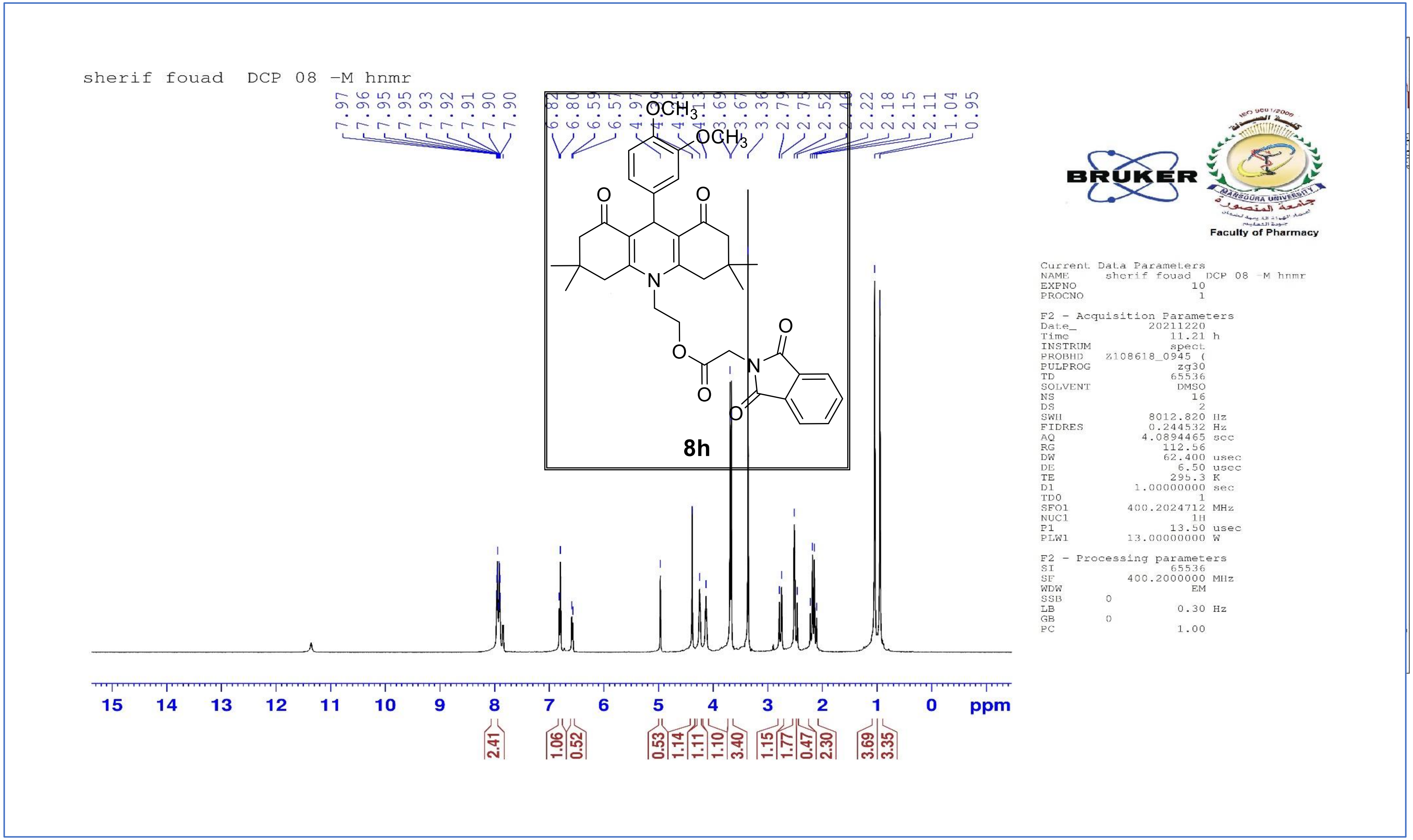
sherif fouad DCP 08 -M hnmr
7.97
7.96
7.95
7.95
7.93
7.92
7.91
7.90
7.90
OCH3
OCH3
O
O
N
O
O
N
O
O
8h
BRUKER
MANSOURA UNIVERSITY
Faculty of Pharmacy
Current Data Parameters
NAME sherif fouad DCP 08 -M hnmr
EXPNO 10
PROCNO 1
F2 - Acquisition Parameters
Date_ 20211220
Time 11.21 h
INSTRUM spect
PROBHD Z108618_0945 (
PULPROG zg30
TD 65536
SOLVENT DMSO
NS 16
DS 2
SWH 8012.820 Hz
FIDRES 0.244532 Hz
AQ 4.0894465 sec
RG 112.56
DW 62.400 usec
DE 6.50 usec
TE 295.3 K
D1 1.00000000 sec
TD0 1
SFO1 400.2024712 MHz
NUC1 1H
P1 13.50 usec
PLW1 13.00000000 W
F2 - Processing parameters
SI 65536
SF 400.2000000 MHz
WDW EM
SSB 0
LB 0.30 Hz
GB 0
PC 1.00
15 14 13 12 11 10 9 8 7 6 5 4 3 2 1 0 ppm
2.41
1.06
0.52
0.53
1.14
1.11
1.10
3.40
1.15
1.77
0.47
2.30
3.69
3.35

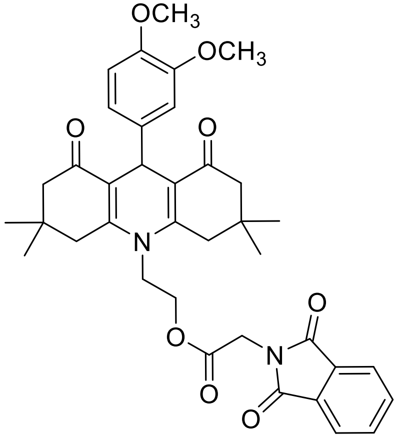

8h

Sample Information

Acquired by : System Administrator
Date Acquired : 01/12/2021 03:10:02 م
Sample Type : Unknown
Sample Name : DCP08
Sample ID :
Dilution Factor : 1
Tray# : 1
Vial# : 32
Injection Volume : 10
Data File : S_32.lcd
Method File : Method_MS_only.lcm
Original Method File : Method_MS_only.lcm
Report Format File : DEFAULT.lsr
Tuning File : default.lct
Processed by : System Administrator
Date Processed : 05/12/2021 09:48:33 ص

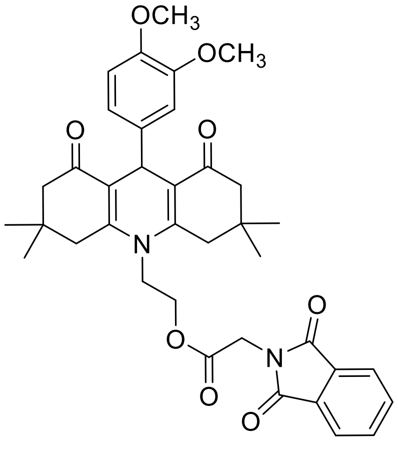

8h

MS Chromatogram

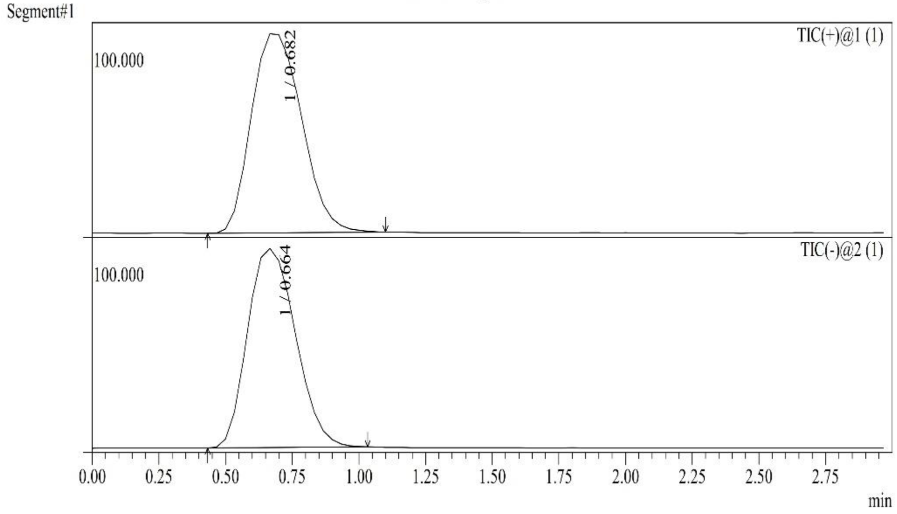

Line#:1 R.Time:0.667(Scan#:41)
MassPeaks:813
Spectrum Mode:Averaged 0.633-0.700(39-43) Base Peak:641(7299803)
BG Mode:Calc Segment 1 - Event 1

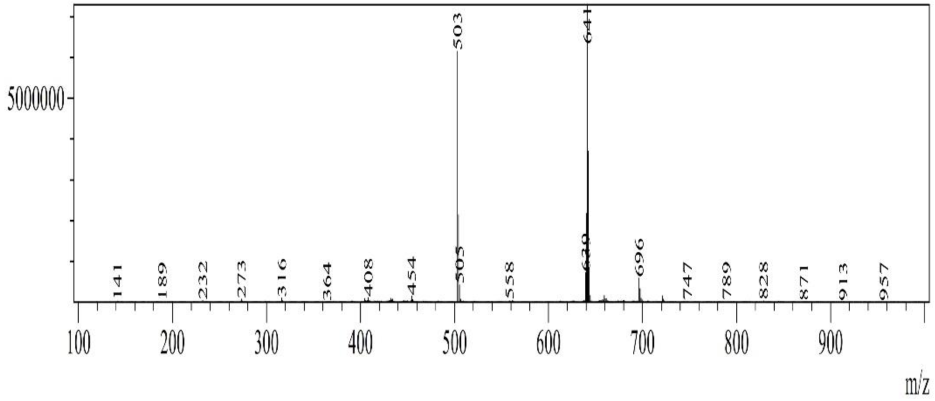

MASS Peak Table ALL MC

Peak#	Ret. Time	m/z	Area	Area%	Mark	A/H	Event#
1	0.682	TIC	392123408	100.000		13.163	1-1
2	0.664	TIC	767836676	100.000		12.548	1-2
Total			1159960084	200.000			

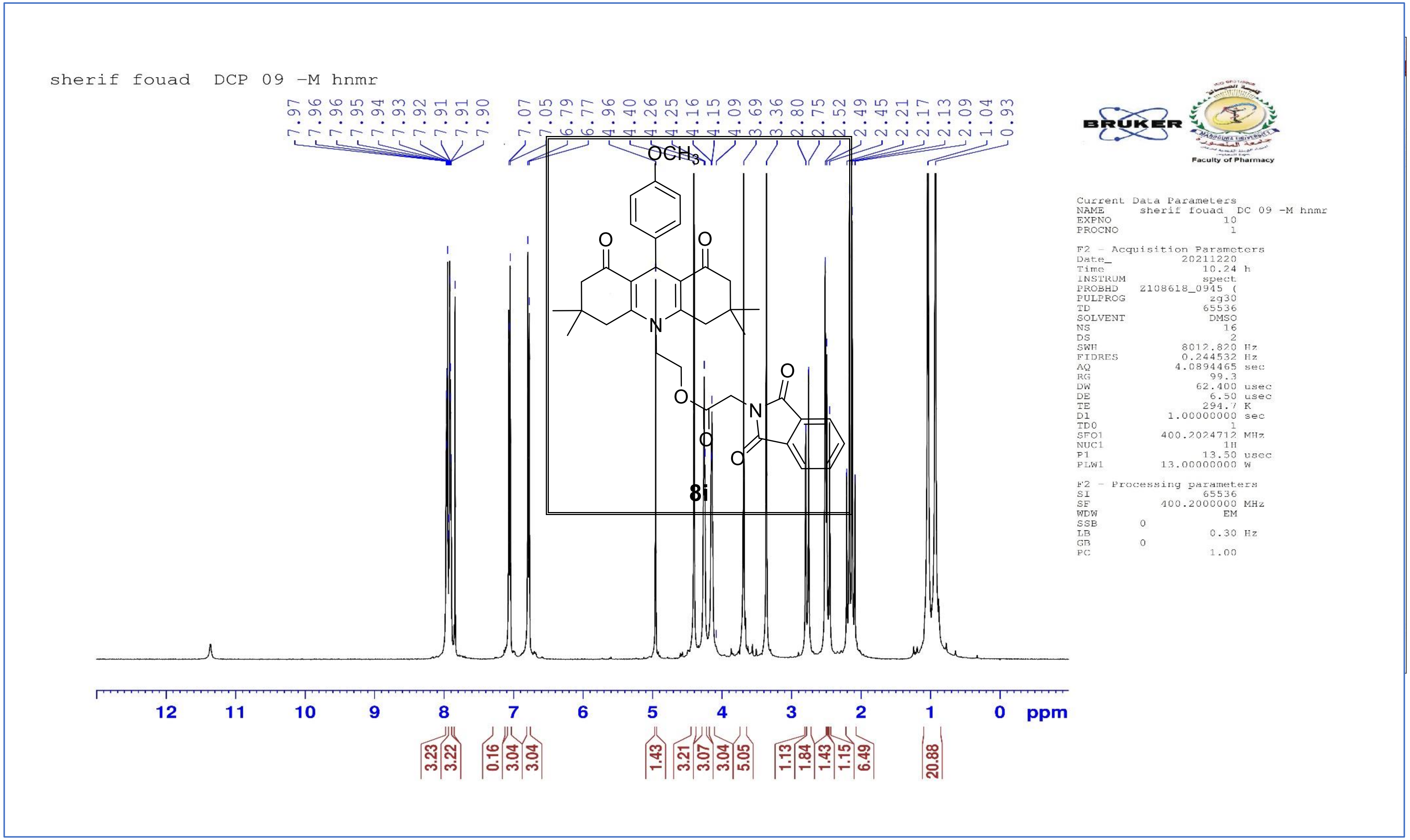
sherif fouad DCP 09 -M hnmr
7.97 7.96 7.96 7.95 7.94 7.93 7.92 7.91 7.91 7.90 7.07 7.05 6.79 6.77 4.96 4.40 4.26 4.25 4.16 4.15 4.09 3.69 3.36 2.80 2.75 2.52 2.49 2.45 2.21 2.17 2.13 2.09 1.04 0.93
BRUKER
Faculty of Pharmacy
Current Data Parameters
NAME sherif fouad DC 09 -M hnmr
EXPNO 10
PROCNO 1
F2 - Acquisition Parameters
Date_ 20211220
Time 10.24 h
INSTRUM spect
PROBHD Z108618_0945 (
PULPROG zg30
TD 65536
SOLVENT DMSO
NS 16
DS 2
SWH 8012.820 Hz
FIDRES 0.244532 Hz
AQ 4.0894465 sec
RG 99.3
DW 62.400 usec
DE 6.50 usec
TE 294.7 K
D1 1.00000000 sec
TD0 1
SFO1 400.2024712 MHz
NUC1 1H
P1 13.50 usec
PLW1 13.00000000 W
F2 - Processing parameters
SI 65536
SF 400.2000000 MHz
WDW EM
SSB 0
LB 0.30 Hz
GB 0
PC 1.00
OCH3
O
O
N
O
O
N
O
O
O
8i
12 11 10 9 8 7 6 5 4 3 2 1 0 ppm
3.23 3.22 0.16 3.04 3.04 1.43 3.21 3.07 3.04 5.05 1.13 1.84 1.43 1.15 6.49 20.88

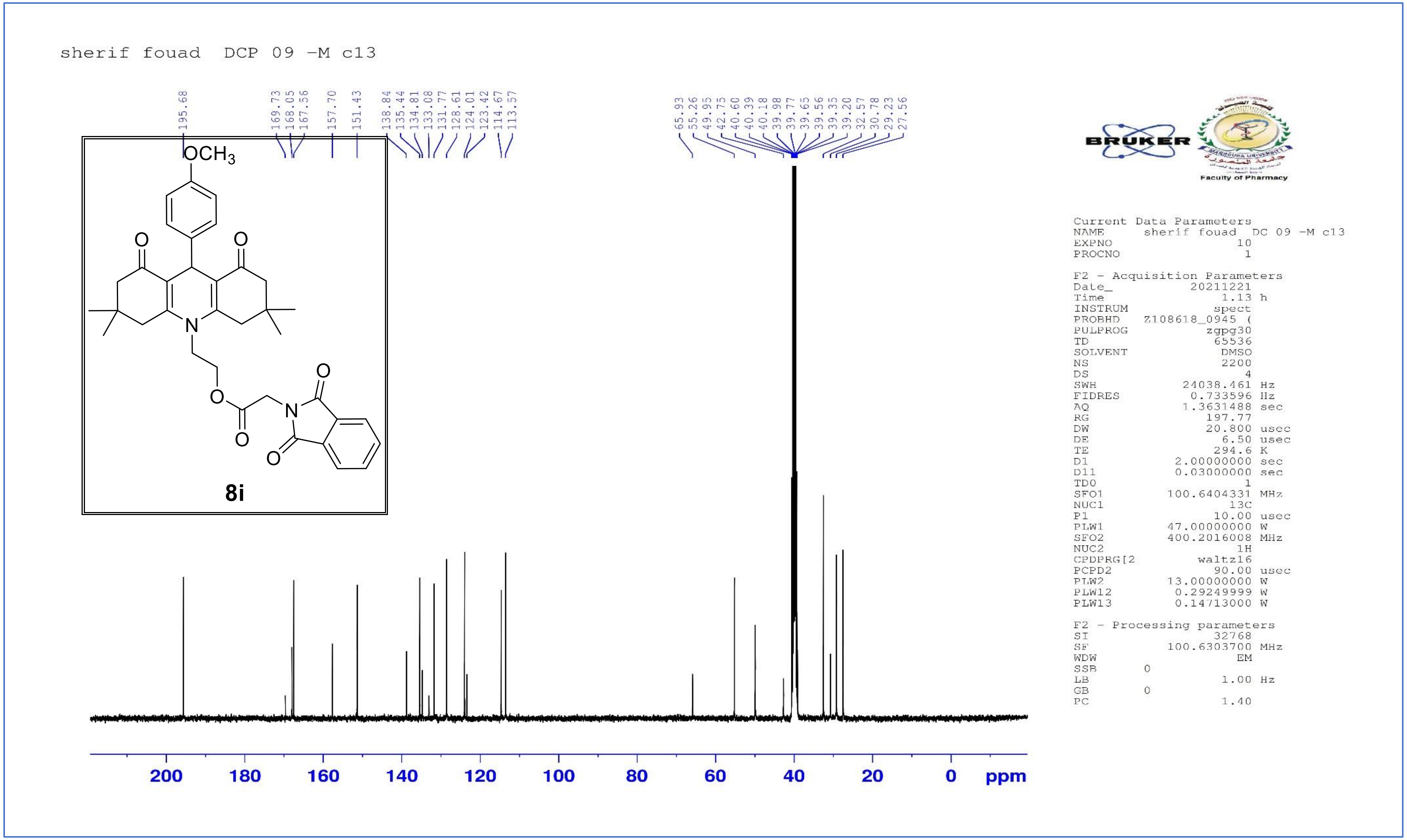
sherif fouad DCP 09 -M c13
195.68
169.73
168.05
167.56
157.70
151.43
138.84
135.44
134.81
133.08
131.77
128.61
124.01
123.42
114.67
113.57
65.93
55.26
49.95
42.75
40.60
40.39
40.18
39.98
39.77
39.65
39.56
39.35
39.20
32.57
30.78
29.23
27.56
OCH3
O
O
N
O
O
N
O
O
8i
BRUKER
Faculty of Pharmacy
Current Data Parameters
NAME sherif fouad DC 09 -M c13
EXPNO 10
PROCNO 1
F2 - Acquisition Parameters
Date_ 20211221
Time 1.13 h
INSTRUM spect
PROBHD Z108618_0945 (
PULPROG zgpg30
TD 65536
SOLVENT DMSO
NS 2200
DS 4
SWH 24038.461 Hz
FIDRES 0.733596 Hz
AQ 1.3631488 sec
RG 197.77
DW 20.800 usec
DE 6.50 usec
TE 294.6 K
D1 2.00000000 sec
D11 0.03000000 sec
TD0 1
SFO1 100.6404331 MHz
NUC1 13C
P1 10.00 usec
PLW1 47.00000000 W
SFO2 400.2016008 MHz
NUC2 1H
CPDPRG[2 waltz16
PCPD2 90.00 usec
PLW2 13.00000000 W
PLW12 0.29249999 W
PLW13 0.14713000 W
F2 - Processing parameters
SI 32768
SF 100.6303700 MHz
WDW EM
SSB 0
LB 1.00 Hz
GB 0
PC 1.40
200
180
160
140
120
100
80
60
40
20
0
ppm

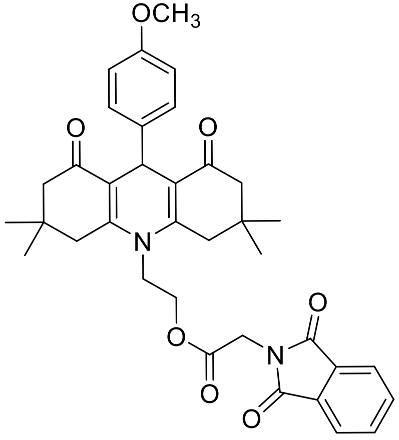

8i

Sample Information

Acquired by	: System Administrator
Date Acquired	: 01/12/2021 03:13:32 م
Sample Type	: Unknown
Sample Name	: DCP09
Sample ID	:
Dilution Factor	: 1
Tray#	: 1
Vial#	: 33
Injection Volume	: 10
Data File	: S_33.lcd
Method File	: Method_MS_only.lcm
Original Method File	: Method_MS_only.lcm
Report Format File	: DEFAULT.lsr
Tuning File	: default.lct
Processed by	: System Administrator
Date Processed	: 05/12/2021 09:49:02 ص

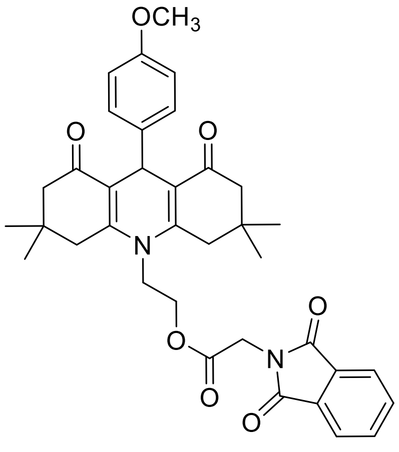

8i

MS Chromatogram

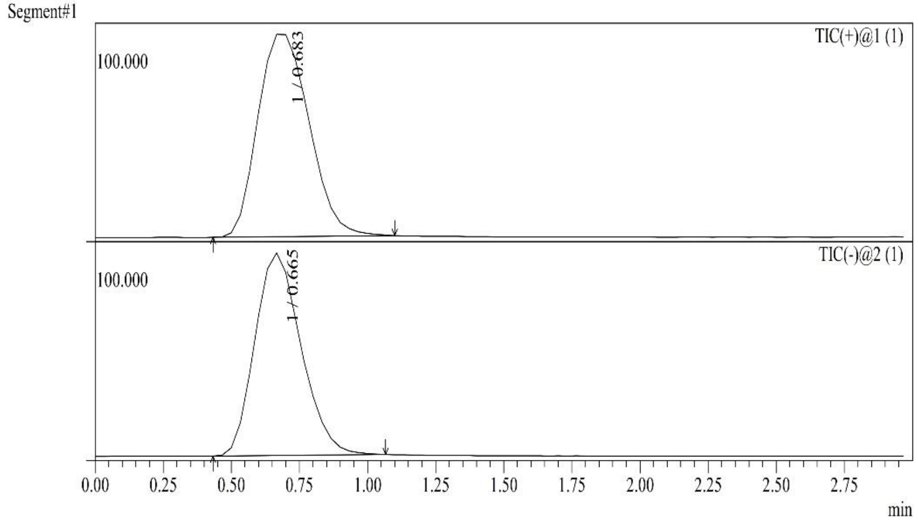

Line#:1 R.Time:0.667(Scan#:41)
MassPeaks:828
Spectrum Mode:Averaged 0.633-0.700(39-43) Base Peak:611(7023152)
BG Mode:Calc Segment 1 - Event 1

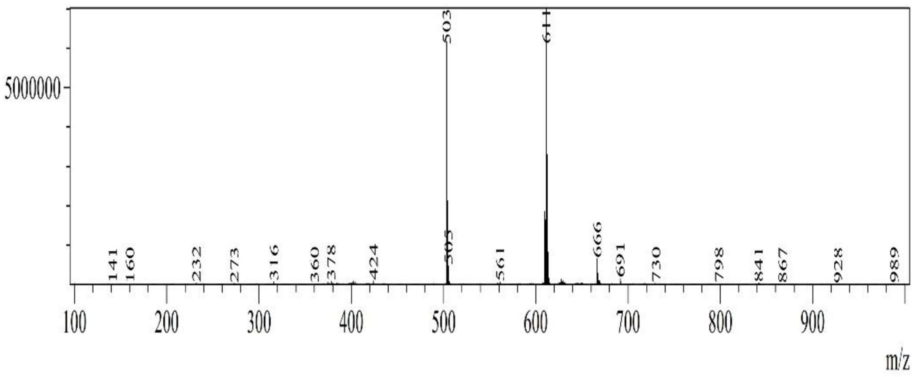

MASS Peak Table ALL MC

Peak#	Ret. Time	m/z	Area	Area%	Mark	A/H	Event#
1	0.683	TIC	386193662	100.000		13.109	1-1
2	0.665	TIC	694067939	100.000		11.801	1-2
Total			1080261601	200.000			

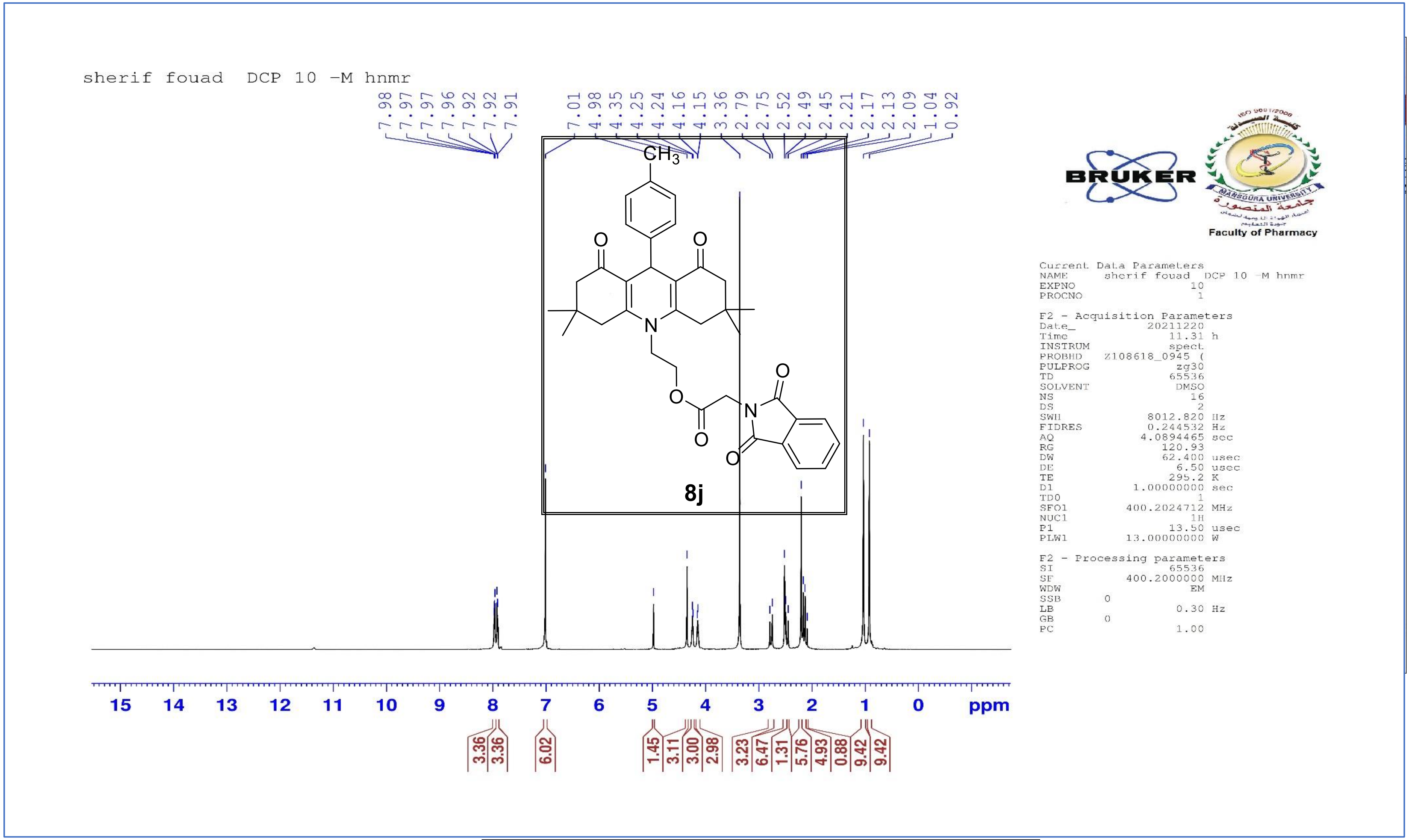

^{1}H NMR Spectrum of compound **8j**

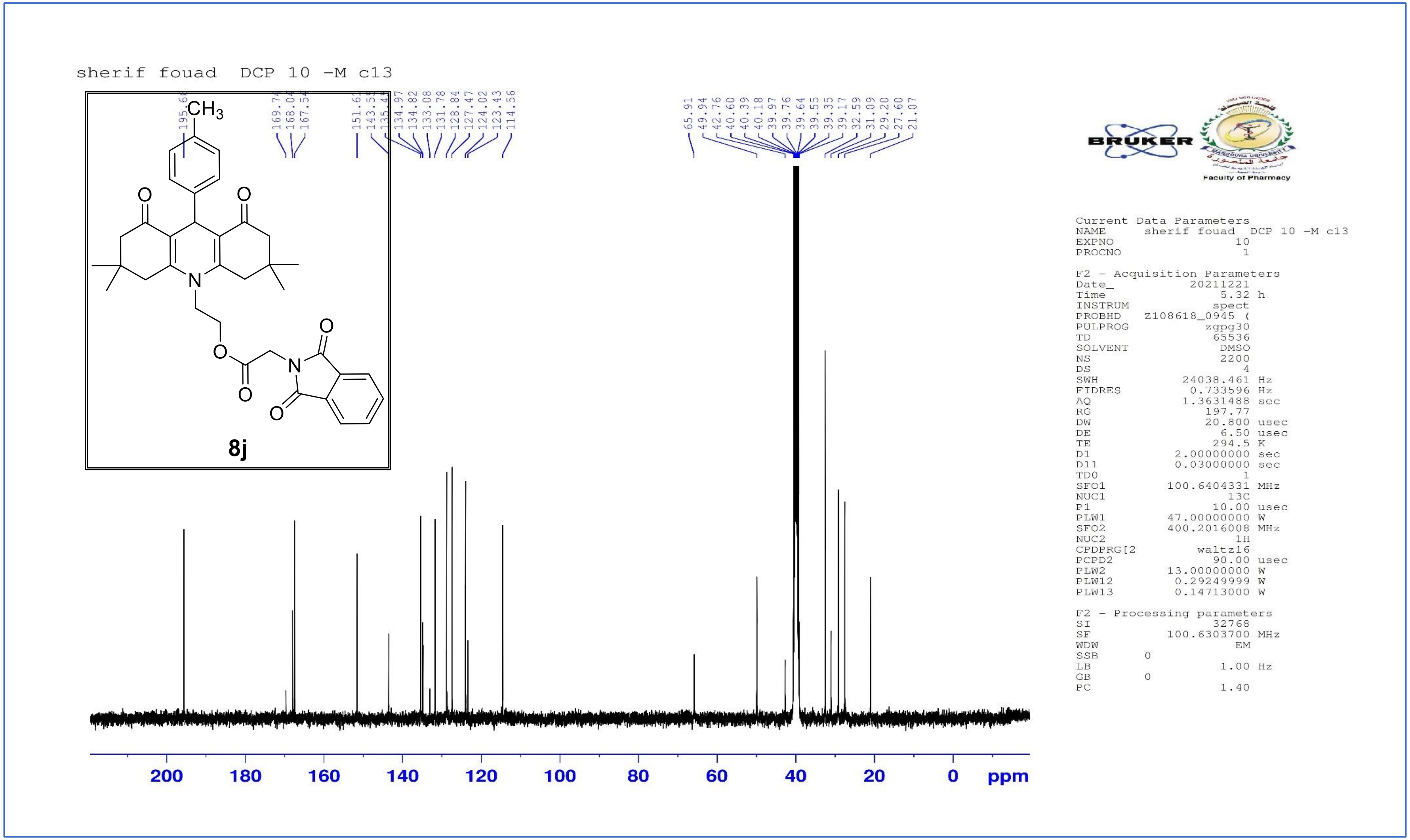

^{13}C NMR Spectrum of compound **8j**

Sample Information

Acquired by	: System Administrator
Date Acquired	: 01/12/2021 03:17:03 م
Sample Type	: Unknown
Sample Name	: DCP10
Sample ID	:
Dilution Factor	: 1
Tray#	: 1
Vial#	: 34
Injection Volume	: 10
Data File	: S_34.lcd
Method File	: Method_MS_only.lcm
Original Method File	: Method_MS_only.lcm
Report Format File	: DEFAULT.lsr
Tuning File	: default.lct
Processed by	: System Administrator
Date Processed	: 05/12/2021 09:49:20 ص

MS Chromatogram

Segment#1

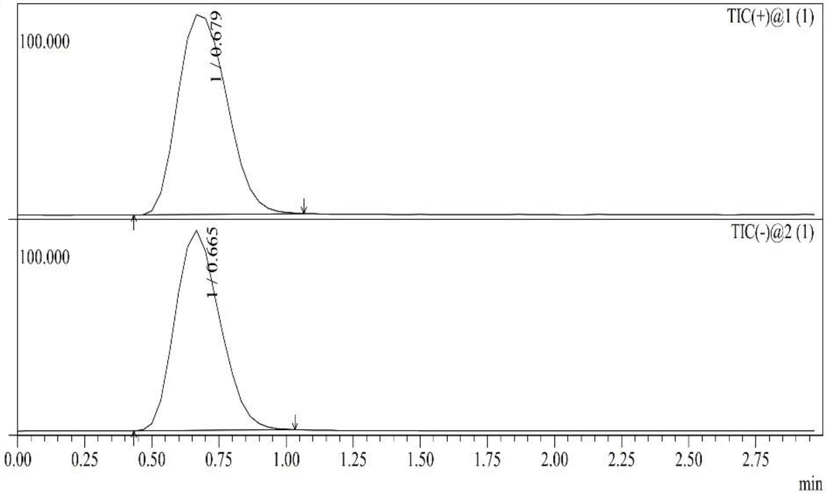

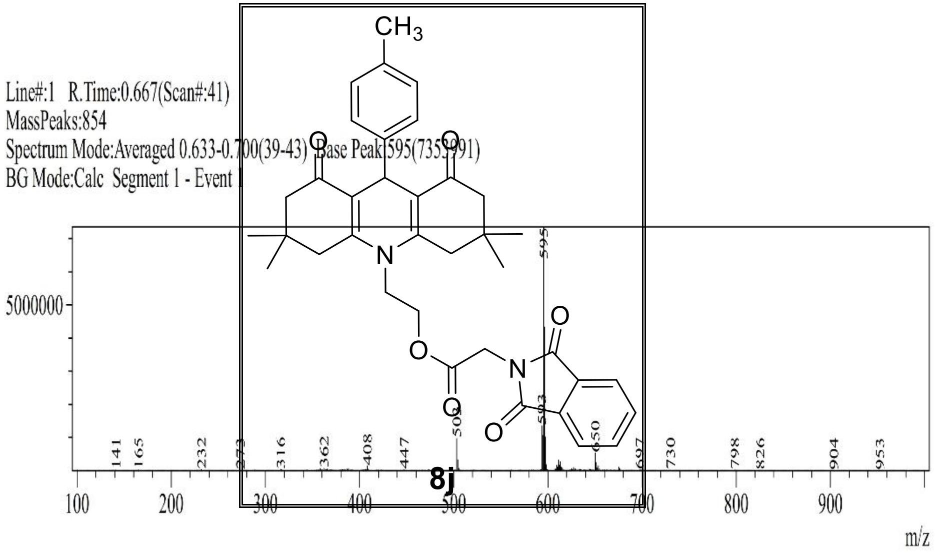

8j

MASS Peak Table ALL MC

Peak#	Ret. Time	m/z	Area	Area%	Mark	A/H	Event#
1	0.679	TIC	304766115	100.000		12.767	1-1
2	0.665	TIC	574757910	100.000		11.588	1-2
Total			879524025	200.000			

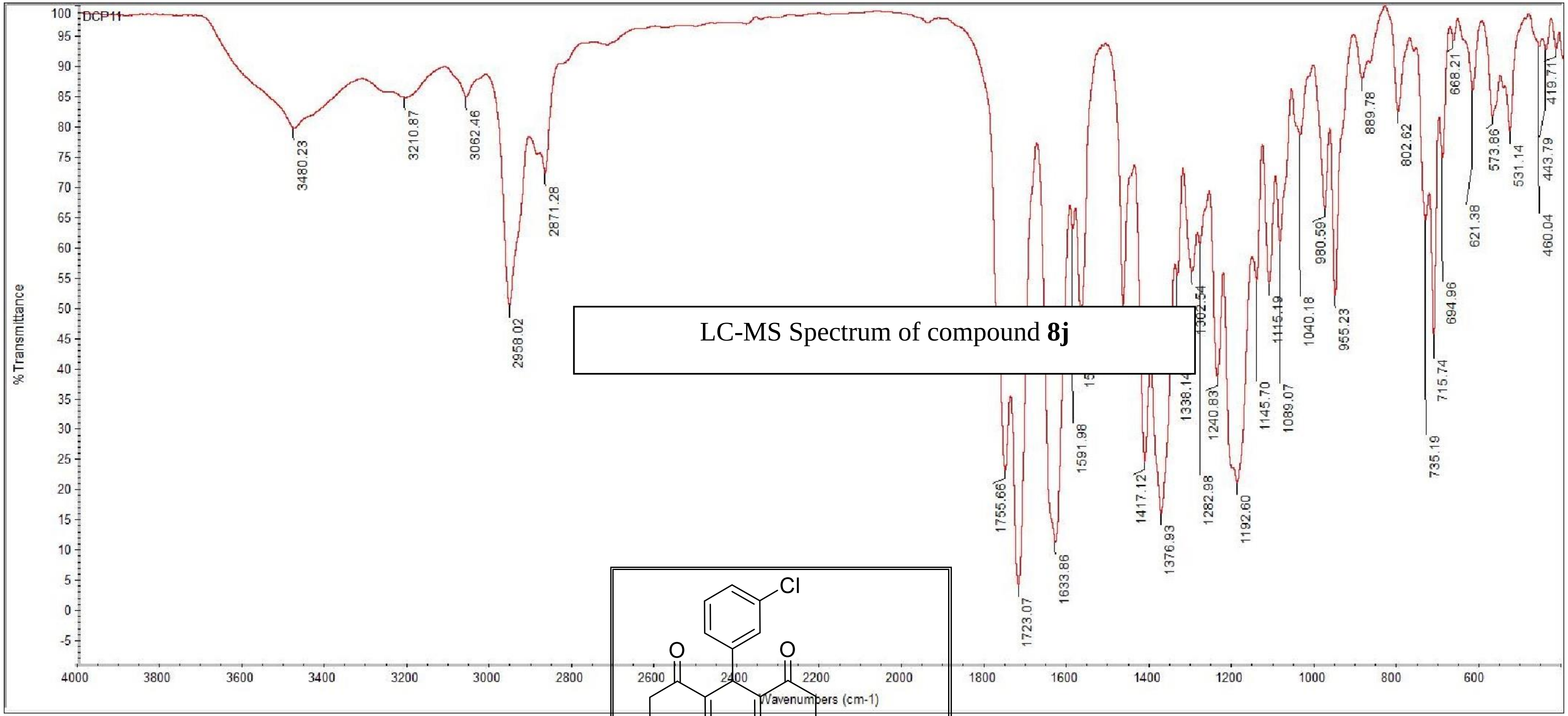

LC-MS Spectrum of compound **8j**

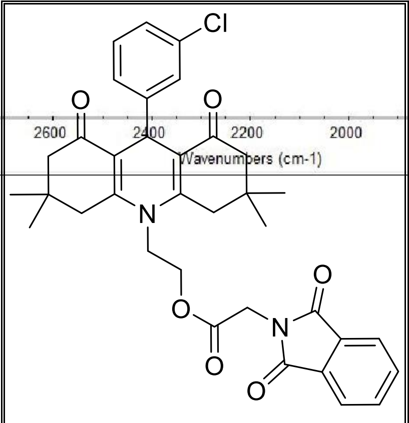

8k

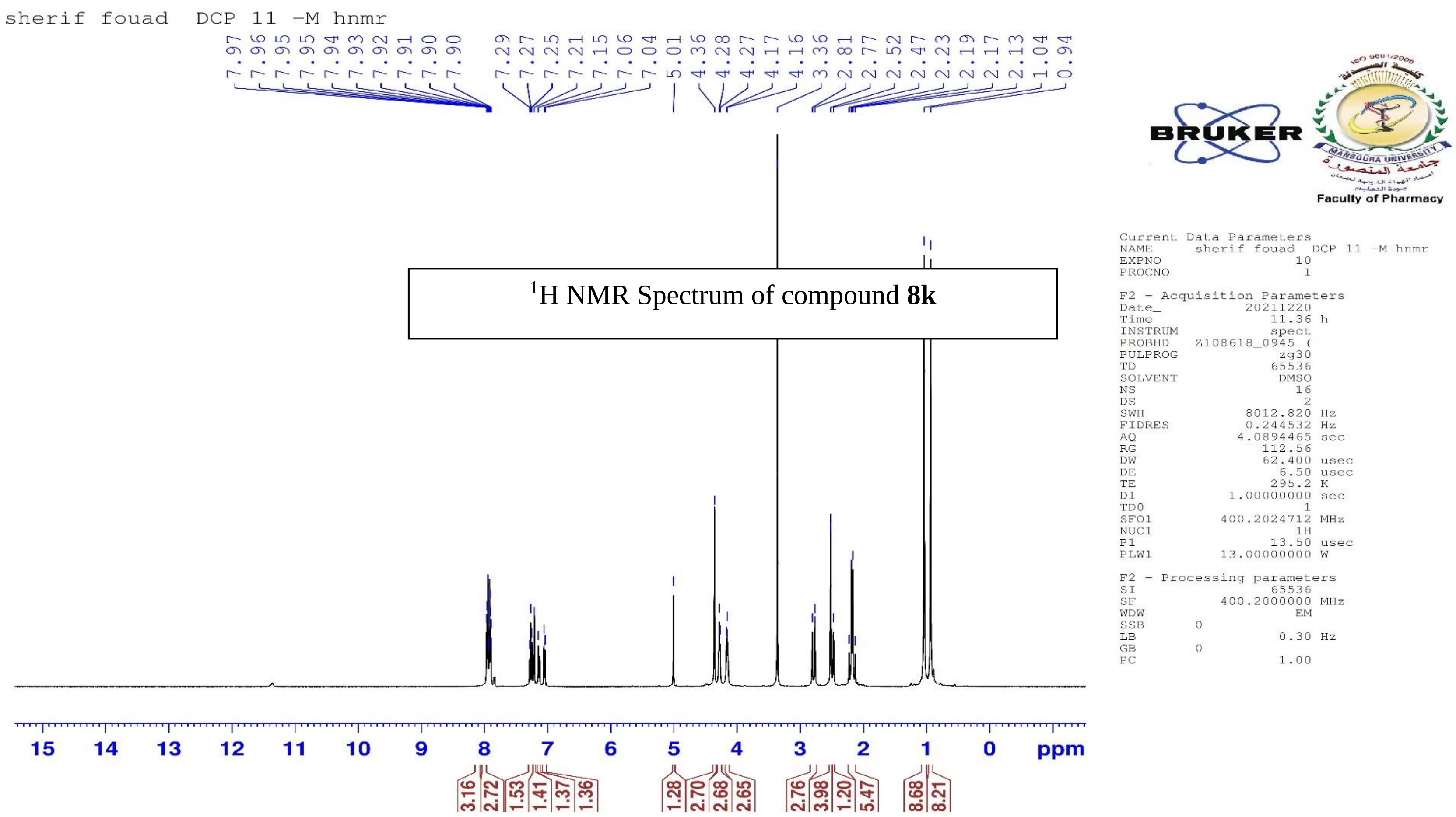

^{1}H NMR Spectrum of compound **8k**

Sample Information

Acquired by	: System Administrator
Date Acquired	: 01/12/2021 03:20:33 م
Sample Type	: Unknown
Sample Name	: DCP11
Sample ID	:
Dilution Factor	: 1
Tray#	: 1
Vial#	: 35
Injection Volume	: 10
Data File	: S_35.lcd
Method File	: Method_MS_only.lcm
Original Method File	: Method_MS_only.lcm
Report Format File	: DEFAULT.lsr
Tuning File	: default.lct
Processed by	: System Administrator
Date Processed	: 05/12/2021 09:49:38 ص

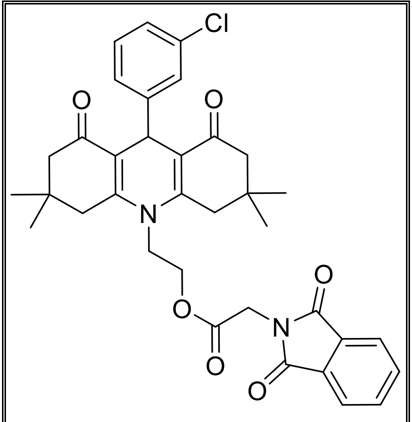

8k

MS Chromatogram

Segment#1

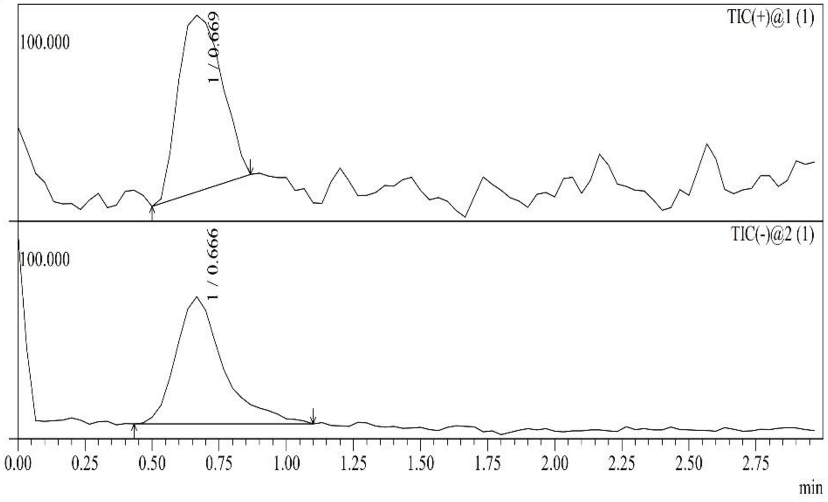

Line#:1 R.Time:0.667(Scan#:41)
MassPeaks:500
Spectrum Mode:Averaged 0.633-0.700(39-43) Base Peak:615(36310)
BG Mode:Calc Segment 1 - Event 1

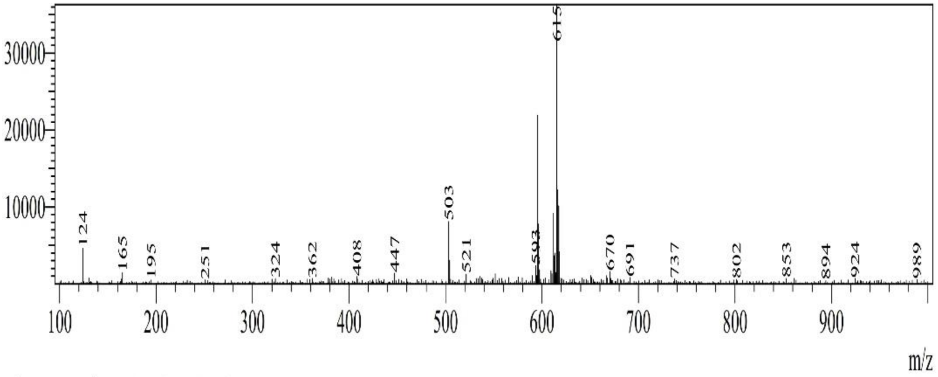

MASS Peak Table ALL MC

Peak#	Ret. Time	m/z	Area	Area%	Mark	A/H	Event#
1	0.669	TIC	2055071	100.000		11.512	1-1
2	0.666	TIC	4163958	100.000		12.269	1-2
Total			6219029	200.000			

LC-MS Spectrum of compound **8k**

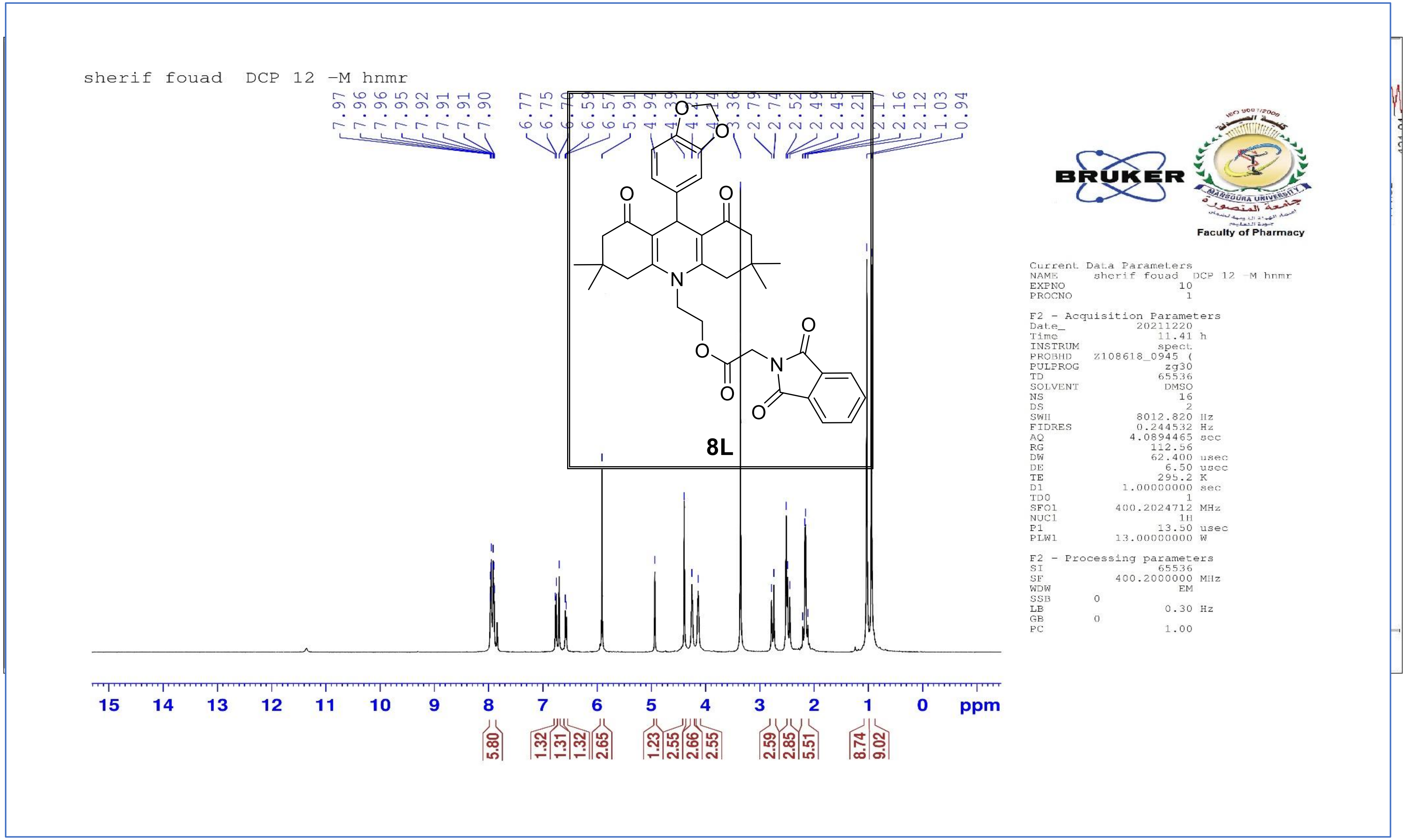

^{1}H NMR Spectrum of compound **8L**

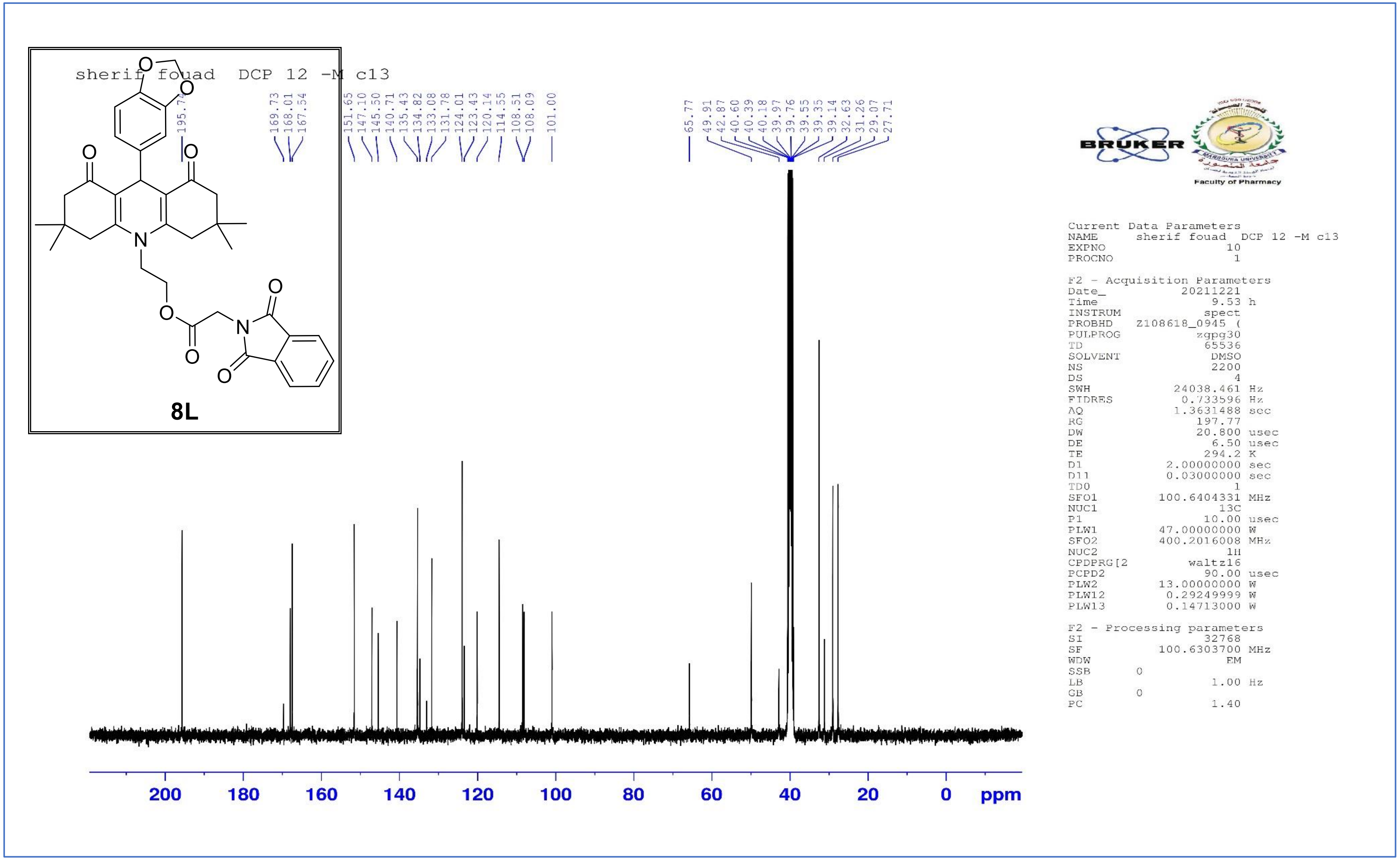

^{13}C NMR Spectrum of compound **8L**

Sample Information

Acquired by	: System Administrator
Date Acquired	: 01/12/2021 03:24:04 م
Sample Type	: Unknown
Sample Name	: DCP12
Sample ID	:
Dilution Factor	: 1
Tray#	: 1
Vial#	: 36
Injection Volume	: 10
Data File	: S_36.lcd
Method File	: Method_MS_only.lcm
Original Method File	: Method_MS_only.lcm
Report Format File	: DEFAULT.lsr
Tuning File	: default.lct
Processed by	: System Administrator
Date Processed	: 05/12/2021 09:50:02 ص

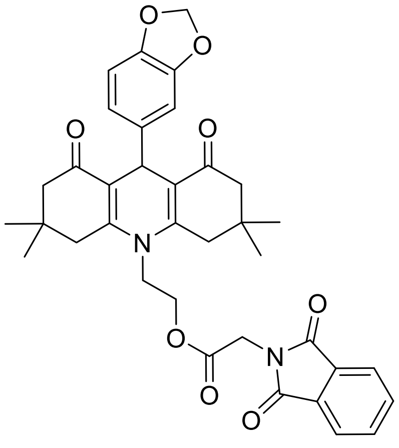

8L

MS Chromatogram

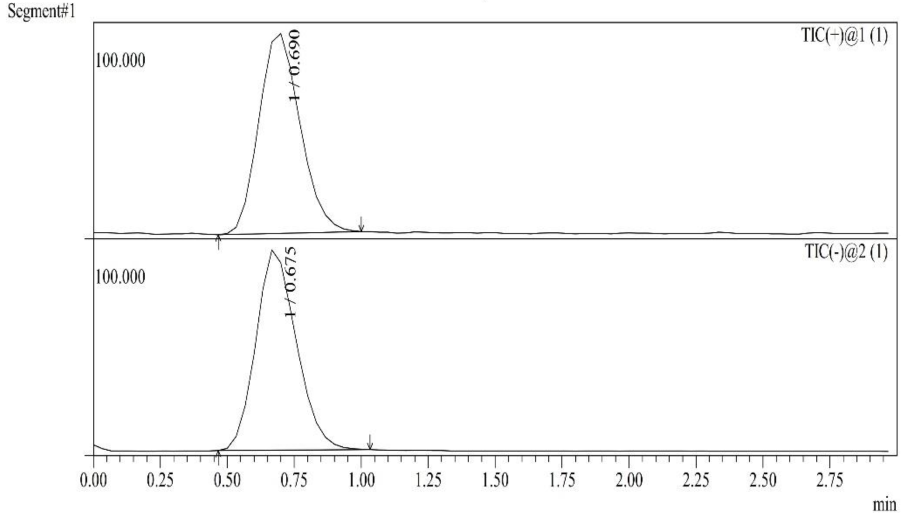

Line#:1 R.Time:0.667(Scan#:41)
MassPeaks:741
Spectrum Mode:Averaged 0.633-0.700(39-43) Base Peak:625(2032202)
BG Mode:Calc Segment 1 - Event 1

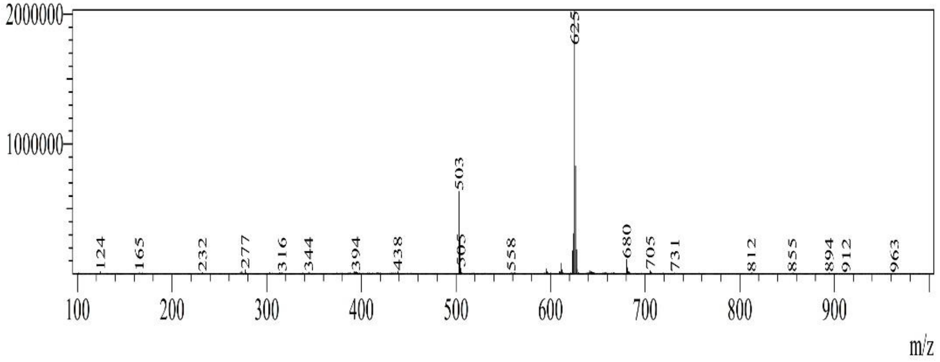

MASS Peak Table ALL MC

Peak#	Ret. Time	m/z	Area	Area%	Mark	A/H	Event#
1	0.690	TIC	68493244	100.000		10.735	1-1
2	0.675	TIC	119614493	100.000		10.501	1-2
Total			188107737	200.000			

LC-MS Spectrum of compound **8L**
